# Relationship between dietary fiber physicochemical properties and feedstuff fermentation characteristics and their effects on nutrient utilization, energy metabolism, and gut microbiota in growing pigs

**DOI:** 10.1186/s40104-024-01129-x

**Published:** 2025-01-02

**Authors:** Feng Yong, Bo Liu, Huijuan Li, Houxu Hao, Yueli Fan, Osmond Datsomor, Rui Han, Hailong Jiang, Dongsheng Che

**Affiliations:** Key Laboratory of Animal Production, Product Quality and Security, Ministry of Education, Jilin Provincial Key Laboratory of Animal Nutrition and Feed Science, Jilin Provincial Science and Technology Innovation Center of Pig industry Technology, College of Animal Science and Technology, Jilin Agricultural University, Changchun, China

**Keywords:** Dietary fiber, Energy metabolism, Fermentation characteristics, Growing pigs, Gut microbiota, Nutrient utilization, Physicochemical properties

## Abstract

**Background:**

There is a growing focus on using various plant-derived agricultural by-products to increase the benefits of pig farming, but these feedstuffs are fibrous in nature. This study investigated the relationship between dietary fiber physicochemical properties and feedstuff fermentation characteristics and their effects on nutrient utilization, energy metabolism, and gut microbiota in growing pigs.

**Methods:**

Thirty-six growing barrows (47.2 ± 1.5 kg) were randomly allotted to 6 dietary treatments with 2 apparent viscosity levels and 3 β-glucan-to-arabinoxylan ratios. In the experiment, nutrient utilization, energy metabolism, fecal microbial community, and production and absorption of short-chain fatty acid (SCFA) of pigs were investigated. In vitro digestion and fermentation models were used to compare the fermentation characteristics of feedstuffs and ileal digesta in the pig’s hindgut.

**Results:**

The production dynamics of SCFA and dry matter corrected gas production of different feedstuffs during in vitro fermentation were different and closely related to the physical properties and chemical structure of the fiber. In animal experiments, increasing the dietary apparent viscosity and the β-glucan-to-arabinoxylan ratios both increased the apparent ileal digestibility (AID), apparent total tract digestibility (ATTD), and hindgut digestibility of fiber components while decreasing the AID and ATTD of dry matter and organic matter (*P* < 0.05). In addition, increasing dietary apparent viscosity and β-glucan-to-arabinoxylan ratios both increased gas exchange, heat production, and protein oxidation, and decreased energy deposition (*P* < 0.05). The dietary apparent viscosity and β-glucan-to-arabinoxylan ratios had linear interaction effects on the digestible energy, metabolizable energy, retained energy (RE), and net energy (NE) of the diets (*P* < 0.05). At the same time, the increase of dietary apparent viscosity and β-glucan-to-arabinoxylan ratios both increased SCFA production and absorption (*P* < 0.05). Increasing the dietary apparent viscosity and β-glucan-to-arabinoxylan ratios increased the diversity and abundance of bacteria (*P* < 0.05) and the relative abundance of beneficial bacteria. Furthermore, increasing the dietary β-glucan-to-arabinoxylan ratios led to a linear increase in SCFA production during the in vitro fermentation of ileal digesta (*P* < 0.001). Finally, the prediction equations for RE and NE were established.

**Conclusion:**

Dietary fiber physicochemical properties alter dietary fermentation patterns and regulate nutrient utilization, energy metabolism, and pig gut microbiota composition and metabolites.

**Supplementary Information:**

The online version contains supplementary material available at 10.1186/s40104-024-01129-x.

## Background

The transition from traditional dietary preparation patterns (corn and soybean meal) to diversified dietary preparation patterns (i.e., diets composed of multiple grains and other plant-derived agricultural by-products) is an inevitable trend for the future sustainability and development of the pig industry [1–3]. However, apart from a few conventional feedstuffs, the application of most non-conventional feedstuffs (agricultural by-products) in the pig industry has been limited due to their richness in fiber [4]. Studies have shown that the fermentation capacity of dietary fiber in pig hindgut and its effect on nutrient utilization depends on its physical properties and chemical structure [5, 6]. Therefore, regulating the physicochemical properties of fibers in diets, especially when incorporating higher levels of fibers into the diets, may contribute to the further utilization of fibrous agricultural by-products.

Dietary fiber, as the most widely distributed and abundant renewable energy substance in plant cell walls, can be partially or entirely fermented in the hindgut of monogastric animals to produce metabolites such as short-chain fatty acids (SCFA) and gases and thus participate in host energy metabolism [7, 8]. When more fibrous agricultural by-products are incorporated into pig diets, the carbohydrate composition inevitably shifts from high starch to low starch and high dietary fiber, thus changing the structure of energy supply. Although the classification system of soluble dietary fiber (SDF) and insoluble dietary fiber (IDF) helps to reveal potential mechanisms of fiber’s effects on nutrient digestion and metabolism in pigs [9, 10], recent studies on the fermentation characteristics of fiber have found the opposite result, with in vivo studies showing that the concentration of SCFA in growing pig feces is related to IDF but not SDF [11, 12]. This suggests that a simple solubility perspective is insufficient to explain the interaction between fiber and nutrients in the intestine [13]. Therefore, it is necessary to elucidate further the specific relationship between the fermentation characteristics of feedstuffs and their fiber physicochemical properties.

In this study, we first evaluated the fermentation characteristics of 13 conventional and non-conventional feedstuffs by in vitro digestion and fermentation modeling in growing pigs and explored the relationship between them and the physicochemical properties of dietary fiber. Subsequently, based on in vitro outcome, 6 diets were formulated using dietary apparent viscosity and β-glucan-to-arabinoxylan ratios as factors, and the effects of dietary fiber physicochemical properties on nutrient utilization, energy metabolism and gut microbiota in growing pigs were explored. Additionally, ileal digesta was used as a substrate for in vitro fermentation, which further verified the regulating effect of fiber physicochemical properties on dietary fermentation efficiency. Finally, prediction equations for retained energy (RE) and net energy (NE) were established by combining diets’ chemical components and fiber physicochemical properties.

On the whole, the objective of the present study was to explore the relationship between dietary fiber physicochemical properties and feedstuff fermentation characteristics and their effects on nutrient utilization, energy metabolism, and gut microbiota in growing pigs.

## Materials and methods

### Feedstuffs

Thirteen conventional and unconventional feedstuffs, including corn, barley, wheat, soybean meal, rapeseed meal, sugar beet pulp, corn gluten meal, corn distillers dried grains with solubles (corn DDGS), corn germ meal, corn husk, soybean husk, wheat bran, and alfalfa meal, were used in this study. These feedstuffs were selected based on their nutrient profiles and potential use in pig diets, and their nutrients and physical properties were quantified (Table 1).

**Table 1:** Nutrients and physical properties of the feedstuffs (as-fed basis)

Item	Corn	Wheat	Barley	Corn husk	Wheat bran	Soybean husk	Soybean meal	Rapeseed meal	Sugar beet pulp	Corn germ meal	Corn gluten meal	Corn DDGS	Alfalfa meal
DM, %	89.80	88.85	90.74	94.38	93.58	93.58	91.94	92.95	93.29	92.99	94.20	90.73	93.38
OM, %	88.78	87.39	88.45	91.17	88.86	89.33	85.50	85.34	87.91	92.26	92.76	86.45	81.79
EE, %	3.75	1.18	2.83	3.30	4.56	1.47	1.57	2.27	0.54	3.22	0.93	4.51	1.66
CP, %	10.09	12.78	11.53	8.11	17.37	9.84	49.79	38.20	9.88	17.56	57.66	28.26	18.06
Ash, %	1.02	1.46	2.29	3.21	4.72	4.25	6.44	7.61	5.38	0.73	1.44	4.28	11.59
CF, %	2.41	2.65	5.45	21.60	11.11	40.25	6.14	10.77	28.93	14.56	3.07	11.65	27.10
NFE, %	72.19	70.66	67.62	55.47	54.88	37.78	30.34	40.03	47.29	56.91	26.11	47.11	35.02
TDF, %	12.09	11.91	20.45	57.83	39.98	54.39	18.59	32.23	69.51	47.61	8.23	32.66	45.66
SDF, %	1.39	1.66	5.57	6.17	4.74	9.61	2.44	7.83	29.30	5.81	1.60	3.76	8.44
IDF, %	10.24	9.73	14.66	51.66	35.86	44.78	16.15	24.4	40.21	41.43	6.63	29.90	37.22
SDF/IDF	0.14	0.17	0.38	0.12	0.13	0.21	0.15	0.32	0.73	0.14	0.24	0.13	0.23
ADF, %	2.62	2.25	5.66	14.14	10.98	45.95	5.37	22.15	22.33	13.94	1.35	10.25	31.51
NDF, %	11.33	16.27	23.72	49.47	37.05	64.00	12.09	32.61	37.53	48.79	4.54	30.07	43.29
Cellulose, %	1.88	0.86	4.36	7.45	8.17	40.36	4.52	14.14	20.25	9.31	0.74	5.21	26.10
Lignin, %	0.74	1.39	1.30	6.69	2.81	5.59	0.85	7.75	2.08	4.63	0.61	5.04	5.41
NSP, %	11.35	10.52	19.15	51.14	37.17	48.80	17.74	24.48	67.43	42.98	7.62	27.62	40.25
β-Glucan, %	0.11	0.81	6.66	2.32	0.17	0.42	0.61	0.83	0.97	5.86	0.33	6.26	0.26
Arabinose, %	2.27	0.94	2.32	0.61	10.00	3.21	0.31	0.29	18.11	8.61	0.58	4.99	0.29
Xylose, %	3.27	3.24	3.17	3.25	3.10	3.14	3.51	3.25	2.98	3.02	3.20	3.28	3.19
Arabinoxylan, %	6.20	8.62	7.90	19.78	12.15	9.26	2.32	1.72	19.87	18.59	1.25	13.12	1.20
Mannose, %	0.20	0.00	0.20	0.40	0.50	3.00	1.30	0.60	1.52	0.00	0.30	2.00	7.00
Galactose, %	0.63	0.31	0.19	1.83	0.83	2.95	3.82	1.66	6.45	2.03	0.66	5.45	1.41
Glucose, %	2.57	2.48	1.20	1.27	0.83	0.31	0.74	2.07	1.23	1.52	1.90	1.69	1.37
Rhamnose, %	-	-	-	-	3.76	0.83	0.70	1.96	1.35	1.49	-	-	2.25
Uronic acid, %	0.71	0.21	0.20	3.84	1.41	1.97	5.00	6.64	33.26	3.43	0.51	5.10	2.31
Fructan, %	0.18	1.48	0.40	1.91	1.96	1.04	-	-	0.94	0.42	0.20	0.12	0.57
β-Glucan-to-arabinoxylan ratios	0.02	0.09	0.84	0.12	0.01	0.05	0.26	0.48	0.05	0.32	0.27	0.48	0.22
Ca, %	0.02	0.06	0.03	0.15	0.09	0.97	0.39	0.75	0.81	0.16	0.02	0.10	1.14
TP, %	0.58	0.21	0.34	0.50	0.81	0.28	0.66	0.87	0.15	0.68	0.09	0.73	0.30
GE, MJ/kg	16.51	15.88	16.55	18.17	17.36	15.82	17.67	17.70	15.96	18.12	20.81	18.17	16.06
Viscosity, cP	1.07	1.12	1.31	1.14	1.10	1.25	1.00	1.32	2.12	1.14	1.10	1.02	1.26
WHC, g/g	2.48	1.76	2.84	4.04	3.93	5.64	3.90	3.46	8.81	4.62	3.71	4.38	4.55
SC, mL/g	1.68	0.50	1.66	3.25	3.28	3.99	3.38	2.25	6.91	3.68	2.48	1.22	2.81
BW, g/L	487.80	598.90	492.80	404.40	388.40	456.90	636.90	547.00	643.40	457.30	458.10	426.30	358.10

*DM* Dry matter, *OM* Organic matter, *EE* Ether extract, *CP* Crude protein, *CF* Crude fiber, *NFE* Nitrogen-free extract, *TDF* Total dietary fiber, *SDF* Soluble dietary fiber, *IDF* Insoluble dietary fiber, *ADF* Acid detergent fiber, *NDF* Neutral detergent fiber, *NSP* Non-starch polysaccharides, *TP* Total phosphorus, *GE* Gross energy, *WHC* Water holding capacity, *SC* Swelling capacity, *BW* Bulk weight, *Corn DDGS* Corn distillers dried grains with solubles

### Exp. 1

#### In vitro digestion

The 3-step in vitro digestion technique simulates digestion activities occurring in the upper gastrointestinal tract of pigs and provides information on the in vitro apparent ileal digestibility of dry matter (DM) and gross energy (GE; Table S1).

The feedstuffs were ground and passed through a 1-mm sieve for uniformity using a water mill (HBM-103B, Zhejiang, China; 2,840 r/min). According to the method described by Minekus et al. [14], 8 ± 0.0001 g of feedstuff samples were placed in 16 mL of saliva α-amylase solution (75 U/mL, Sigma 10065; Sigma Aldrich, Darmstadt, Germany) at 39 °C for 2 min using a constant-temperature shaker (ZQTY-50E, Shanghai, China) to simulate oral digestion. After the oral simulation, in vitro digestion of the stomach and small intestine was performed according to the method described by Chen et al. [15]. Briefly, 560 mL of 0.1 mol/L disodium phosphate buffer and 16 mL of 10 g/L pepsin solution (P-7000; Sigma Aldrich, Darmstadt, Germany) was added on the basis of oral digestion, then the pH of the final solution adjusted to 3.5 with HCl, and agitated for 2 h under the same conditions to simulate gastric phase digestion. The digestion in the small intestinal was simulated by adding 240 mL of 0.2 mol/L disodium phosphate buffer, 16 mL of freshly prepared pancreatic enzyme solution (0.1 g/mL, Sigma P1750; Sigma Aldrich, Darmstadt, Germany), 27.5 mg/mL starch glucosidase solution (Sigma 10113; Sigma Aldrich, Darmstadt, Germany), and 8 mL 150 g/L bile salt solution (Sigma 48305; Sigma Aldrich, Darmstadt, Germany), with the pH adjusted to 6.8 using NaOH, and agitated for 4 h. After in vitro digestion, the residues were washed with 95% ethanol and collected by filtration through nylon cloth (40 μm) [16]. In vitro digestion of the feedstuff samples was repeated to obtain sufficient samples for analysis (DM and GE) and in vitro fermentation.

#### In vitro fermentation

The basal fermentation medium was prepared according to the method described by Menke et al. [17]. Ten healthy, near-weight growing barrows (Duroc × Landrace × Yorkshire) with an average body weight of 45.0 ± 1.0 kg were selected as fecal donors. These pigs were fed a standard commercial diet to meet their growth requirements and had not received any antibiotics for at least one month before the experiment. Fresh feces were collected from each pig using the rectal collection method, and the storage method was performed as previously described [18]. The fecal inoculum was prepared based on a previous report [19], with minor modifications. Briefly, feces were mixed with sterile preheated saline (39 °C) to produce a 20% (w/v) fecal slurry. The fecal slurry was filtered through four layers of sterile gauze, and the filtrate was used as fecal inoculum. The process was done in a sterile environment and was completed over a relatively short period.

The feedstuff residue after in vitro digestion (dried residue, dried for 12 h at 65 °C) was weighed to 200 mg and transferred into 50-mL sterile fermentation tubes. A 2 mL fecal inoculum and 28 mL basal medium were added, and the tubes were continuously flushed with CO_2_ for 10 s, and sealed immediately to maintain anaerobic conditions. The tubes were then transferred into a constant temperature shaker set at 39 ± 0.25 °C and a speed of 120 r/min for incubation. All processes were performed under aseptic conditions and completed within a short time (finish in 2 min). All feedstuffs were subjected to in vitro batch fermentation, with 6 replicates at each fermentation time point. During the period of in vitro fermentation from 0 to 24 h, every 3 h, and then at 36, 48, 60, and 72 h, the fermentation tubes were removed and placed on ice to terminate the fermentation. The fermentation broth was removed and stored at −20 °C to determine SCFA production later.

#### Gas production kinetics

An ANKOM RFS gas production system (Ankom Technology Corp., Fairport, NY, USA) was used for the in vitro fermentation. The experimental conditions were expanded fourfold based on the in vitro fermentation scheme described above. Briefly, 800 mg feedstuffs residue, 112 mL basal medium, and 8 mL inoculum were placed in a fermentation flask, which was continuously flushed with CO_2_ for 30 s and then immediately capped and incubated in a constant air bath incubator. Simultaneously, the GPM software was used to monitor and record gas production, which was determined based on the following equation:

- *V*_*x*_ = *V*_*j*_ × *P*_*psi*_ × 0.068004084
- *V*_*j*_ = headspace volume in the glass bottle (mL)
- *V*_*x*_ = gas produced (mL)
- *P*_*psi*_ = gas pressure (psi)

### Exp. 2

This study was conducted under the Animal Care and Use Committee guidelines of the Jilin Agricultural University, Jilin Province, China. All experimental procedures followed the Guidelines for the Care and Use of Experimental Animals of the Jilin Agricultural University. This animal study was approved by the Ethics Committee of Jilin Agricultural University (approval number: KT2023006).

#### Experimental design, animals and diets

A 2 × 3 factorial treatment arrangement was explored for this study, with the main factors being 2 different apparent viscosity levels and 3 different β-glucan-to-arabinoxylan ratios. Thirty-six growing barrows (Duroc × Landrace × Yorkshire) with similar body weight (47.2 ± 1.5 kg) were randomly allotted into 6 dietary treatment groups. The 6 dietary treatment groups were namely, (1) low apparent viscosity and low β-glucan-to-arabinoxylan ratios (L_V_L_β/AX_); (2) low apparent viscosity and medium β-glucan-to-arabinoxylan ratios (L_V_M_β/AX_); (3) low apparent viscosity and high β-glucan-to-arabinoxylan ratios (L_V_H_β/AX_); (4) high apparent viscosity and low β-glucan-to-arabinoxylan ratios (H_V_L_β/AX_); (5) high apparent viscosity and medium β-glucan-to-arabinoxylan ratios (H_V_M_β/AX_); and (6) high apparent viscosity and high β-glucan-to-arabinoxylan ratios (H_V_H_β/AX_). The experimental diets and their nutritional compositions are presented in Table 2. All experimental diet formulations followed the recommendations of NRC (2012) for the nutritional requirements of growing pigs and maintained relative consistency in NE, standardized ileal digestible crude protein (SID CP), and total dietary fiber (TDF) levels [20].

**Table 2:** Ingredients and chemical composition of experimental diets^a^

Item	L_V_L_β/AX_	L_V_M_β/AX_	L_V_H_β/AX_	H_V_L_β/AX_	H_V_M_β/AX_	H_V_H_β/AX_
Ingredient (as-fed basis), %						
Corn	29.00	18.70	8.90	-	-	-
Barley	20.00	29.50	39.00	24.00	35.00	46.00
Wheat	8.00	10.00	12.00	38.96	28.44	17.96
Soybean meal	3.00	2.35	1.70	2.00	3.60	5.20
Rapeseed meal	-	-	-	-	4.00	8.00
Corn gluten meal	3.00	1.75	-	4.00	2.00	-
Corn DDGS	5.00	11.75	18.50	-	-	-
Corn husk	9.82	9.90	10.00	-	-	-
Soybean husk	12.00	6.00	-	4.00	2.00	-
Sugar beet pulp	-	-	-	16.00	14.00	12.00
Soybean oil	4.80	4.65	4.50	5.20	5.35	5.50
Salt	0.80	0.80	0.80	0.80	0.80	0.80
Limestone	1.50	1.65	1.80	1.50	1.50	1.50
Dicalcium phosphate	0.40	0.41	0.42	0.80	0.61	0.42
L-Lysine (98%)	0.77	0.75	0.73	0.75	0.66	0.57
L-Methionine (99%)	0.12	0.13	0.14	0.14	0.15	0.15
L-Cysteine (99%)	0.03	0.04	0.04	0.03	0.03	0.03
L-Threonine (98%)	0.31	0.31	0.31	0.33	0.33	0.32
L-Tryptophan (98%)	0.09	0.08	0.07	0.06	0.05	0.04
L-Isoleucine (99%)	0.11	0.09	0.06	0.07	0.06	0.05
L-Leucine (99%)	0.04	0.02	-	0.20	0.28	0.35
L-Valine (99%)	0.12	0.07	0.02	0.10	0.08	0.06
L-Histidine (99%)	0.03	0.02	0.01	0.06	0.05	0.04
L-Phenylalanine (99%)	0.06	0.03	-	-	0.01	0.01
TiO_2_	0.40	0.40	0.40	0.40	0.40	0.40
Vitamin and mineral premix^b^	0.60	0.60	0.60	0.60	0.60	0.60
Total	100.00	100.00	100.00	100.00	100.00	100.00
Calculated nutrition content (as dry matter basis), %^c^						
Net energy, MJ/kg	10.42	10.46	10.49	10.50	10.51	10.51
Metabolizable energy, MJ/kg	14.39	14.37	14.36	14.56	14.53	14.50
Gross energy, MJ/kg	17.12	17.12	17.12	16.70	16.82	16.95
Crude protein (SID)^d^	10.82	10.83	10.84	10.84	10.83	10.83
Crude protein	13.26	13.59	13.92	13.54	13.97	14.40
Ether extract	7.41	7.33	7.26	6.66	7.03	7.40
Carbohydrate	67.77	67.29	66.82	67.57	66.57	65.56
Crude fiber	9.79	8.39	6.99	8.83	8.23	7.63
Nitrogen-free extract	54.13	54.91	55.69	54.51	54.40	54.28
Ash	2.25	2.35	2.44	2.40	2.66	2.93
Total dietary fiber	23.01	22.78	22.56	23.28	23.26	23.24
β-Glucan-to-arabinoxylan ratios	0.24	0.34	0.45	0.26	0.36	0.46
Viscosity, cP	1.04	1.05	1.05	1.21	1.21	1.21
Calcium	0.70	0.70	0.71	0.75	0.74	0.74
Available phosphorus	0.22	0.24	0.25	0.27	0.25	0.23
Lysine (SID)	0.97	0.96	0.96	0.97	0.97	0.98
Methionine (SID)	0.30	0.30	0.30	0.30	0.30	0.30
Cysteine (SID)	0.26	0.26	0.26	0.26	0.26	0.26
Threonine (SID)	0.60	0.60	0.59	0.60	0.60	0.60
Tryptophan (SID)	0.17	0.17	0.16	0.17	0.17	0.17
Isoleucine (SID)	0.50	0.50	0.49	0.50	0.50	0.51
Leucine (SID)	1.21	1.22	1.23	1.22	1.22	1.22
Valine (SID)	0.65	0.65	0.64	0.65	0.65	0.65
Arginine (SID)	0.52	0.54	0.55	0.52	0.60	0.67
Histidine (SID)	0.33	0.33	0.33	0.33	0.33	0.33
Phenylalanine (SID)	0.60	0.61	0.62	0.61	0.61	0.60
Tyrosine (SID)	0.36	0.37	0.37	0.41	0.39	0.37
Methionine + Cysteine (SID)	0.56	0.56	0.56	0.56	0.56	0.56
Phenylalanine + Tyrosine (SID)	0.96	0.98	0.99	1.02	0.99	0.97

^a^*L*_*V*_*L*_*β/AX*_ Diet with low viscosity and low β-glucan-to-arabinoxylan ratios, *L*_*V*_*M*_*β/AX*_ Diet with low viscosity and middle β-glucan-to-arabinoxylan ratios, *L*_*V*_*H*_*β/AX*_ Diet with low viscosity and high β-glucan-to-arabinoxylan ratios, *H*_*V*_*L*_*β/AX*_ Diet with high viscosity and low β-glucan-to-arabinoxylan ratios, *H*_*V*_*M*_*β/AX*_ Diet with high viscosity and middle β-glucan-to-arabinoxylan ratios, *H*_*V*_*H*_*β/AX*_ Diet with high viscosity and high β-glucan-to-arabinoxylan ratios, *Corn DDGS* Corn distillers dried grains with solubles

^b^Supplied the following per kilogram complete diet, vitamin A, 28,500 IU; vitamin D, 36,000 IU; vitamin E, 67.5 IU; vitamin K, 37.5 mg; vitamin B_1_, 17.5 mg; vitamin B_2_, 215 mg; vitamin B_6_, 69 mg; vitamin B_12_, 0.075 mg, nicotinic acid, 70 mg, folic acid, 3 mg, calcium pantothenate, 0.375 mg, antioxidant, 0.15 mg, choline chloride, 105 mg; Co as CoSO₄, 1 mg; Cu as CuSO_4_·5H_2_O, 155 mg; Fe as FeSO_4_·H_2_O, 145 mg; Mn as MnO, 75 mg; Zn as ZnSO_4_, 125 mg; I as KI, 0.3 mg; Se as Na_2_SeO_3_, 0.3 mg

^c^Data are calculated according to NRC (2012) standards [20]

^d^*SID* Standardized ileal digestible values

#### Energy metabolism experiment and feeding management

A closed reflux respiratory calorimeter at the Jilin Academy of Agricultural Sciences was employed for this study. The experiment lasted 35 d, and each group of experimental pigs was raised in a single pen for the first 14 d to adapt to the diet and environmental conditions. Starting on the d 15, each group of experimental pigs was transferred to a respiratory calorimeter in three batches (12 pigs per batch, 2 pigs per group, one batch lasting for 7 d) to measure the daily O_2_ consumption and CO_2_ and CH_4_ excretion. During this period, the experimental pigs were fed the experimental diets at a daily feeding level of three times their metabolizable energy (ME) requirement for maintenance (419 kJ/kg body weight^0.75^) [21, 22], and they were weighed every 7 d to correct the feeding amount. After each batch of pigs was transferred to the respiratory calorimeter chamber, total feces and urine were collected from d 2 to 6 of each batch, and the gas exchange volume during the same period was recorded to calculate total heat production (THP). Fasting metabolism was assessed by quantifying fasting metabolic heat production (FHP), as previously described [23].

Throughout the experiment, all pigs were allowed free access to water, and their daily diet was divided into two equal portions fed at 08:00 and 16:00. Feces and urine were collected every morning, weighed, and stored at −20 °C. The subsequent calculations and analysis did not include the gas exchange volume during this period. After the experiment, feces and urine collected from each pig were mixed, and a portion of the sample was collected for nutrient determination.

#### Slaughter procedure and sample collection

The slaughter procedure was carried out according to the method reported by Li et al. [24]. On the final day of the experiment, all pigs, after being fed for 2 h in the morning, were euthanized, and blood from the hepatic portal vein and digesta from the ileal were separately collected and stored at −20 °C to measure the concentration of SCFA and the apparent ileal digestibility (AID) of nutrients. Sufficient ileal digesta were collected for subsequent in vitro fermentation experiments during this period.

### Exp. 3

#### In vitro fermentation of ileal digesta

The ileal digesta (dried for 72 h at 65 °C, *n* = 6) of each group of pigs was used for validation experiments on the in vitro fermentation characteristics of the diet. The in vitro fermentation method described in Exp. 1 was used to conduct the in vitro fermentation experiment of ileal digesta, and fermentation broth samples were collected at the end of fermentation and stored at −20 °C to measure the SCFA production.

### Chemical compositions and physical properties analyses

Concentrations of DM (method 930.15) and crude protein (CP; method 954.01) in feedstuffs, urine, and fecal samples were measured according to the procedures of the AOAC (2000) [25]. The concentrations of crude fiber (CF; method 978.10), ether extract (EE; method 920.39), ash (method 924.05), total calcium (method 984.01), and total phosphorus (method 965.17) in the feedstuffs samples were measured according to the procedures of the AOAC (2006) [26]. Acid detergent fiber (ADF), neutral detergent fiber (NDF), and acid detergent lignin (ADL) in the feedstuffs were determined using a fiber analyzer, according to a previous report [27]. The TDF, SDF, and IDF were determined using the enzyme gravimetric method (method 985.29, AOAC, 2007) [28]. The total β-glucan and arabinoxylan contents of the feedstuffs were determined using a Megazyme assay kit (Megazyme International Ireland Ltd., Co. Wicklow, Ireland). The estimated lignin content was presented as ADL. The content of GE was determined using an oxygen bomb calorimeter (Parr Bomb Calorimeter 6200, Parr Instrument Company, Moline, IL, USA). The diet and fecal samples were analyzed to establish the TiO_2_ content using a UV spectrophotometer, as reported by Biasato et al. [29].

The monosaccharides composition was hydrolyzed by the method reported by Englyst et al. [30], and determined by high-performance liquid chromatography (STD standard Testing Co., Ltd., Qingdao, China) [31]. Briefly, the hydrolysate was extracted repeatedly (three times) with chloroform after pre-column derivatization using 1-phenyl-3-methyl-5-pyrazolone (PMP), and then determined on the machine after 0.45 μm filter membrane. The analysis of PMP-labeled monosaccharides was carried out on an Agilent 1200 high-performance liquid chromatography system equipped with a quaternary gradient pump unit, an ultraviolet detector, an auto-sampler, and the column oven controlled by an Agilent chromatography workstation. The analytical column was an RP-C_18_ column (Agilent; 4.6 mm × 250 mm × 5 μm). The mobile phase comprises 0.1 mol/L KH_2_PO_4_ solution (A) and acetonitrile (B). The elution conditions were as follows: equal elution (A:B = 82:18), flow rate: 1.0 mL/min, column temperature: 25 °C, sample size: 10 µL, detection wavelength: 254 nm.

The water holding capacity (WHC) was determined as previously described [32]. Briefly, dry feedstuffs (0.3 g) were weighed and left to stand in distilled water (10 mL) for 1 h at 25 °C, followed by centrifugation at 14,000 × *g* for 20 min, allowing the residues to stand for 30 min, drying overnight at 110 °C, and weight measurement. To determine the swelling capacity (SC), dry feedstuffs (0.2 g) were hydrated in 10 mL of distilled water in a graduated test tube at room temperature for 18 h [33]. The SC was calculated using the equation *SC* (mL/g) = (*V*_1_ − *V*_0_)/*W*_0_, where *V*_1_ is the volume of the hydrated feedstuffs; *V*_0_ is the volume of feedstuffs prior to hydration, and *W*_0_ is the weight of feedstuffs prior to hydration. Bulk weight (BW) was determined based on previous reports [34]. Briefly, the feedstuffs were placed in a 250-mL beaker, and the sample’s top was flattened (not compacted). The BW was calculated using the equation *BW* (g/L) = (*W*_1_ − *W*_2_)/250 mL, where *W*_1_ is the weight of the beaker after loading the sample, and *W*_2_ is the weight of the beaker. Viscosity was measured using a procedure modified by Serena and Knudsen [35] and expressed in centipoises. Briefly, feedstuffs (2 g) were dissolved in 10 mL of 0.9% NaCl solution and extracted in a water bath at 40 °C for 1 h, followed by centrifugation at 3,500 × *g* for 25 min at 23 °C. The supernatant (0.5 mL) was removed by suction. The shear viscosity of each suspension was measured using an Anton Paar MCR 102 rotational rheometer (Anton Paar GmbH, Graz, Austria) with the concentric cylinder geometry (28.907 mm measuring cup diameter, 26.663 mm bob diameter, and 40 mm gap length). Measurements were performed at 39 °C, with declining shear rates from 50/s to 1/s in 25 steps after a 30 s pre-shear at 10/s.

### Short-chain fatty acid concentration analyses

SCFA concentrations were measured as described by Tiwari et al. [16]. Briefly, 1 mL of supernatant from each time point of fermentation (20,000 × *g* centrifuged for 15 min at 4 °C) was added to the metaphosphoric acid solution (0.2 mL, 25%) and mixed thoroughly by vortex shaking. The 70 µL mixture was analyzed for SCFA using a GC-System (Agilent 7890A-G3440A-GC System; Agilent Technologies, Santa Clara, USA). The SCFA concentrations in the hepatic portal vein plasma measurement procedures were the same.

SCFA concentrations in the digesta and feces were measured following a previously described protocol [36]. Briefly, samples were thawed on ice, and approximately 0.5 g of the sample was added to 8 mL of deionized water. The mixture was then thoroughly homogenized by vortexing for 1 min and centrifuged at 13,000 × *g* for 5 min. The remaining procedures were the same as those described above.

### DNA extraction, 16S sequence processing and analysis

Microbial DNA was extracted from the fermentation samples using the E.Z.N.A.^®^ soil DNA Kit (Omega Biotechnology, Norcross, GA, USA) following the manufacturer’s instructions. DNA quality and concentration were assessed using gel electrophoresis and a NanoDrop^®^ ND-2000 spectrophotometer (Thermo Scientific Inc., USA) and stored at −80 °C. The V3–V4 hypervariable regions of the bacterial 16S rRNA gene were amplified using primer pairs 338F and 806R on an ABI GeneAmp^®^ 9700 PCR thermocycler (ABI, CA, USA). Amplification consisted of 27 cycles of denaturation, annealing, extension, and final step at 4 °C. As previously reported, the PCR products were purified, quantified, pooled, and sequenced on the Illumina MiSeq PE300 platform. Microbial data were analyzed using the Majorbio Cloud platform. All amplicon sequence variants (ASV) were classified against the Silva 132 database using a naïve Bayes classifier constructed using the scikit-learn software. α- and β-diversity were calculated using the vegan package (version 2.5–6) in the R studio package. Principal co-ordinates analysis (PCoA) was performed using weighted Bray-Curtis and UniFrac distance metrics. Permutational multivariate analysis of variance (PERMANOVA) was used to evaluate factors shaping the microbiota using the adonis function of the “vegan” package (999 permutations). Linear discriminant analysis effect size (LEFSe) analysis was used to identify microbial differences between groups, and significant differences were indicated when the linear discriminant analysis score was greater than or equal to 3.5.

### Calculations

Organic matter (OM), TDF, nitrogen-free extract (NFE), cellulose, non-starch polysaccharides (NSP), apparent total tract digestibility (ATTD), AID, and hindgut digestibility (HD) were calculated according to Zhao et al. [12] as follows:

1 $$\mathrm{OM}\;\left(\%\right)=\mathrm{DM}\;\left(\%\right)-\mathrm{Ash}\;\left(\%\right)$$

2 $$\mathrm{TDF}\;\left(\%\right)=\mathrm{SDF}\;\left(\%\right)+\mathrm{IDF}\;\left(\%\right)$$

3 $$\mathrm{NFE}\;\left(\%\right)=\mathrm{DM}\;\left(\%\right)-\mathrm{CP}\;\left(\%\right)-\mathrm{EE}\;\left(\%\right)-\mathrm{CF}\;\left(\%\right)-\mathrm{Ash}\;\left(\%\right)$$

4 $$\mathrm{Cellulose}\;\left(\%\right)=\mathrm{ADF}\;\left(\%\right)-\mathrm{ADL}\;\left(\%\right)$$

5 $$\mathrm{NSP}\;\left(\%\right)=\mathrm{TDF}\;\left(\%\right)-\mathrm{ADL}\;\left(\%\right)$$

6 $$\mathrm{ATTD}\;(\%)=100\times\left[\mathrm{nutrient}\;\mathrm{intake}\;(\mathrm g/\mathrm d)-\mathrm{nutrient}\;\mathrm{excretion}\;(\mathrm g/\mathrm d)\right]/\mathrm{nutrient}\;\mathrm{intake}\;(\mathrm g/\mathrm d)$$

7 $$\mathrm{AID}\;(\%)=1-\left[\mathrm{nutrient}\;\mathrm{content}\;\mathrm{in}\;\mathrm{ileal}\;\mathrm{digesta}\;(\%)/\mathrm{nutrient}\;\mathrm{content}\;\mathrm{in}\;\mathrm{diet}\;(\%)\right]\times\left[\mathrm{Ti}\;\mathrm{content}\;\mathrm{in}\;\mathrm{diet}\;(\%)/\mathrm{Ti}\;\mathrm{content}\;\mathrm{in}\;\mathrm{ileal}\;\mathrm{digesta}\;(\%)\right]\times100$$

8 $$\mathrm{HD}\;(\%)=\mathrm{ATTD}\;(\%)-\mathrm{AID}\;(\%)$$

Digestible energy (DE), ME, THP, heat increment (HI), RE, NE, CH_4_ energy, and respiratory quotient (RQ) were calculated according to previous reports [23, 37]:

9 $$\mathrm{DE}\;\left(\mathrm{MJ}/\mathrm d\right)=\mathrm{GE}\;\mathrm{intake}\;\left(\mathrm{MJ}/\mathrm d\right)-\mathrm{fecal}\;\mathrm{energy}\;\left(\mathrm{FE},\;\mathrm{MJ}/\mathrm d\right)$$

10 $$\mathrm{ME}\;\left(\mathrm{MJ}/\mathrm d\right)=\mathrm{DE}\;\left(\mathrm{MJ}/\mathrm d\right)-\mathrm{urinary}\;\mathrm{energy}\;\left(\mathrm{UE},\;\mathrm{MJ}/\mathrm d\right)-{\mathrm{CH}}_4\;\mathrm{energy}\;\left(\mathrm{MJ}/\mathrm d\right)$$

11 $$\mathrm{THP}\;(\mathrm{kJ})=16.181\;{\mathrm O}_2\;\mathrm{consumption}\;(\mathrm L)+5.023\;{\mathrm{CO}}_2\;\mathrm{excretion}\;(\mathrm L)-2.168\;{\mathrm{CH}}_4\;\mathrm{excretion}\;(\mathrm L)-5.989\;\mathrm{urinary}\;\mathrm{nitrogen}\;(\mathrm{UN},\;\mathrm g)$$

12 $$\mathrm{HI}\;(\mathrm{MJ}/\mathrm d)=\mathrm{THP}\;(\mathrm{MJ}/\mathrm d)-\mathrm{FHP}\;(\mathrm{MJ}/\mathrm d)$$

13 $$\mathrm{RE}\;(\mathrm{MJ}/\mathrm d)=\mathrm{ME}\;(\mathrm{MJ}/\mathrm d)-\mathrm{THP}\;(\mathrm{MJ}/\mathrm d)$$

14 $$\mathrm{NE}\;(\mathrm{MJ}/\mathrm d)=\mathrm{RE}\;(\mathrm{MJ}/\mathrm d)+\mathrm{FHP}\;(\mathrm{MJ}/\mathrm d)$$

15 $$\mathrm{RQ}={\mathrm{CO}}_2\;\mathrm{excretion}\;(\mathrm L)/{\mathrm O}_2\;\mathrm{consumption}\;(\mathrm L)$$

16 $${\mathrm{CH}}_4\;\mathrm{energy}\;(\mathrm{kJ}/\mathrm d)={\mathrm{CH}}_4\;(\mathrm L/\mathrm d)\times39.6\;(\mathrm{kJ}/\mathrm L)$$

Protein oxidation (OXP) and carbohydrate oxidation (OXCHO) were calculated according to previous reports [38]:

17 $$\mathrm{OXP}\;(\mathrm{kJ})=\mathrm{UN}\;(\mathrm g)\times6.25\times18.42\;(\mathrm{kJ}/\mathrm g)$$

18 $$\mathrm{OXCHO}\;(\mathrm{kJ})=\left[-2.968\;{\mathrm O}_2\;\mathrm{consumption}\;(\mathrm L)+4.174\;{\mathrm{CO}}_2\;\mathrm{excretion}\;(\mathrm L)-1.761\;{\mathrm{CH}}_4\;\mathrm{excretion}\;(\mathrm L/\mathrm d)-2.466\;\mathrm{UN}\;(\mathrm g)\right]\times17.58\;(\mathrm{kJ}/\mathrm g)$$

### Statistical analysis

Data were analyzed using the *Y*_*ijk*_ = *µ* + *α*_*i*_ + *β*_*j*_ + *α*_*i*_ × *β*_*j*_ + *ɛ*_*ijk*_ model, where *µ* is the population mean, *α*_*i*_ is the effect of dietary apparent viscosity (*i* = 1, 2), *β*_*j*_ is the β-glucan-to-arabinoxylan ratios (*j* = 1, 2, 3), *α*_*i*_ × *β*_*j*_ is the linear interaction between dietary apparent viscosity and the β-glucan-to-arabinoxylan ratios, and *ɛ*_*ijk*_ is the residual effect. The data were analyzed using SPSS version 27.0 (IBM Corp., Armonk, NY, USA). All data were checked for normal distribution and homogeneity of variance using Levene’s test. The indicators related to in vitro digestion and fermentation of feedstuffs were analyzed using one-way ANOVA. The apparent viscosity, β-glucan-to-arabinoxylan ratios, and linear interaction effects were analyzed using two-way ANOVA. Differences between the 6 treatments were analyzed by multiple comparisons using Tukey’s method. Regression curve estimation was used to analyze the linear and quadratic relationships between dietary fiber structure (β-glucan-to-arabinoxylan ratios) and dependent variables. Pearson correlation analysis was used to study the relationship between the physicochemical properties of dietary fiber and feedstuffs in vitro fermentation characteristics. Multiple regression analysis was used to establish predictive equations for RE and NE. Differences at a *P* < 0.01 were considered highly significant, at a *P* < 0.05 were considered significant, and at a 0.05 ≤ *P* < 0.1 were considered a trend. The results were expressed as the mean and standard error of the mean.

## Results

### Changes of short-chain fatty acids and gas production of feedstuffs during in vitro fermentation

**Fig. 1:**
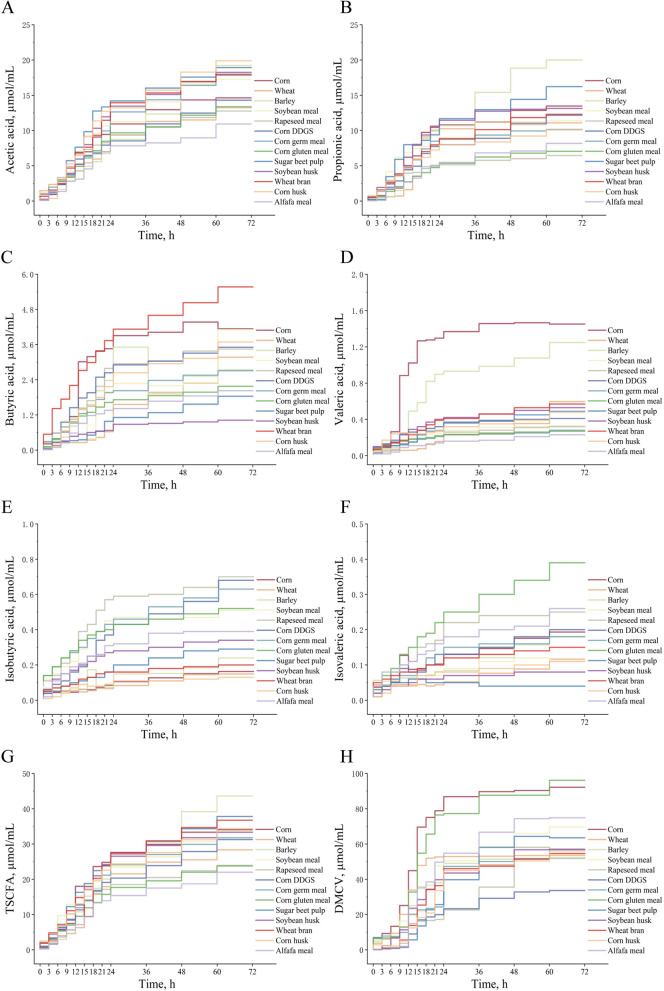
Dynamic changes of acetic acid (**A**), propionic acid (**B**), butyric acid (**C**), valeric acid (**D**), isobutyric acid (**E**), isovaleric acid (**F**), total short-chain fatty acids (**G**), dry matter correction gas production (**H**) of different feedstuffs during in vitro fermentation. *Corn DDGS* Corn distillers dried grains with solubles, *TSCFA* Total short-chain fatty acids, *DMCV* Dry matter correction gas production

Overall, there were differences in the concentrations of SCFA, and DM corrected gas production (DMCV) of the different feedstuffs during in vitro fermentation (Fig. 1 and Fig. S1 and S2). When fermented for 3 h, the total SCFA (TSCFA) concentrations of wheat, barley, wheat bran, and corn were higher than those of other feedstuffs (*P* < 0.01), specifically observed in the generation of acetic acid and propionic acid. The TSCFA content of wheat bran and corn germ meal increased rapidly at 6 h, while that of soybean meal increased at 9–12 h. During the 15–18 h period, the TSCFA of corn, sugar beet pulp, and soybean meal were higher than those of other feedstuffs (*P* < 0.01). At 21–24 h, the TSCFA of corn and sugar beet pulp was still the highest. At 36 h, the TSCFA of sugar beet pulp, wheat bran, corn, and soybean husk were higher than those of the other feedstuffs (*P* < 0.01). At 60–72 h, the TSCFA of barley was higher than that of other feedstuffs (*P* < 0.01). At the end of fermentation, the TSCFA production of barley was the highest, followed by sugar beet pulp, wheat bran, corn, corn husk, soybean husk, soybean meal, corn germ meal, corn DDGS, wheat, rapeseed meal, corn gluten meal, and alfalfa meal.

In addition, the DMCV results showed that corn exhibited the highest DMCV during fermentation for 0–60 h (*P* < 0.01). During 3 and 6 h of fermentation, the DMCV of corn, corn germ meal, corn gluten meal, barley, and wheat were higher than those of other feedstuffs (*P* < 0.01). After 9 h of in vitro fermentation, the DMCV of wheat, soybean meal, and soybean husks increased rapidly. The DMCV of wheat and soybean husks reached stability at the 18^th^ and 36^th^ hours of fermentation, respectively, whereas the DMCV of soybean meal continued to increase after 9 h until 72 h. The DMCV of corn gluten meal after in vitro fermentation for 18 h was higher than that of other feedstuffs, except corn (*P* < 0.01). Except for corn, the DMCV of corn gluten meal was higher than that of 11 other feedstuffs, such as wheat, barley, and alfalfa meal, during the fermentation process from 18 to 72 h (*P* < 0.01). After 15 h of in vitro fermentation, alfalfa meal fermentation began to increase, and DMCV gradually increased. At 60 h, the DMCV of alfalfa meal was higher than that of the 10 feedstuffs, except for corn and corn gluten meal (*P* < 0.01). Corn DDGS had the lowest DMCV during the entire fermentation process, which was lower than that of the other 12 feedstuffs after 48, 60, and 72 h of in vitro fermentation (*P* < 0.01). At the end of fermentation, corn gluten meal exhibited the highest DMCV production, followed by corn, alfalfa meal, soybean meal, sugar beet pulp, soybean husk, corn husk, corn germ meal, wheat, wheat bran, barley, rapeseed meal, and corn DDGS.

### Relationship between physicochemical properties of dietary fiber and feedstuffs in vitro fermentation characteristics

**Fig. 2:**
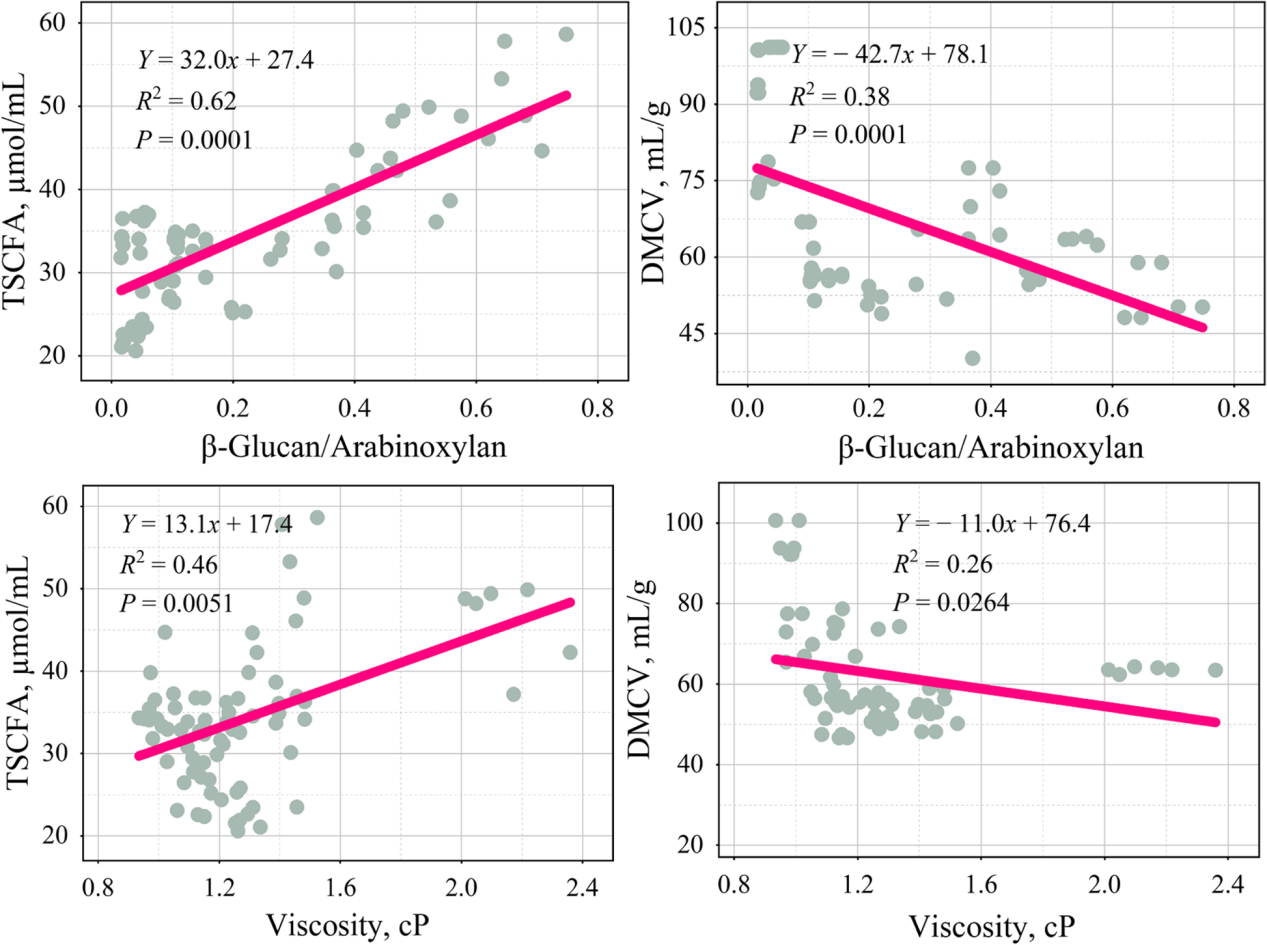
Linear regression analysis of the relationship between feedstuffs in vitro fermentation characteristics and physicochemical properties of dietary fiber

The results showed that acetic acid and the β-glucan-to-arabinoxylan ratios, β-glucan, fructan, arabinose, SDF, viscosity, SC, and WHC showed a positive correlation (*R* ≥ 0.04, *P* < 0.05; Table 3) and a negative correlation with NSP and uronic acid (*R* ≤ −0.11, *P* < 0.05). Propionic acid and β-glucan, β-glucan-to-arabinoxylan ratios, and rhamnose were positively correlated (*R* ≥ 0.18, *P* < 0.05). In contrast, it negatively correlated with arabinose, uronic acid, galactose, cellulose, and IDF (*R* ≤ −0.10, *P* < 0.05). Butyric acid and rhamnose, mannose, BW, SC, and WHC showed a positive correlation (*R* ≥ 0.17, *P* < 0.05), while negatively correlated with galactose, glucose, uronic acid, the β-glucan-to-arabinoxylan ratios, IDF, SDF, lignin, and cellulose (*R* ≤ −0.05, *P* < 0.05). Valeric acid and mannose, glucose, and rhamnose were positively correlated (*R* ≥ 0.17, *P* < 0.01), but there was a negative correlation with IDF, SDF, NSP, cellulose, and WHC (*R* ≤ −0.24, *P* < 0.05). Isobutyric acid and IDF, mannose, and fructan showed a positive correlation (*R* ≥ 0.06, *P* < 0.05), whereas there was a negative correlation with glucose, rhamnose, and viscosity (*R* ≤ −0.12, *P* < 0.01). Isovaleric acid and arabinose, galactose, and uronic acid were positively correlated (*R* ≥ 0.04, *P* < 0.01), whereas there was a negative correlation with the SDF, fructan, β-glucan-to-arabinoxylan ratios, arabinoxylan and SC (*R* ≤ −0.05, *P* < 0.05). The TSCFA and β-glucan-to-arabinoxylan ratios, β-glucan, galactose, and viscosity showed a positive correlation (*R* ≥ 0.25, *P* < 0.05) but negatively correlated with IDF, lignin, mannose, and rhamnose (*R* ≤ −0.29, *P* < 0.05). The DMCV was positively correlated with cellulose and mannose (*R* ≥ 0.28, *P* < 0.05) and was negatively correlated with the β-glucan-to-arabinoxylan ratios, β-glucan, arabinoxylan, glucose, galactose, lignin, viscosity, and SC (*R* ≤ −0.20, *P* < 0.05). Further linear regression analysis showed a linear relationship between the β-glucan-to-arabinoxylan ratios and apparent viscosity with TSCFA and DMCV (Fig. 2). The TSCFA was positively correlated with the β-glucan-to-arabinoxylan ratios (*R*^2^ = 0.62, *P* < 0.001) and apparent viscosity (*R*^2^ = 0.46, *P* < 0.01). The DMCV was negatively correlated with the β-glucan-to-arabinoxylan ratios (*R*^2^ = 0.38, *P* < 0.001) and apparent viscosity (*R*^2^ = 0.26, *P* < 0.05).

**Table 3:** Correlation analysis between physicochemical properties of dietary fiber and feedstuffs in vitro fermentation characteristics

Item	Acetic acid	Propionic acid	Butyric acid	Valeric acid	Isobutyric acid	Isovaleric acid	TSCFA	DMCV
IDF	0.26	−0.28^*^	−0.29^*^	−0.56^*^	0.24^*^	0.02	−0.29^*^	0.05
SDF	0.23^*^	0.19	−0.48^**^	−0.24^*^	0.01	−0.25^*^	−0.03	0.05
Lignin	0.21	0.38	−0.41^**^	0.01	−0.05	−0.22	−0.39^**^	−0.53^**^
NSP	−0.40^**^	−0.4	0.07	−0.46^**^	0.22	0.26	−0.18	0.13
Cellulose	0.34	−0.10^**^	−0.43^**^	−0.48^**^	0.09	−0.11	−0.23	0.30^*^
Arabinose	0.04^*^	−0.32^**^	−0.46	−0.37	0.35	0.34^**^	0.08	−0.02
Xylose	0.33	0.41	−0.02	0.02	−0.19	−0.37	−0.02	−0.08
Mannose	−0.05	−0.09	0.17^**^	0.21^**^	0.11^**^	0.04	−0.38^**^	0.28^*^
Galactose	−0.02	−0.12^*^	−0.49^**^	−0.35	0.37	0.13^**^	0.25^*^	−0.67^***^
Glucose	0.07	0.23	−0.33^**^	0.17^**^	−0.12^**^	−0.34	0.15	−0.35^*^
Rhamnose	−0.06	0.18^*^	0.69^*^	0.42^**^	−0.37^**^	−0.09	−0.41^**^	0.09
Uronic acid	−0.11^*^	−0.25^*^	−0.29^*^	−0.32	0.39	0.04^**^	−0.04	−0.09
Fructan	0.26^**^	0.28	−0.21	−0.17	0.06^*^	−0.35^*^	−0.16	0.06
β-Glucan	0.38^***^	0.58^***^	0.04	−0.21	−0.36	−0.23	0.65^***^	−0.61^***^
Arabinoxylan	0.38	0.52	0.07	0.16	0.1	−0.05^**^	0.04	−0.20^*^
β-Glucan-to-arabinoxylan ratios	0.58^***^	0.50^***^	−0.05^*^	−0.1	−0.11	−0.37^**^	0.66^***^	−0.67^***^
Viscosity	0.42^***^	0.01	0.21	−0.04	−0.39^***^	−0.22	0.43^**^	−0.27^*^
WHC	0.25^*^	−0.12	0.49^***^	−0.26^*^	−0.07	−0.04	0.04	−0.13
SC	0.40^***^	0.05	0.50^***^	−0.18	−0.09	−0.29^*^	0.17	−0.26^**^
BW	0.05	0.05	0.65^***^	0.17	−0.14	−0.16	0.15	−0.21

*IDF* Insoluble dietary fiber, *SDF* Soluble dietary fiber, *NSP* Non-starch polysaccharides, *TSCFA* Total short-chain fatty acids, *DMCV* Dry matter corrected gas production, *WHC* Water holding capacity, *SC* Swelling capacity, *BW* Bulk weight

The correlation analysis results are represented by Pearson correlation coefficient

^*^P < 0.05, ^**^P < 0.01, ^***^P < 0.001

### Effects of dietary fiber structure and apparent viscosity on nutrients digestibility

The effects of dietary fiber structure and apparent viscosity on nutrient digestibility of growing pigs are shown in Table 4. The AID and ATTD of DM and OM and the HD of ash in the H_V_ group were lower than those in the L_V_ group, while the AID, ATTD, and HD of TDF, SDF, and IDF were higher than those in the L_V_ group (*P* < 0.05). The AID of DM and OM in the H_β/AX_ and M_β/AX_ groups were lower than those in the L_β/AX_ group, and the AID of TDF was higher than that in the L_β/AX_ group (*P* < 0.05). The AID of SDF and IDF in the H_β/AX_ group was higher than in the L_β/AX_ group (*P* < 0.05). The ATTD of the DM and OM in the L_β/AX_ group was higher than that in the H_β/AX_ group, whereas the ATTD of the TDF, SDF, and IDF was lower than that in the H_β/AX_ group (*P* < 0.05). In addition, the HD of TDF, SDF, IDF, and ash in the H_β/AX_ group was higher than those in the L_β/AX_ and M_β/AX_ groups, and the HD of DM was higher than that in the L_β/AX_ group (*P* < 0.05). When all 6 dietary treatments were compared, there were differences in the AID of DM, OM, TDF, and SDF, ATTD of OM, TDF, SDF, and IDF, and the HD of TDF, SDF, and IDF (*P* < 0.05). Dietary β-glucan-to-arabinoxylan ratios and apparent viscosity had an interaction effect on ATTD and HD of TDF, SDF, IDF, and HD of DM (*P* < 0.05). Linear comparison analysis showed that the dietary β-glucan-to-arabinoxylan ratios had linear effects on the AID and ATTD of DM, OM, TDF, SDF, and IDF, and the HD of TDF, SDF, and IDF (*P* < 0.01). At the same time, the dietary β-glucan-to-arabinoxylan ratios had quadratic effect on ATTD of OM (*P* < 0.05).

**Table 4:** Effects of dietary fiber structure and apparent viscosity on nutrients digestibility of growing pigs

Item	Viscosity		β-Glucan-to-arabinoxylan ratios			Treatments						SEM	*P*-value				
	L_V_	H_V_	L_β/AX_	M_β/AX_	H_β/AX_	L_V_L_β/AX_	L_V_M_β/AX_	L_V_H_β/AX_	H_V_L_β/AX_	H_V_M_β/AX_	H_V_H_β/AX_		V	β/AX	V × β/AX	Linear	Quadratic
AID, %																	
DM	67.49^m^	62.42^n^	67.71^x^	64.42^y^	62.73^y^	69.79^a^	66.41^ab^	66.28^ab^	65.63^ab^	62.44^bc^	59.18^c^	0.51	< 0.001	< 0.001	0.384	< 0.001	0.317
OM	68.30^m^	61.12^n^	68.33^x^	64.25^y^	61.55^y^	70.82^a^	67.69^ab^	66.39^ab^	65.84^ab^	60.81^bc^	56.72^c^	0.54	< 0.001	< 0.001	0.230	< 0.001	0.120
Ash	20.78	20.44	21.55	20.94	19.34	21.95	21.15	19.24	21.16	20.74	19.43	0.49	0.741	0.191	0.921	0.256	0.761
CF	7.01	7.06	6.70	6.98	7.42	6.85	6.89	7.29	6.56	7.07	7.54	0.16	0.888	0.230	0.772	0.346	0.492
TDF	9.99^n^	12.67^m^	10.31^y^	11.82^x^	11.85^x^	8.98^b^	10.40^ab^	10.58^ab^	11.65^a^	13.25^a^	13.11^a^	0.23	< 0.001	0.016	0.963	< 0.001	0.688
SDF	19.82^n^	22.71^m^	20.06^y^	21.12^xy^	22.61^x^	18.26^d^	20.15^c^	21.05^bc^	21.86^bc^	22.09^b^	24.17^a^	0.28	< 0.001	0.004	0.482	< 0.001	0.794
IDF	3.36^n^	3.67^m^	3.34^y^	3.51^xy^	3.69^x^	3.16	3.36	3.55	3.51	3.67	3.84	0.06	0.023	0.105	0.986	0.004	0.872
ATTD, %																	
DM	77.85^m^	72.74^n^	77.48^x^	74.57^xy^	73.83^y^	78.64	77.40	77.51	76.33	71.73	70.15	0.62	< 0.001	0.054	0.255	< 0.001	0.151
OM	79.63^m^	72.88^n^	78.65^x^	76.34^xy^	73.77^y^	80.70^a^	79.24^ab^	78.95^ab^	76.60^ab^	73.45^bc^	68.59^c^	0.54	< 0.001	0.004	0.065	< 0.001	0.026
Ash	39.31	37.61	36.62	37.84	40.93	39.31	36.78	41.85	33.93	38.90	40.01	1.06	0.431	0.251	0.369	0.930	0.462
CF	27.18	27.57	26.07	27.30	28.75	25.78	27.27	28.49	26.37	27.34	29.01	0.66	0.772	0.276	0.985	0.307	0.996
TDF	37.36^n^	44.07^m^	38.43^y^	40.30^xy^	43.40^x^	34.30^d^	36.76^c^	41.01^bc^	42.56^b^	43.85^ab^	45.78^a^	0.81	0.002	< 0.001	0.040	< 0.001	0.514
SDF	66.31^n^	76.24^m^	67.61^y^	71.46^xy^	74.76^x^	61.34^d^	66.67^c^	70.92^bc^	73.88^ab^	76.24^a^	78.60^a^	0.97	0.002	< 0.001	< 0.001	< 0.001	0.514
IDF	22.93^n^	26.61^m^	23.26^y^	24.35^y^	26.70^x^	21.68^c^	22.41^bc^	24.71^bc^	24.84^bc^	26.29^ab^	28.69^a^	0.35	< 0.001	0.002	< 0.001	< 0.001	0.537
HD, %																	
DM	10.36	10.32	9.77^y^	10.15^xy^	11.10^x^	8.85	11.00	11.22	10.70	9.29	10.98	0.24	0.943	< 0.001	0.020	0.326	0.118
OM	11.33	11.76	10.32	12.10	12.22	9.88	11.56	12.56	10.77	12.64	11.87	0.78	0.786	0.547	0.880	0.481	0.621
Ash	18.53^m^	17.17^n^	15.07^y^	16.89^y^	21.59^x^	17.36	15.64	22.61	12.78	18.15	20.58	0.66	0.316	0.001	0.107	0.323	0.347
CF	20.17	20.51	19.37	20.32	21.33	18.93	20.38	21.20	19.81	20.27	21.47	0.52	0.746	0.325	0.928	0.317	0.821
TDF	27.37^n^	31.40^m^	28.12^y^	28.48^y^	31.55^x^	25.33^d^	26.36^c^	30.43^bc^	30.61^b^	30.91^b^	32.69^a^	0.77	0.015	< 0.001	0.020	< 0.001	0.487
SDF	46.49^n^	53.53^m^	47.55^z^	50.34^y^	52.15^x^	43.08^e^	46.52^d^	49.87^c^	52.02^b^	54.15^a^	54.43^a^	0.83	0.019	< 0.001	< 0.01	0.009	0.467
IDF	19.58^n^	22.94^m^	19.92^y^	20.84^y^	23.01^x^	18.52^d^	19.05^d^	21.16^c^	21.33^bc^	22.62^b^	25.85^a^	0.29	< 0.001	0.001	0.040	< 0.001	0.442

*L*_*V*_*L*_*β/AX*_ Diet with low viscosity and low β-glucan-to-arabinoxylan ratios, *L*_*V*_*M*_*β/AX*_ Diet with low viscosity and middle β-glucan-to-arabinoxylan ratios, *L*_*V*_*H*_*β/AX*_ Diet with low viscosity and high β-glucan-to-arabinoxylan ratios, *H*_*V*_*L*_*β/AX*_ Diet with high viscosity and low β-glucan-to-arabinoxylan ratios, *H*_*V*_*M*_*β/AX*_ Diet with high viscosity and middle β-glucan-to-arabinoxylan ratios, *H*_*V*_*H*_*β/AX*_ Diet with high viscosity and high β-glucan-to-arabinoxylan ratios, *SEM* Standard error of the mean, *V* Apparent viscosity of diets, *β/AX* β-glucan-to-arabinoxylan ratios of diets, *V × β/AX* Linear interaction effect between apparent viscosity and β-glucan-to-arabinoxylan ratios of diets, *DM* Dry matter, *OM* Organic matter, *CF* Crude fiber, *TDF* Total dietary fiber, *SDF* Soluble dietary fiber, *IDF* Insoluble dietary fiber

^a−e^Different letters denote significant differences between experimental treatments within nutrients digestibility of each intestinal segment (*P* < 0.05)

^m,n^Different letters denote significant differences between the low and high viscosity groups within nutrients digestibility of each intestinal segment (*P* < 0.05)

^x,y,z^Different letters denote significant differences between the low, middle and high β-glucan-to-arabinoxylan ratios groups within nutrients digestibility of each intestinal segment (*P* < 0.05)

Linear contrasts analysis was used to test the linear and quadratic effects of β-glucan-to-arabinoxylan ratios on nutrients digestibility of each intestinal segment

### Effects of dietary fiber structure and apparent viscosity on energy metabolism

The effects of the dietary fiber structure and apparent viscosity on energy metabolism in growing pigs are shown in Table 5. For the reason that the pigs in each group were fed different amounts of diet with their metabolic weight to provide the same level of NE; the intakes of DE and ME were different between the H_V_ and L_V_ groups (*P* < 0.05), but the intakes of GE were not different. Compared with the L_V_ group, FE, UE, O_2_ consumption, CO_2_ excretion, CH_4_ energy, CH_4_ excretion, THP, HI, and OXP in the H_V_ group increased, while DE, ME, RE, and NE decreased (*P* < 0.05). The FE, CH_4_ energy and CH_4_ excretion of pigs in the H_β/AX_ group were higher than those in the L_β/AX_ and M_β/AX_ groups (*P* < 0.05). The O_2_ consumption, CO_2_ excretion, THP, HI, and OXP in the L_β/AX_ group were lower than those in the H_β/AX_ group (*P* < 0.05), whereas ME, RE, and NE in the L_β/AX_ group were higher than those in the M_β/AX_ and H_β/AX_ groups (*P* < 0.05). In addition, the HI of pigs in the M_β/AX_ group was higher than that of pigs in the L_β/AX_ group (*P* < 0.05). When comparing the 6 dietary treatments, there were differences in FE, O_2_ consumption, CO_2_ excretion, CH_4_ excretion, CH_4_ energy, THP, HI, ME, RE, and NE (*P* < 0.05), and there was linear interaction effect between dietary β-glucan-to-arabinoxylan ratios and apparent viscosity on FE, DE, ME, RE, and NE (*P* < 0.05). Linear comparison analysis showed that the dietary β-glucan-to-arabinoxylan ratios had linear effects on FE, UE, O_2_ consumption, CO_2_ excretion, CH_4_ excretion, CH_4_ energy, THP, HI, ME, RE, NE, and OXP (*P* < 0.01).

**Table 5:** Effects of dietary fiber structure and apparent viscosity on energy metabolism in growing pigs

Item	Viscosity		β-Glucan-to-arabinoxylan ratios			Treatments						SEM	*P*-value				
	L_V_	H_V_	L_β/AX_	M_β/AX_	H_β/AX_	L_V_L_β/AX_	L_V_M_β/AX_	L_V_H_β/AX_	H_V_L_β/AX_	H_V_M_β/AX_	H_V_H_β/AX_		V	β/AX	V × β/AX	Linear	Quadratic
GE intake, MJ/d	30.07	29.78	29.97	29.51	30.29	30.52	29.11	30.57	29.42	29.92	30.01	0.19	0.463	0.261	0.122	0.746	0.394
FE, MJ/d	5.10^n^	5.91^m^	5.23^z^	5.38^y^	5.90^x^	5.02^bc^	4.92^c^	5.35^bc^	5.43^b^	5.85^b^	6.45^a^	0.04	< 0.001	< 0.001	0.005	< 0.001	0.412
UE, MJ/d	1.38^n^	1.62^m^	1.40	1.49	1.61	1.31	1.35	1.47	1.49	1.63	1.75	0.04	0.007	0.147	0.836	0.001	0.581
DE intake, MJ/d	24.97^m^	23.87^n^	24.75	24.13	24.38	25.50^a^	24.19^ab^	25.21^b^	23.99^b^	24.07^c^	23.55^d^	0.16	0.002	0.312	0.117	0.352	0.955
ME intake, MJ/d	23.59^m^	22.25^n^	23.35	22.64	22.77	24.19^a^	22.84^ab^	23.75^a^	22.50^b^	22.44^b^	21.80^b^	0.13	< 0.001	0.092	0.049	0.303	0.915
O_2_ consumption, L/d	550.53^n^	601.53^m^	560.5^y^	572.03^xy^	595.56^x^	533.22^b^	544.87^ab^	573.51^ab^	587.79^ab^	599.18^ab^	617.60^a^	5.93	< 0.001	0.064	0.919	< 0.001	0.794
CO_2_ excretion, L/d	570.33^n^	616.04^m^	575.77^y^	591.38^xy^	612.39^x^	551.66^c^	568.32^bc^	591.00^abc^	599.88^abc^	614.45^ab^	633.78^a^	5.46	< 0.001	0.035	0.979	< 0.001	0.878
CH_4_ excretion, L/d	3.36^n^	4.21^m^	3.48^z^	3.78^y^	4.09^x^	3.08^c^	3.35^bc^	3.65^abc^	3.89^ab^	4.20^a^	4.54^a^	0.05	< 0.001	0.001	0.956	< 0.001	0.755
RQ	1.04	1.02	1.03	1.04	1.03	1.04	1.05	1.03	1.02	1.03	1.03	0.01	0.264	0.826	0.872	0.324	0.779
CH_4_ energy, MJ/d	0.13^n^	0.17^m^	0.14^z^	0.15^y^	0.16^x^	0.12^c^	0.13^bc^	0.14^ab^	0.15^ab^	0.17^a^	0.18^a^	0.01	< 0.001	0.001	0.956	< 0.001	0.755
THP, MJ/d	11.69^n^	12.74^m^	11.88^y^	12.14^xy^	12.62^x^	11.32^b^	11.59^ab^	12.17^ab^	12.44^ab^	12.69^ab^	13.08^a^	0.12	< 0.001	0.048	0.930	< 0.001	0.798
FHP, MJ/d	7.20	7.12	7.19	7.03	7.26	7.29	7.01	7.30	7.09	7.06	7.23	0.09	0.679	0.581	0.858	0.839	0.640
HI, MJ/d	4.49^n^	5.61^m^	4.69^y^	5.11^x^	5.36^x^	4.03^d^	4.58^cd^	4.87^c^	5.35^bc^	5.64^ab^	5.86^a^	0.05	< 0.001	< 0.001	0.495	< 0.001	0.227
DE, MJ/d	24.91^m^	23.87^n^	24.71	24.13	24.39	25.50	24.19	25.22	23.99	24.07	23.56	0.17	< 0.001	0.062	0.044	0.232	0.164
ME, MJ/d	23.46^m^	22.08^n^	23.21^x^	22.49^y^	22.61^y^	24.07^a^	22.71^ab^	23.60^a^	22.35^b^	22.28^b^	21.62^b^	0.13	< 0.001	0.078	0.048	< 0.001	0.910
RE, MJ/d	11.76^m^	9.35^n^	11.32^x^	10.35^y^	9.99^y^	12.74^a^	11.11^abc^	11.44^ab^	9.91^bc^	9.59^c^	8.54^d^	0.10	< 0.001	< 0.001	0.020	< 0.001	0.885
NE, MJ/d	18.96^m^	16.47^n^	18.51^x^	17.38^y^	17.25^y^	20.03^a^	18.12^ab^	18.74^a^	17.01^bc^	16.65^bc^	15.68^d^	0.11	< 0.001	< 0.001	0.011	< 0.001	0.881
OXP, MJ/d	1.37^n^	1.55^m^	1.40^y^	1.44^xy^	1.54^x^	1.34	1.33	1.43	1.46	1.54	1.64	0.03	0.002	0.100	0.734	< 0.001	0.436
OXCHO, MJ/d	12.34	12.92	12.2	12.71	12.97	11.89	12.50	12.61	12.50	12.93	13.33	0.22	0.199	0.361	0.965	0.076	0.958

*L*_*V*_*L*_*β/AX*_ Diet with low viscosity and low β-glucan-to-arabinoxylan ratios, *L*_*V*_*M*_*β/AX*_ Diet with low viscosity and middle β-glucan-to-arabinoxylan ratios, *L*_*V*_*H*_*β/AX*_ Diet with low viscosity and high β-glucan-to-arabinoxylan ratios, *H*_*V*_*L*_*β/AX*_ Diet with high viscosity and low β-glucan-to-arabinoxylan ratios, *H*_*V*_*M*_*β/AX*_ Diet with high viscosity and middle β-glucan-to-arabinoxylan ratios, *H*_*V*_*H*_*β/AX*_ Diet with high viscosity and high β-glucan-to-arabinoxylan ratios, *SEM* Standard error of the mean, *V* Apparent viscosity of diets, *β/AX* β-glucan-to-arabinoxylan ratios of diets, *V × β/AX* Linear interaction effect between apparent viscosity and β-glucan-to-arabinoxylan ratios of diets, *GE* Gross energy, *FE* Fecal energy, *UE* Urinary energy, *DE* Digestible energy, *ME* Metabolizable energy, *RQ* Respiratory quotient, *THP* Total heat production, *FHP* Fasting heat production, *HI* Heat increment, *RE* Retained energy, *NE* Net energy, *OXP* Protein oxidation, *OXCHO* Carbohydrate oxidation

^a−d^Different letters denote significant differences between experimental treatments within each energy metabolism variable (*P* < 0.05)

^m,n^Different letters denote significant differences between the low and high viscosity groups within each energy metabolism variable (*P* < 0.05)

^x,y,z^Different letters denote significant differences between the low, middle and high β-glucan-to-arabinoxylan ratios groups within each energy metabolism variable (*P* < 0.05)

Linear contrasts analysis was used to test the linear and quadratic effects of β-glucan-to-arabinoxylan ratios on each energy metabolism variable

### Effects of dietary fiber structure and apparent viscosity on short-chain fatty acids

The SCFA concentrations in the digesta and feces are shown in Table 6. The results showed that the concentrations of acetic acid, butyric acid, and TSCFA in the colon and acetic acid, propionic acid, butyric acid, isobutyric acid, and TSCFA in the cecum and feces of the H_V_ group were higher than those in the L_V_ group (*P* < 0.001). Isovaleric acid in the cecum and valeric acid in the colon of the L_V_ group were higher than those of the H_V_ group (*P* < 0.05). Acetic acid, propionic acid, butyric acid, valeric acid, isobutyric acid, and TSCFA in the cecum; propionic acid, butyric acid, and TSCFA in the colon; and propionic acid and isobutyric acid in the feces of the H_β/AX_ group were higher than those in the L_β/AX_ and M_β/AX_ groups (*P* < 0.05). At the same time, propionic acid, butyric acid, valeric acid, isobutyric acid, isovaleric acid, and TSCFA in the cecum; propionic acid, butyric acid, valeric acid, and TSCFA in the colon; and all SCFA in the feces of the L_β/AX_ group were lower than those in the M_β/AX_ group (*P* < 0.05). When comparing the 6 dietary treatments, there were differences in SCFA in the cecum, colon, and feces (*P* < 0.05). The dietary β-glucan-to-arabinoxylan ratios and apparent viscosity had linear interaction effect on the concentrations of acetic acid, propionic acid, butyric acid, isobutyric acid, isovaleric acid, and TSCFA in the cecum; propionic acid, butyric acid, valeric acid, and isovaleric acid in the colon; and acetic acid, butyric acid, and TSCFA in feces (*P* < 0.001). The dietary β-glucan-to-arabinoxylan ratios had linear effect on the concentrations of acetic acid, propionic acid, butyric acid, valeric acid, isobutyric acid and TSCFA in cecum; propionic acid, butyric acid, isovaleric acid and TSCFA in colon; and SCFA in feces of growing pigs (*P* < 0.05). The dietary β-glucan-to-arabinoxylan ratios had quadratic effect on the concentrations of valeric acid and isovaleric acid in cecum and colon, and propionic acid and valeric acid in feces of growing pigs.

**Table 6:** Effects of dietary fiber structure and apparent viscosity on short-chain fatty acids (SCFA) concentrations in different intestinal digesta and feces of growing pigs

Item	Viscosity		β-Glucan-to-arabinoxylan ratios			Treatments						SEM	*P*-value				
	L_V_	H_V_	L_β/AX_	M_β/AX_	H_β/AX_	L_V_L_β/AX_	L_V_M_β/AX_	L_V_H_β/AX_	H_V_L_β/AX_	H_V_M_β/AX_	H_V_H_β/AX_		V	β/AX	V × β/AX	Linear	Quadratic
SCFA production in the cecum, µmol/g																	
Acetic acid	37.06^n^	47.39^m^	34.91^y^	42.92^y^	48.85^x^	35.95^cd^	36.15^cd^	39.09^c^	33.86^d^	49.69^b^	58.60^a^	1.61	< 0.001	< 0.001	< 0.001	< 0.001	0.714
Propionic acid	14.24^n^	18.28^m^	11.17^z^	17.36^y^	20.26^x^	11.10^d^	15.04^c^	16.59^c^	11.24^d^	19.67^b^	23.92^a^	0.81	< 0.001	< 0.001	< 0.001	< 0.001	0.138
Butyric acid	4.21^n^	5.07^m^	3.58^z^	4.73^y^	5.60^x^	3.84^de^	4.28^cd^	4.52^e^	3.36^d^	5.17^b^	6.68^a^	0.19	< 0.001	< 0.001	< 0.001	< 0.001	0.652
Valeric acid	0.72	0.70	0.47^z^	0.75^y^	0.91^x^	0.46^d^	0.80^b^	0.91^a^	0.48^d^	0.70^c^	0.90^a^	0.03	0.230	< 0.001	0.066	< 0.001	0.001
Isobutyric acid	0.15^n^	0.18^m^	0.12^z^	0.17^y^	0.20^x^	0.08^c^	0.17^b^	0.19^ab^	0.16^b^	0.17^b^	0.21^a^	0.01	< 0.001	< 0.001	< 0.001	< 0.001	0.425
Isovaleric acid	0.33^m^	0.25^n^	0.23^y^	0.34^x^	0.31^x^	0.29^bc^	0.45^a^	0.26^c^	0.17^d^	0.24^cd^	0.34^b^	0.01	< 0.001	< 0.001	< 0.001	0.064	0.030
TSCFA	56.72^n^	71.86^m^	50.47^z^	66.27^y^	76.12^x^	51.72^d^	56.88^c^	61.56^c^	49.23^d^	75.66^b^	90.68^a^	2.55	< 0.001	< 0.001	< 0.001	< 0.001	0.456
SCFA production in the colon, µmol/g																	
Acetic acid	27.40^n^	32.77^m^	28.86^y^	29.86^xy^	31.54^x^	26.31^c^	27.30^c^	28.60^bc^	31.42^ab^	32.41^a^	34.48^a^	0.65	< 0.001	0.071	0.920	0.086	0.800
Propionic acid	12.39	12.12	8.92^z^	12.65^y^	15.20^x^	10.10^c^	12.38^b^	14.68^a^	7.73^d^	12.93^b^	15.71^a^	0.47	0.373	< 0.001	< 0.001	< 0.001	0.154
Butyric acid	5.45^n^	6.14^m^	4.69^z^	5.71^y^	7.00^x^	4.60^c^	5.66^b^	6.11^b^	4.79^c^	5.76^b^	7.88^a^	0.20	< 0.001	< 0.001	< 0.001	< 0.001	0.646
Valeric acid	0.79^m^	0.59^n^	0.60^y^	0.80^x^	0.66^xy^	0.67^b^	1.02^a^	0.68^b^	0.53^b^	0.58^b^	0.64^b^	0.03	< 0.001	< 0.001	< 0.001	0.396	0.043
Isobutyric acid	0.27	0.32	0.27	0.35	0.28	0.28^ab^	0.33^ab^	0.21^b^	0.26^ab^	0.37^a^	0.34^ab^	0.01	0.173	0.169	0.187	0.914	0.075
Isovaleric acid	0.38^m^	0.37^n^	0.44^x^	0.40^y^	0.28^z^	0.48^a^	0.43^b^	0.24^e^	0.40^bc^	0.38^c^	0.32^d^	0.01	0.100	< 0.001	< 0.001	< 0.001	0.012
TSCFA	46.50^n^	52.32^m^	43.78^z^	49.49^y^	54.96^x^	42.44^d^	46.55^cd^	50.52^bc^	45.12^d^	52.43^b^	59.40^a^	1.09	< 0.001	< 0.001	0.095	< 0.001	0.811
SCFA production in the feces, µmol/g																	
Acetic acid	17.77^n^	25.04^m^	17.83^y^	22.48^x^	23.90^x^	16.64^c^	17.11^bc^	19.56^b^	19.02^bc^	17.85^a^	28.26^a^	0.87	< 0.001	< 0.001	< 0.001	0.033	0.346
Propionic acid	7.21^n^	8.37^m^	5.80^z^	8.36^y^	9.20^x^	5.67^d^	7.58^c^	8.37^bc^	5.93^d^	9.14^ab^	10.02^a^	0.29	< 0.001	< 0.001	0.064	< 0.001	0.022
Butyric acid	3.51^n^	4.87^m^	2.84^y^	4.69^x^	5.03^x^	2.70^d^	3.75^bc^	4.06^b^	2.98^cd^	5.63^a^	6.01^a^	0.24	< 0.001	< 0.001	0.021	< 0.001	0.064
Valeric acid	0.63	0.61	0.39^y^	0.71^x^	0.76^x^	0.38^b^	0.74^a^	0.77^a^	0.40^b^	0.69^a^	0.75^a^	0.03	0.743	< 0.001	0.846	< 0.001	0.009
Isobutyric acid	0.36^n^	0.42^m^	0.32^z^	0.39^y^	0.46^x^	0.30^c^	0.36^bc^	0.41^b^	0.35^bc^	0.41^b^	0.50^a^	0.01	< 0.001	< 0.001	0.668	< 0.001	0.854
Isovaleric acid	0.58	0.74	0.52^y^	0.70^x^	0.76^x^	0.476^d^	0.57^cd^	0.68^bc^	0.57^cd^	0.82^ab^	0.84^a^	0.03	< 0.001	< 0.001	0.305	< 0.001	0.301
TSCFA	30.04^n^	40.06^m^	27.71^y^	37.33^x^	40.12^x^	26.18^c^	30.11^bc^	33.84^b^	29.24^c^	44.54^a^	46.39^a^	1.40	< 0.001	< 0.001	< 0.01	< 0.001	0.154

*L*_*V*_*L*_*β/AX*_ Diet with low viscosity and low β-glucan-to-arabinoxylan ratios, *L*_*V*_*M*_*β/AX*_ Diet with low viscosity and middle β-glucan-to-arabinoxylan ratios, *L*_*V*_*H*_*β/AX*_ Diet with low viscosity and high β-glucan-to-arabinoxylan ratios, *H*_*V*_*L*_*β/AX*_ Diet with high viscosity and low β-glucan-to-arabinoxylan ratios, *H*_*V*_*M*_*β/AX*_ Diet with high viscosity and middle β-glucan-to-arabinoxylan ratios, *H*_*V*_*H*_*β/AX*_ Diet with high viscosity and high β-glucan-to-arabinoxylan ratios, *SEM* Standard error of the mean, *V* Apparent viscosity of diets, *β/AX* β-glucan-to-arabinoxylan ratios of diets, *V × β/AX* Linear interaction effect between apparent viscosity and β-glucan-to-arabinoxylan ratios of diets, *TSCFA* Total short-chain fatty acids

^a−e^Different letters denote significant differences between experimental treatments within each short-chain fatty acids variable (*P* < 0.05)

^m,n^Different letters denote significant differences between the low and high viscosity groups within each short-chain fatty acids variable (*P* < 0.05)

^x,y,z^Different letters denote significant differences between the low, middle and high β-glucan-to-arabinoxylan ratios groups within each short-chain fatty acids variable (*P* < 0.05)

Linear contrasts analysis was used to test the linear and quadratic effects of β-glucan-to-arabinoxylan ratios on each short-chain fatty acids variable

The results of hepatic portal vein plasma SCFA concentration are presented in Table 7. It showed that acetic acid, butyric acid, and TSCFA in the HV group were higher than those in the L_V_ group (*P* < 0.05). Acetic acid, propionic acid, butyric acid, and TSCFA in the H_β/AX_ group were higher than in the L_β/AX_ and M_β/AX_ groups. In the H_β/AX_ group, valeric acid was higher than that in the L_β/AX_ group, and isobutyric acid was lower than in the L_β/AX_ and M_β/AX_ groups (*P* < 0.05). Acetic acid, propionic acid, butyric acid, and TSCFA in the M_β/AX_ group were higher than in the L_β/AX_ group, whereas isobutyric acid was lower than in the L_β/AX_ group (*P* < 0.05). When the 6 dietary treatments were compared, there were differences in all SCFA (*P* < 0.05). The dietary β-glucan-to-arabinoxylan ratios and apparent viscosity had linear interaction effect on propionic acid and butyric acid absorption (*P* < 0.05). The dietary β-glucan-to-arabinoxylan ratios had linear effect on hepatic portal vein plasma SCFA concentration of growing pigs (*P* < 0.001).

**Table 7:** Effects of dietary fiber structure and apparent viscosity on short-chain fatty acids (SCFA) concentrations in hepatic portal vein plasma of growing pigs

Item	Viscosity		β-Glucan-to-arabinoxylan ratios			Treatments						SEM	*P*-value				
	L_V_	H_V_	L_β/AX_	M_β/AX_	H_β/AX_	L_V_L_β/AX_	L_V_M_β/AX_	L_V_H_β/AX_	H_V_L_β/AX_	H_V_M_β/AX_	H_V_H_β/AX_		V	β/AX	V × β/AX	Linear	Quadratic
SCFA concentrations in hepatic portal vein plasma, µmol/mL																	
Acetic acid	3.47^n^	4.18^m^	3.40^z^	3.79^y^	4.29^x^	3.03^d^	3.50^c^	3.89^bc^	3.77^bc^	4.09^b^	4.68^a^	0.10	< 0.001	< 0.001	0.793	< 0.001	0.784
Propionic acid	0.25	0.25	0.15^z^	0.26^y^	0.35^x^	0.16^c^	0.24^b^	0.34^a^	0.13^c^	0.28^b^	0.35^a^	0.01	0.555	< 0.001	0.047	< 0.001	0.234
Butyric acid	0.35^n^	0.56^m^	0.38^z^	0.43^y^	0.56^x^	0.29^e^	0.34^d^	0.42^c^	0.47^b^	0.51^b^	0.70^a^	0.02	< 0.001	< 0.001	< 0.001	< 0.001	0.309
Valeric acid	0.11	0.11	0.09^y^	0.11^xy^	0.12^x^	0.11^ab^	0.12^a^	0.12^a^	0.08^b^	0.11^ab^	0.13^a^	0.05	0.327	0.032	0.394	< 0.001	0.662
Isobutyric acid	0.58	0.57	0.66^x^	0.56^y^	0.49^z^	0.68^a^	0.59^b^	0.47^d^	0.65^a^	0.54^bc^	0.51^cd^	0.01	0.374	< 0.001	0.050	< 0.001	0.414
Isovaleric acid^1^	-	-	-	-	-	-	-	-	-	-	-	-	-	-	-	-	-
TSCFA	4.76^n^	5.67^m^	4.69^z^	5.16^y^	5.80^x^	4.27^d^	4.79^c^	5.23^bc^	5.11^bc^	5.52^b^	6.27^a^	0.13	< 0.001	< 0.001	0.454	< 0.001	0.683

*L*_*V*_*L*_*β/AX*_ Diet with low viscosity and low β-glucan-to-arabinoxylan ratios, *L*_*V*_*M*_*β/AX*_ Diet with low viscosity and middle β-glucan-to-arabinoxylan ratios, *L*_*V*_*H*_*β/AX*_ Diet with low viscosity and high β-glucan-to-arabinoxylan ratios, *H*_*V*_*L*_*β/AX*_ Diet with high viscosity and low β-glucan-to-arabinoxylan ratios, *H*_*V*_*M*_*β/AX*_ Diet with high viscosity and middle β-glucan-to-arabinoxylan ratios, *H*_*V*_*H*_*β/AX*_ Diet with high viscosity and high β-glucan-to-arabinoxylan ratios, *SEM* Standard error of the mean, *V* Apparent viscosity of diets, *β/AX* β-glucan-to-arabinoxylan ratios of diets, *V × β/AX* Linear interaction effect between apparent viscosity and β-glucan-to-arabinoxylan ratios of diets, *TSCFA* Total short-chain fatty acids

^a−e^Different letters denote significant differences between experimental treatments within each short-chain fatty acids variable (*P* < 0.05)

^m,n^Different letters denote significant differences between the low and high viscosity groups within each short-chain fatty acids variable (*P* < 0.05)

^x,y,z^Different letters denote significant differences between the low, middle and high β-glucan-to-arabinoxylan ratios groups within each short-chain fatty acids variable (*P* < 0.05)

^1^﻿Isovaleric acid was not detected in the hepatic portal vein plasma

Linear contrasts analysis was used to test the linear and quadratic effects of β-glucan-to-arabinoxylan ratios on each short-chain fatty acids variable

### Dietary fiber structure and apparent viscosity modulated the fecal bacterial community

**Fig. 3:**
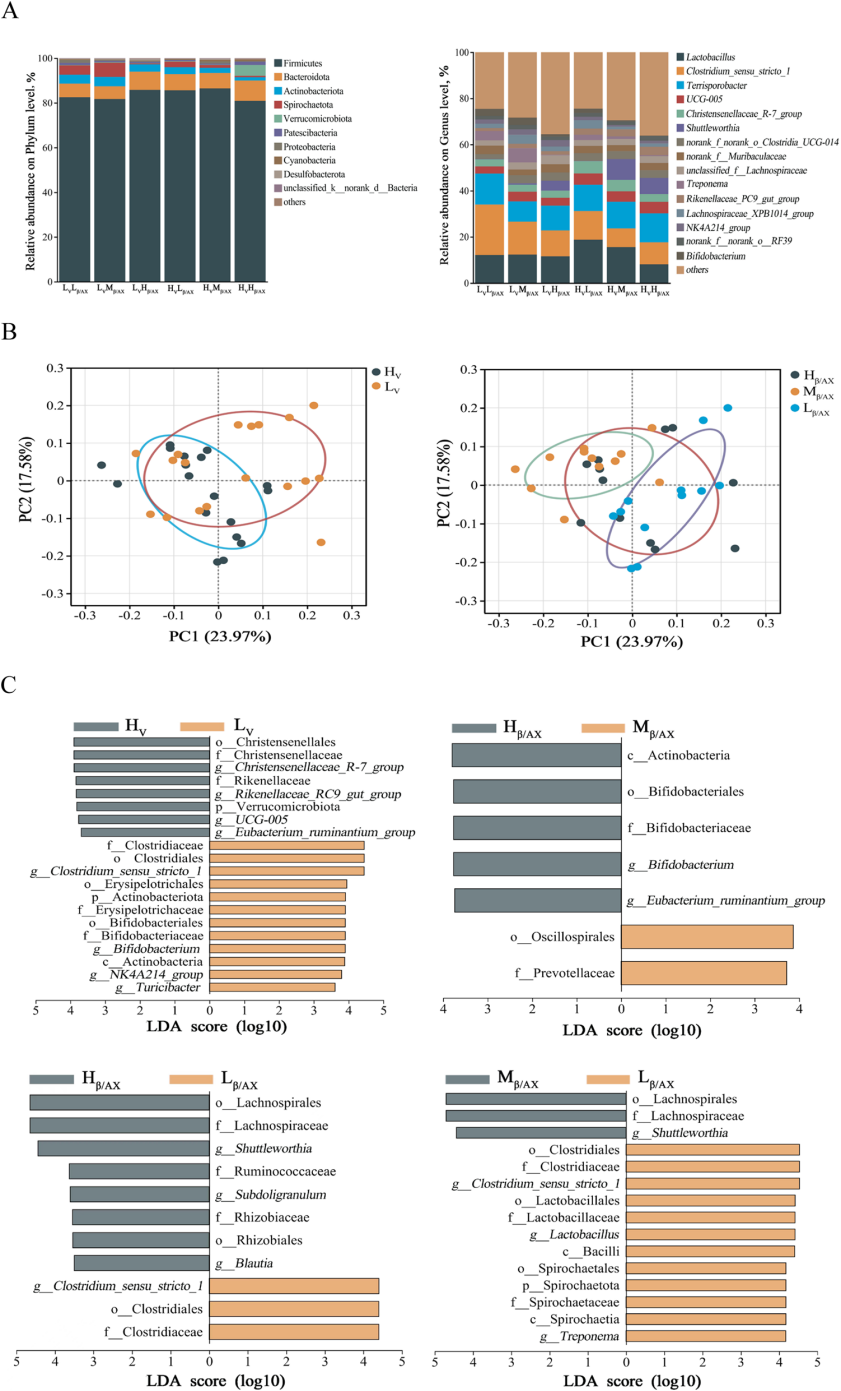
Effects of dietary fiber structure and apparent viscosity on fecal microbial community. Microbial composition at phylum and genus level (**A**). Under the factors of fiber structure and apparent viscosity, PCoA analysis of microbial community structure at the level of amplicon sequence variants taxonomy (**B**) and linear discriminant analysis (LDA) > 3.5 (**C**) were performed

The α-diversity of the microbial community (Table S2) showed that the Sobs and Chao index in the H_V_ group were higher than those in the L_V_ group (*P* < 0.05). The Sobs, Chao index, and Shannon index of the L_β/AX_ group were lower than those of the H_β/AX_ groups (*P* < 0.001), while the Simpson index was higher than that of the H_β/AX_ group (*P* < 0.05). The dietary β-glucan-to-arabinoxylan ratios and apparent viscosity interacted with the Chao index (*P* < 0.05). The dietary β-glucan-to-arabinoxylan ratios had linear effect on the Sobs, Chao index, Shannon index, and Simpson index (*P* < 0.05). The microbiota composition under the 6 dietary treatments are shown in Fig. 3A. A phylum-level analysis showed that Firmicutes and Bacteroidota consistently dominated the microbiota composition in pigs’ feces. *Lactobacillus*, *Clostridium_sensu_stricto_1*, and *Terrisporobacter* were the dominant bacteria at the genus level. A binary Pearson distance matrix was constructed based on the ASV of each treated sample to analyze the microbial community structure. The results of PCoA showed that changes in dietary apparent viscosity and β-glucan-to-arabinoxylan ratios affected the microbial community and functional structure (*P* < 0.05; Fig. 3B).

The LEFSe analysis results of the microbial composition between the different treatment groups under the conditions of dietary β-glucan-to-arabinoxylan ratios and apparent viscosity are shown in Fig. 3C. The comparison results between the H_V_ and L_V_ groups showed enrichment of bacteria such as *Christensenellaceae R-7_group*, *Rikenellaceae RC9_gut_group*, *UCG-005*, and *Eubacteria ruminantium_group* in the H_V_ group, while bacteria such as *Clostridium_sensu_stricto_1*, *Bifidobacterium*, *NK4A214_group*, and *Turiciactor* were enriched in the L_V_ group. Compared with the M_β/AX_ group, *Bifidobacterium* and *Eubacterium_ruminantium_group* in the H_β/AX_ group increased, while the abundance of Oscillospirales and Prevotellaceae decreased. Compared with the L_β/AX_ group, the abundance of Lachnospiraceae, *Shuttleworthia*, *Subdoligranulum*, and *Blautia* increased in the H_β/AX_ group, whereas the abundance of *Clostridium_sensu_stricto_1* decreased. The comparison between the M_β/AX_ and L_β/AX_ groups showed that Lachnospiraceae and *Shuttleworthia* bacteria were enriched in the M_β/AX_ group, while *Clostridium_sensu_stricto_1*, *Lactobacillus*, Spirochaetaceae and *Treponema* were enriched in L_β/AX_ group.

### Effects of dietary fiber structure and apparent viscosity on short-chain fatty acid production during in vitro fermentation of ileal digesta

To verify the fermentation potential of diets with different apparent viscosities and fiber structures in the hindgut of pigs, we used ileal digesta collected from Exp. 2 as a substrate for in vitro fermentation and observed the SCFA production (Table 8). The results showed that compared to the L_V_ group, the H_V_ group had an increased butyric acid and a decreased isobutyric acid (*P* < 0.05). Acetic acid, propionic acid, butyric acid, valeric acid, isovaleric acid, and TSCFA in the H_β/AX_ group were higher than those in the L_β/AX_ and M_β/AX_ groups (*P* < 0.05). Isobutyric acid levels in the L_β/AX_ group were lower than in the M_β/AX_ and H_β/AX_ groups (*P* < 0.05). When the 6 dietary treatments were compared, there were differences in all SCFA and interaction effects (*P* < 0.05). The dietary β-glucan-to-arabinoxylan ratios had linear effect on SCFA production (*P* < 0.05). Meanwhile, the dietary β-glucan-to-arabinoxylan ratios had quadratic effect on acetic acid, valeric acid, isobutyric acid and TSCFA production (*P* < 0.05).

**Table 8:** Effects of dietary fiber structure and apparent viscosity on short-chain fatty acids (SCFA) production during in vitro fermentation of ileal digesta of growing pigs

Item	Viscosity		β-Glucan-to-arabinoxylan ratios			Treatments						SEM	*P*-value				
	L_V_	H_V_	L_β/AX_	M_β/AX_	H_β/AX_	L_V_L_β/AX_	L_V_M_β/AX_	L_V_H_β/AX_	H_V_L_β/AX_	H_V_M_β/AX_	H_V_H_β/AX_		V	β/AX	V × β/AX	Linear	Quadratic
SCFA concentrations (at 72 h of in vitro fermentation), µmol/mL																	
Acetic acid	14.97	15.27	14.03^z^	14.92^y^	16.41^x^	13.94^e^	14.89^c^	16.08^b^	14.12^d^	14.95^c^	16.74^a^	0.17	0.389	< 0.001	< 0.001	< 0.001	< 0.001
Propionic acid	11.69	11.31	10.92^y^	11.25^y^	12.33^x^	10.39^d^	12.42^a^	12.26^b^	11.45^c^	10.08^e^	12.39^a^	0.16	0.244	< 0.001	< 0.001	< 0.001	0.182
Butyric acid	4.68^n^	5.39^m^	4.72^y^	4.94^y^	5.45^x^	4.37^f^	4.53^e^	5.14^c^	5.07^d^	5.36^b^	5.76^a^	0.08	< 0.001	< 0.001	< 0.001	< 0.001	0.300
Valeric acid	3.05	3.07	3.03^y^	3.04^y^	3.12^x^	3.05^c^	3.04^d^	3.07^b^	3.01^e^	3.03^d^	3.16^a^	0.01	0.404	< 0.001	< 0.001	< 0.001	< 0.001
Isobutyric acid	1.13^m^	1.07^n^	1.06^y^	1.13^x^	1.10^x^	1.12^b^	1.13^a^	1.13^a^	1.01^d^	1.13^a^	1.08^c^	0.01	< 0.001	< 0.001	< 0.001	< 0.001	< 0.001
Isovaleric acid	1.04	1.03	1.05^z^	1.04^y^	1.03^x^	1.05^ab^	1.04^b^	1.03^c^	1.05^a^	1.03^d^	1.02^d^	0.01	0.066	< 0.001	< 0.001	< 0.001	0.247
TSCFA	36.56	37.14	34.81^z^	36.31^y^	39.43^x^	33.91^e^	37.05^c^	38.72^b^	35.71^d^	35.57^d^	40.15^a^	0.35	0.423	< 0.001	< 0.001	< 0.001	0.010

*L*_*V*_*L*_*β/AX*_ Diet with low viscosity and low β-glucan-to-arabinoxylan ratios, *L*_*V*_*M*_*β/AX*_ Diet with low viscosity and middle β-glucan-to-arabinoxylan ratios, *L*_*V*_*H*_*β/AX*_ Diet with low viscosity and high β-glucan-to-arabinoxylan ratios, *H*_*V*_*L*_*β/AX*_ Diet with high viscosity and low β-glucan-to-arabinoxylan ratios, *H*_*V*_*M*_*β/AX*_ Diet with high viscosity and middle β-glucan-to-arabinoxylan ratios, *H*_*V*_*H*_*β/AX*_ Diet with high viscosity and high β-glucan-to-arabinoxylan ratios, *SEM* Standard error of the mean, *V* Apparent viscosity of diets, *β/AX* β-glucan-to-arabinoxylan ratios of diets, *V × β/AX* Linear interaction effect between apparent viscosity and β-glucan-to-arabinoxylan ratios of diets, *TSCFA* Total short-chain fatty acids

^a−f^Different letters denote significant differences between experimental treatments within each short-chain fatty acids variable (*P* < 0.05)

^m,n^Different letters denote significant differences between the low and high viscosity groups within each short-chain fatty acids variable (*P* < 0.05)

^x,y,z^Different letters denote significant differences between the low, middle and high β-glucan-to-arabinoxylan ratios groups within each short-chain fatty acids variable (*P* < 0.05)

Linear contrasts analysis was used to test the linear and quadratic effects of β-glucan-to-arabinoxylan ratios on each short-chain fatty acids variable

### Prediction of RE and NE of diets

To accurately predict dietary RE and NE in growing pigs, we used dietary chemical components and physical properties as predictors and established the 6 best equations using a multiple linear regression model (Table 9). Overall, RE and NE can be well predicted when dietary chemical components are combined with β-glucan-to-arabinoxylan ratios and apparent viscosity (with *R*^2^ ≥ 0.87 and *P* < 0.01 for the RE prediction equation and *R*^2^ ≥ 0.90 and *P* < 0.01 for the NE prediction equation).

**Table 9:** Prediction of retained energy (RE) and net energy (NE) of diets (MJ/kg DM) from dietary chemical composition and physical properties

Numbers	Regressive equation	*R*^2^	*P*-value
1	RE = 14.22 + 0.26 TDF (%) − 2.16 β-glucan-to-arabinoxylan ratios − 11.71 V (cP)	0.87	< 0.001
2	RE = 277.06 − 7.21 GE (MJ/kg) + 3.21 EE (%) − 2.64 NFE (%) − 15.44 Ash (%) + 32.69 β-glucan-to-arabinoxylan ratios	0.88	< 0.001
3	RE = 314.31 − 20.01 GE (MJ/kg) + 5.38 EE (%) − 0.53 CF (%) + 2.03 TDF (%) − 43.93 V (cP)	0.88	< 0.001
4	NE = 13.33 + 0.55 TDF (%) − 1.12 β-glucan-to-arabinoxylan ratios − 14.14 V (cP)	0.90	< 0.001
5	NE = 103.44 + 2.42 GE (MJ/kg) − 0.66 EE (%) − 2.08 NFE (%) − 9.34 Ash (%) + 20.49 β-glucan-to-arabinoxylan ratios	0.90	< 0.001
6	NE = 67.44 − 4.83 GE (MJ/kg) + 0.97 EE (%) − 0.27 CF (%) + 2.13 TDF (%) − 26.07 V (cP)	0.91	< 0.001

*TDF* Total dietary fiber, *V* Apparent viscosity, *GE* Gross energy, *EE* Ether extract, *CF* Crude fiber, *NFE* Nitrogen-free extract

## Discussion

The use of dietary fibers in the development of the pig industry and the benefits in terms of intestinal health have been widely reported [5, 39–42], but the relationship between the structural and physical properties of the fibers and the fermentation characteristics of the feedstuffs and their interactions have not yet been thoroughly investigated in terms of their effects on nutrients and energy utilization in pigs. Previous studies have generally focused on fiber sources (plant or purified fiber), fiber level, fiber solubility, and other aspects to reveal their functions and application effects [43–46]. However, there are still many negative and positive heterogeneities among the results of various studies, especially concerning energy nutrition. In this study, we first used in vitro digestion and fermentation models to evaluate the fermentation dynamics of 13 conventional and non-conventional feedstuffs and explored the relationship between feedstuff fermentation characteristics and fiber physicochemical properties. We found differences in the fermentation characteristics of different feedstuffs in the hindgut of pigs (in vitro), which were closely related to the physicochemical properties of the fibers. Among them, the β-glucan-to-arabinoxylan ratios and apparent viscosity had a linear relationship with TSCFA and DMCV. Then, the effects of dietary β-glucan-to-arabinoxylan ratios and apparent viscosity as factors on nutrient utilization, energy metabolism, and intestinal microbial community of growing pigs were studied, with NE, SID CP, and TDF levels relatively constant. According to the results of the present study, dietary β-glucan-to-arabinoxylan ratios and apparent viscosity have different degrees of interaction and independent effects on nutrient utilization and energy metabolism, as well as regulating the intestinal microbial community in growing pigs. In general, an increase in the dietary apparent viscosity and β-glucan-to-arabinoxylan ratios, the AID, ATTD, and HD of fiber components increased, especially the HD of nutrients (degree of fermentation), and the production and absorption of SCFA and relative abundance of beneficial microorganisms being promoted. However, this increase in fermentation efficiency did not further increase the RE and NE. Lower dietary apparent viscosity and β-glucan-to-arabinoxylan ratios decreased the digestibility of fiber components and the SCFA production but increased energy use efficiency. This indicates that diets with different apparent viscosity and β-glucan-to-arabinoxylan ratios changed the utilization pattern of energy substances in growing pigs under the same nutrient level conditions. Diets with high apparent viscosity and β-glucan-to-arabinoxylan ratios affect energy allocation in a manner that increases heat and gas production, which in turn decreases energy deposition efficiency. Subsequently, the effects of dietary apparent viscosity and the β-glucan-to-arabinoxylan ratios on fermentation potential and SCFA production in the hindgut of pigs were further verified by in vitro fermentation experiments using ileal digesta as a substrate. It was found that the dietary apparent viscosity and β-glucan-to-arabinoxylan ratios had an additive effect when formulating the diets. The SCFA production after fermentation improved with increased dietary apparent viscosity and β-glucan-to-arabinoxylan ratios. Finally, we combined the dietary fiber composition, physical properties, and other chemical components to establish an optimal prediction equation for RE and NE.

Presently, there are few studies on the fermentation characteristics of conventional and non-conventional feedstuffs in the hindgut of growing pigs. The SCFA and gases are usually produced by the fermentation of nutrients that are not digested in the stomach and small intestine of pigs under the action of hindgut microorganisms [47]; therefore, the SCFA and gas production is an essential index for evaluating the fermentation characteristics of feedstuffs [48, 49]. This study demonstrated the differences in SCFA and DMCV production between different feedstuffs when fermented in vitro. This is consistent with some previous reports and assumptions because the change in substrate chemical composition will affect the fermentation efficiency of the feedstuff itself [48, 50]. However, it is essential to note that, compared to the results obtained by in vitro fermentation of purified or synthetic fibers as substrates [11, 49], the TSCFA and DMCV trends were not all consistent across feedstuffs. This shows that purified and synthetic fibers are not equivalent to natural plant-derived fibers and have different fermentation patterns. Moreover, from the perspective of the current generation pathways and mechanisms of SCFA, in addition to the consumption of CO_2_ by acetyl-CoA in the ketovalerate pathway through acetic acid, the generation of propionic acid, butyric acid, and branched-chain fatty acids releases CO_2_ or H_2_ through complex pathways such as succinic acid pathway and acrylic ester pathway [51]. For example, the generation of one butyric acid molecule usually requires the production of two CO_2_ molecules through the butyrate kinase pathway, whereas isobutyric acid and isovaleric acid are more abundant [52, 53]. This may help us understand why some feedstuffs, such as corn gluten and alfalfa meals, have higher DMCV and relatively fewer TSCFA. Compared to other feedstuffs, they had relatively higher levels of isovaleric acid and isobutyric acid during the fermentation process, whereas the yields of acetic acid and propionic acid were consistently lower. A similar phenomenon was reported by Mou et al. [50] when evaluating the fermentation characteristics of nine feedstuffs using an in vitro gestating sow model; however, no further discussion was provided. In addition, unlike the results of a study in which feedstuffs were directly fermented without pre-digestion [48], both soybean husks and corn husks showed a higher DMCV than wheat bran, which may be related to the fact that the fermentation of the feedstuffs in this experiment was based on the leftover digested residue as the substrate.

In recent years, an increasing number of studies have linked the fermentation potential of fibers to their solubility and have pointed to the high fermentability of sugar beet pulp owing to its high SDF and the low fermentability of corn DDGS due to its richness in IDF [54]. In addition, the fermentation of fiber mixtures with different SDF-to-IDF ratios of purified inulin and NSP combinations as substrates was found to increase the fermentability of the fibers with increasing SDF [11]. However, some in vivo studies have found that the concentration of TSCFA in growing pig feces was positively correlated with the digestibility of IDF but not with that of SDF [12]. This suggests that limitations still exist when interpreting the fermentability of fibrous feedstuffs in terms of fiber classification systems. Therefore, to further investigate the quantitative relationship between the fermentation characteristics of feedstuffs and their fiber physicochemical properties, we performed a correlation analysis between each fiber component and fermentation-related indexes and found that the fermentation characteristics of feedstuffs in the hindgut of growing pigs were closely related to their fiber physicochemical properties. Regression analyses confirmed a linear relationship between the β-glucan-to-arabinoxylan ratios and apparent viscosity with TSCFA and DMCV. This implies that the dietary β-glucan-to-arabinoxylan ratios and apparent viscosity play important roles in regulating the fermentation potential of fibrous feedstuffs. Previous studies have shown that the molecular structure of β-glucan is simpler than that of arabinoxylan and is more easily degraded by gut microbiota to produce SCFA [55, 56], which is consistent with the positive correlation between the β-glucan-to-arabinoxylan ratios and TSCFA in this study. Furthermore, viscous fibers may increase the contact time between fibers and microbial communities, thereby improving the degradation efficiency of fiber components [57].

In the in vivo experiments, 6 diets were formulated, and the same levels of NE, SID CP, and TDF were obtained in each group of pigs. Compared with the L_V_ group, the AID and ATTD of DM and OM in the H_V_ group decreased, whereas the AID, ATTD, and HD in TDF, SDF, and IDF increased, indicating that dietary apparent viscosity has different effects on the digestibility of different nutrient components. Previous studies have shown that low-viscosity inulin can increase the transport rate of ileal digesta, while high-viscosity carboxymethyl cellulose can decrease the transport rate of ileal digesta and increase the contact time of carbohydrates with carbohydrate enzymes and intestinal microorganisms, thus improving the digestibility of carbohydrate components [58]. This could explain the improved digestibility of the fiber components in diets with high apparent viscosity. In addition, Hung et al. [59] adjusted dietary viscosity to feed growing pigs with purified fibers (5% non-viscose cellulose, 6.5% medium viscose carboxymethyl cellulose, and 6.5% high viscose carboxymethyl cellulose) and found that the AID of DM, CP, and other nutrients was decreased with the increase of dietary viscosity, and this effect was independent of fiber content. This is consistent with the results obtained in this study by adjusting the apparent viscosity of natural feedstuffs to formulate rations with different viscosities, suggesting that the viscosity characteristics of the diets themselves have an important effect on the efficiency of nutrient utilization in pigs. When the β-glucan-to-arabinoxylan ratios were adjusted, it was found that increasing the β-glucan-to-arabinoxylan ratios in the diet reduced the AID and ATTD of DM and OM but increased HD. Simultaneously, the AID, ATTD, and HD of the TDF, SDF, and IDF increased. The nutrient digestibility results of different intestinal segments indicated that changes in fiber structure could affect the digestibility of fiber components in the intestines of growing pigs. This reiterates our findings from Exp. 1, which suggest that the β-glucan-to-arabinoxylan ratios in diet can regulate the degree of digestion, especially the fermentation potential in the hindgut. In previous studies, Wilfart et al. [60] used growing pigs with double fistulas in the duodenum and ileum to study the effects of wheat bran fiber on nutrient digestibility of the stomach, small intestine, and hindgut and found that with an increase in wheat bran fiber supplementation level (16.5%, 21%, and 27%), the AID of CP, ash, DM, OM, and TDF showed an increasing trend, while ATTD decreased. This indicates that an increase in wheat bran fiber levels may reduce the HD of nutrients in growing pigs. This is similar to increasing the dietary β-glucan-to-arabinoxylan ratios in this study, which increased the nutrient HD because wheat bran fiber is rich in arabinoxylan and low in β-glucan. In contrast, the digestibility of fiber components was higher than wheat straw and wheat bran fibers when growing pigs were fed sugar beet pulp rich in β-glucan as a fiber source [61]. This is consistent with the fact that an increase in β-glucan-to-arabinoxylan ratios in this study is beneficial for the utilization of fiber components. Additionally, dietary apparent viscosity and β-glucan-to-arabinoxylan ratios have interactive effects on the ATTD and HD of fiber components, indicating that the utilization of fiber components can be further improved by increasing the β-glucan-to-arabinoxylan ratios under conditions of high apparent viscosity. This effect may be caused by the increased apparent viscosity of the diet extending the residence time of digesta in the pig intestine, thus giving the indigestible nutrients a fuller microbial fermentation time in the hindgut [62]. At the same time, as found in Exp.1, appropriately increasing the β-glucan-to-arabinoxylan ratios improved the fermentation capacity of the diet. It is worth noting that in this study, the effect of improving fiber component utilization mediated by regulating fiber physicochemical properties did not further increase the energy use efficiency of growing pigs. In contrast, diets with low apparent viscosity and low β-glucan-to-arabinoxylan ratios were more conducive to energy deposition. Compared with the L_V_ group, the H_V_ group showed increased FE, UE, O_2_ consumption, CO_2_ excretion, CH_4_ excretion, CH_4_ energy, THP, HI, and OXP, and decreased DE, ME, RE, and NE. Recent reports on fiber viscosity characteristics and pig nutrition have shown that β-glucan (viscous purified fiber) can increase the residence time of the liquid portion of digesta in the stomach of growing pigs and reduce the separation of solid and liquid portions of digesta [21]. However, a large number of nutrients that have been degraded or released are dissolved in the liquid portion of digesta to a large extent, and the change in their residence time may change the absorption dynamics of nutrients and affect the deposition. In addition, the viscosity characteristics of fiber may also affect the contact between digestive enzymes and the digesta matrix and interact with the intestinal mucosa to form an absorption barrier, thereby regulating the homeostasis of nutrient metabolism [63–65]. This viscosity effect may change the release time and spatial distribution of nutrients in the gastrointestinal tract, regulate the synchronicity or heterogeneity of nutrient release, and thus affect their metabolism and deposition. On the other hand, it may also increase the oxidative energy supply of some fatty acids and amino acids. Moreover, changes in the physical and chemical properties of fiber can affect the viscosity of the digesta and then affect the diffusion of nutrients in the digesta in the intestinal lumen, altering the energy distribution and deposition efficiency. For example, guar gum and carboxymethylcellulose increase the viscosity of pig digesta and inhibit the activity of digestive enzymes, thereby reducing the energy utilization rate [63, 66]. This is consistent with the results of this study, which show that high apparent viscosity increases metabolic heat production, gas exchange capacity, and nutrient oxidation in growing pigs while decreasing energy deposition. At the same time, diets with low β-glucan-to-arabinoxylan ratios had more positive effects on growing pigs’ energy utilization than those with a medium to high ratio. This is similar to previous reports by Lee et al. [44], who found that high soluble fiber (sugar beet pulp, potato pomace, and pectin) compared to low soluble fiber (pea hulls and ryegrass) increased UE, CH_4_ energy, and THP, while decreasing RE and NE, in growing pigs. The dietary apparent viscosity and β-glucan-to-arabinoxylan ratios had an interaction effect on FE, DE, RE, and NE, indicating that increasing the β-glucan-to-arabinoxylan ratios can further reduce energy use efficiency under high apparent viscosity dietary substrate conditions and vice versa. Previous studies have shown that SCFA produced in the hindgut can provide 28% of the maintenance energy requirements of growing pigs and may be higher in gestating sows [67, 68]. In the present study, we found that the production of SCFA in the hindgut and the absorption of SCFA in the hepatic portal vein of growing pigs increased with increasing dietary apparent viscosity and β-glucan-to-arabinoxylan ratios. This is consistent with the increased HD of the fiber fraction due to the increase in dietary apparent viscosity and the β-glucan-to-arabinoxylan ratios and may be related to the decrease in energy utilization. This is because SCFA are usually less efficient in energy supply than the direct utilization of nutrients by the organism [68]. Nevertheless, SCFA have a positive regulatory role as signaling molecules that regulate the body’s energy metabolism, glucolipid metabolism, intestinal health, and immune homeostasis, among other important life activities [44, 66, 69].

Gut microbial flora and their metabolites have an essential impact on host nutrient utilization [70]; therefore, we investigated the effects of dietary fiber physicochemical properties on the structure and composition of fecal microbial communities. The results showed that the dietary apparent viscosity and β-glucan-to-arabinoxylan ratios modulated the fecal microbial community of growing pigs. Previous studies have shown that bacteria such as *Christensenellaceae_R-7_group*, *Rikenellaceae_RC9_gut_group*, *UCG-005*, and *Eubacterium_ruminantium_group* are commonly associated with fiber digestion [71, 72]. This may be related to the higher digestibility of fiber components in high viscosity diets compared to low viscosity diets in this study. Consistent with the present study, previous studies have found that both pectin and fucoidan can increase *Christensenellaceae_R-7_group* [73, 74], suggesting that this bacterium may have a specific preference for viscous feedstuffs or nutrient matrices. *Rikenellaceae_RC9_gut_group*, *UCG-005*, and *Eubacterium_ruminantium_group* are involved in cell wall degradation and polysaccharide fermentation, which play important roles in the production of SCFA [75, 76]. In addition, it has been demonstrated that the *Christensenellaceae_R-7_group* regulates body lipid metabolism and thus resists the onset of obesity [77], which may be related to the reduction of energy utilization in growing pigs by the high apparent viscosity diet in this study. Compared to the L_β/AX_ group, the M_β/AX_ and H_β/AX_ groups increased the abundance of beneficial bacteria such as Lachnospiraceae and *Shuttleworthia*. Huang et al. [78] found that a high-fat diet-induced gestational diabetes mouse model was enriched in Lachnospiraceae when supplemented with highly fermentable dietary fiber and resisted placental inflammation and adverse pregnancy outcomes through the mediation of butyric acid. In addition, reduced *Shuttleworthia* abundance is usually associated with increased inflammatory factors [79]. Dietary apparent viscosity and β-glucan-to-arabinoxylan ratios positively regulate gut microbes, which may have important potential in modulating individual health and ameliorating nutrient metabolic diseases.

In order to verify the fermentation potential of diets with different apparent viscosities and fiber structures in the pig hindgut, we performed in vitro fermentation using ileal digesta as a substrate and explored the effect on the production of SCFA. The results showed an increase in butyric acid and a decrease in isobutyric acid in the H_V_ group compared to the L_V_ group. Acetic acid, propionic acid, butyric acid, valeric acid, isovaleric acid, and TSCFA were higher in the H_β/AX_ group than in the L_β/AX_ and M_β/AX_ groups. This suggests that feedstuff’s apparent viscosity and β-glucan-to-arabinoxylan ratios have a cumulative effect on the formulation of diets and can influence the fermentation potential of nutrients in the hindgut and the ability to produce SCFA.

Finally, this study established the 6 best prediction equations for dietary RE and NE by combining dietary chemical components, fiber components, and physical properties. From the equations, it was found that apparent viscosity and β-glucan-to-arabinoxylan ratios mainly determined the dietary RE and NE compared to other dietary chemical components.

## Conclusion

Dietary fiber physicochemical properties are related to the fermentation efficiency of feedstuff and diet and have different degrees of interaction and independent effects on the nutrition and metabolism of pigs. Under appropriate dietary fiber level conditions, increasing apparent viscosity and β-glucan-to-arabinoxylan ratios in a pig diet can improve the digestion of fiber components and increase the abundance of beneficial bacteria and SCFA production and absorption while reducing apparent viscosity and β-glucan-to-arabinoxylan ratios is more conducive to energy utilization. This study provides a scientific basis for further utilizing fibrous feedstuffs and formulating diversified diets.

## Supplementary Information

Additional file 1: Table S1. In vitro ileal digestibility of dry matter and gross energy of different feedstuffs. Table S2. Effects of dietary fiber structure and apparent viscosity on α-diversity of the fecal microbial community. Fig. S1. Production of short-chain fatty acid at various time points during in vitro fermentation of different feedstuffs. Fig. S2. Gas production at various time points during in vitro fermentation of different feedstuffs.

## Data Availability

The data analyzed during the current study are available from the corresponding author on reasonable request.

## Abbreviations

ADF: Acid detergent fiber
ADL: Acid detergent lignin
AID: Apparent ileal digestibility
ASV: Amplicon sequence variants
ATTD: Apparent total tract digestibility
BW: Bulk weight
CF: Crude fiber
Corn DDGS: Corn distillers dried grains with solubles
CP: Crude protein
DE: Digestible energy
DM: Dry matter
DMCV: Dry matter correction gas production
EE: Ether extract
FE: Fecal energy
FHP: Fasting heat production
GE: Gross energy
HI: Heat increment
HD: Hindgut digestibility
IDF: Insoluble dietary fiber
LEFSe: Linear discriminant analysis effect size
ME: Metabolizable energy
NDF: Neutral detergent fiber
NE: Net energy
NFE: Nitrogen-free extract
NSP: Non-starch polysaccharides
OM: Organic matter
OXCHO: Carbohydrate oxidation
OXP: Protein oxidation
PCoA: Principal co-ordinates analysis
RE: Retained energy
RQ: Respiratory quotient
SC: Swelling capacity
SDF: Soluble dietary fiber
SID: Standardized ileal digestible
TDF: Total dietary fiber
THP: Total heat production
TSCFA: Total short-chain fatty acid
UE: Urinary energy
UN: Urinary nitrogen
WHC: Water holding capacity
β/AX: β-Glucan-to-arabinoxylan ratios
