# Supplementary material for: Relationship between dietary fiber physicochemical properties and feedstuff fermentation characteristics and their effects on nutrient utilization, energy metabolism, and gut microbiota in growing pigs
Source: J Anim Sci Biotechnol. 2025 Jan 2;16:1. doi: 10.1186/s40104-024-01129-x (PMC11697959; doi:10.1186/s40104-024-01129-x)
Supplement: Supplementary file 1 — Additional file 1: Table S1. In vitro ileal digestibility of dry matter and gross energy of different feedstuffs. Table S2. Effects of dietary fiber structure and apparent viscosity on α-diversity of the fecal microbial community. Fig. S1. Production of short-chain fatty acid at various time points during in vitro fermentation of different feedstuffs. Fig. S2. Gas production at various time points during in vitro fermentation of different feedstuffs. [file 40104_2024_1129_MOESM1_ESM.docx]

**Relationship between dietary fiber** **physicochemical properties and feedstuff fermentation characteristics and their effects on** **nutrient utilization, energy metabolism, and gut microbiota in growing pigs**

Feng Yong^1†^, Bo Liu^1†^, Huijuan Li^1^, Houxu Hao^1^, Yueli Fan^1^, Osmond Datsomor^1^, Rui Han^1^, Hailong Jiang^1*^, and Dongsheng Che^1*^

^1^Key Laboratory of Animal Production, Product Quality and Security, Ministry of Education, Jilin Provincial Key Laboratory of Animal Nutrition and Feed Science, Jilin Provincial Science and Technology Innovation Center of Pig Industry Technology, College of Animal Science and Technology, Jilin Agricultural University, Changchun, China.

^*^ Corresponding author: Dongsheng Che Email:chedongsheng@jlau.edu.cn; Hailong Jiang Email:hljiang@jlau.edu.cn

^†^ These authors contributed equally to this work.

**Table S1** In vitro ileal digestibility of dry matter and gross energy of different feedstuffs

| Items | Corn | Wheat | Barley | Corn husk | Wheat bran | Soybean  husk | Soybean  meal | Rapeseed meal | Sugar beet pulp | Corn germ meal | Corn gluten meal | Corn  DDGS | Alfafa meal | SEM | *P* value |
| --- | --- | --- | --- | --- | --- | --- | --- | --- | --- | --- | --- | --- | --- | --- | --- |
| DM, % | 88.46^a^ | 85.91^b^ | 80.84^c^ | 51.96^i^ | 70.89^f^ | 57.70^h^ | 81.50^c^ | 73.72^e^ | 78.30^d^ | 41.70^j^ | 76.65^d^ | 64.79^g^ | 53.44^i^ | 1.60 | < 0.001 |
| GE, % | 82.52^a^ | 78.49^b^ | 70.30^d^ | 44.74^i^ | 54.63^h^ | 55.37^gh^ | 75.60^c^ | 56.51^gh^ | 67.27^e^ | 56.99^g^ | 71.47^d^ | 62.42^f^ | 24.46^j^ | 1.71 | < 0.001 |

*Corn DDGS* Corn distillers dried grains with solubles, *DM* Dry matter, *GE* Gross energy.

Data were expressed by mean value and standard error of mean, *n* = 6 for each feedstuff treatment group. Mean values within a row with different superscript letters were significantly different (*P* < 0.05).

**Table S2** Effects of dietary fiber structure and apparent viscosity on α-diversity of the fecal microbial community

| Items | Viscosty | | β-glucan-to-arabinoxylan ratios | | | Treatments | | | | | | SEM | *P*-value | | | | |
| --- | --- | --- | --- | --- | --- | --- | --- | --- | --- | --- | --- | --- | --- | --- | --- | --- | --- |
|  | L_V_ | H_V_ | L_β/AX_ | M_β/AX_ | H_β/AX_ | L_V_L_β/AX_ | L_V_M_β/AX_ | L_V_H_β/AX_ | H_V_L_β/AX_ | H_V_M_β/AX_ | H_V_H_β/AX_ |  | V | β/AX | V × β/AX | Linear | Quadratic |
| Sobs^1^ | 799.67^n^ | 1051.83^m^ | 798.50^y^ | 931.58^xy^ | 1047.17^x^ | 744.33^b^ | 801.67^b^ | 853.00^b^ | 852.67^b^ | 1061.50^a^ | 1241.33^a^ | 37.40 | < 0.001 | 0.022 | 0.096 | 0.012 | 0.907 |
| Chao^2^ | 856.73^n^ | 1240.75^m^ | 852.66^y^ | 1074.29^xy^ | 1219.26^x^ | 797.92^b^ | 860.04^b^ | 912.22^b^ | 907.39^b^ | 1288.55^a^ | 1526.30^a^ | 55.60 | < 0.001 | 0.019 | 0.024 | 0.013 | 0.733 |
| Shannon^3^ | 4.59 | 4.70 | 4.41^y^ | 4.61^xy^ | 4.90^x^ | 4.27^b^ | 4.61^ab^ | 4.88^a^ | 4.55^ab^ | 4.62^ab^ | 4.92^a^ | 0.07 | 0.482 | 0.027 | 0.685 | 0.008 | 0.771 |
| Simpson^4^ | 0.05 | 0.05 | 0.06^x^ | 0.05^xy^ | 0.04^y^ | 0.06 | 0.05 | 0.03 | 0.06 | 0.06 | 0.04 | 0.01 | 0.711 | 0.043 | 0.800 | 0.014 | 0.665 |

*L_V_L_β/AX_* Diet with low viscocity and low β-glucan-to-arabinoxylan ratios, *L_V_M_β/AX_* Diet with low viscocity and mideum β-glucan-to-arabinoxylan ratios, *L_V_H_β/AX_* Diet with low viscocity and high β-glucan-to-arabinoxylan ratios, *H_V_L_β/AX_* Diet with high viscocity and low β-glucan-to-arabinoxylan ratios, *H_V_M_β/AX_* Diet with high viscocity and mideum β-glucan-to-arabinoxylan ratios, *H_V_H_β/AX_* Diet with high viscocity and high β-glucan-to-arabinoxylan ratios, *SEM* Standard error of the mean, *V* Apparent viscosity of diets, *β/AX* β-glucan-to-arabinoxylan ratios of diets, *V × β/AX* Linear interaction effect between apparent viscosity and β-glucan-to-arabinoxylan ratios of diets.

^1^ Sobs represent the actual number of species observed.

^2^ Chao index is an estimate of the total number of species commonly used in ecology to reflect species richness.

^3^ Shannon index, an index to measure species diversity: an increase in the Shannon index value represents an increase in species diversity

^4^ Simpson index, describes the probability that the number of individuals sampled from a community twice in a row belong to the same species

^a-f^ Different letters denote significant differences between experimental treatments within each α-diversity variable (*P* < 0.05).

^m,n^ Different letters denote significant differences between the low and high viscosity groups within each α-diversity variable (*P* < 0.05).

^x,y,z^ Different letters denote significant differences between the low, middle and high β-glucan-to-arabinoxylan ratios groups within each α-diversity variable (*P* < 0.05).

A


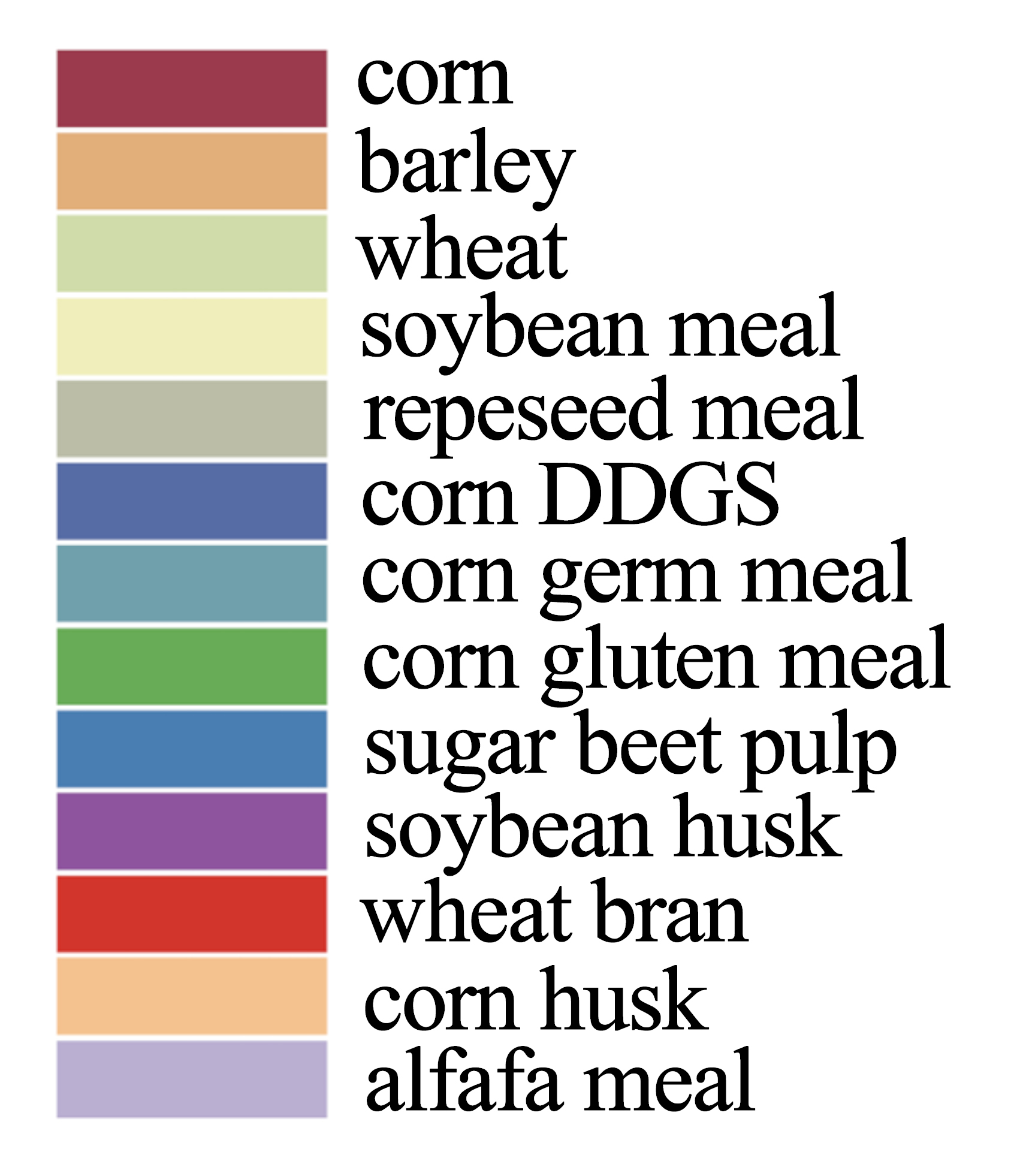


B


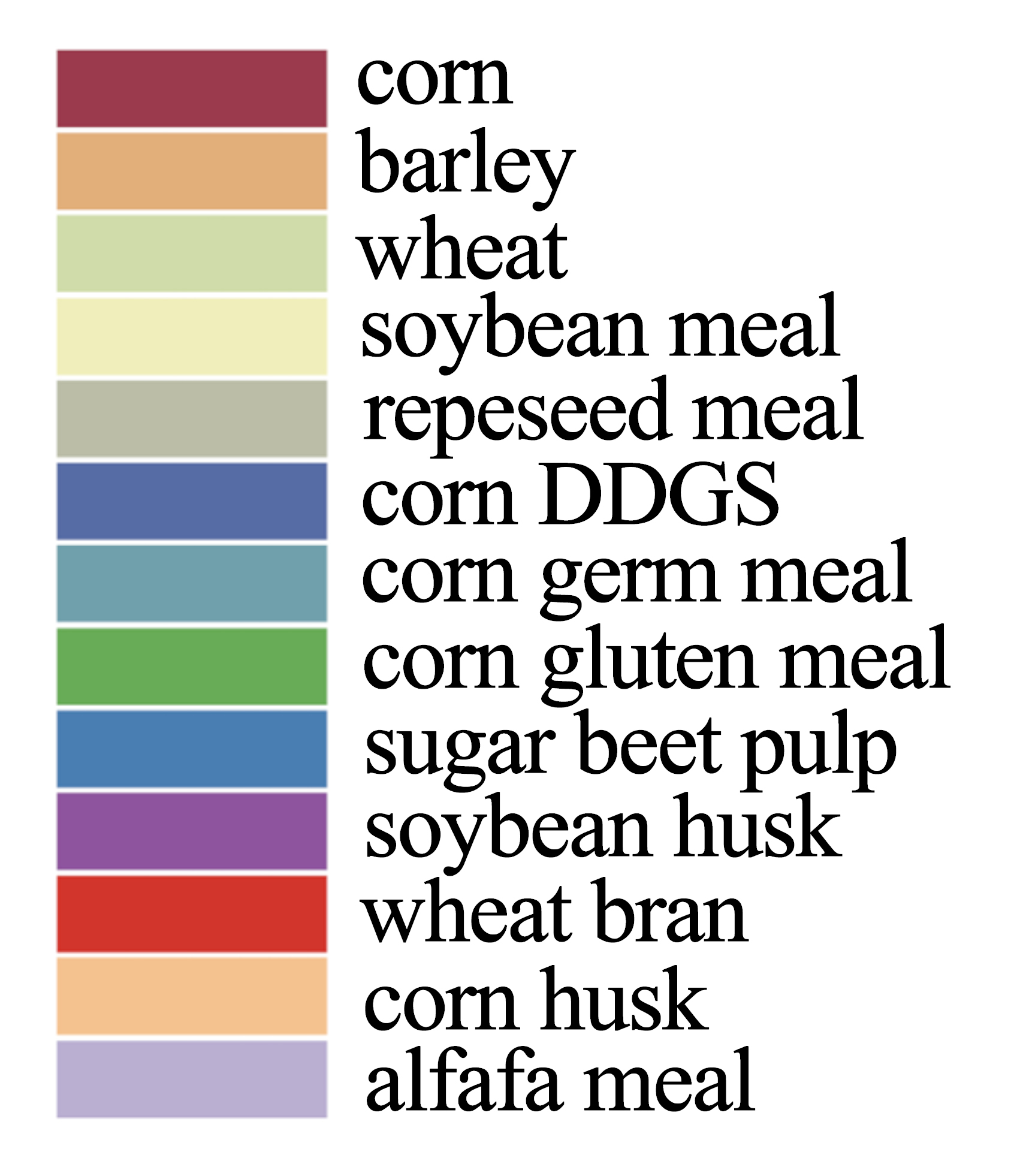


C


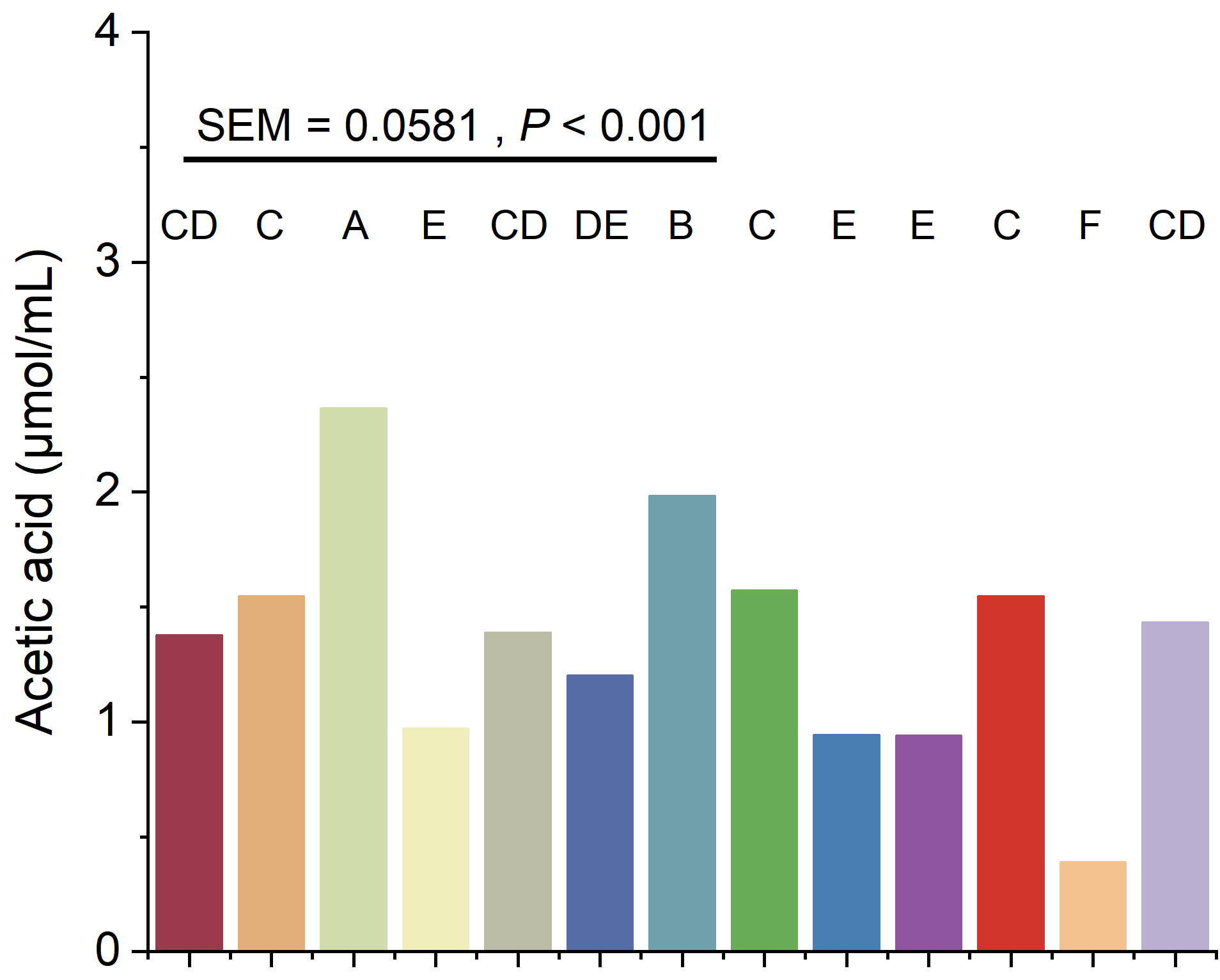

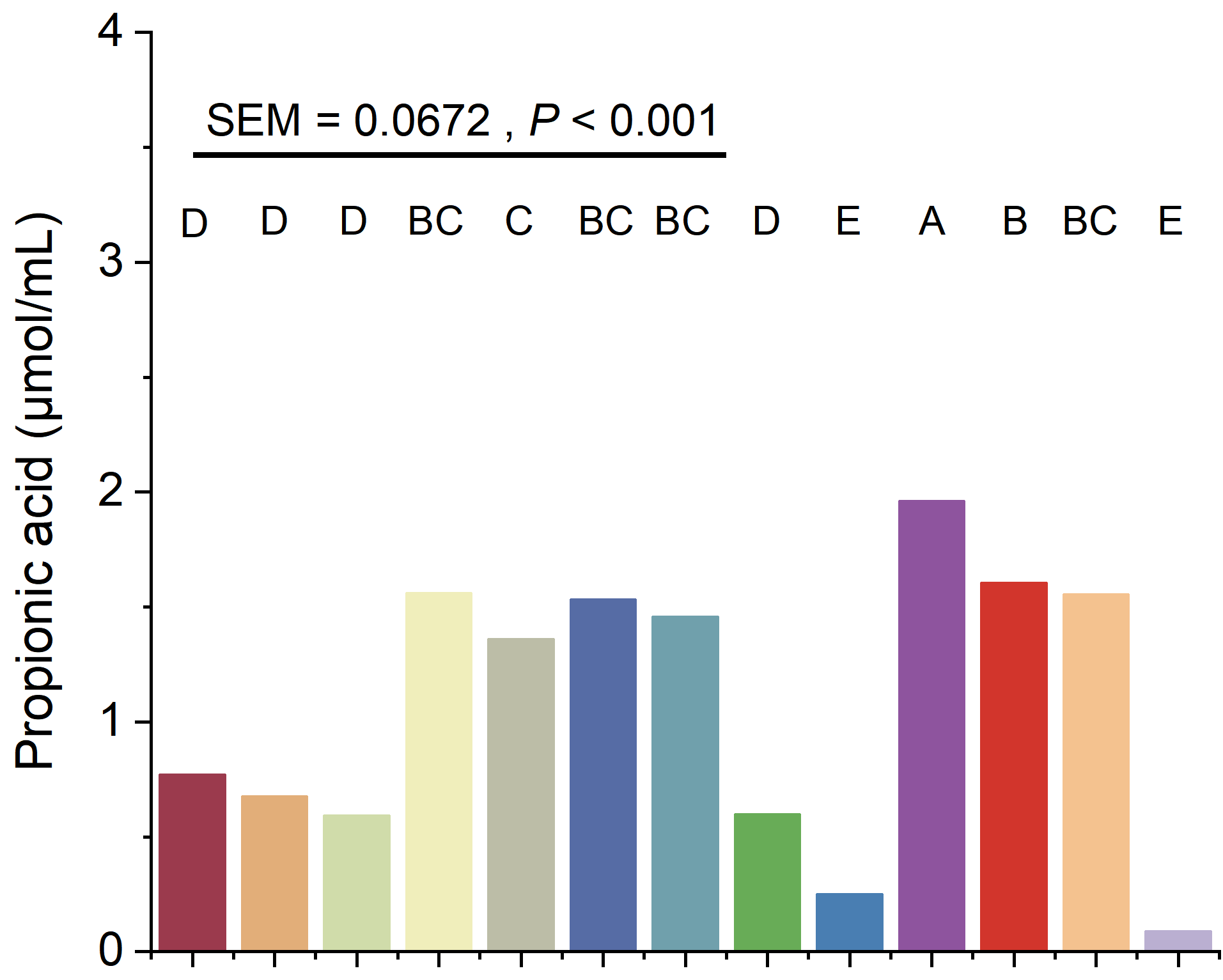

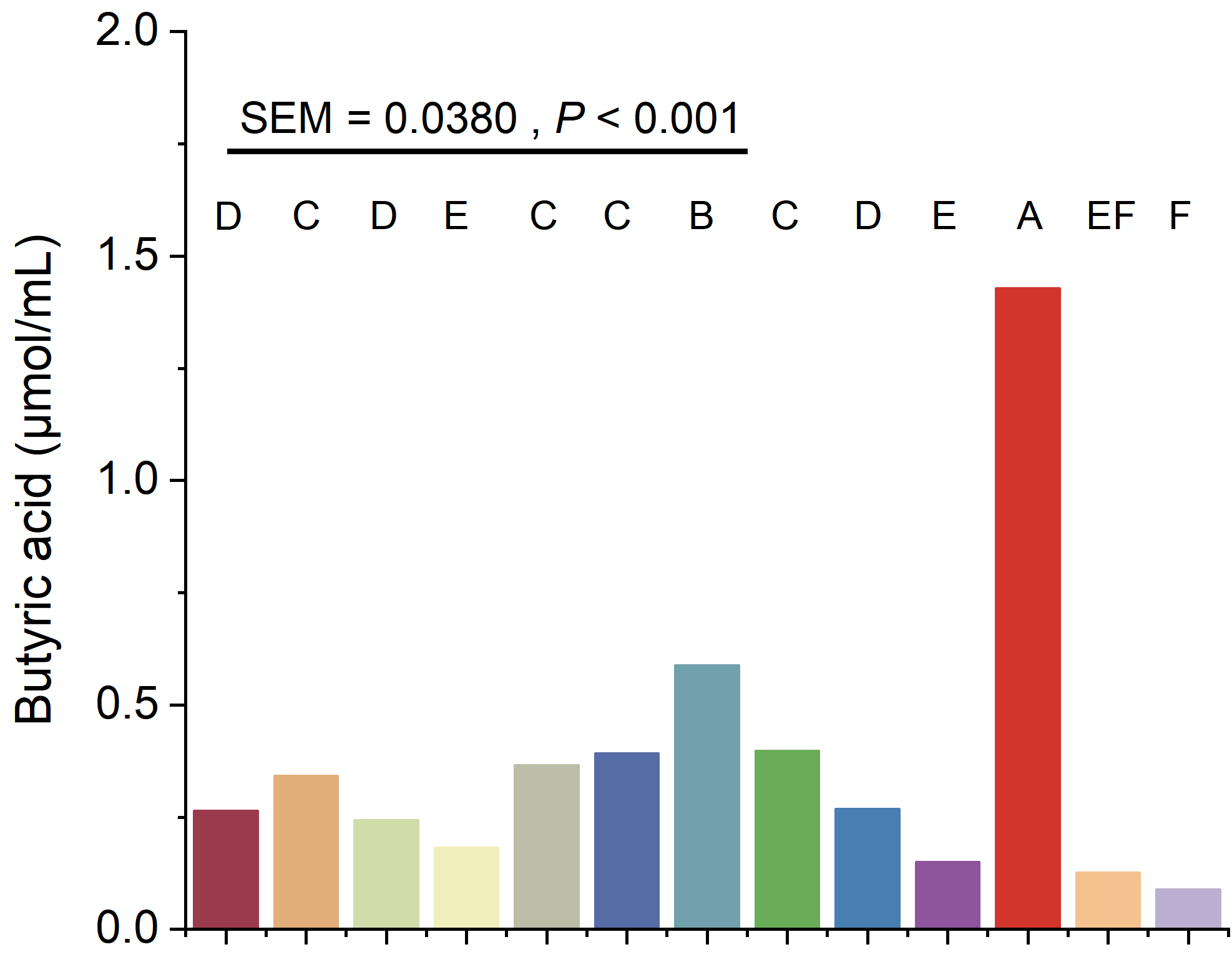


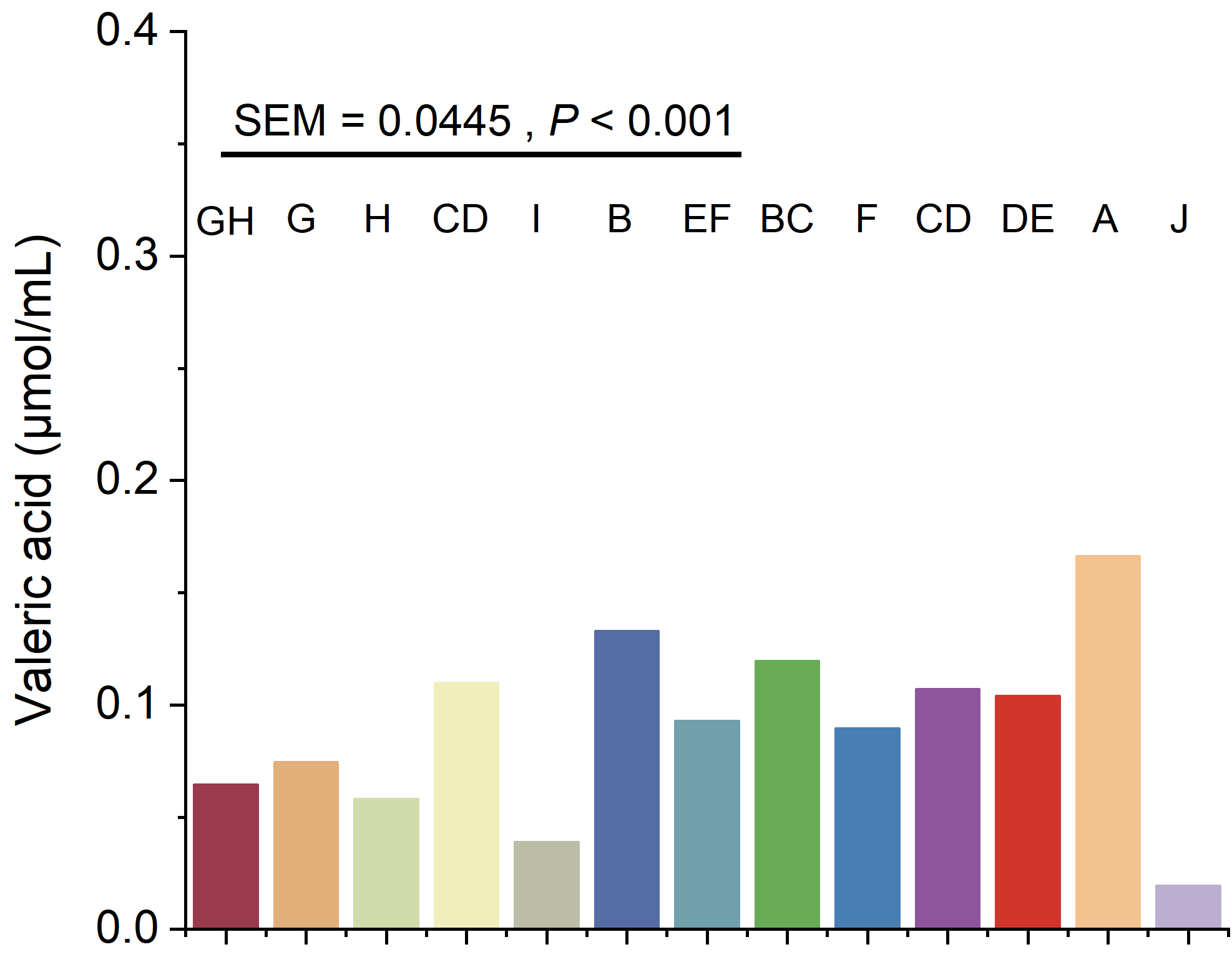

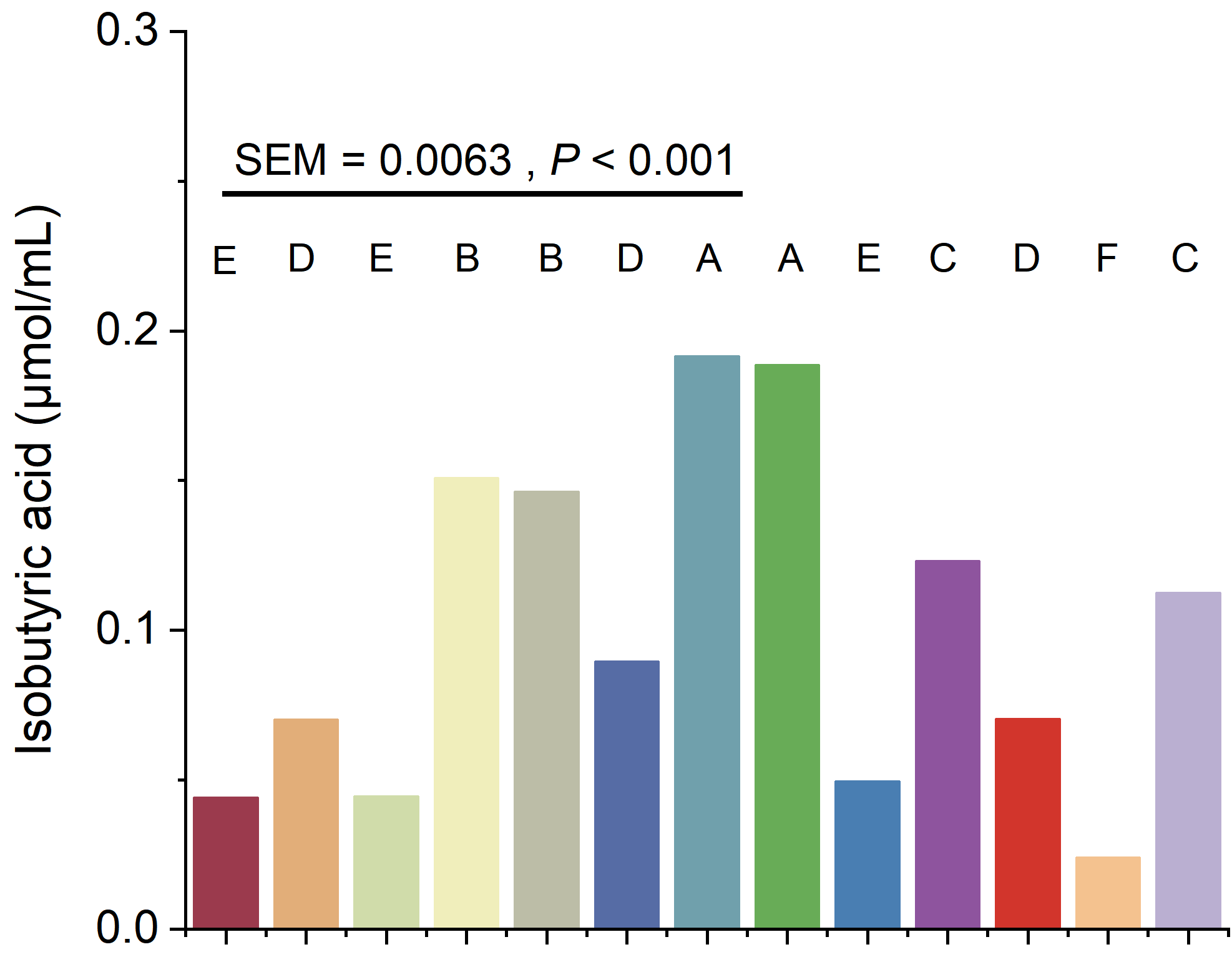

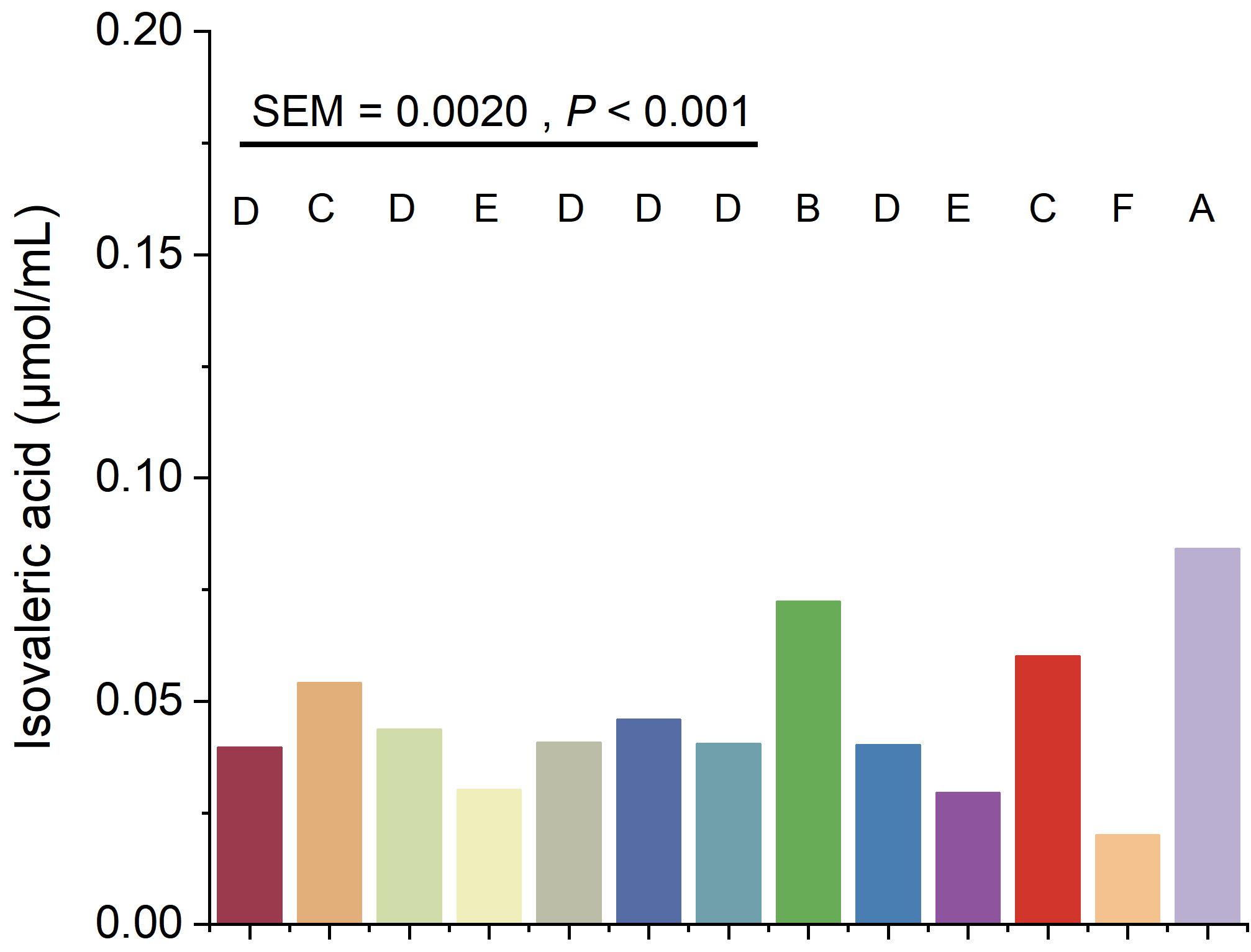


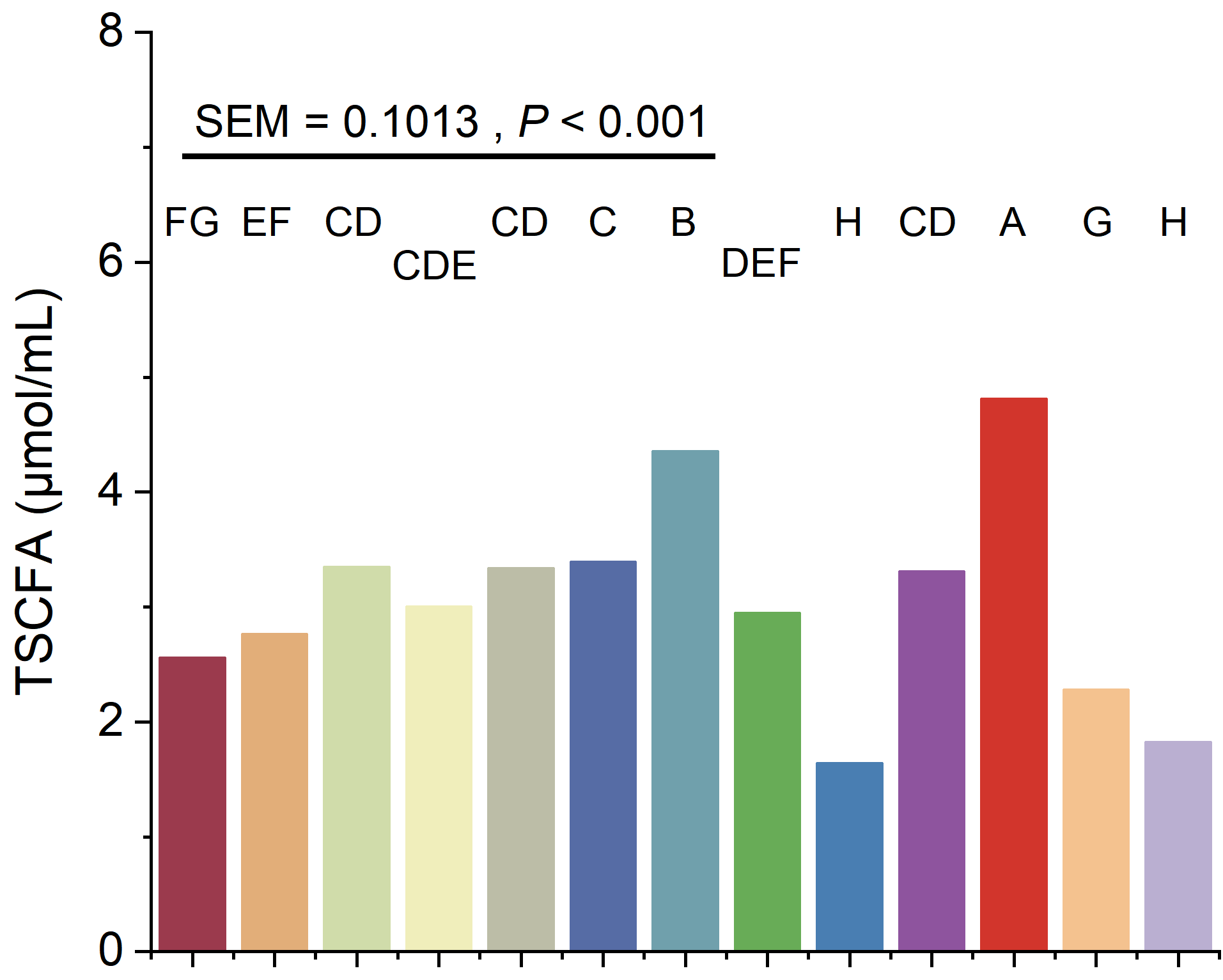

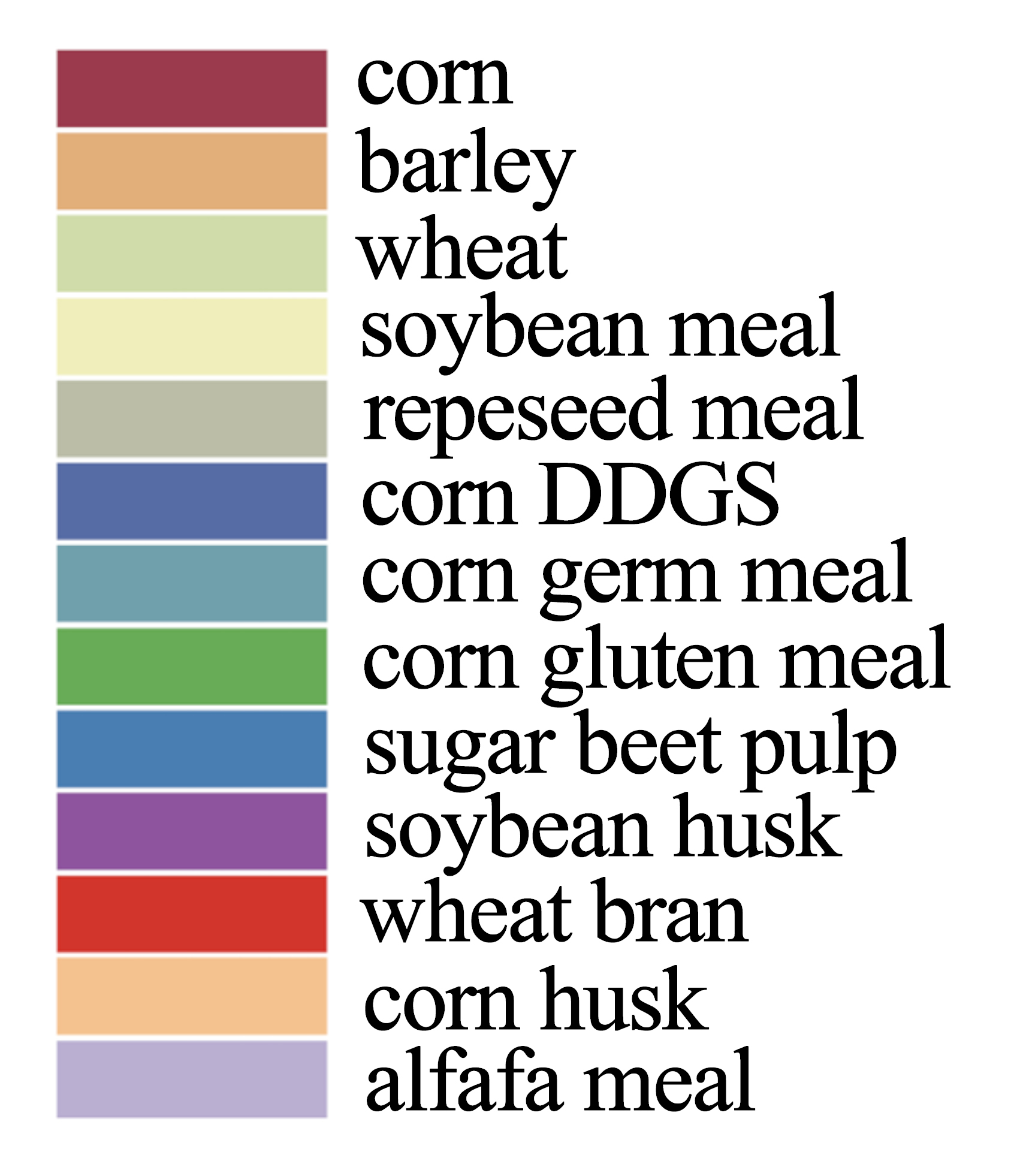


D


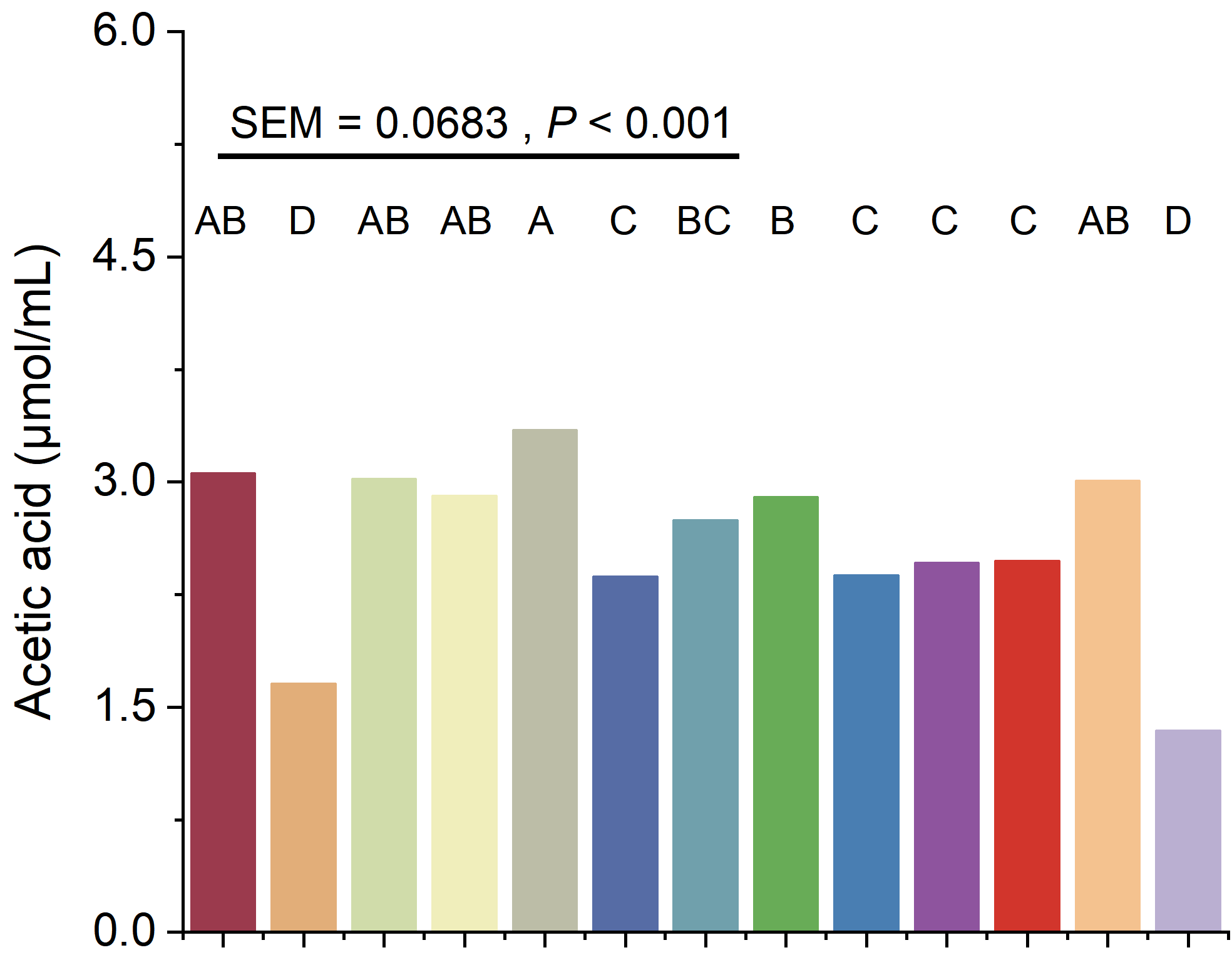

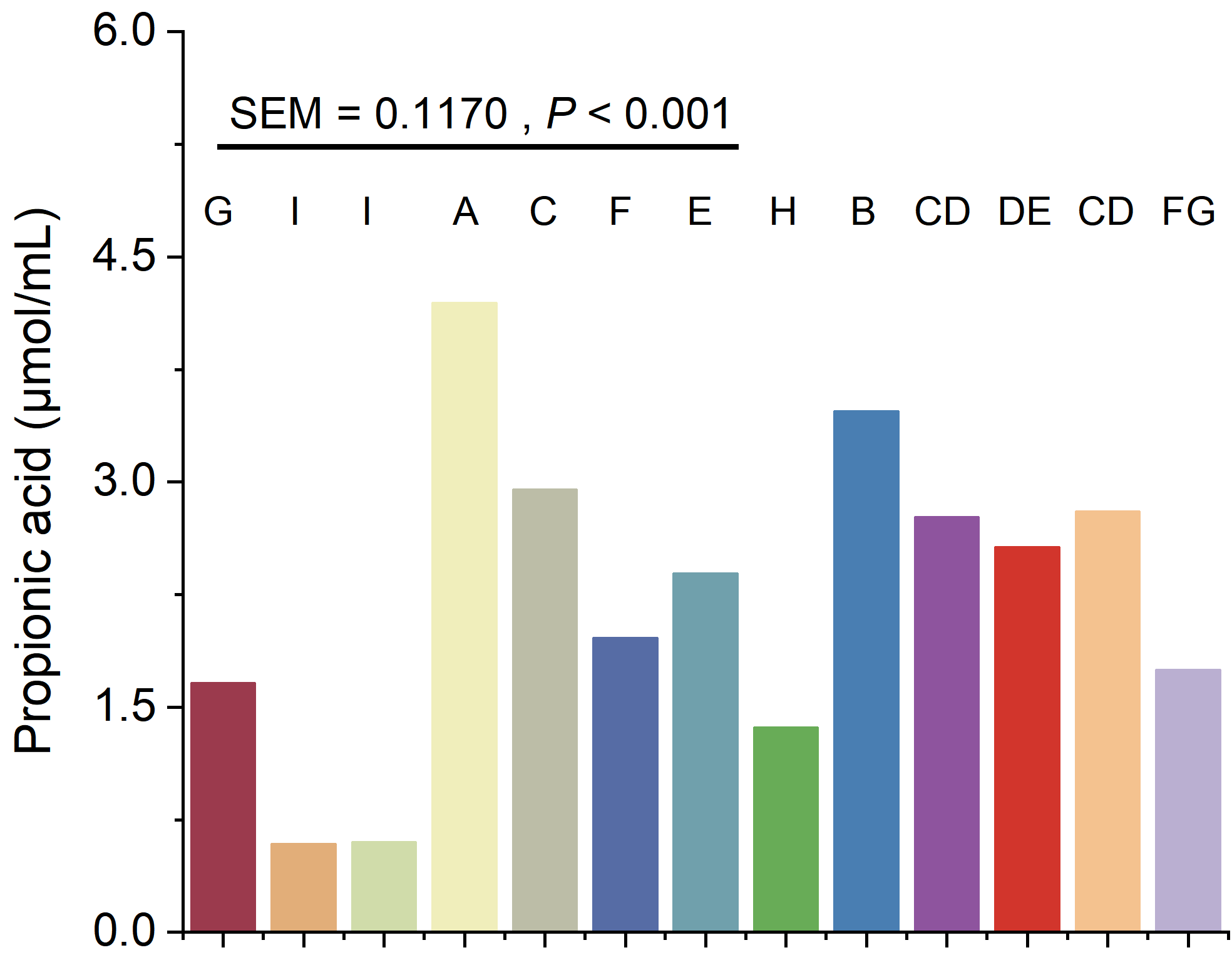

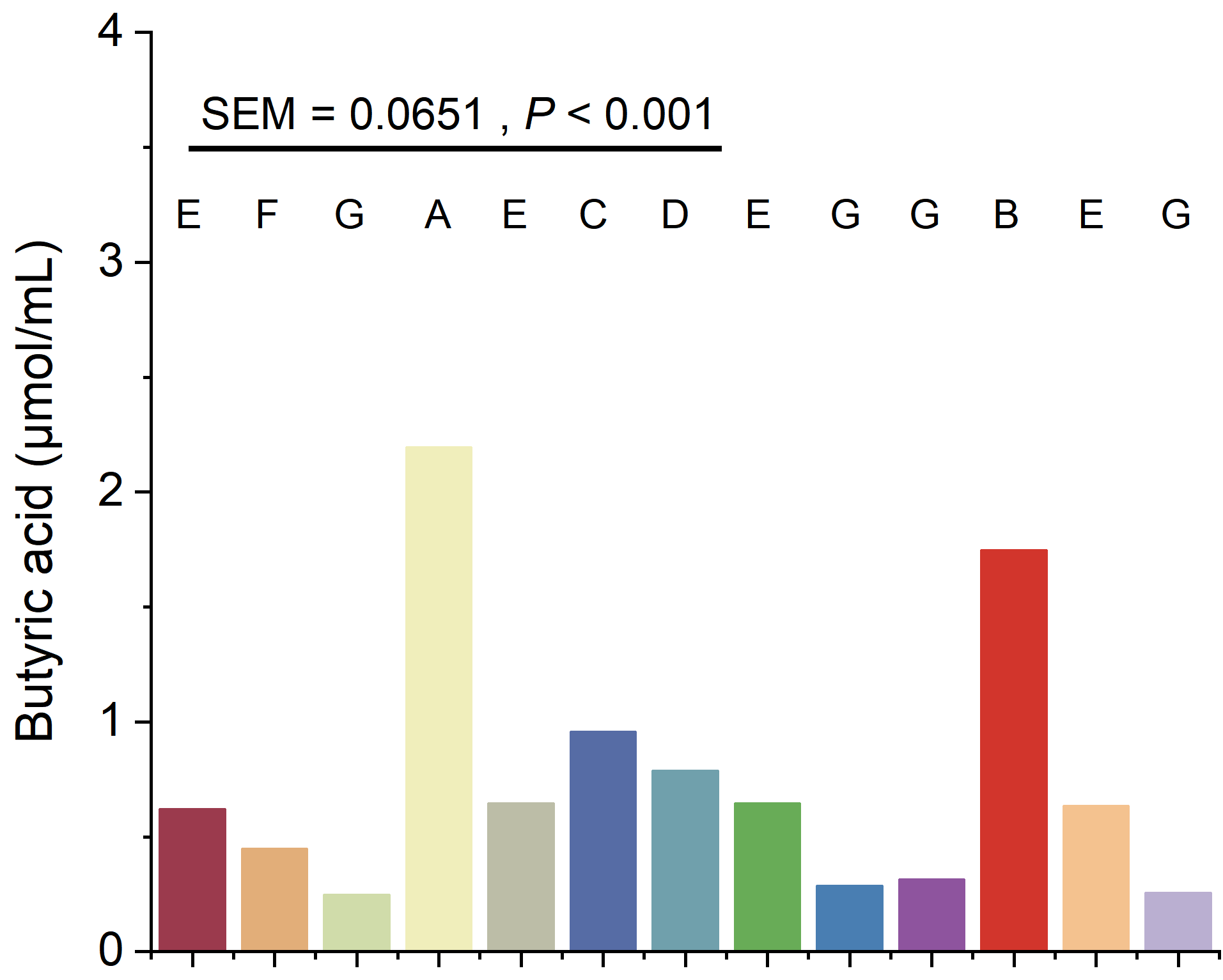


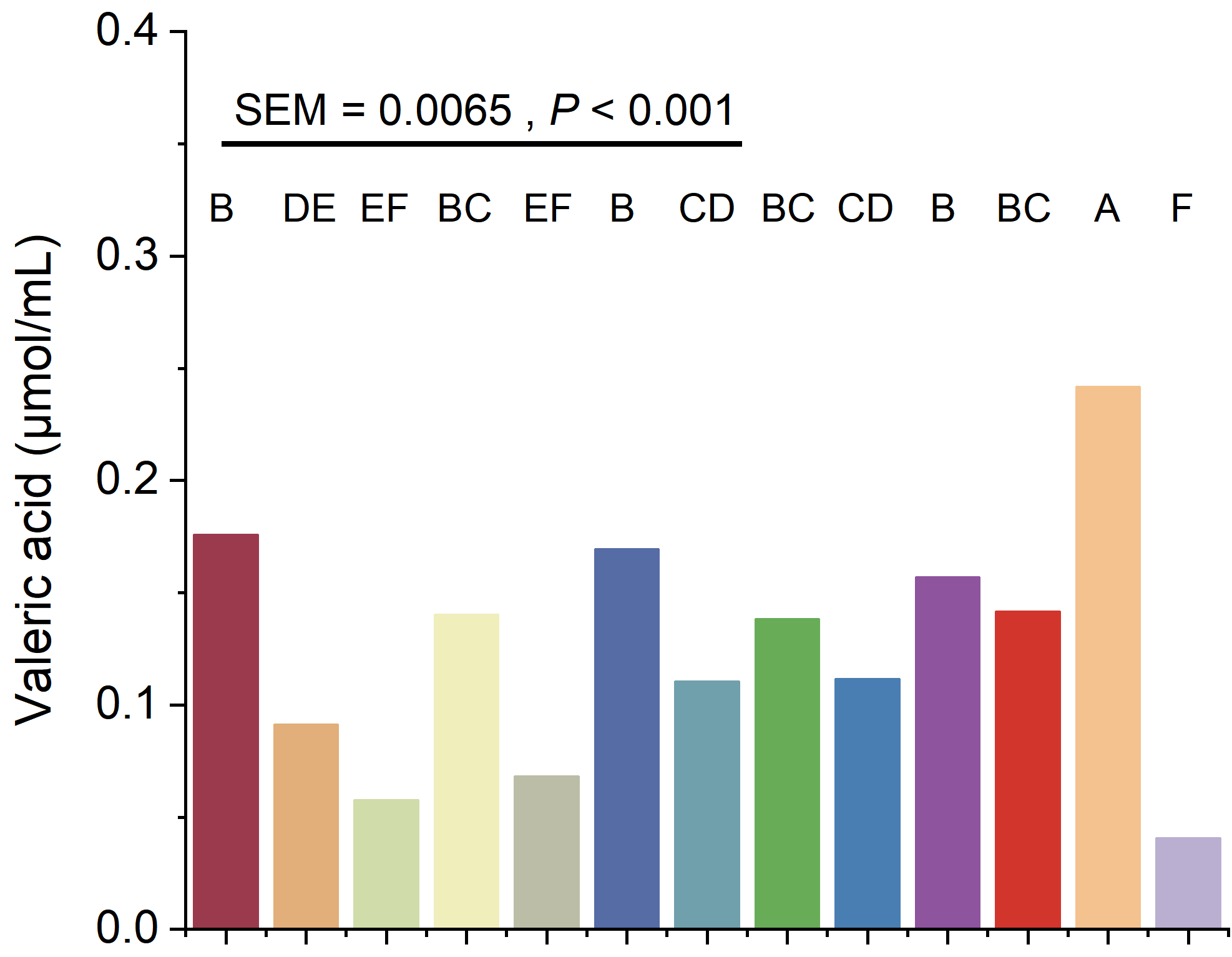

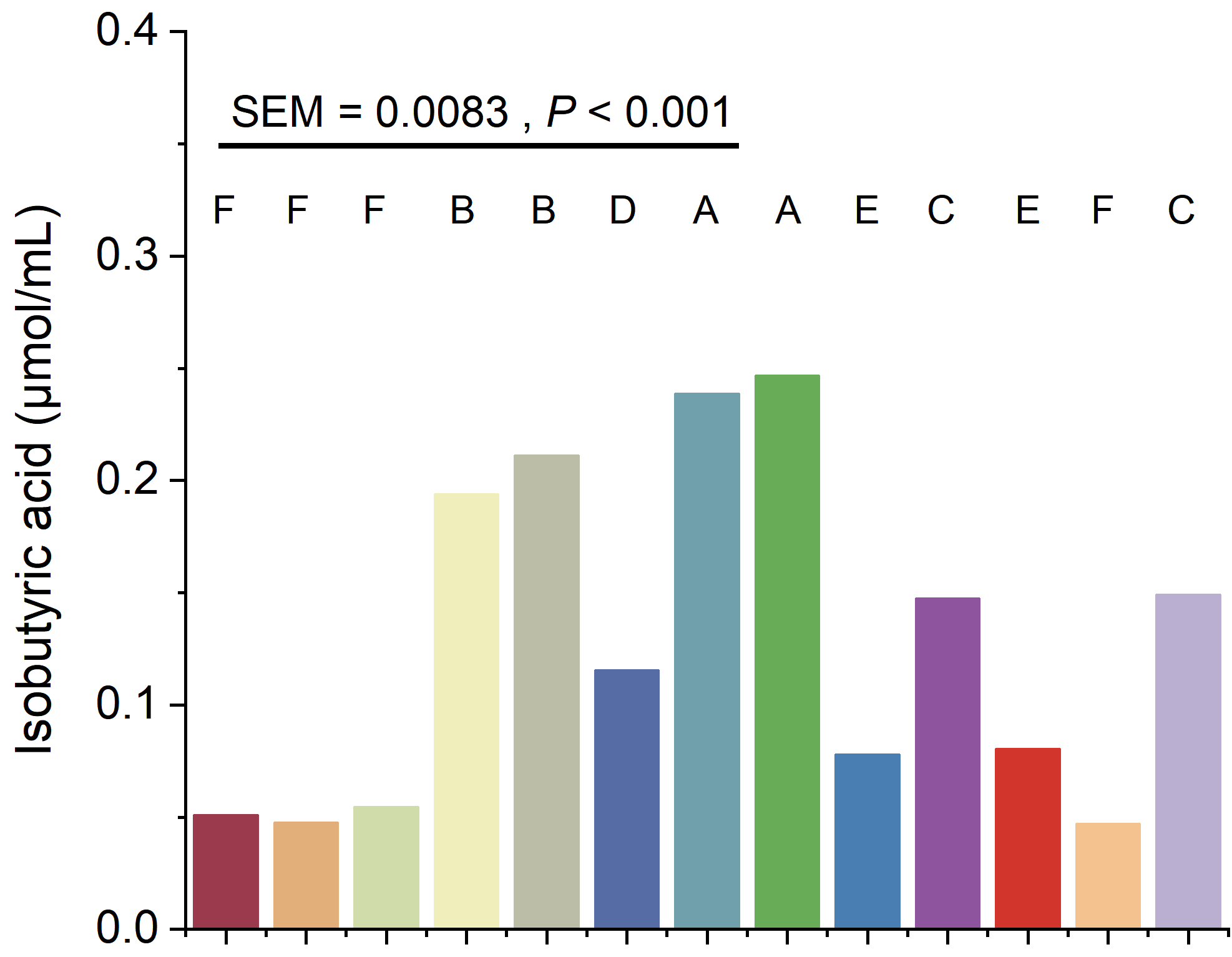

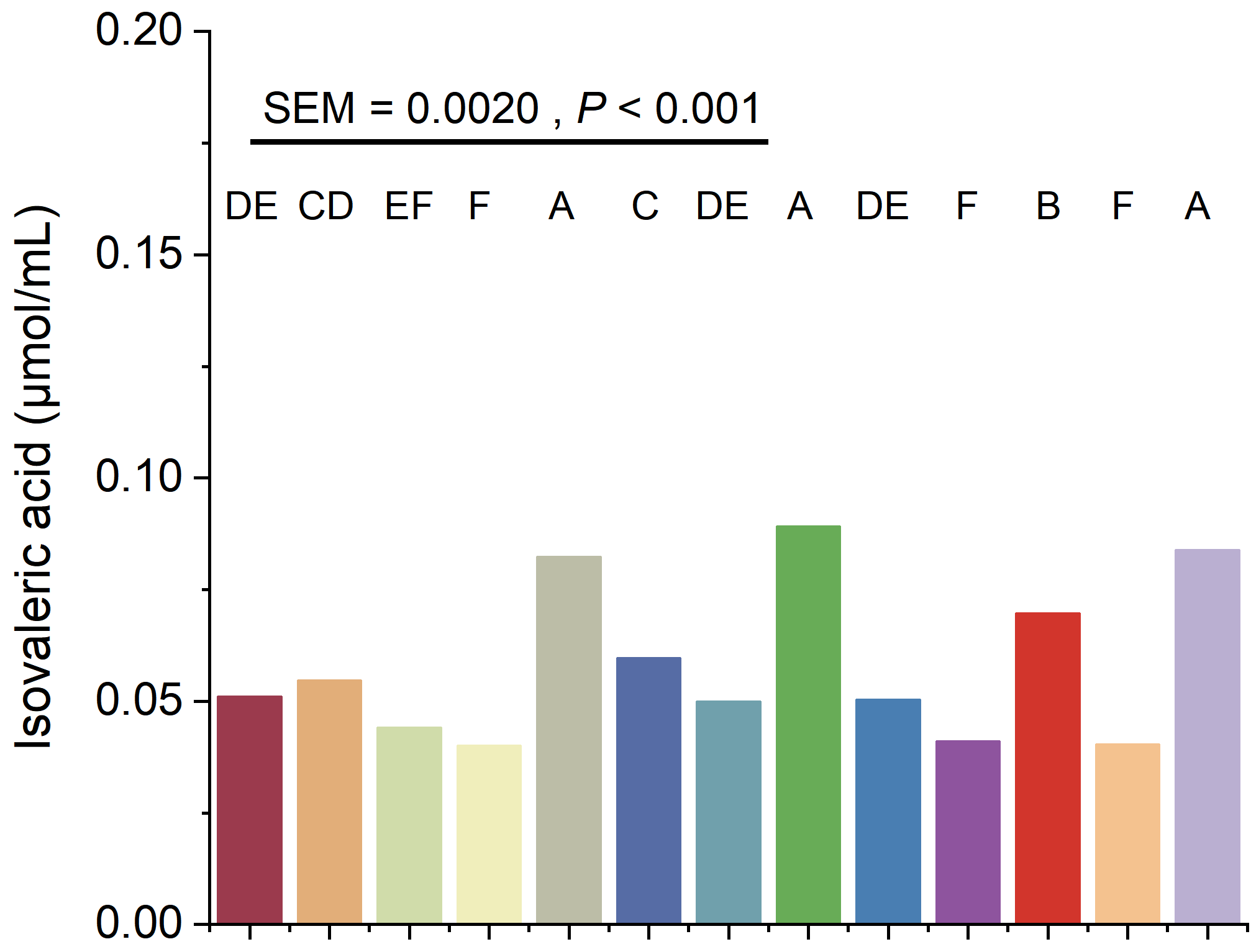


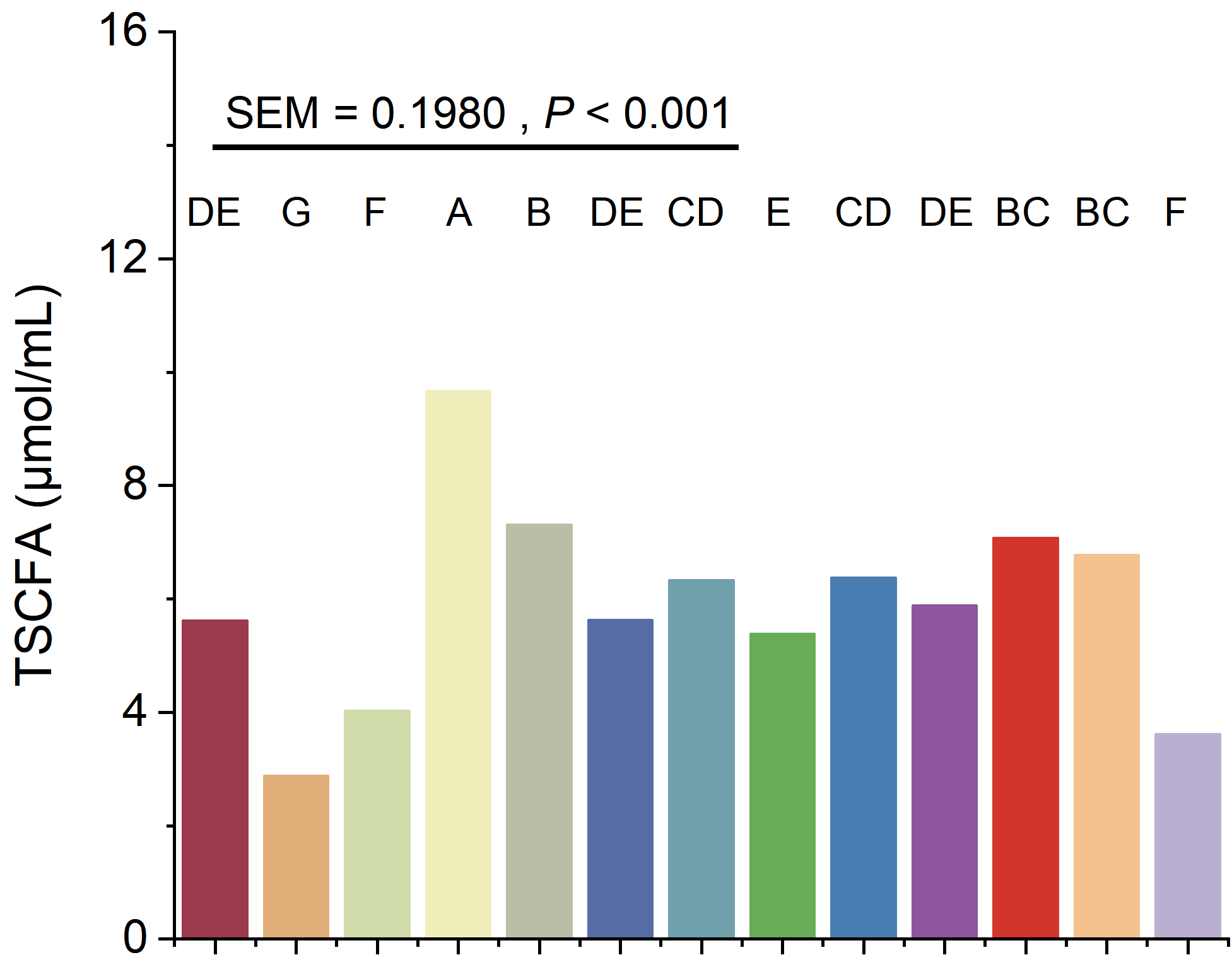

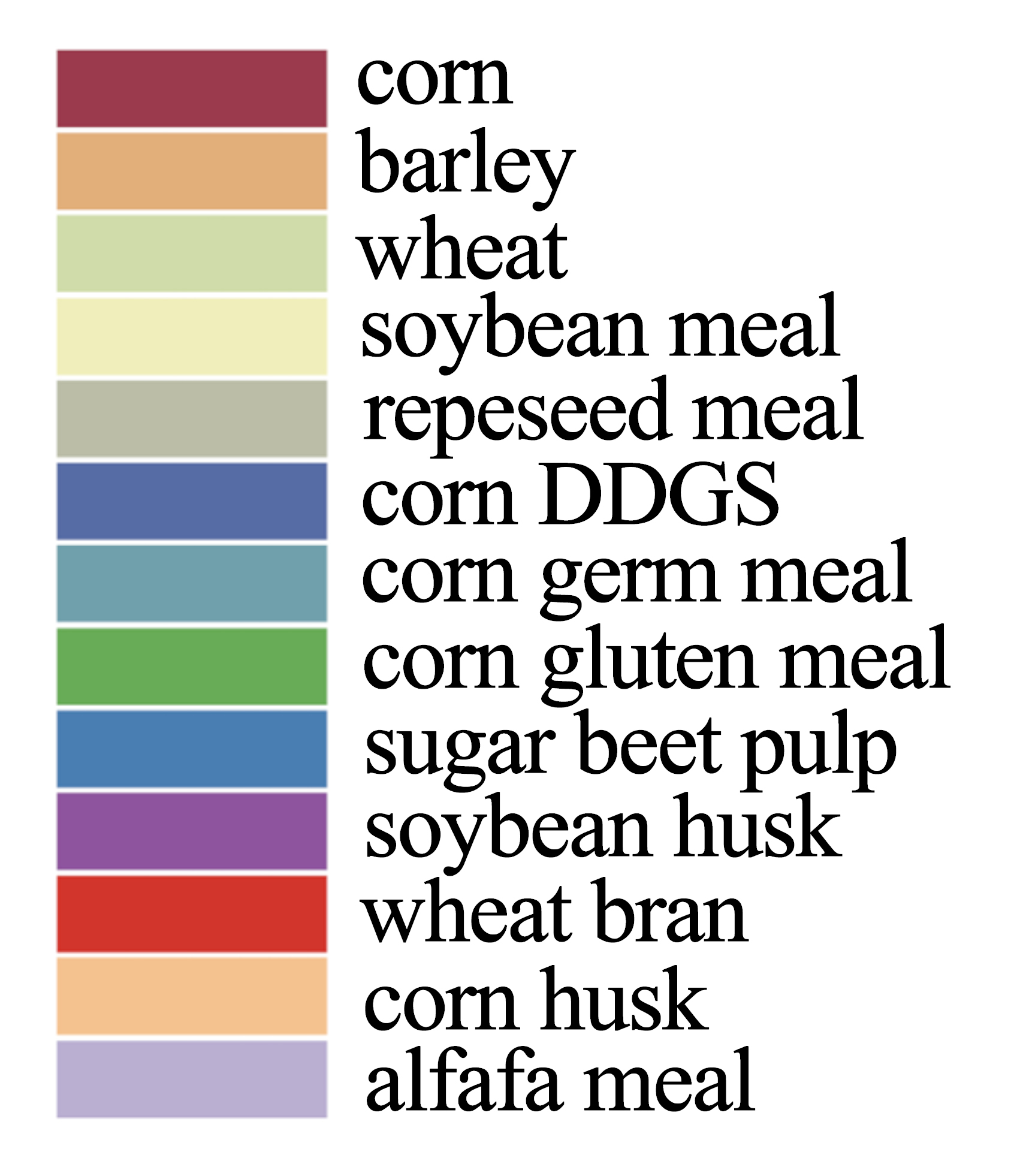


E


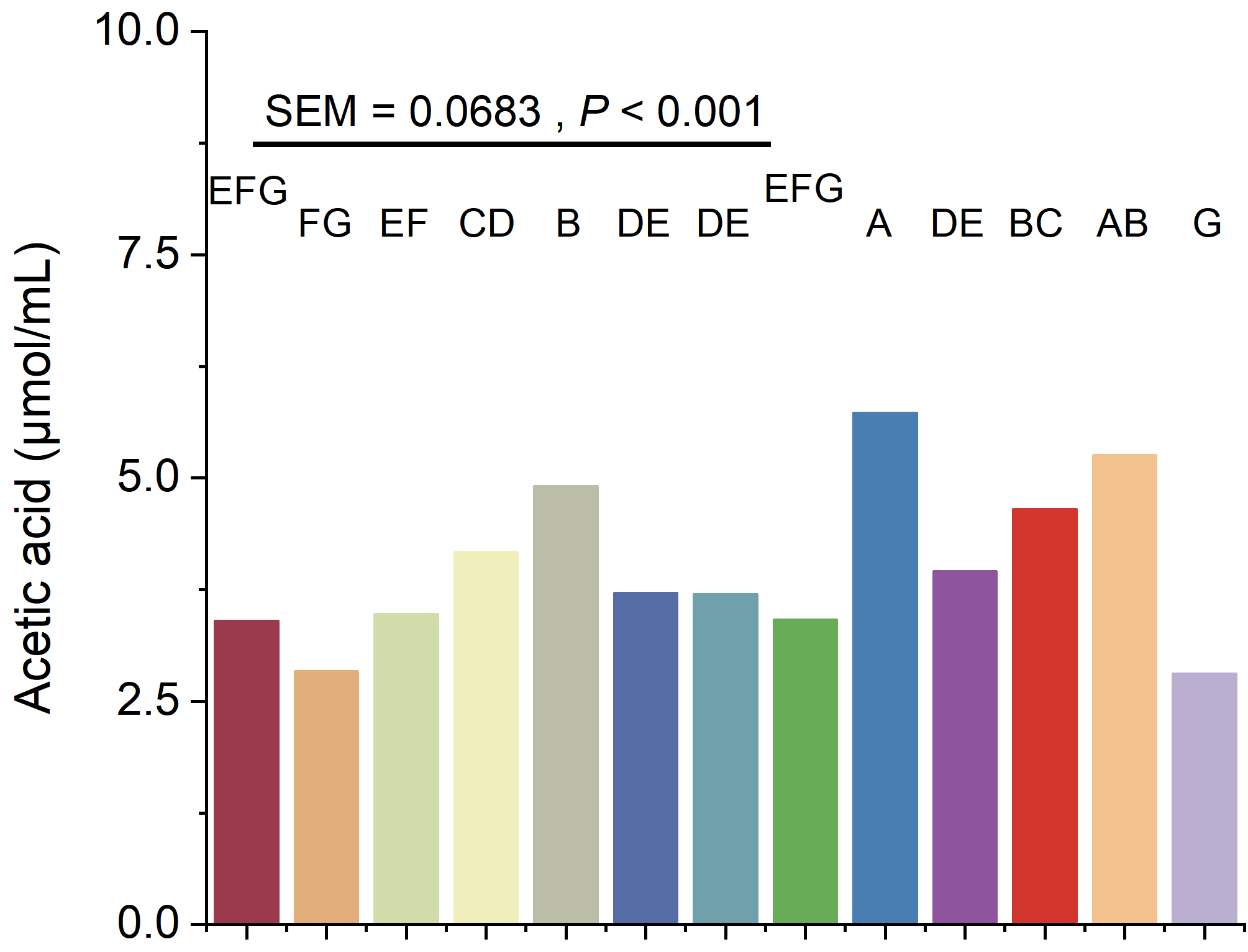

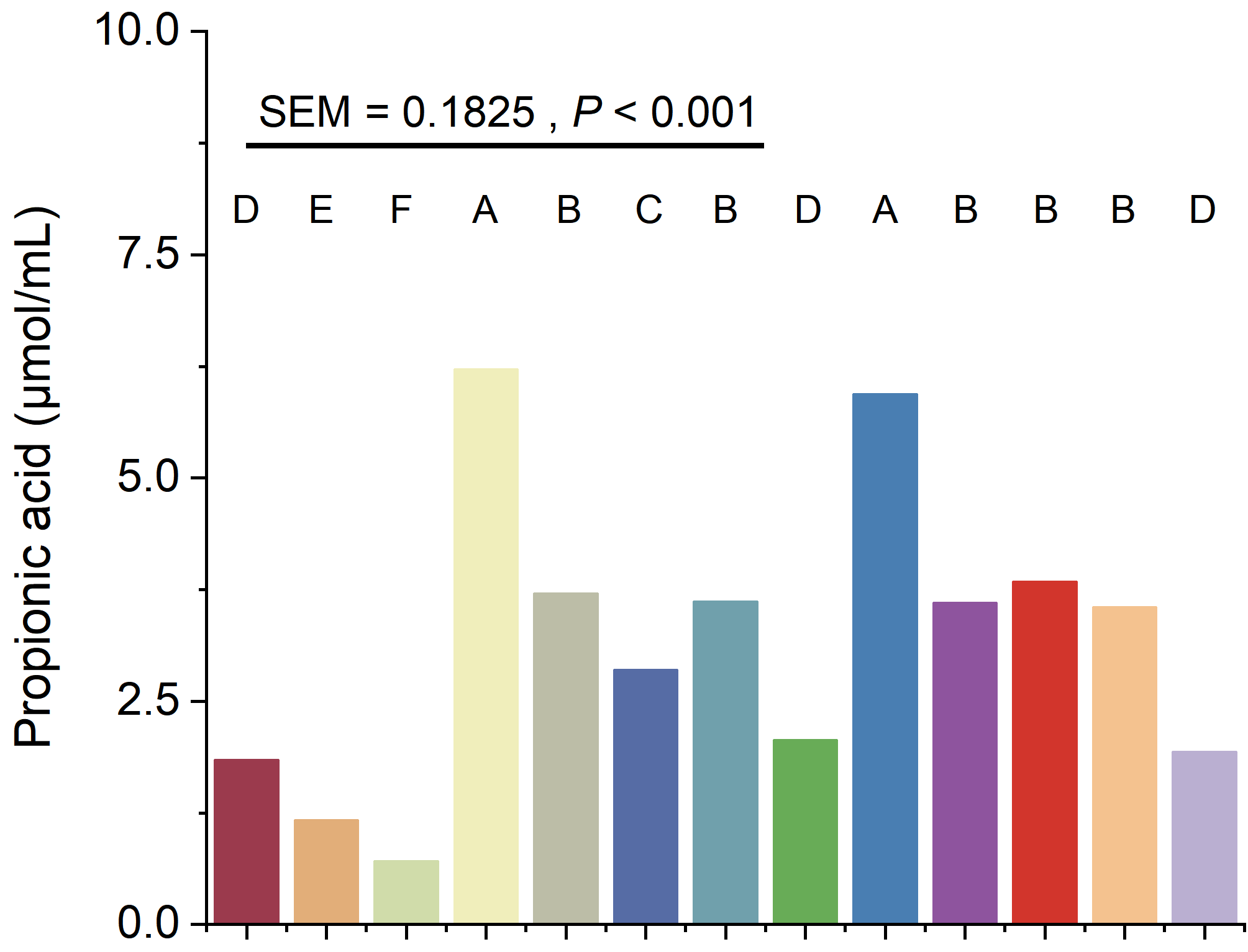

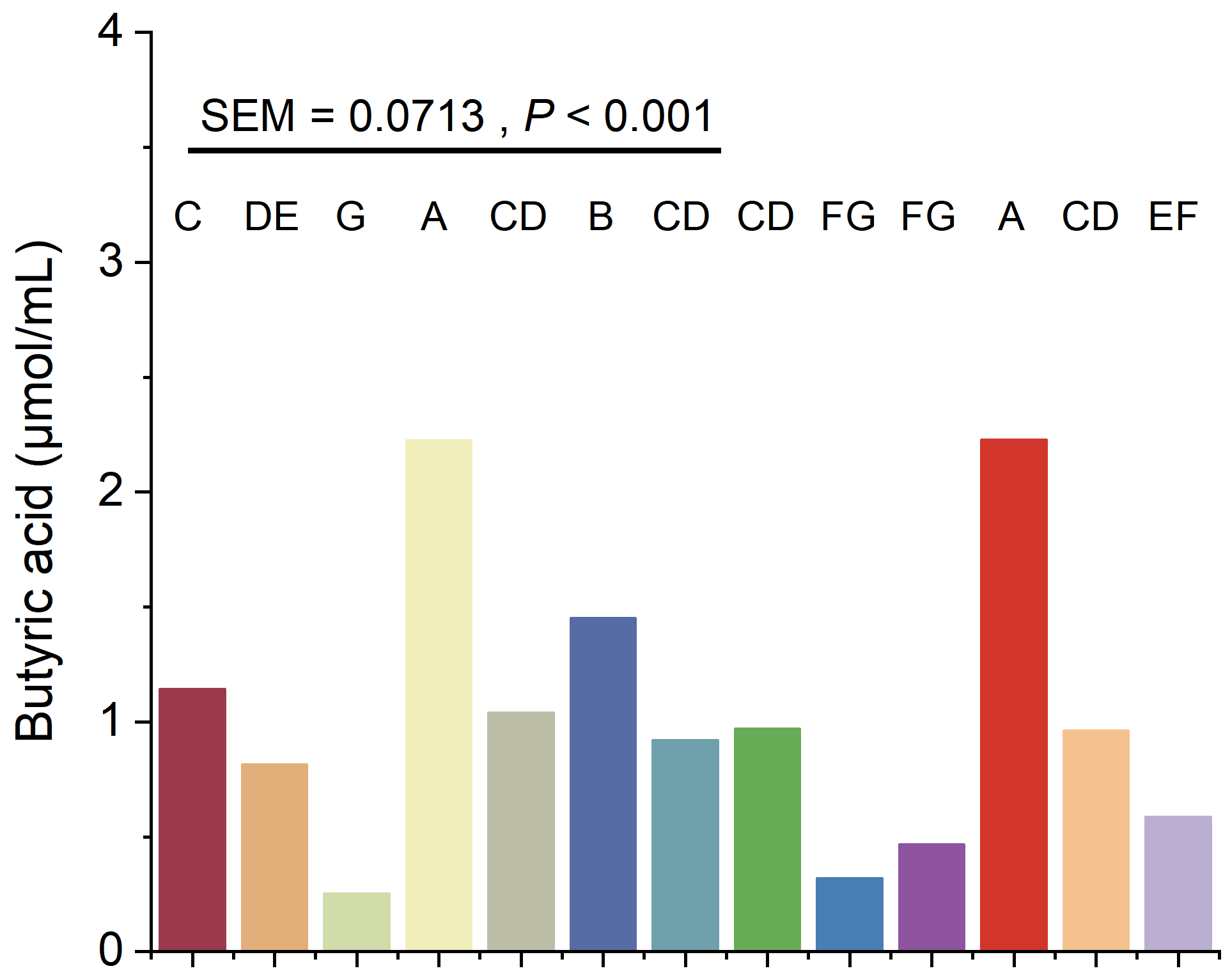


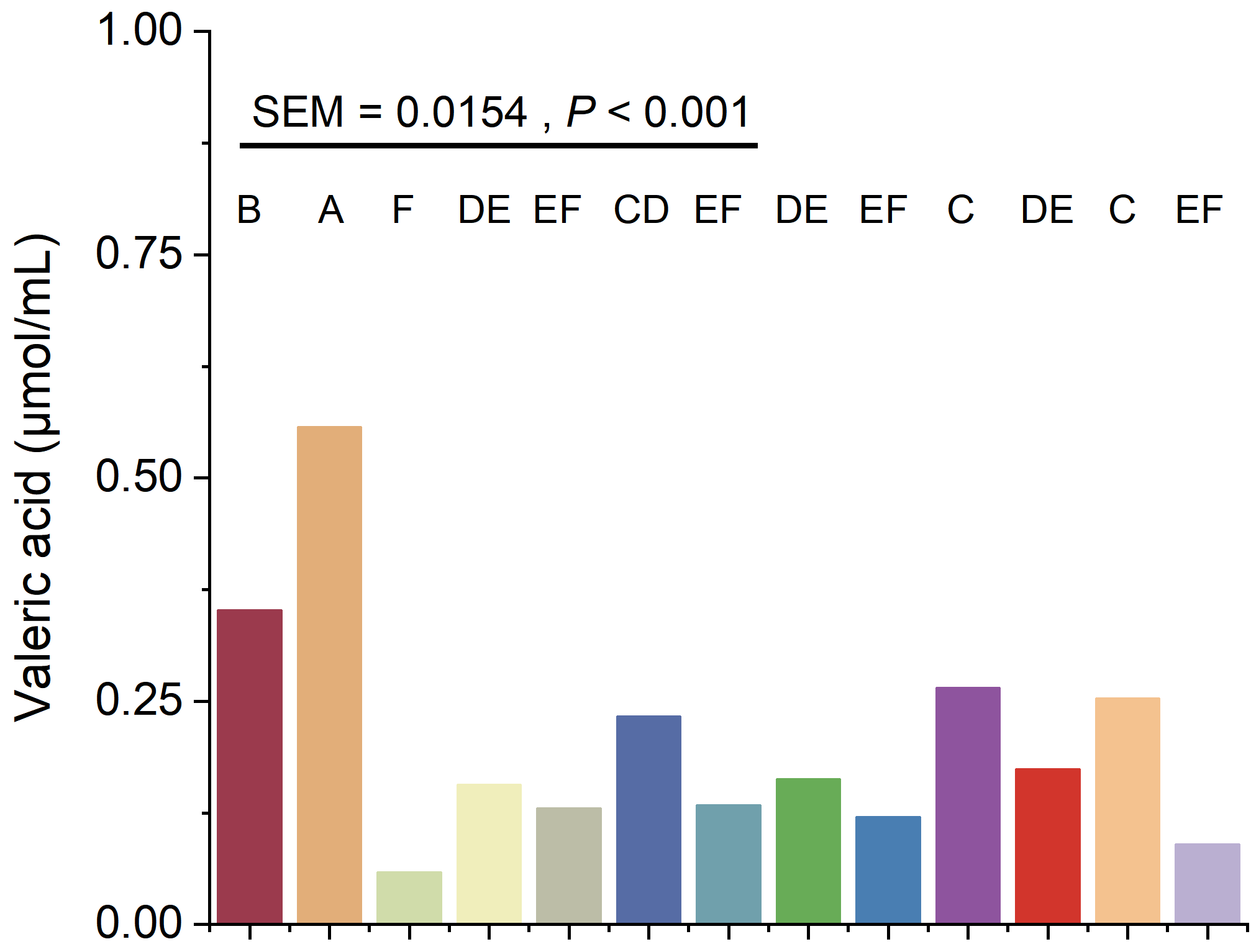

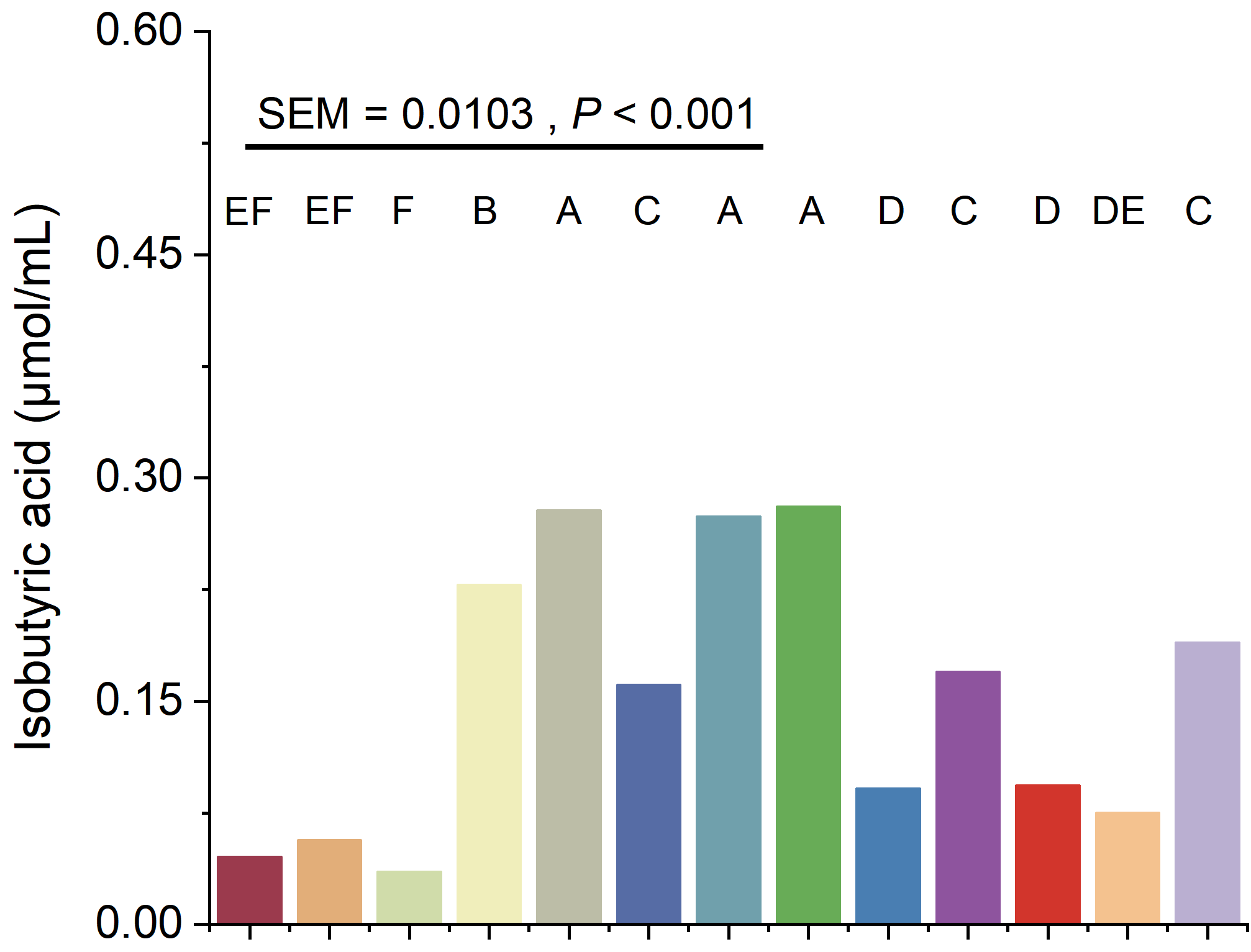

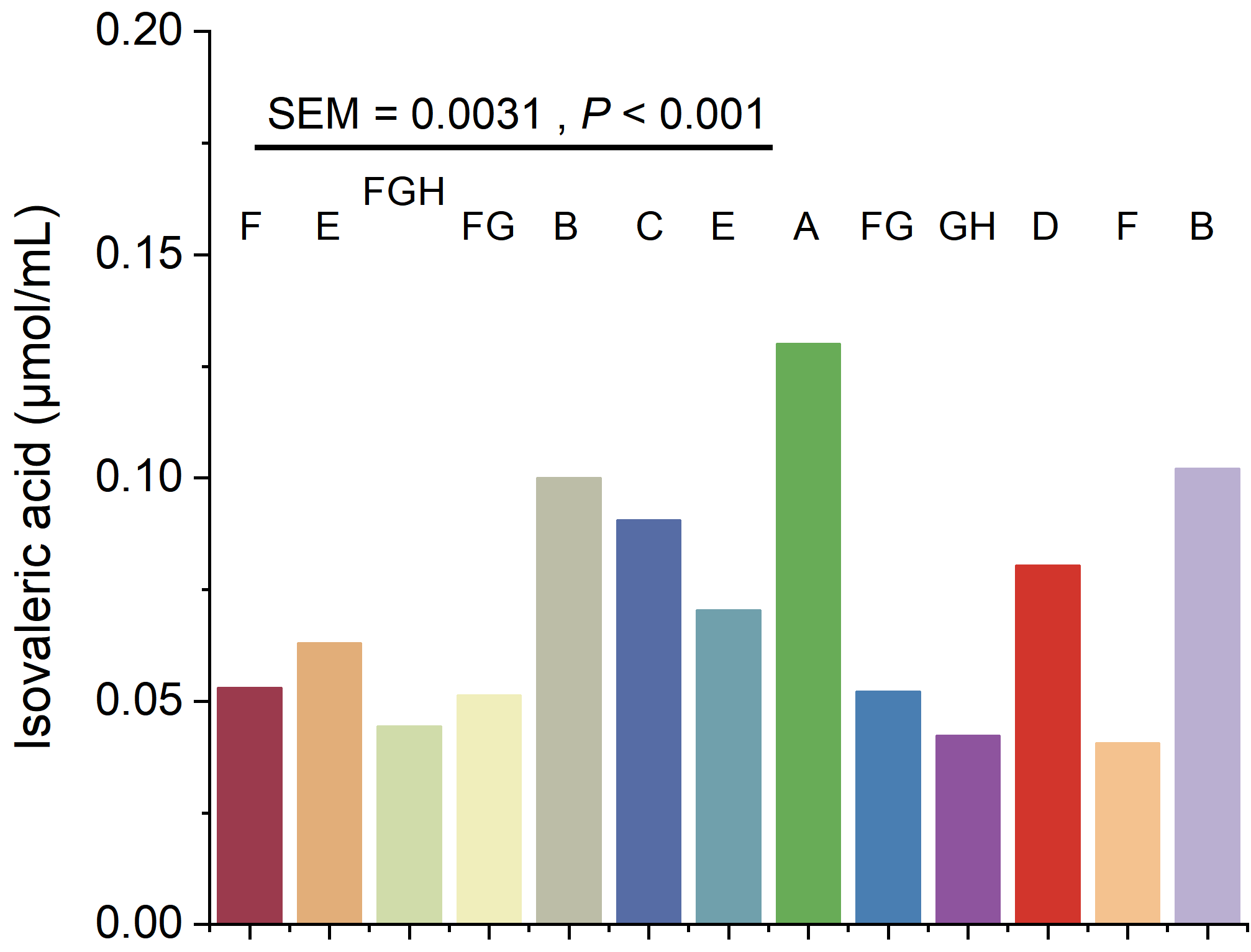


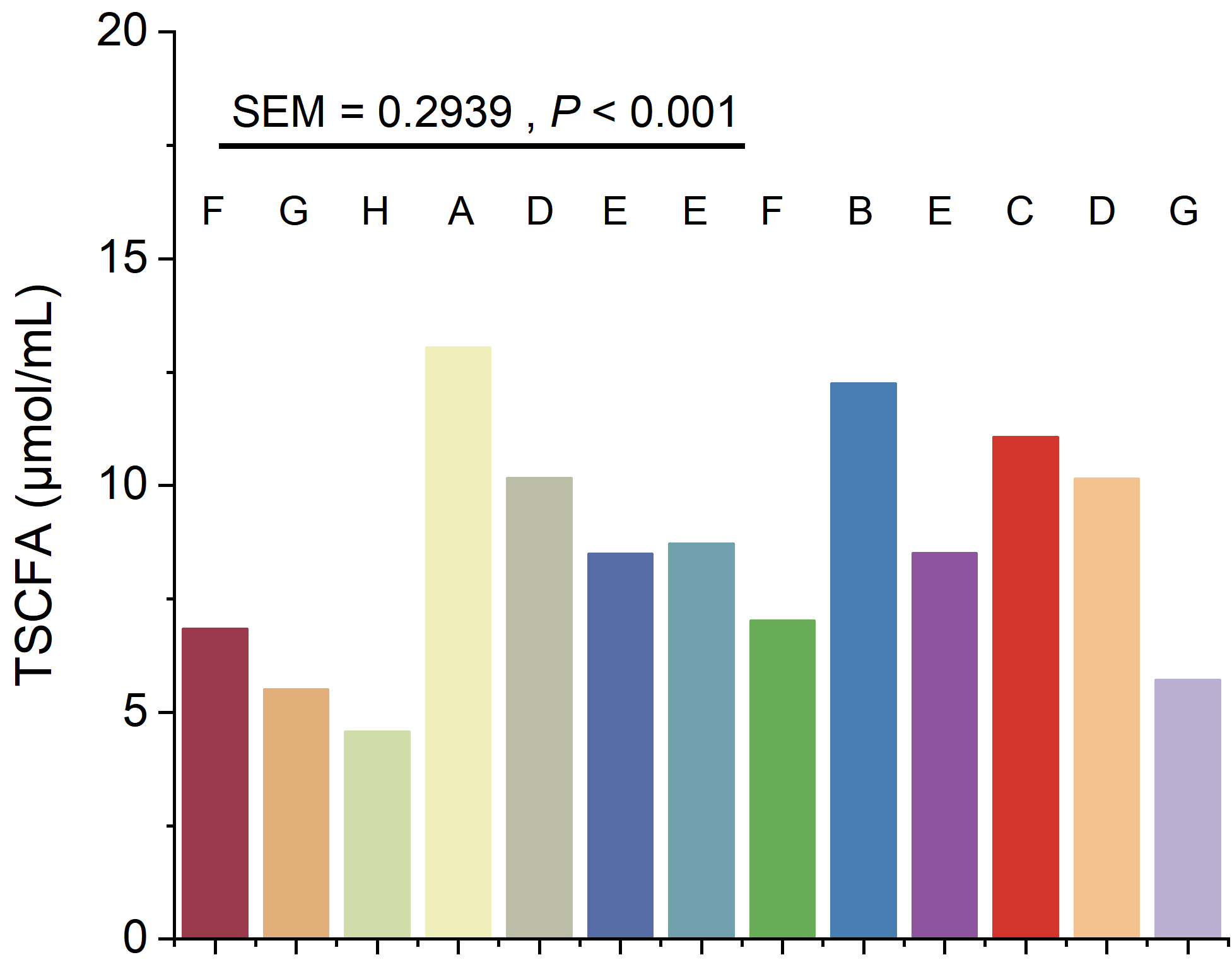

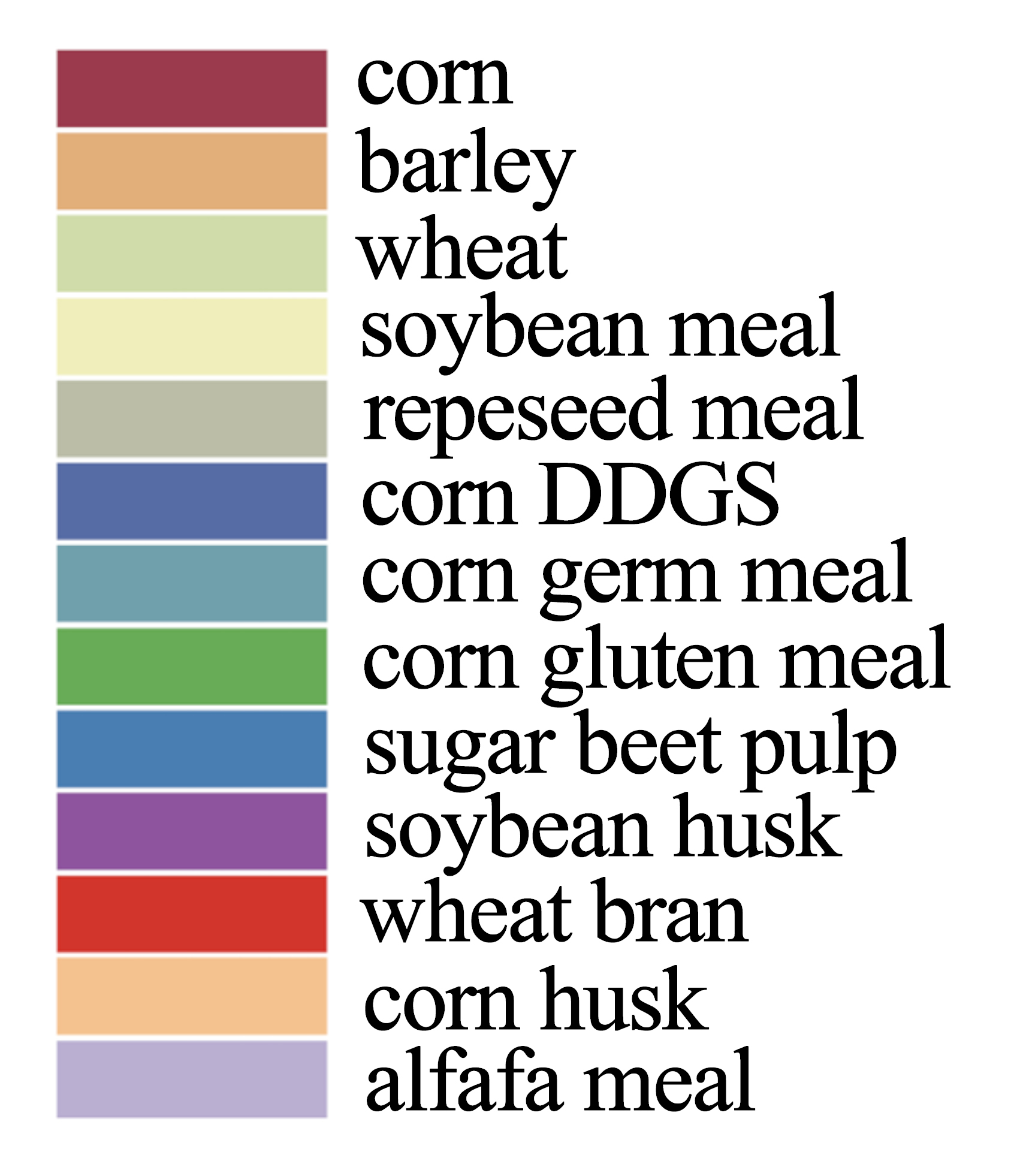


F


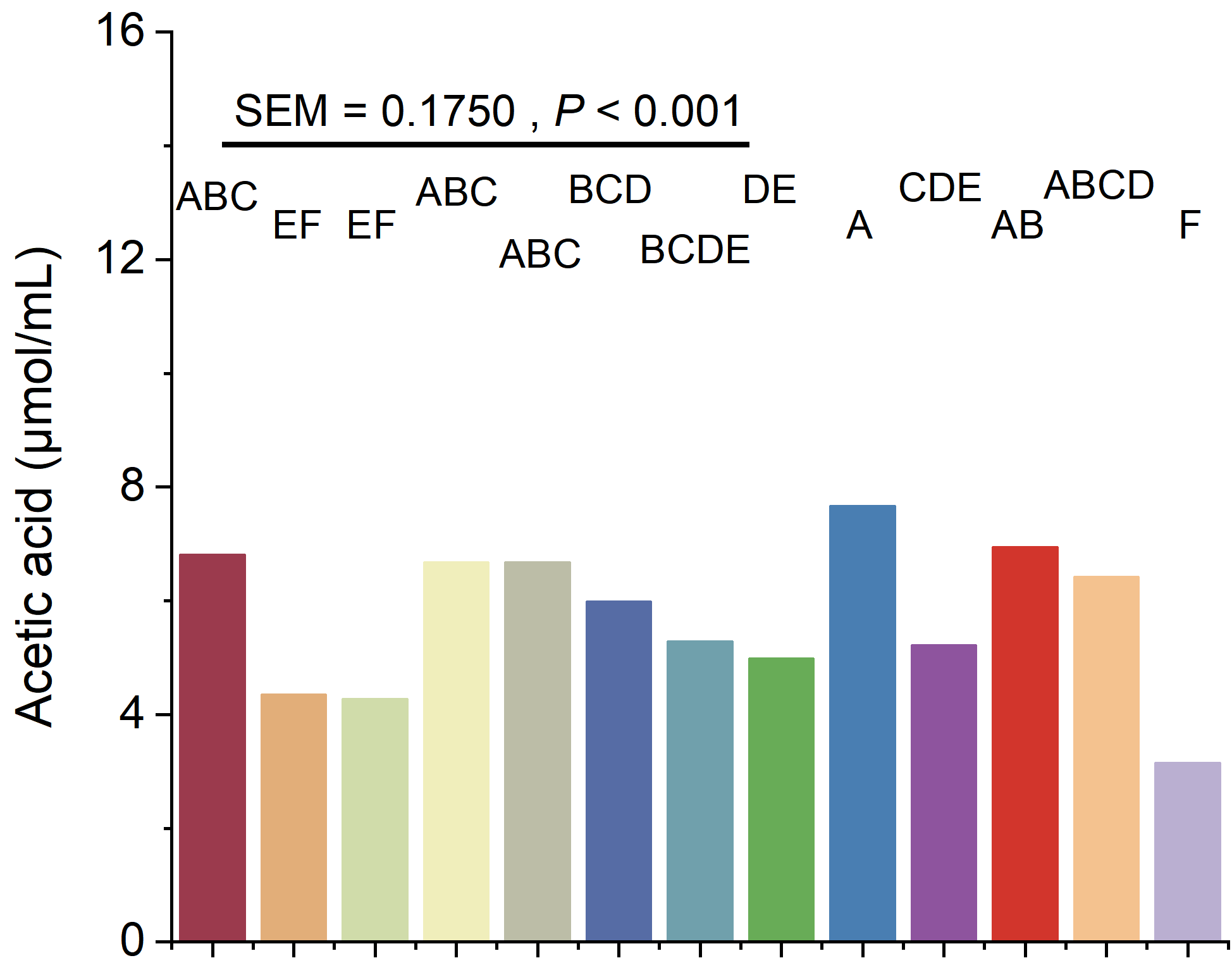

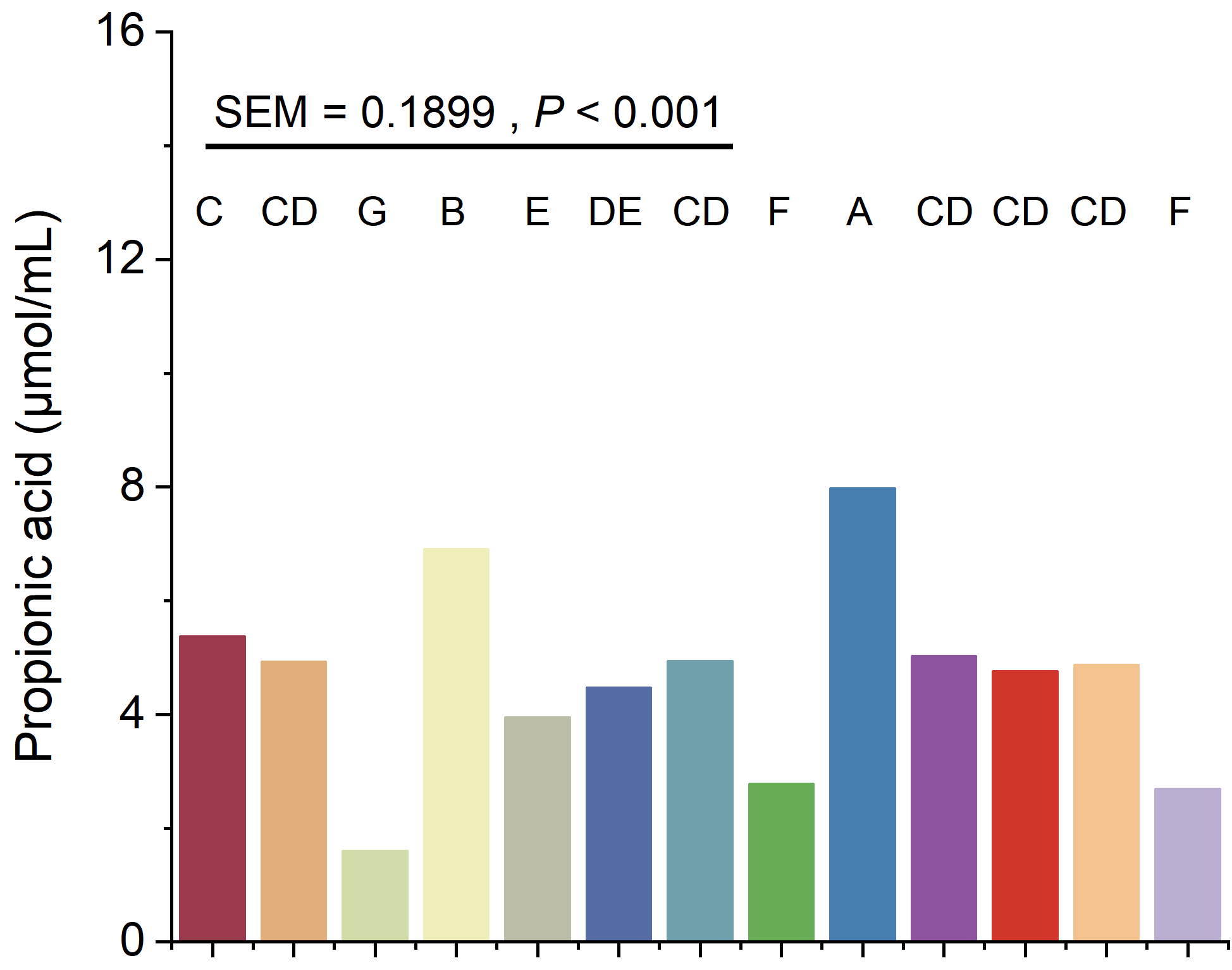

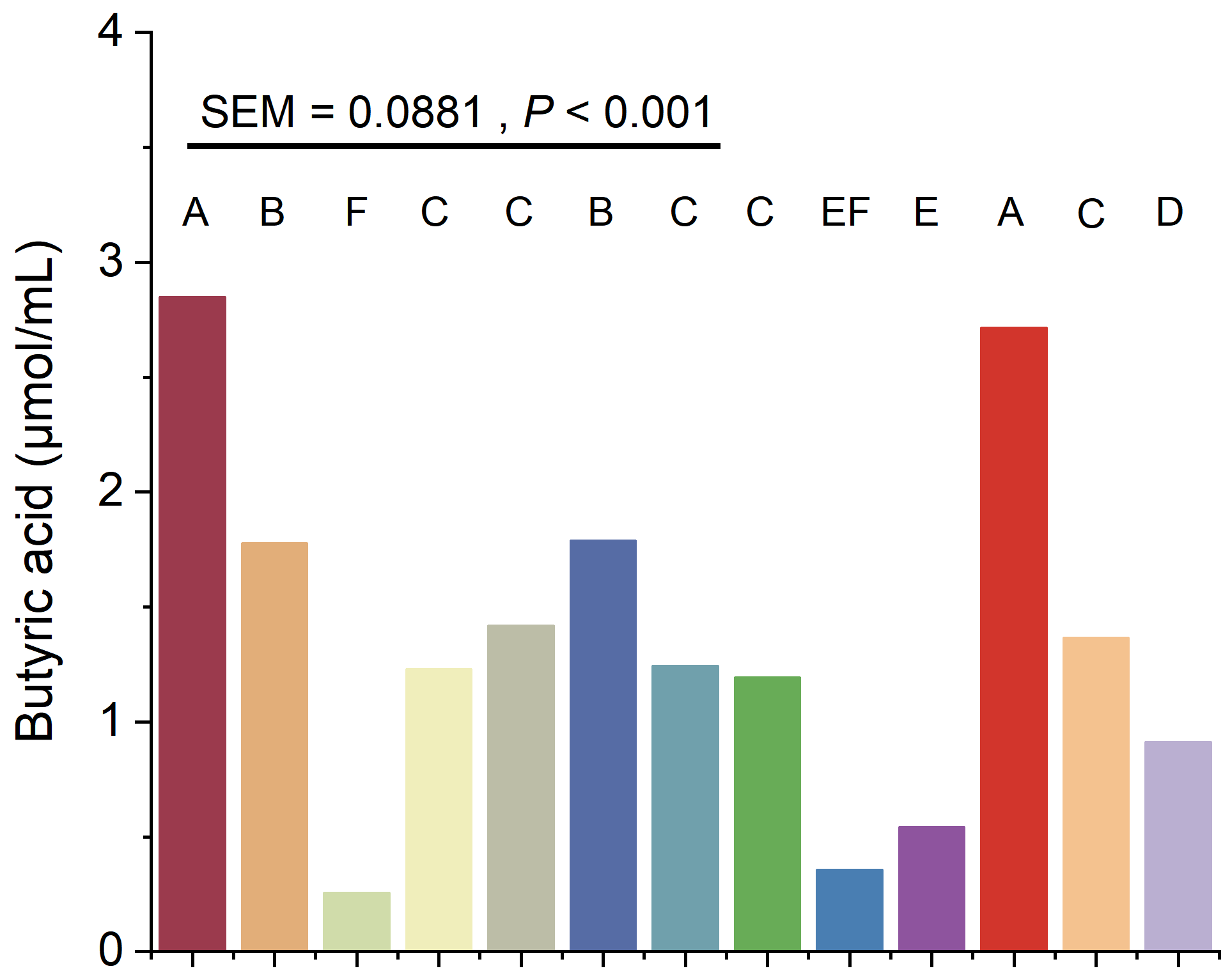


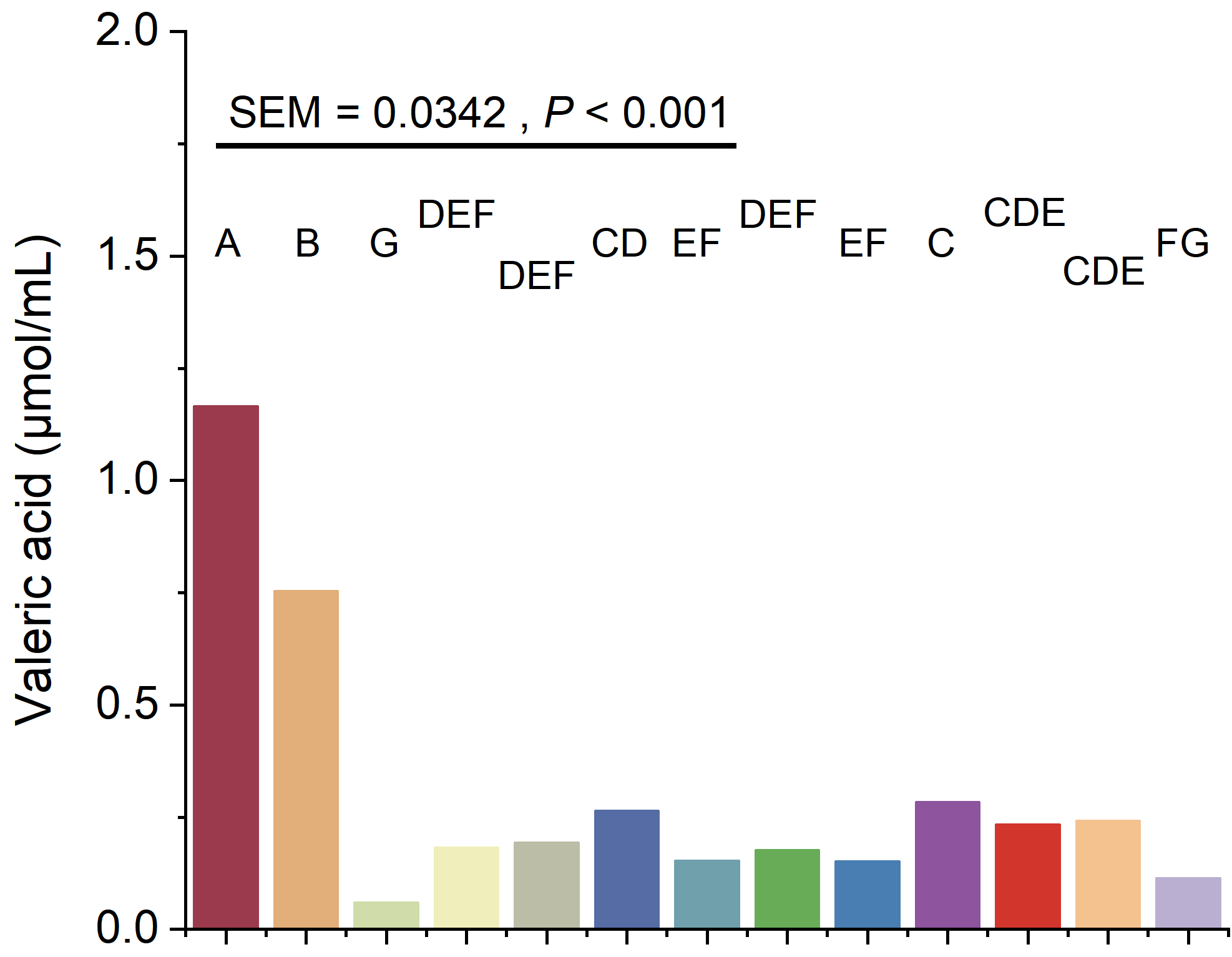

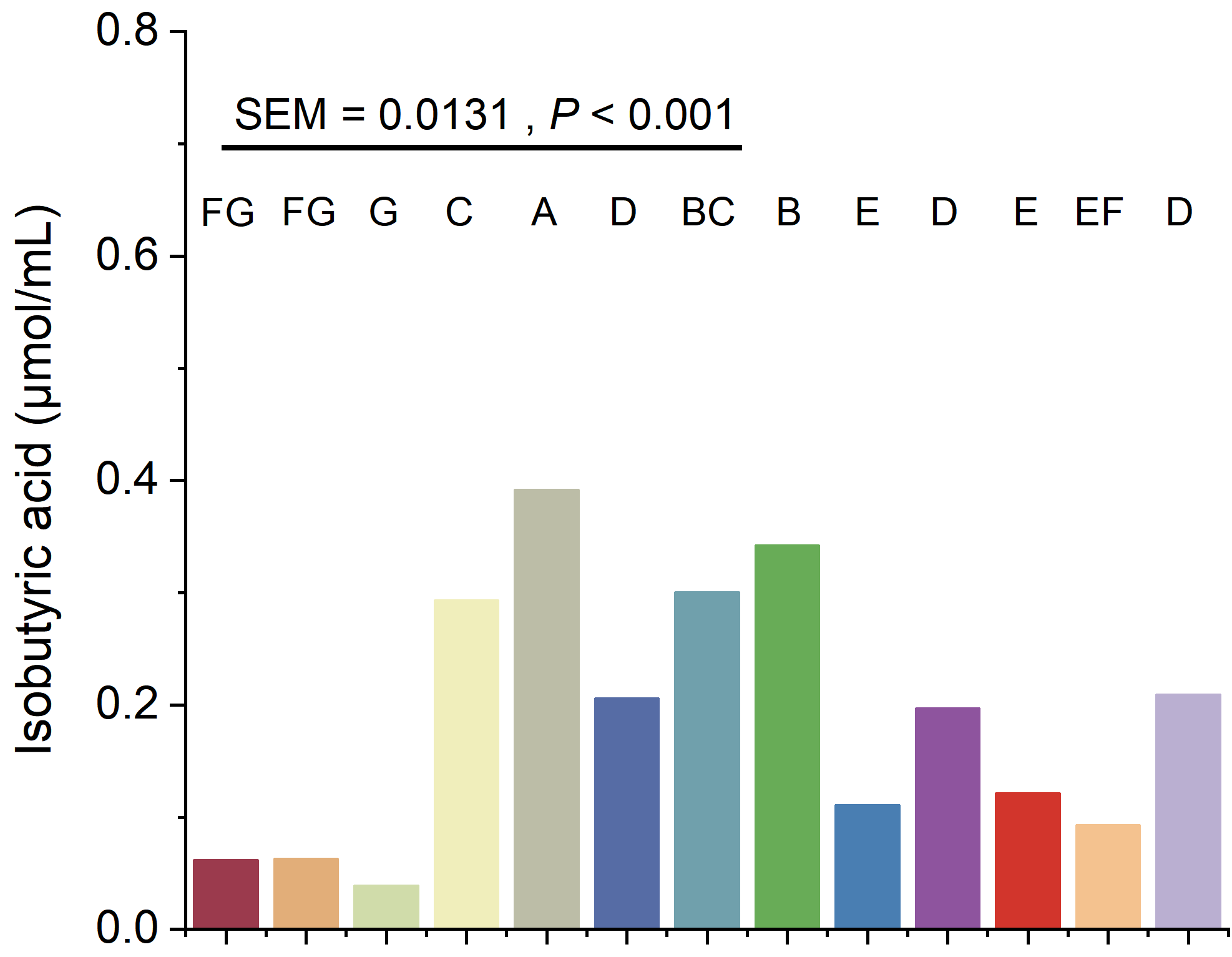

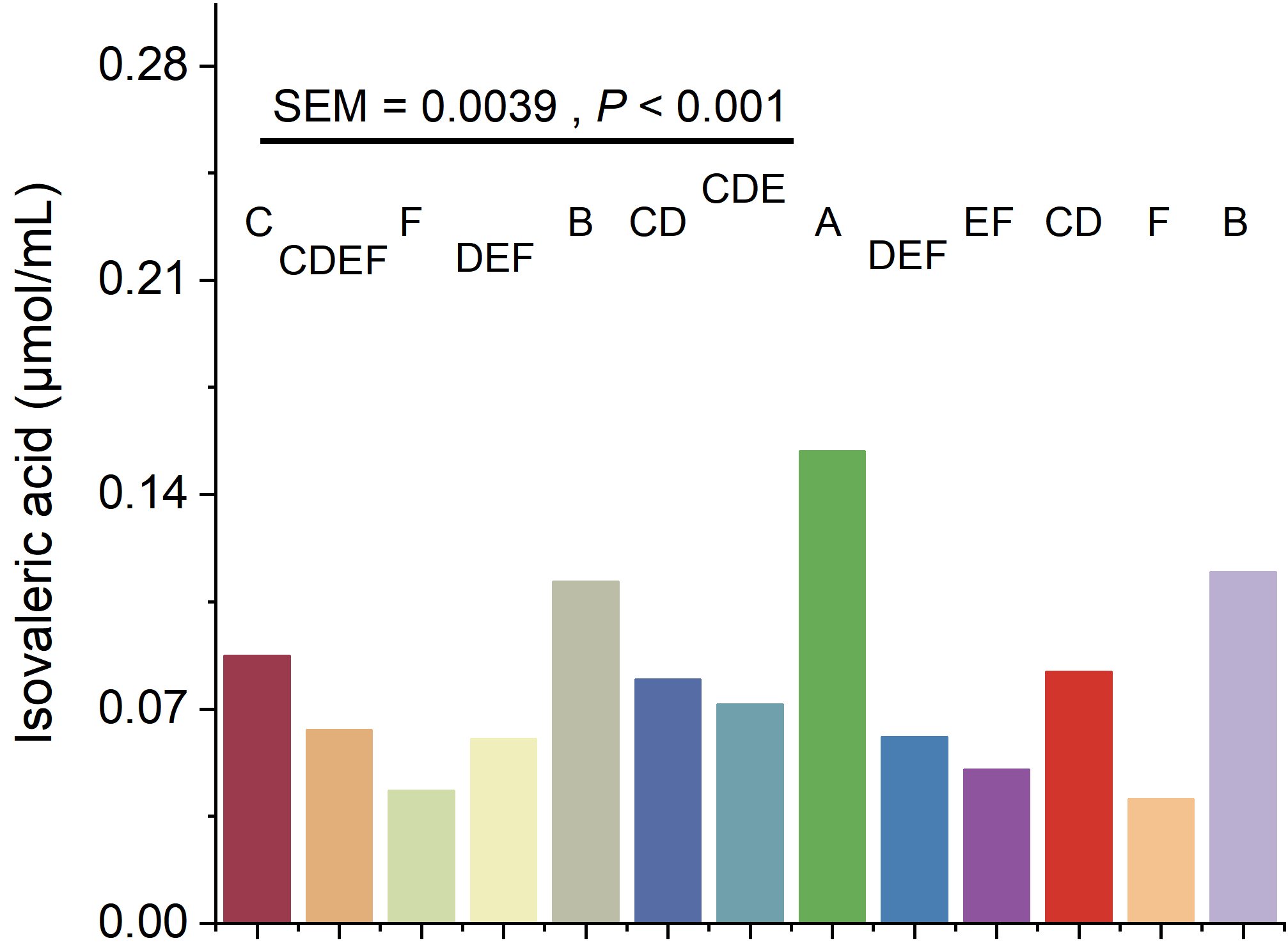


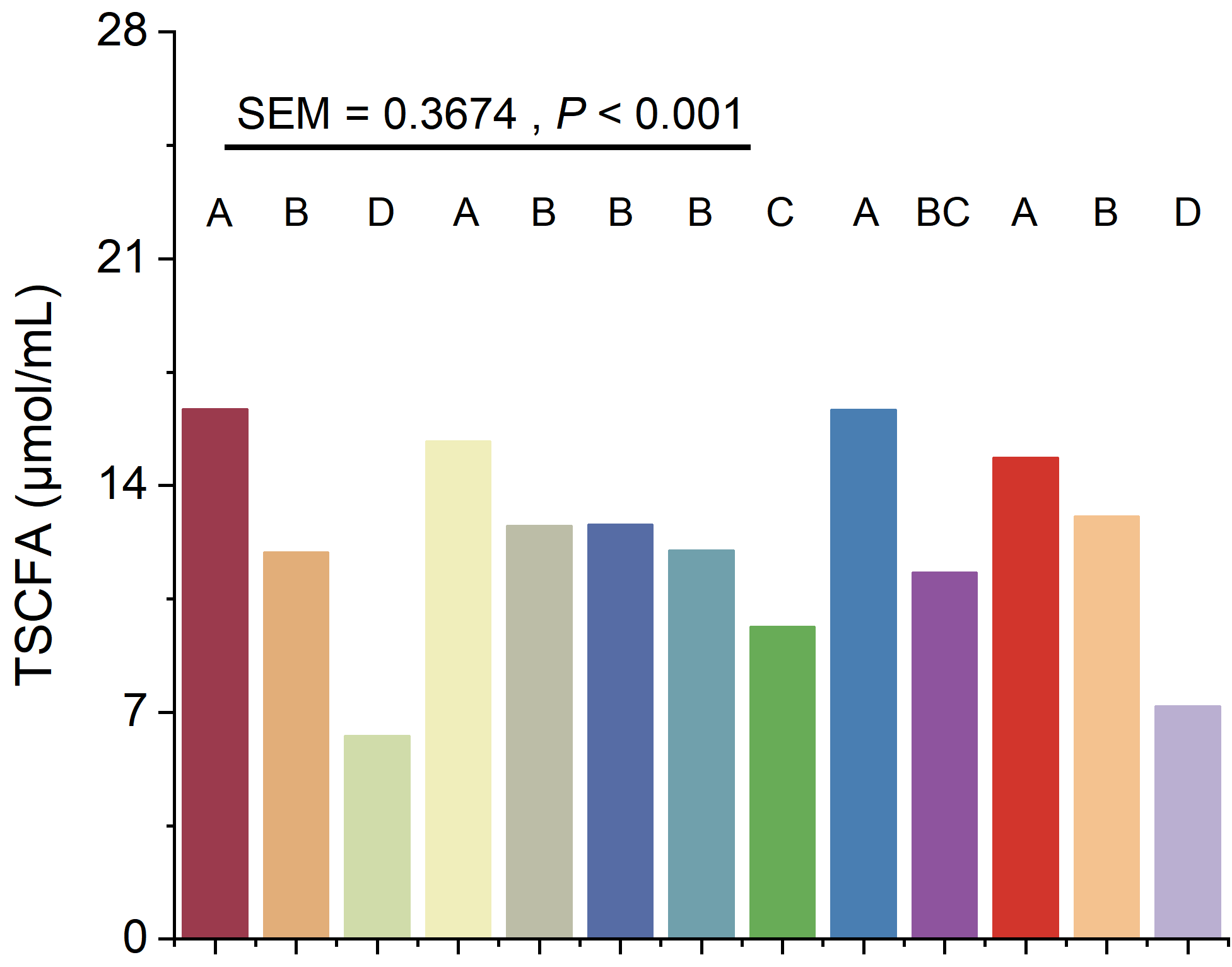

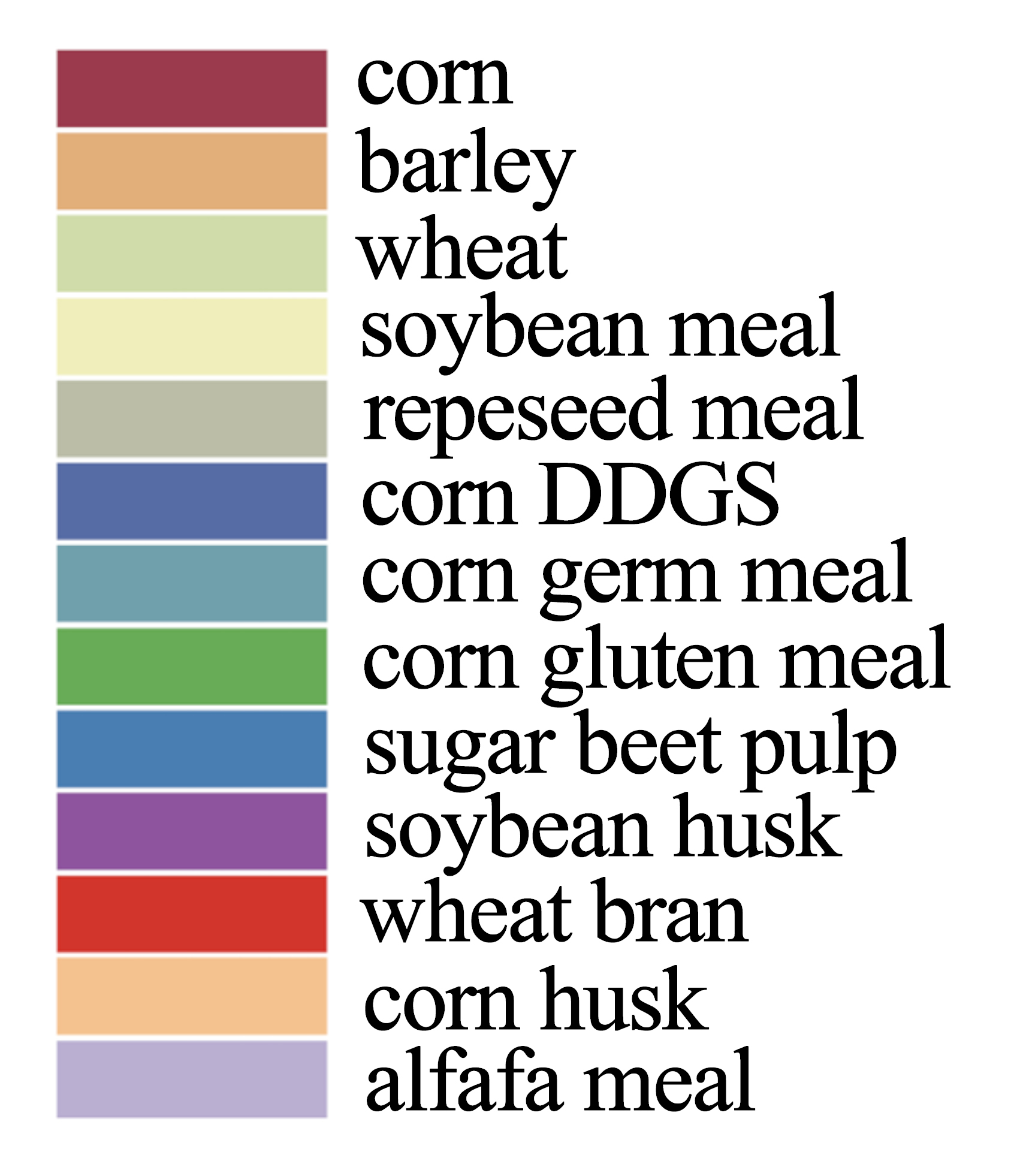


G


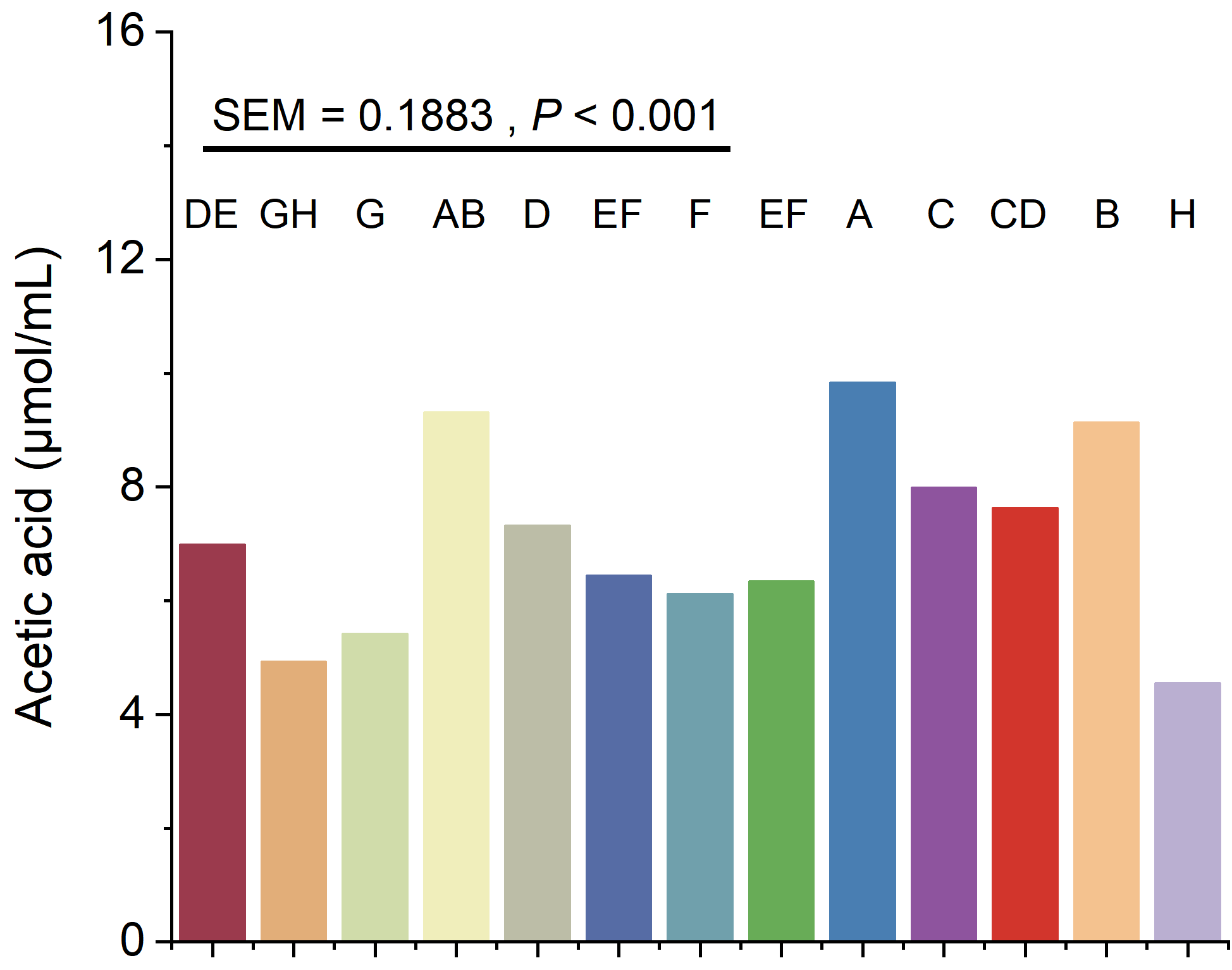

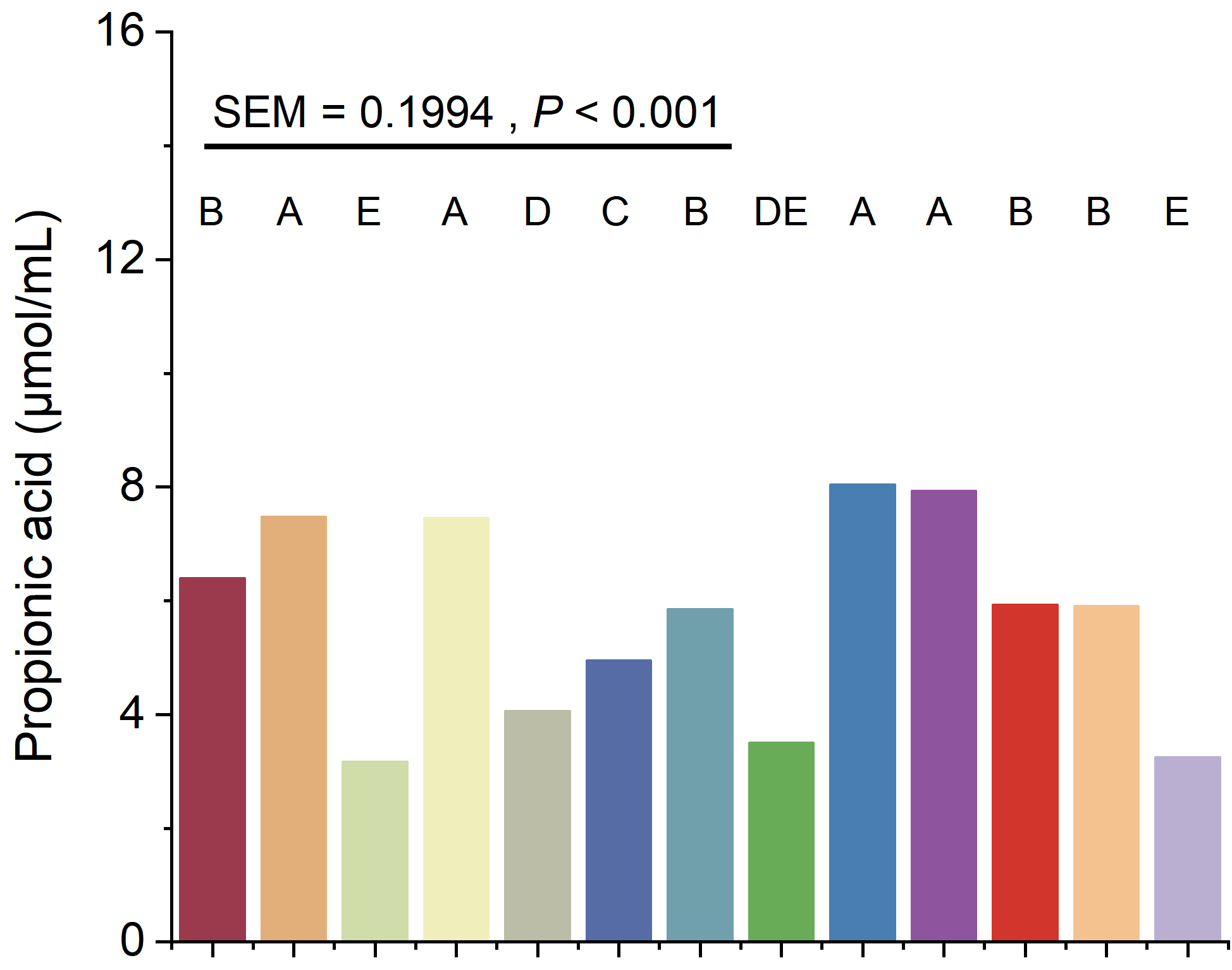

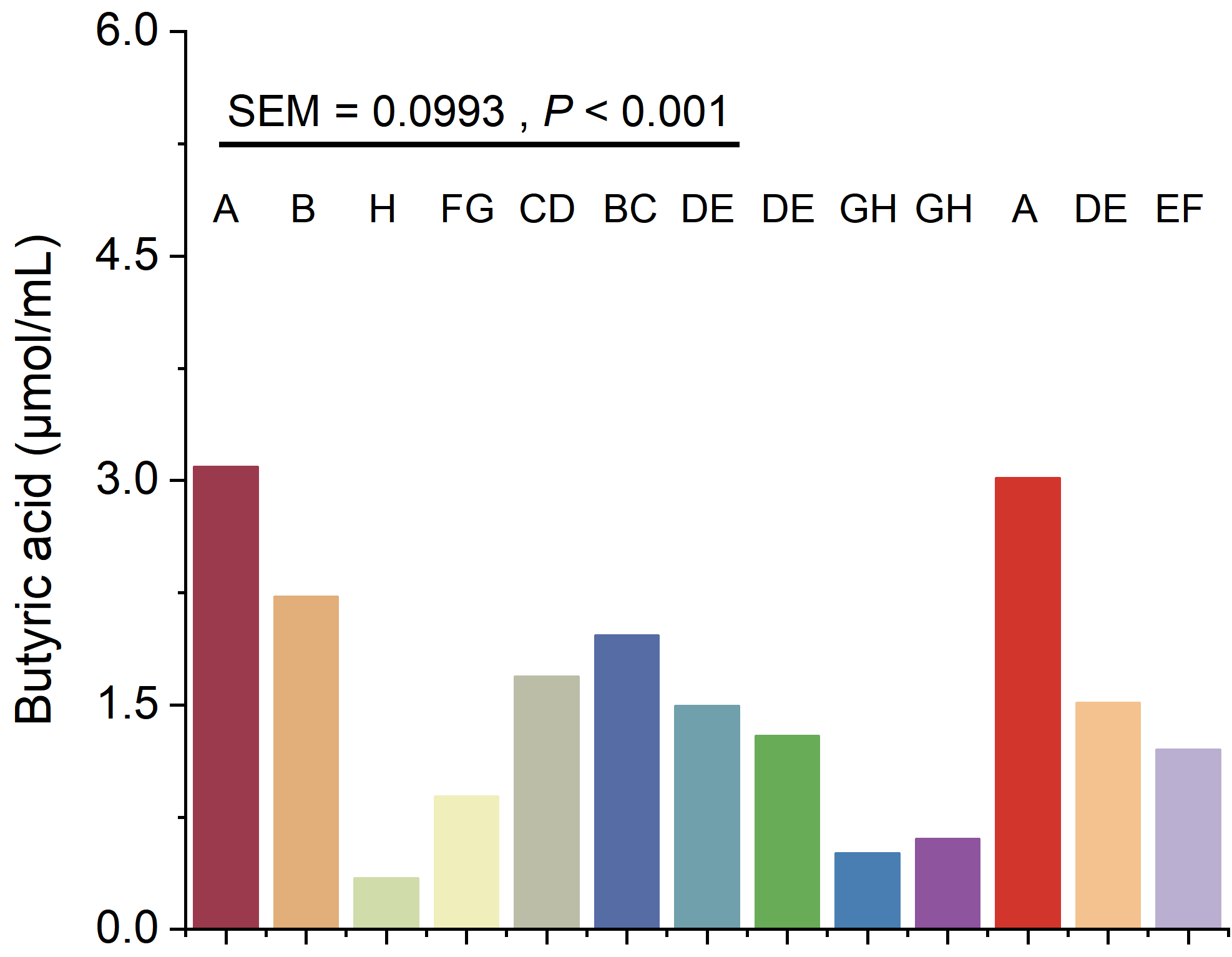


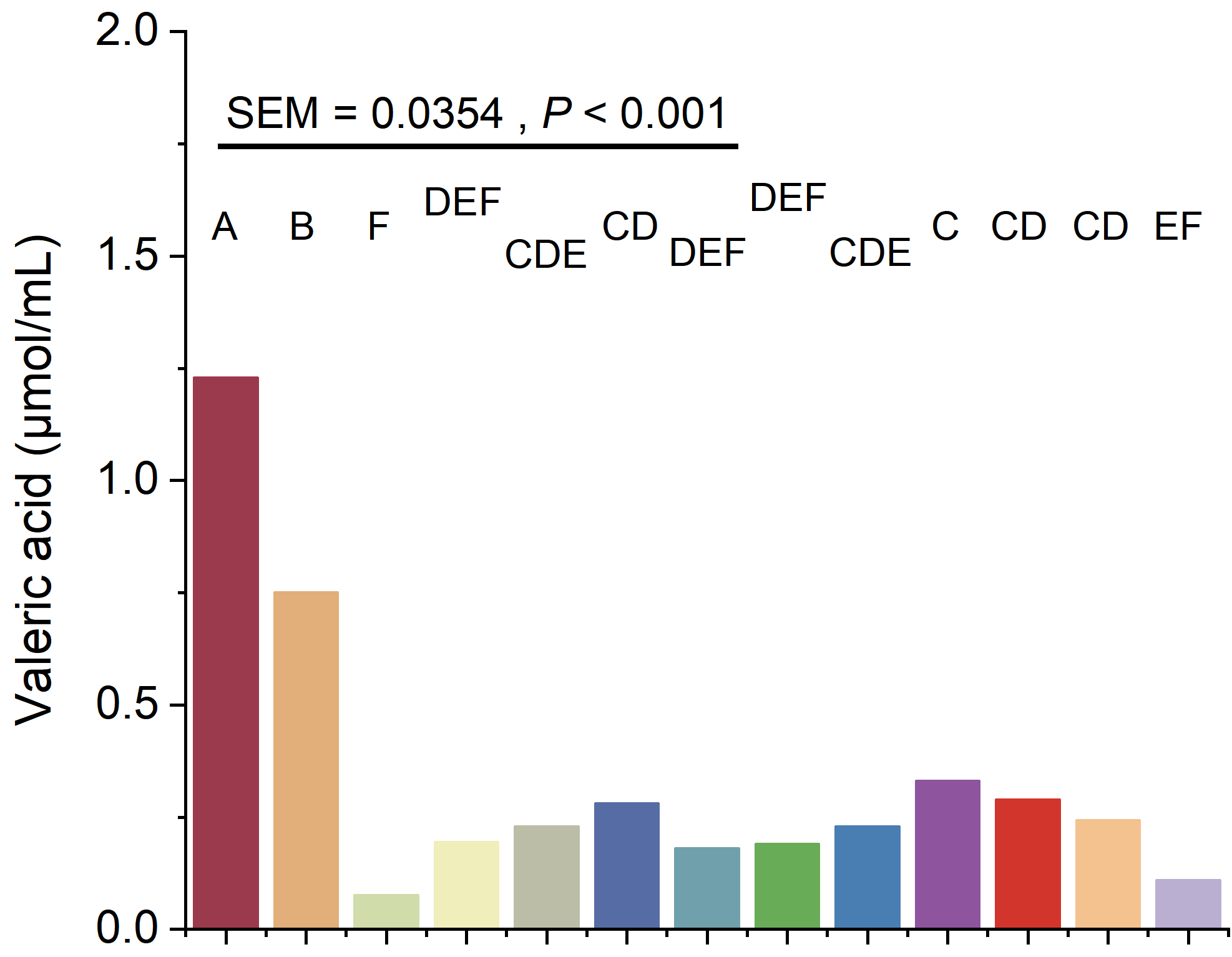

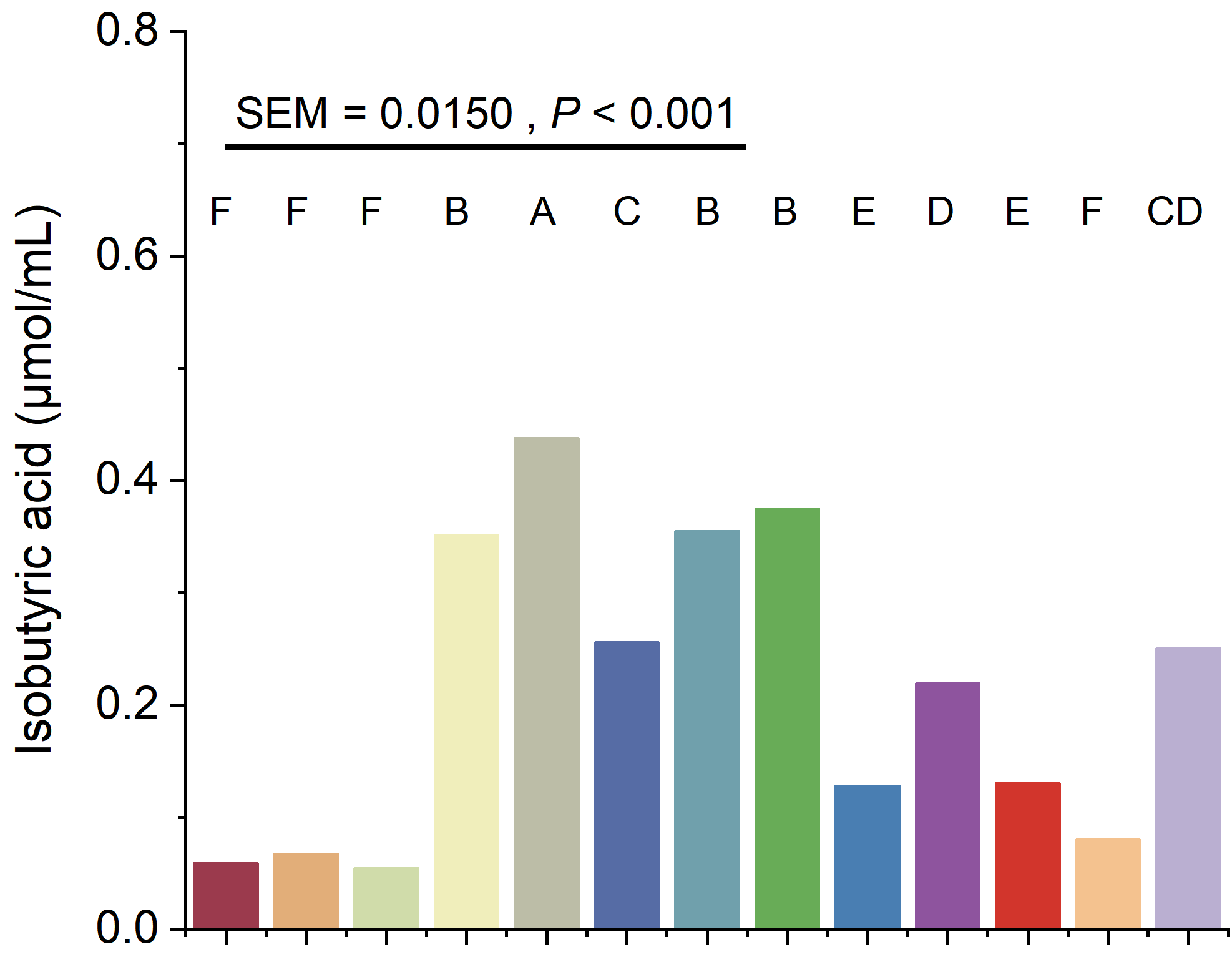

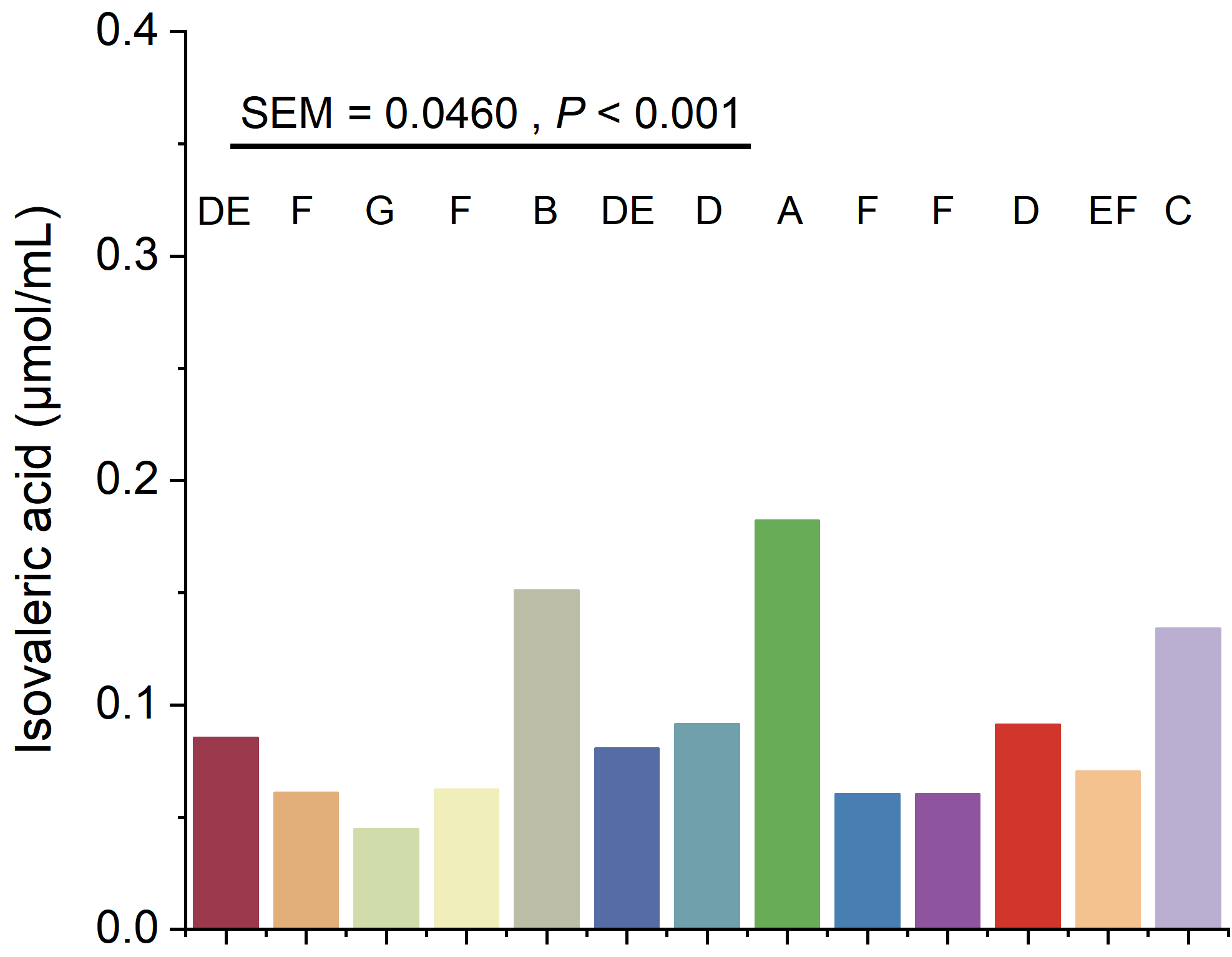


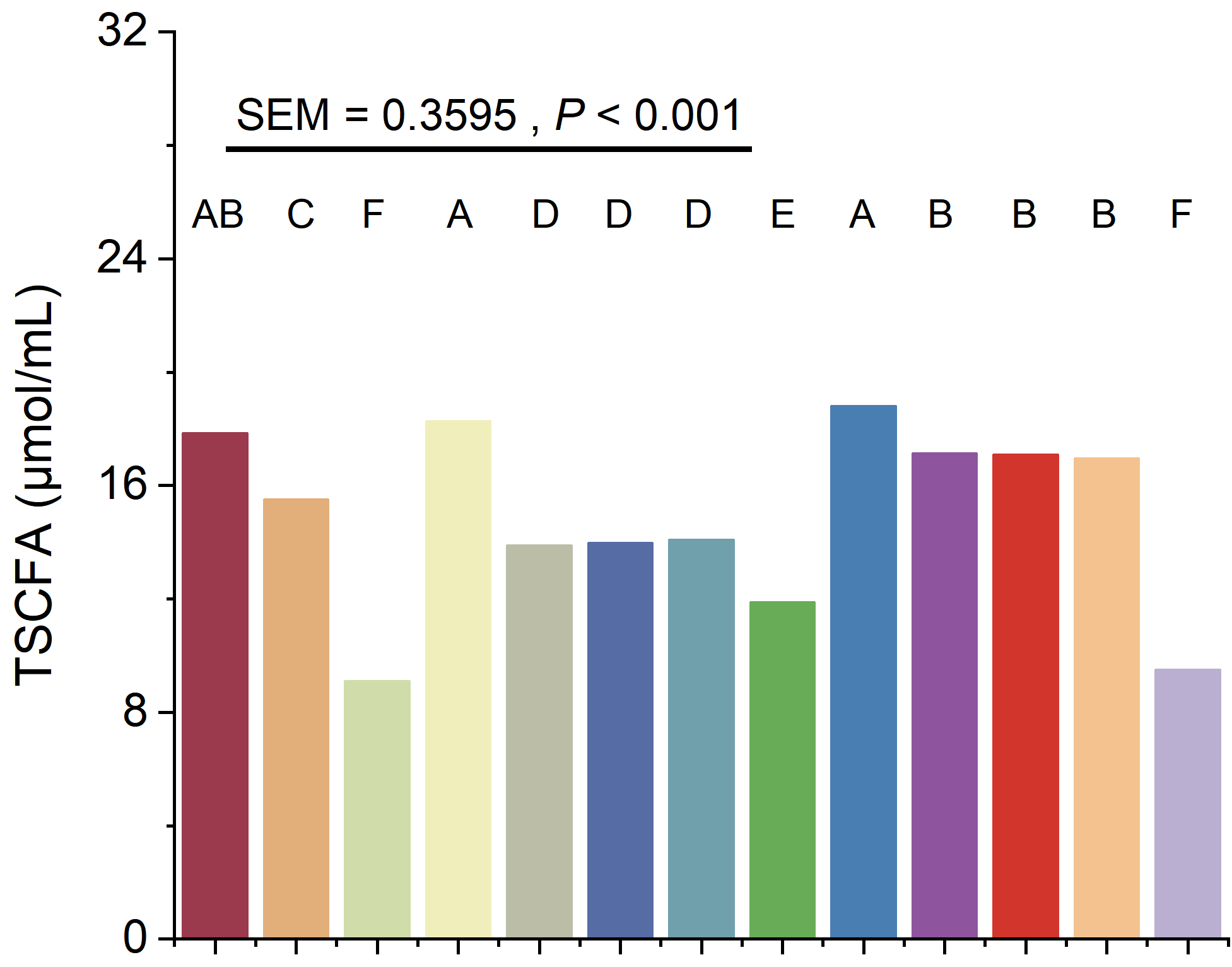

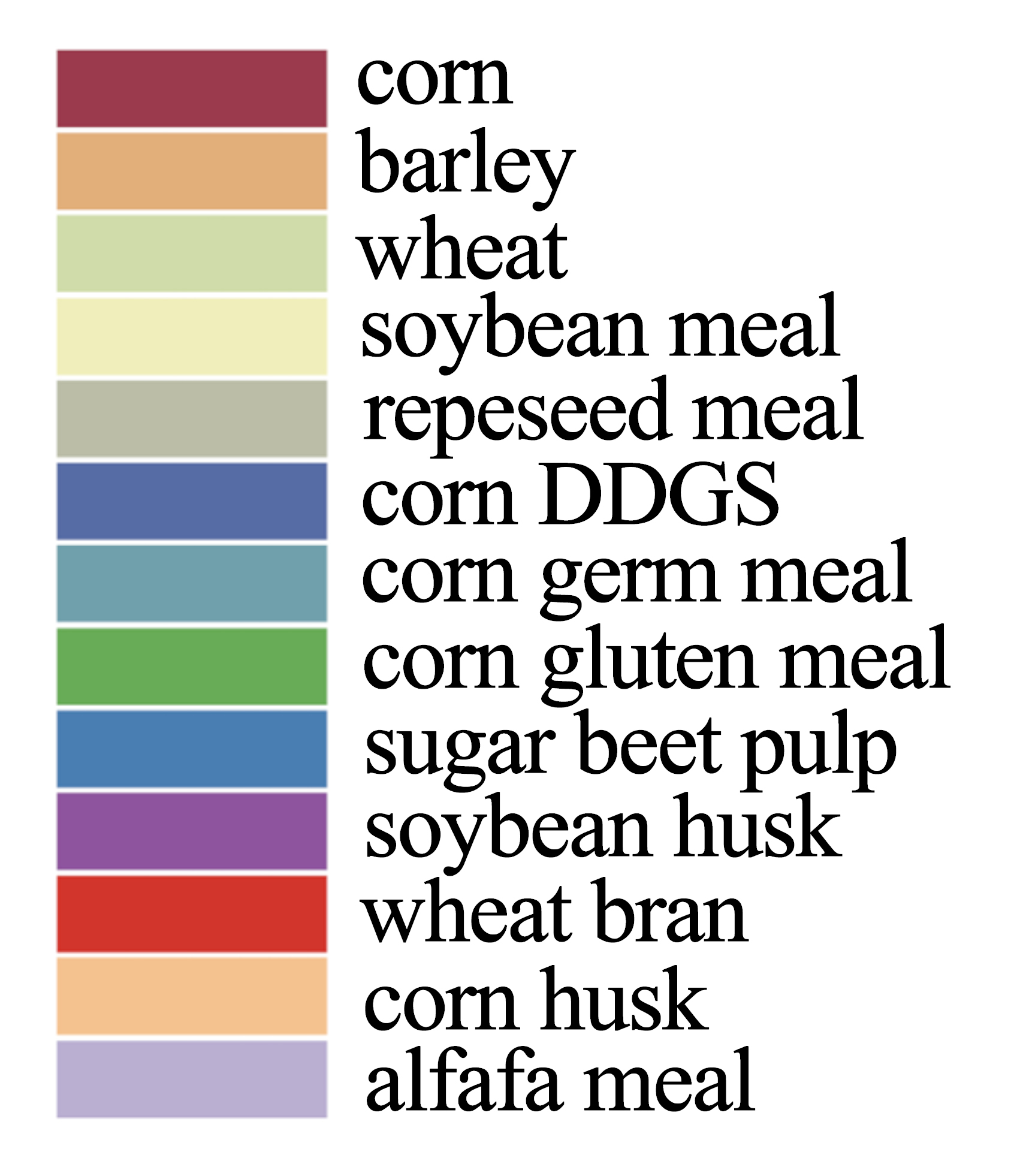


H


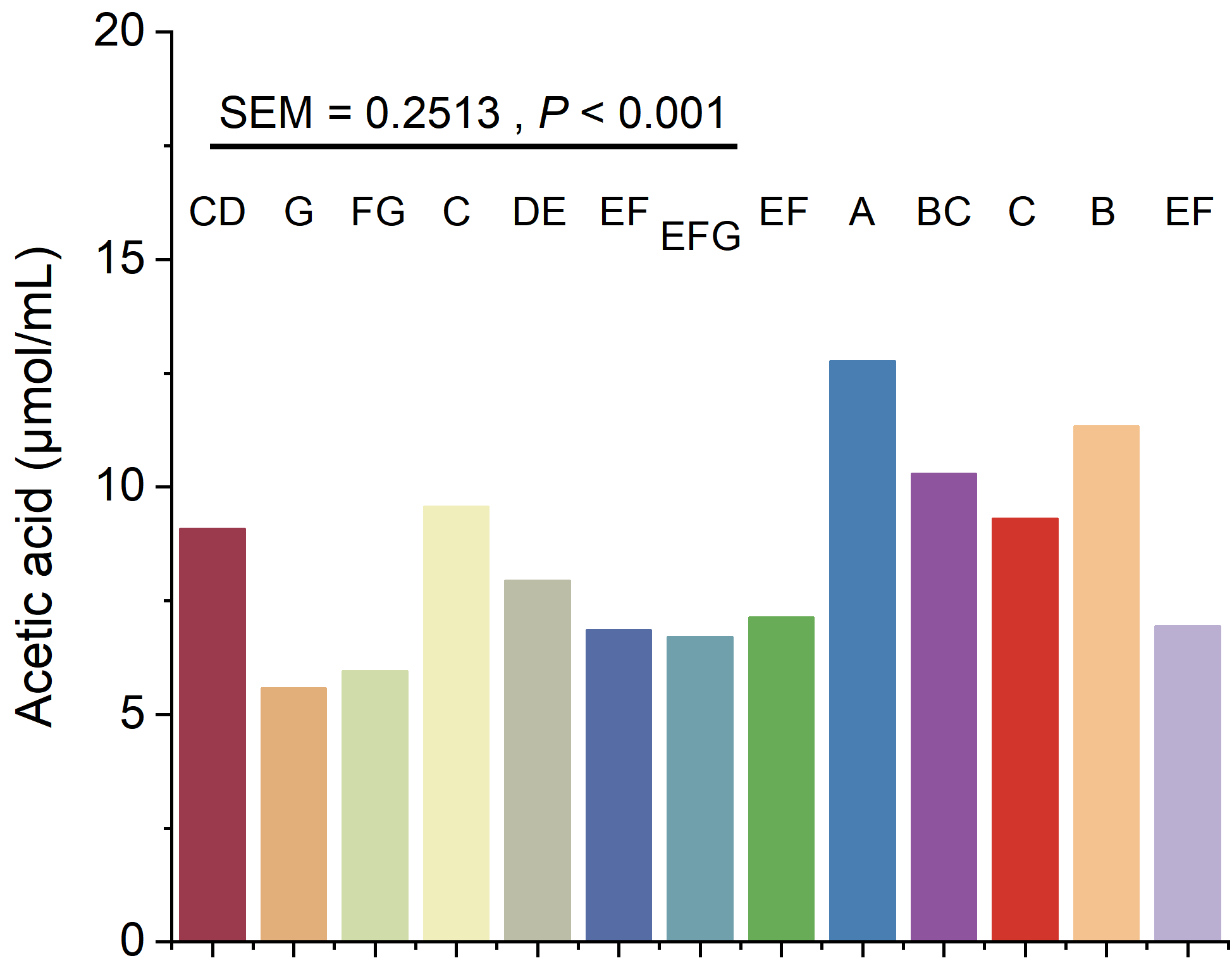

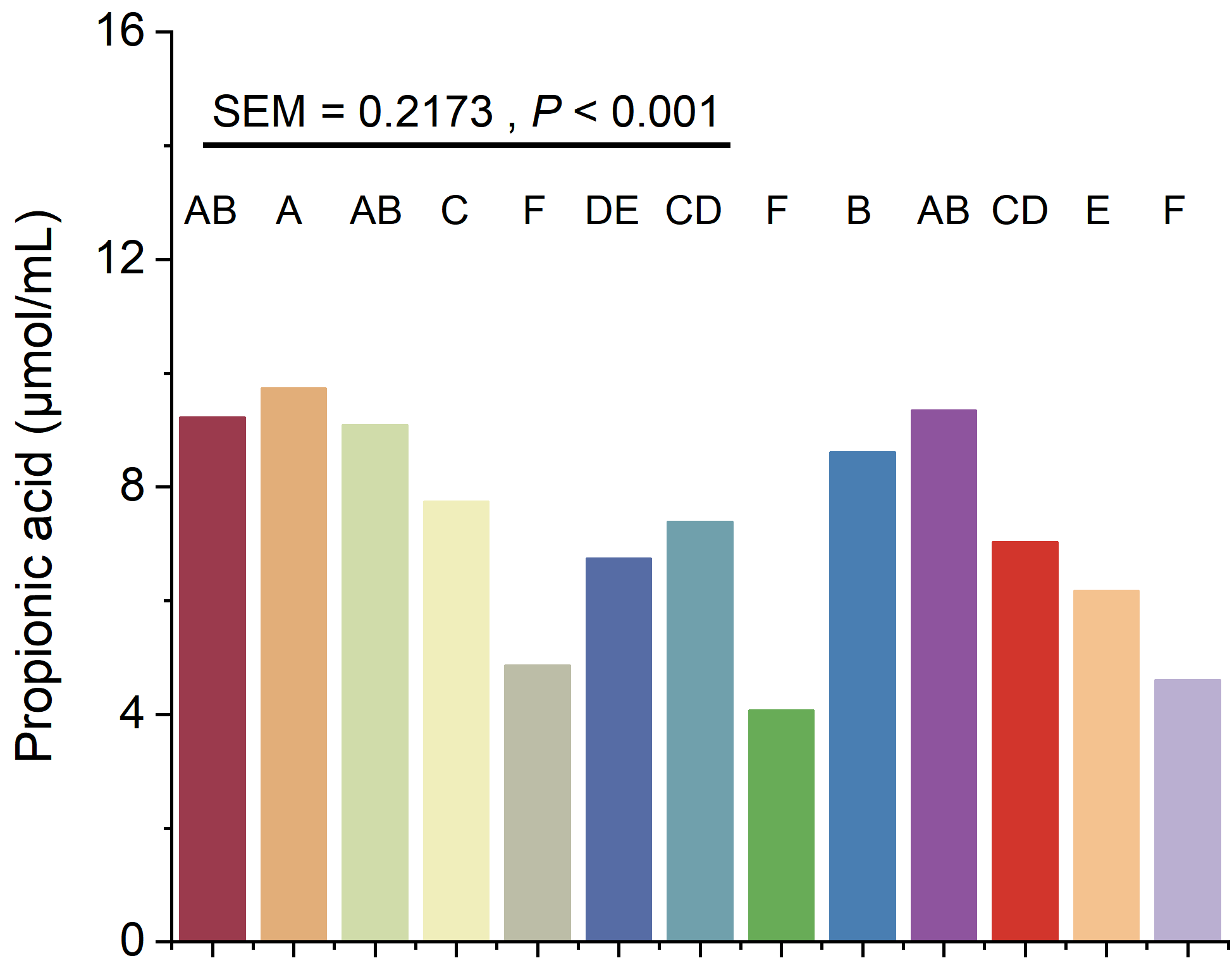

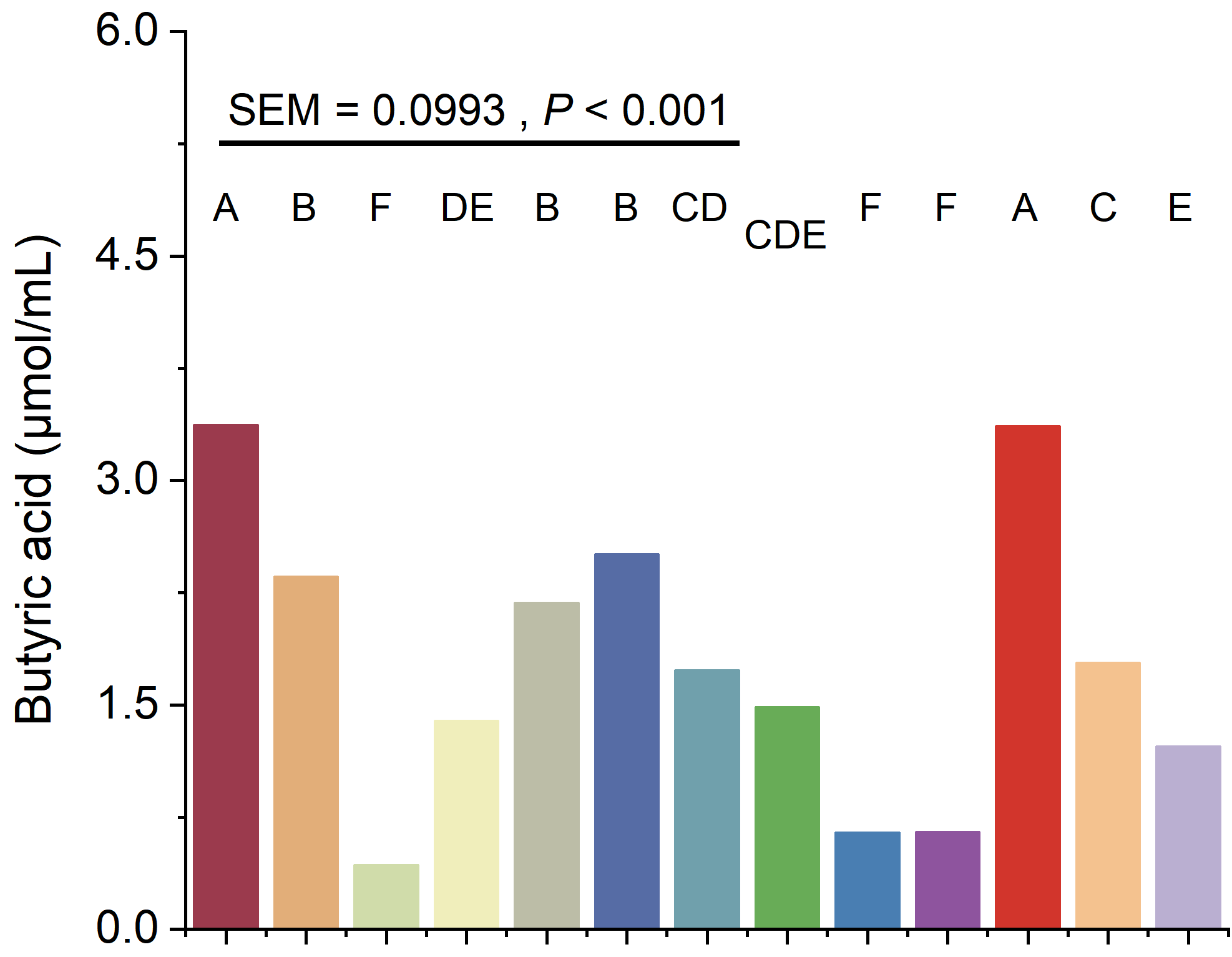


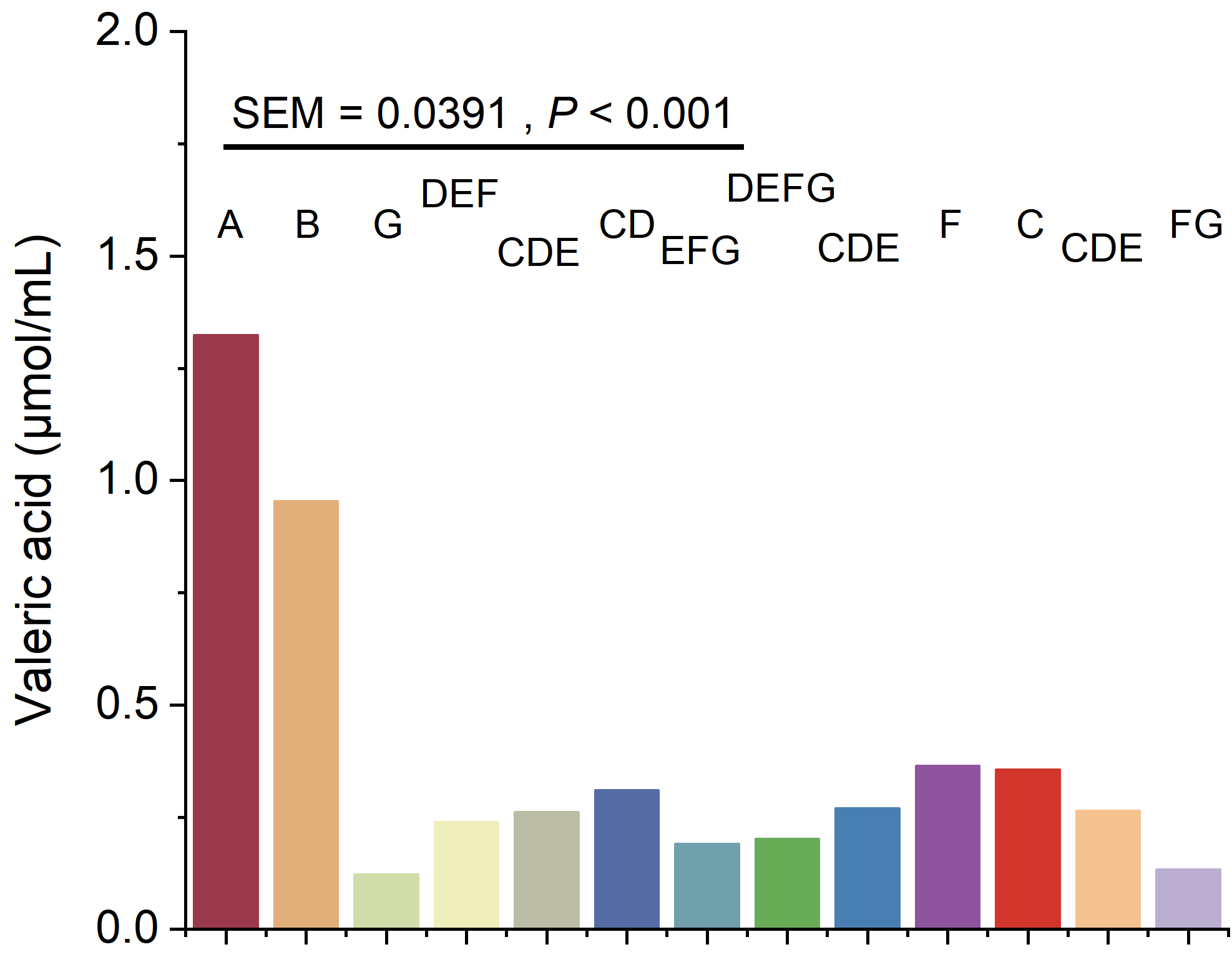

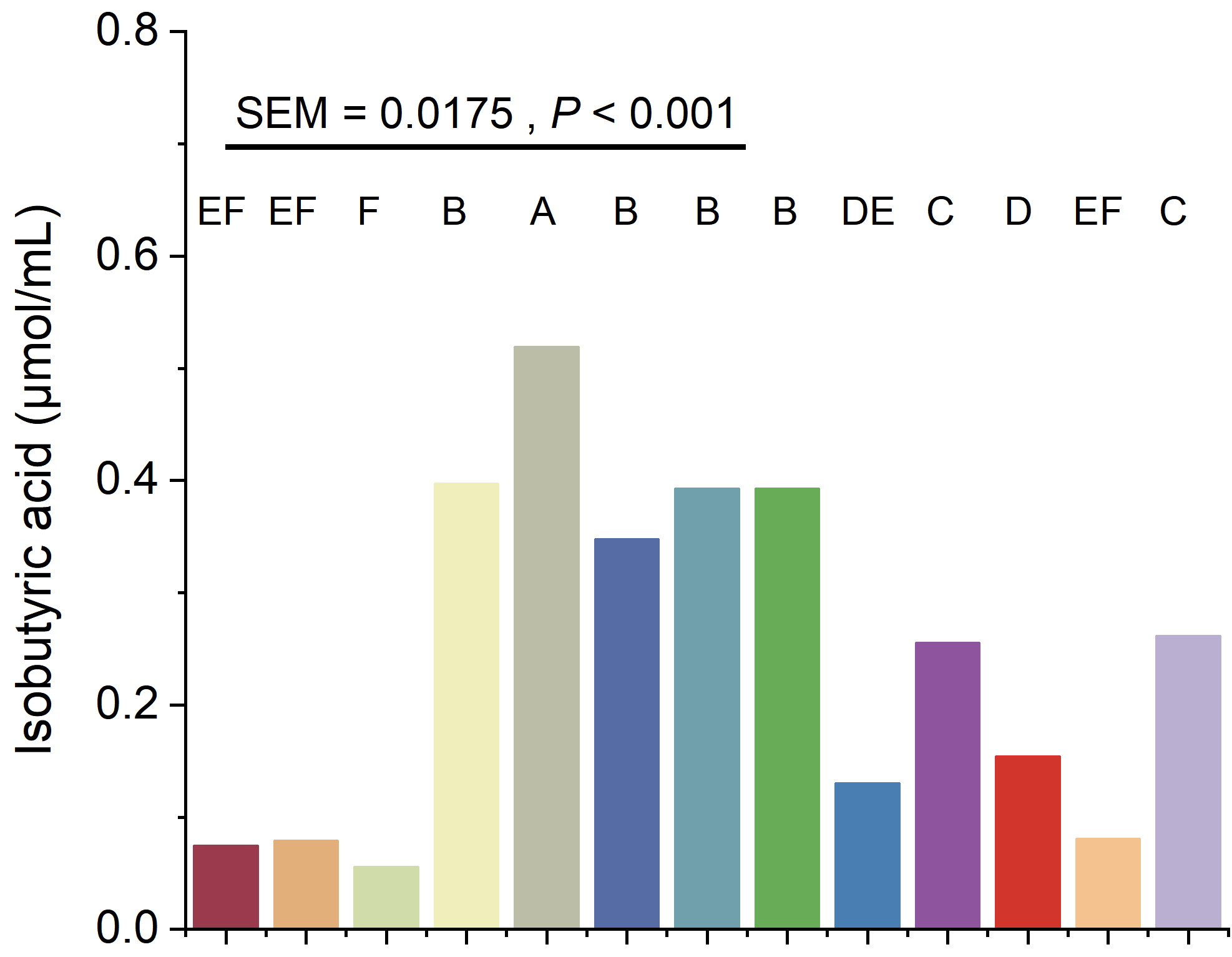

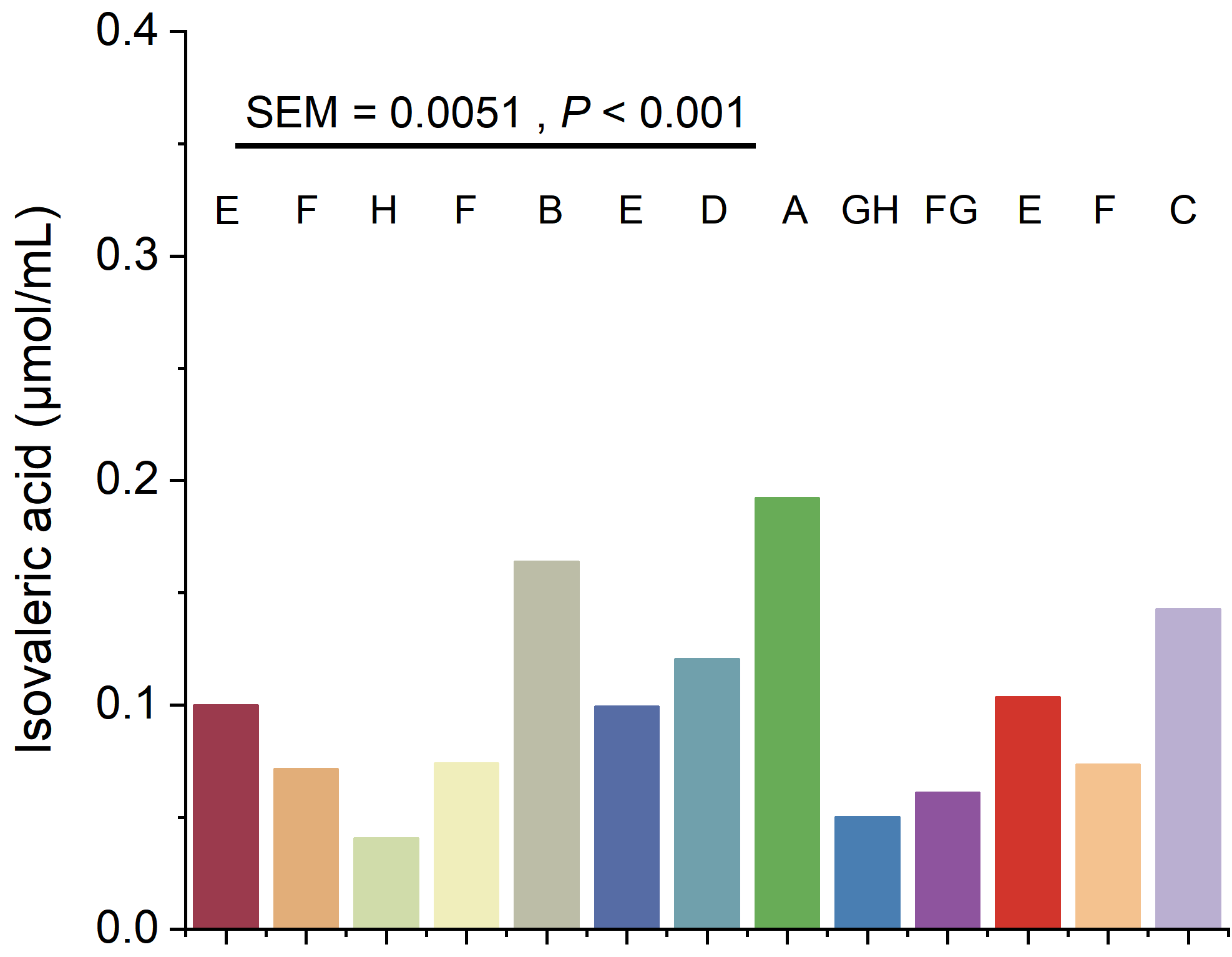


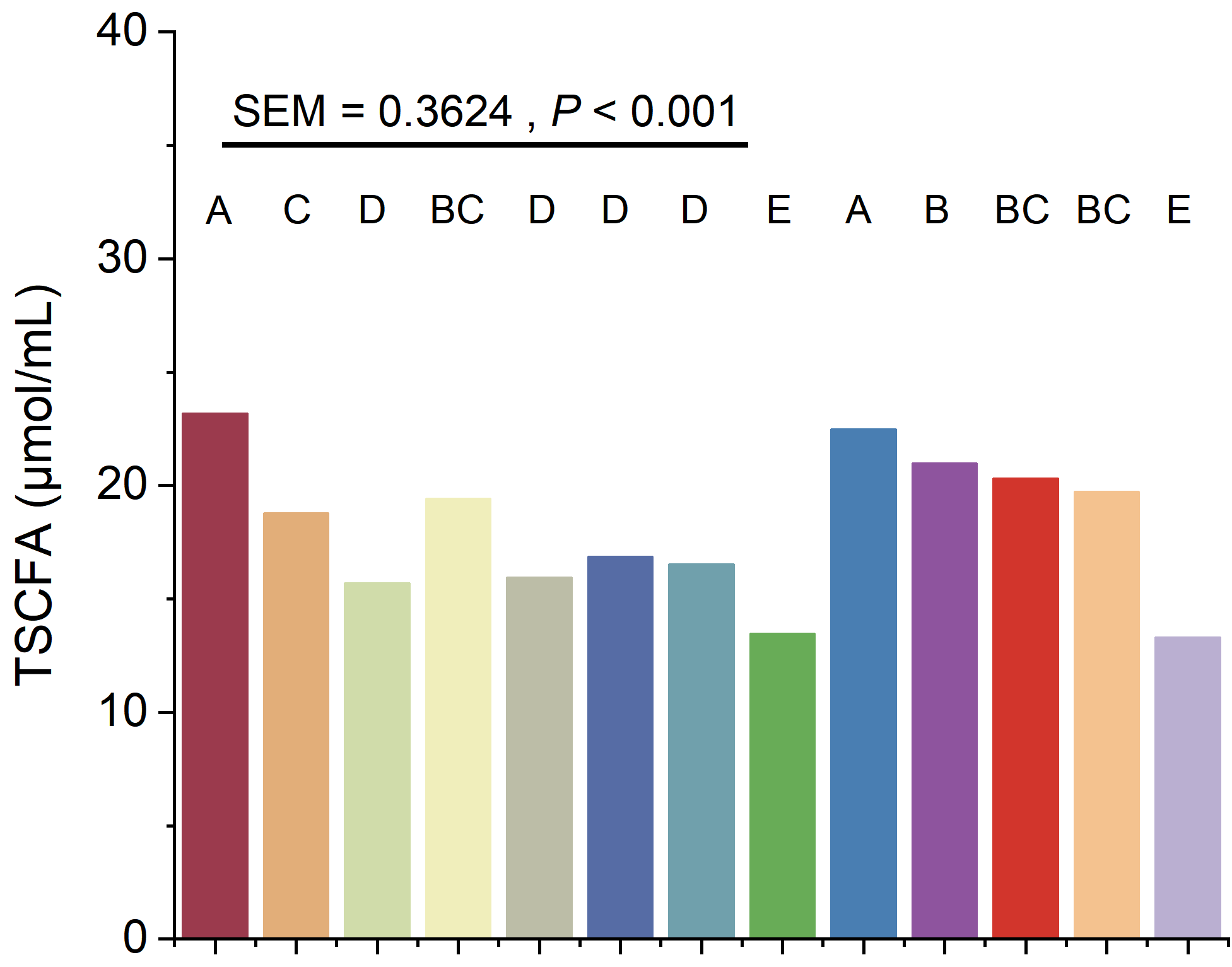

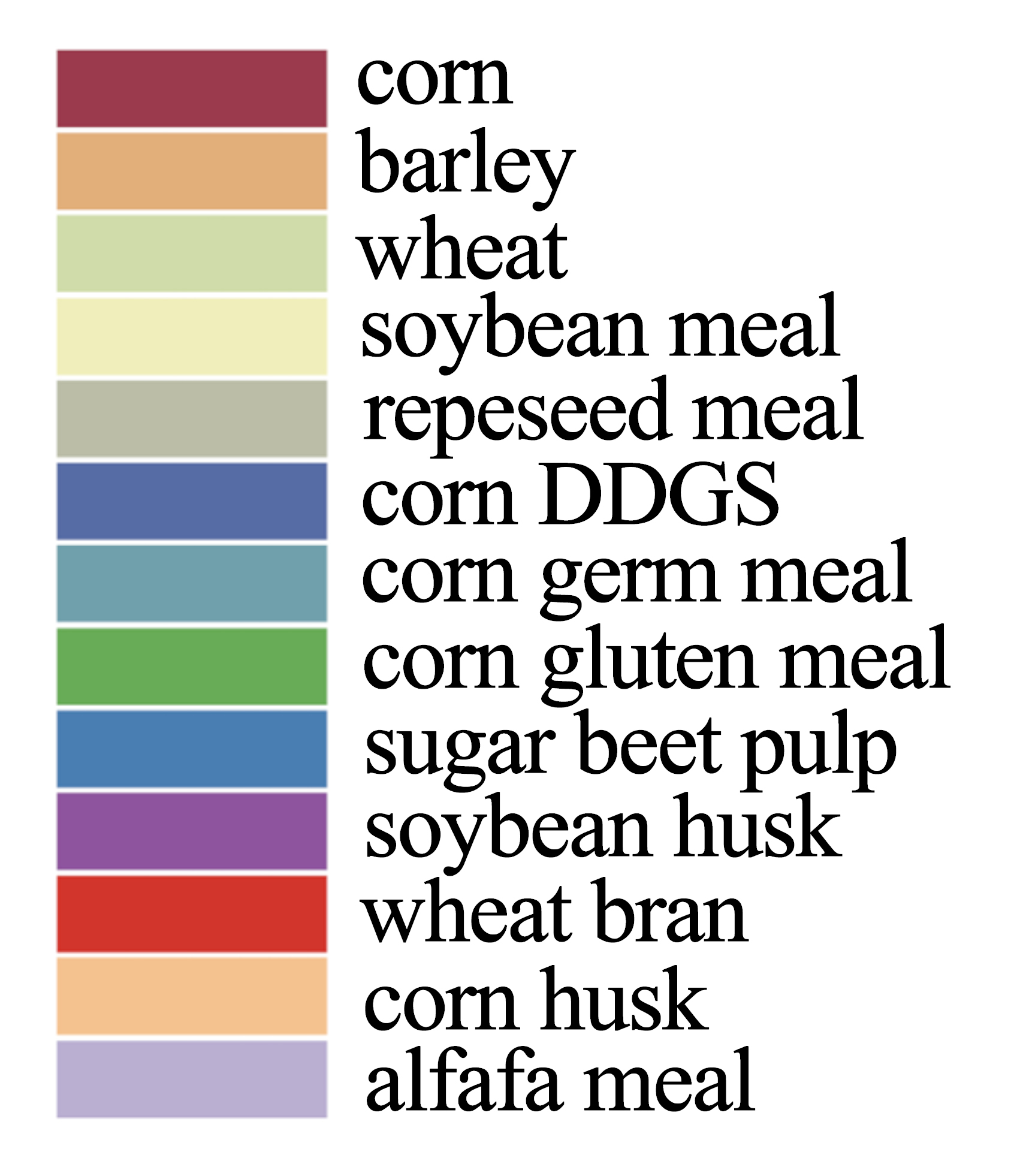


I


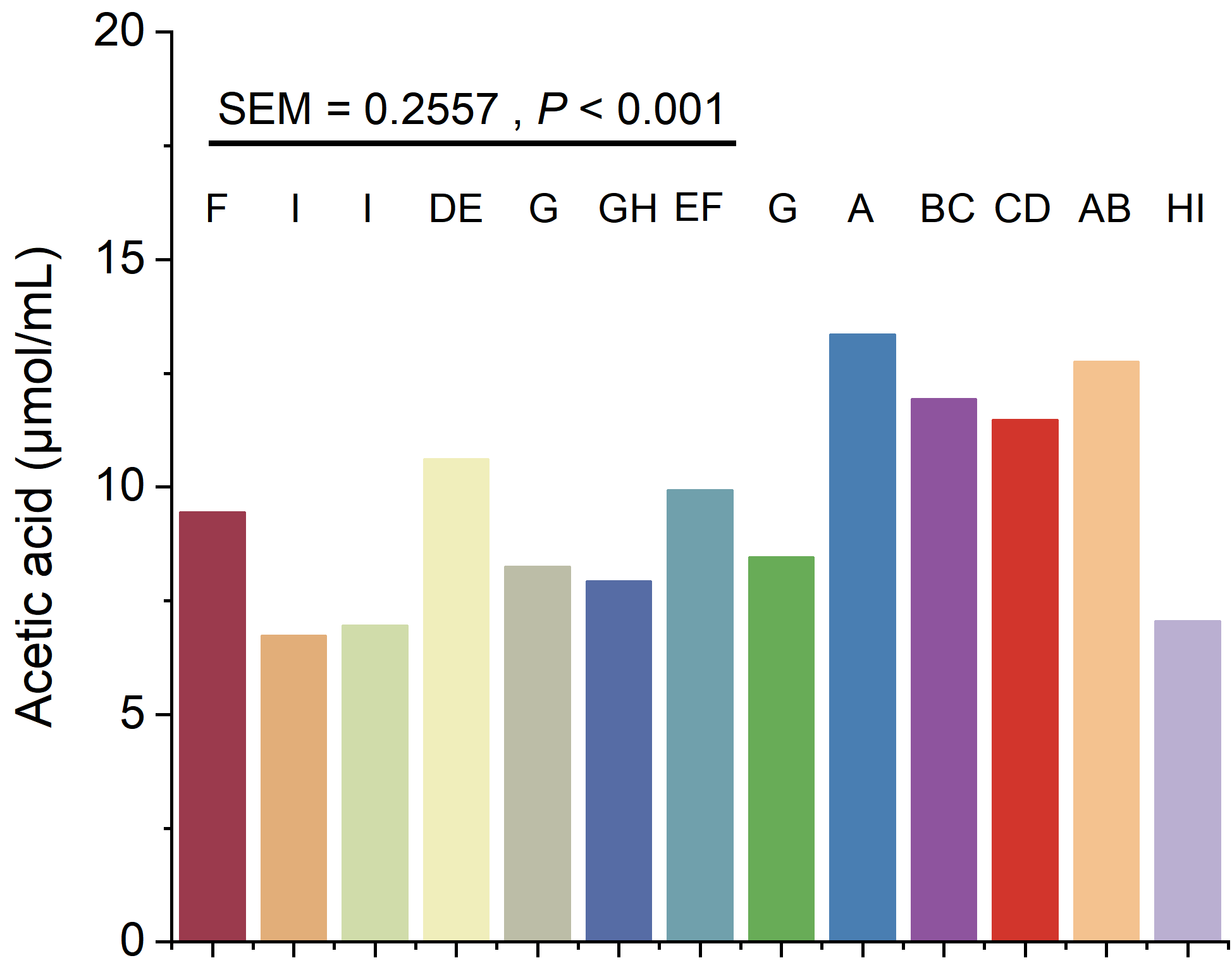

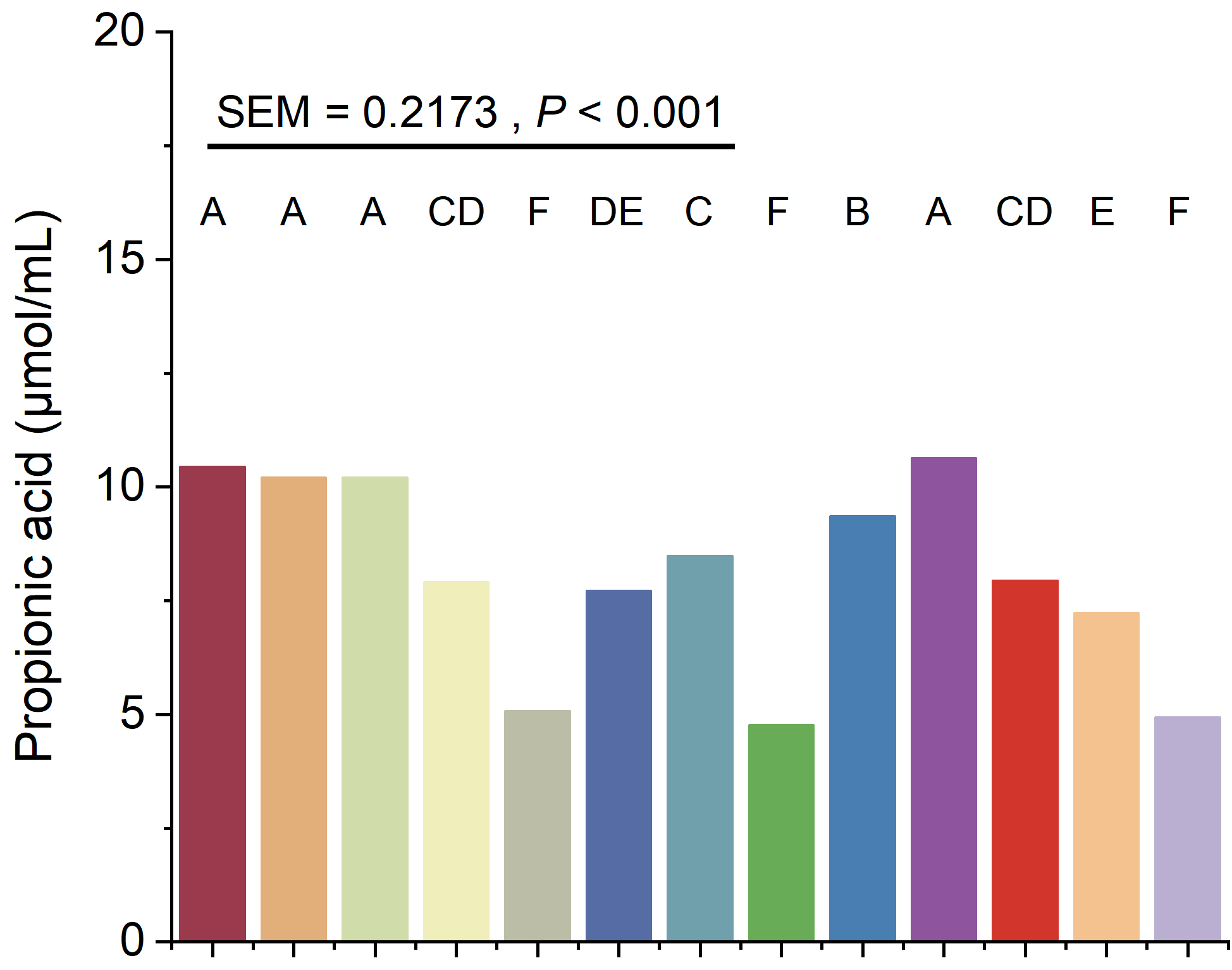

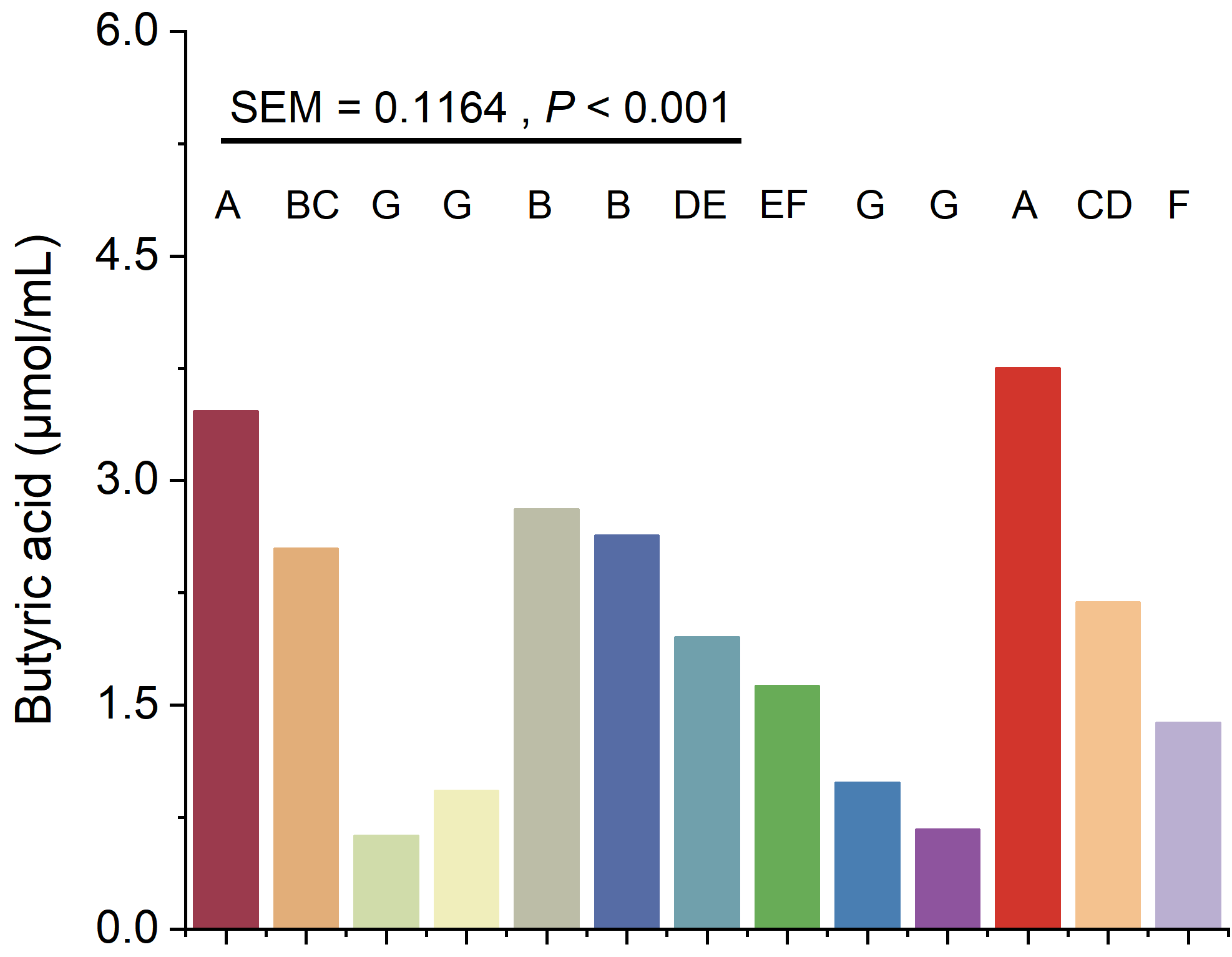


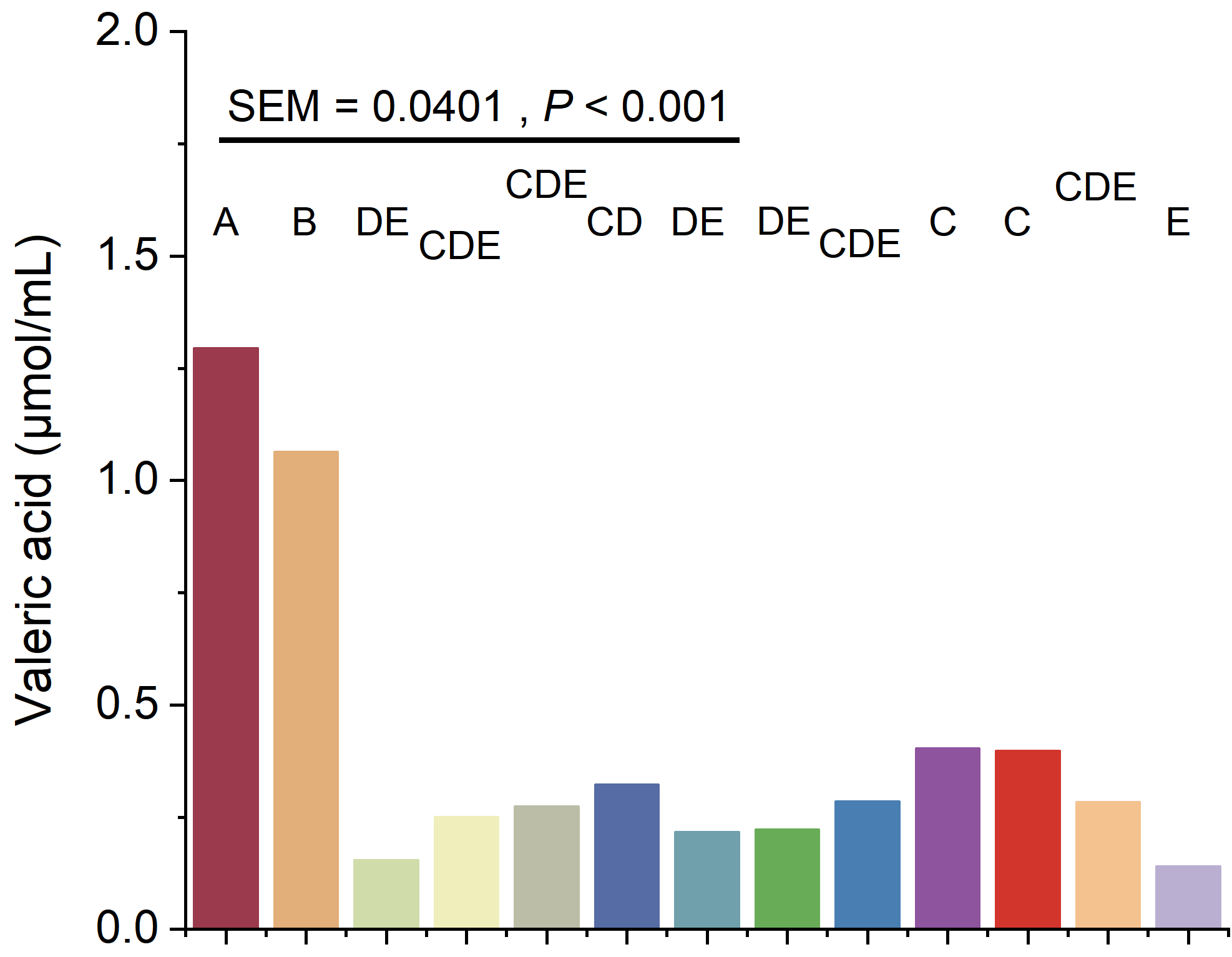

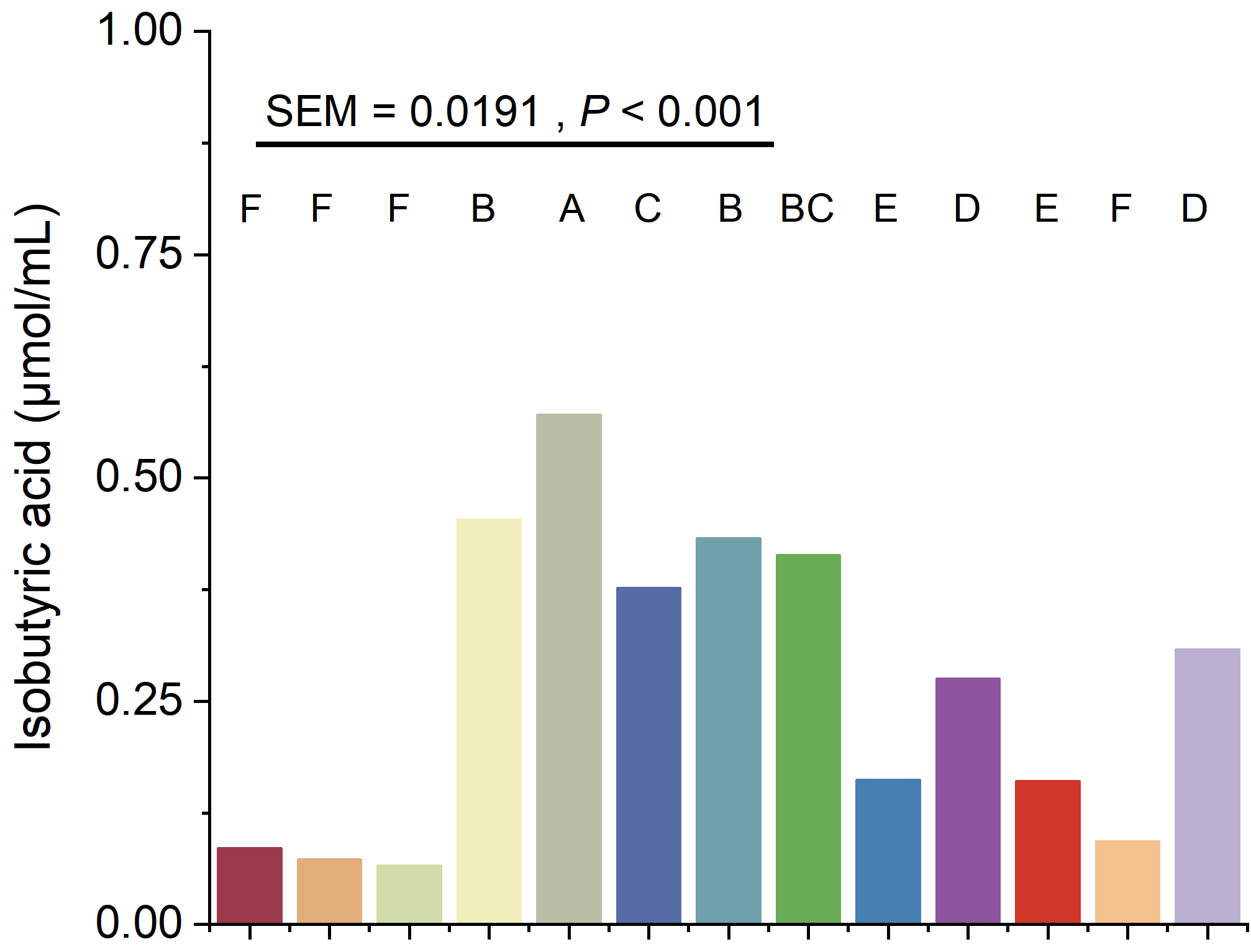

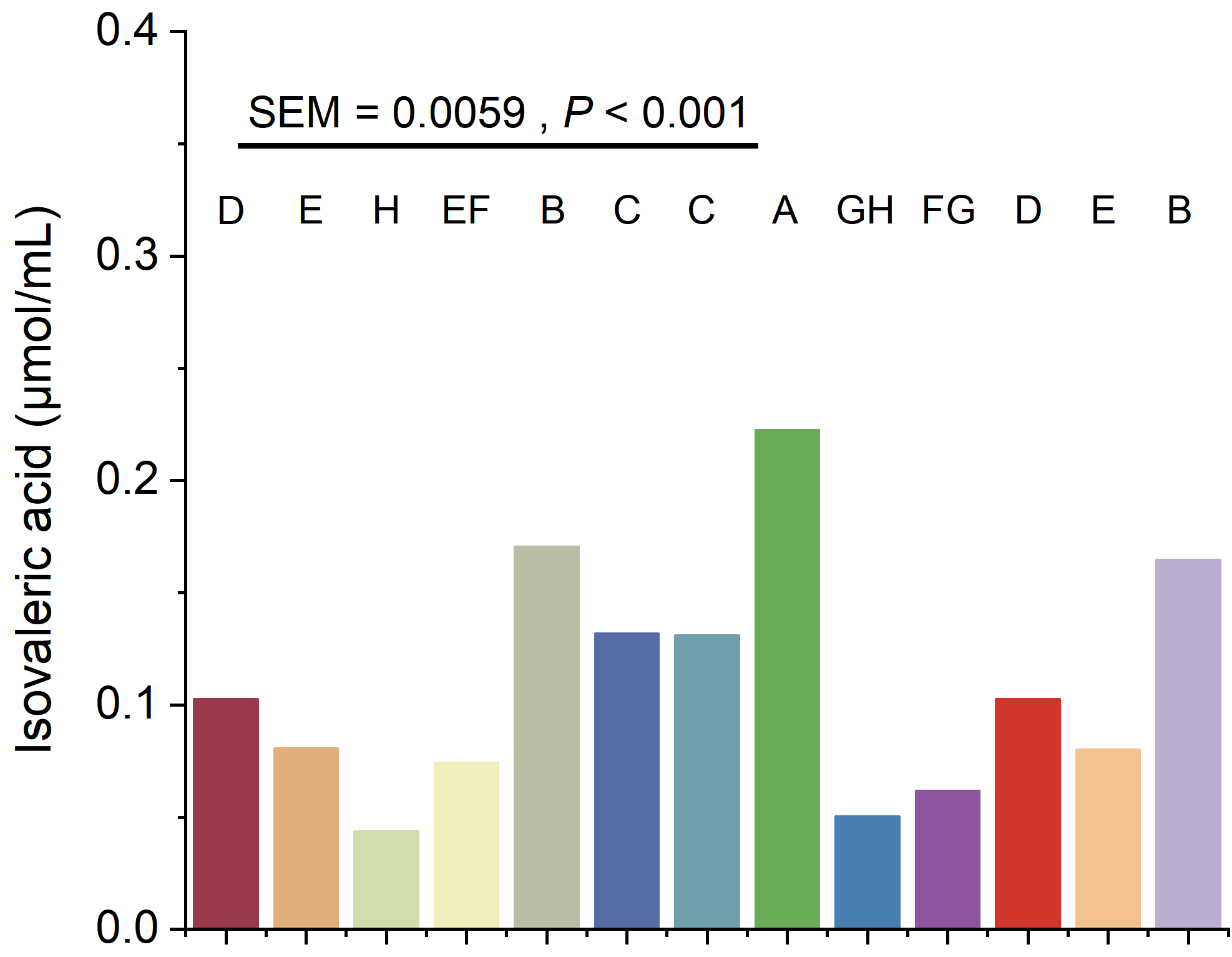


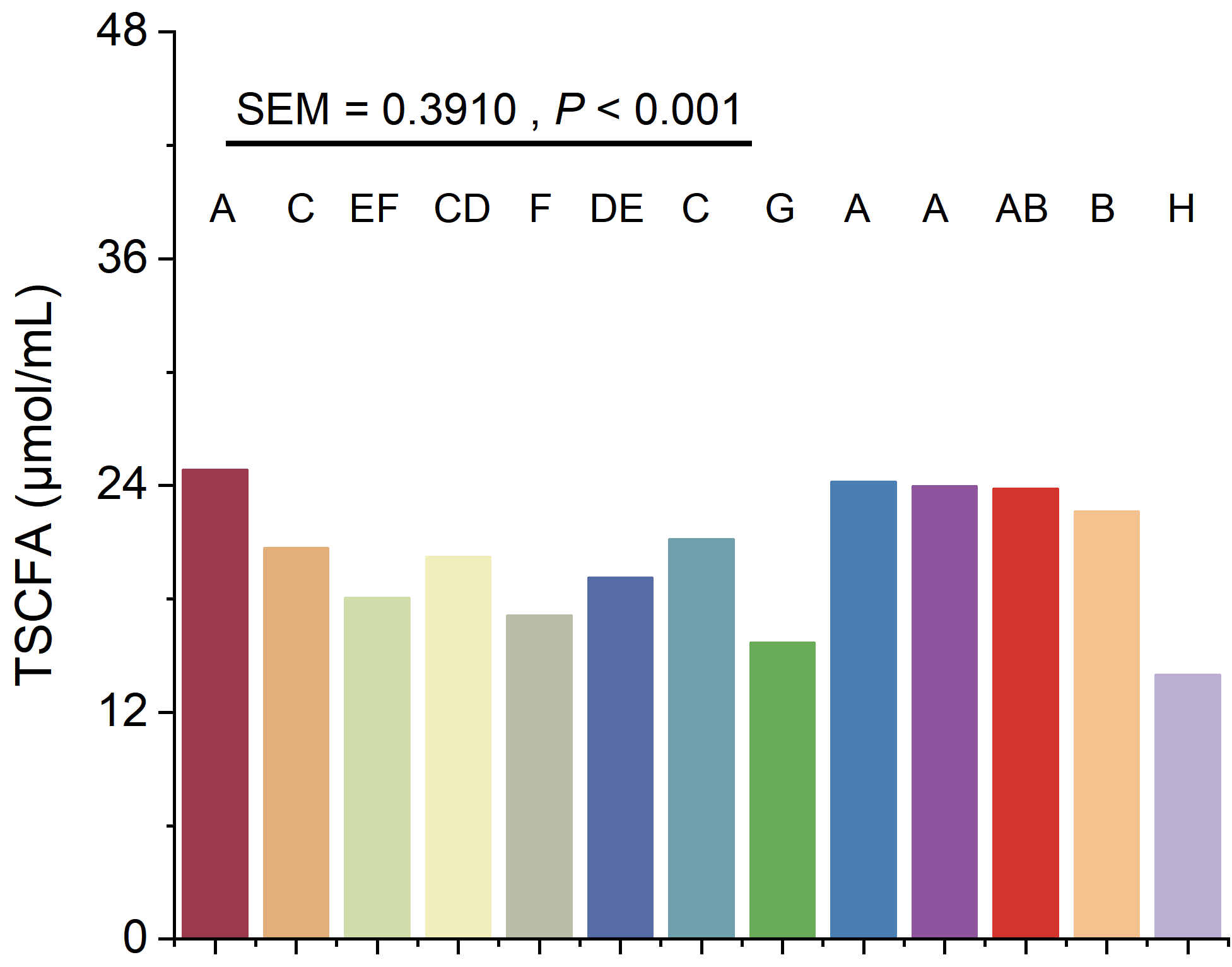

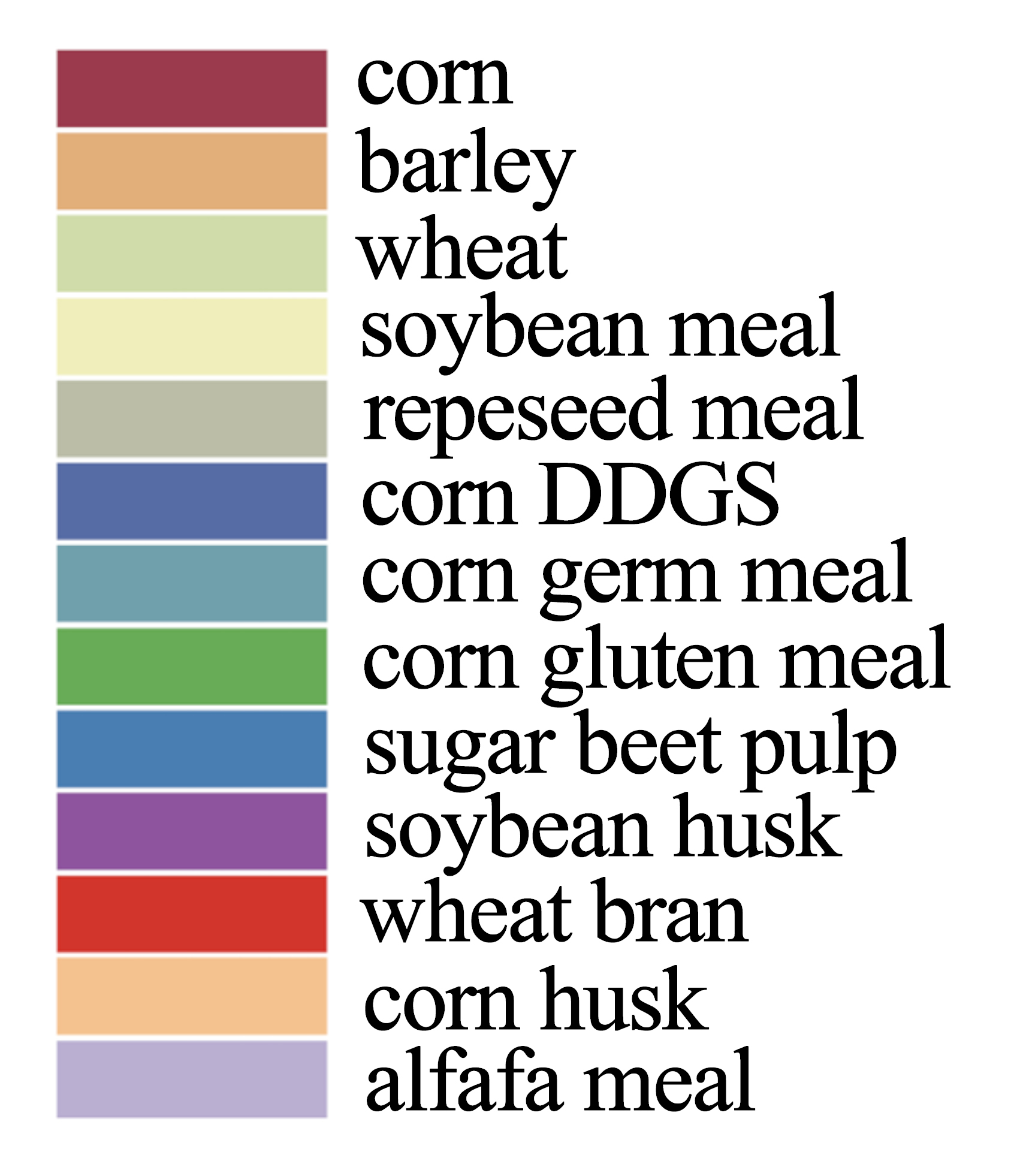


J


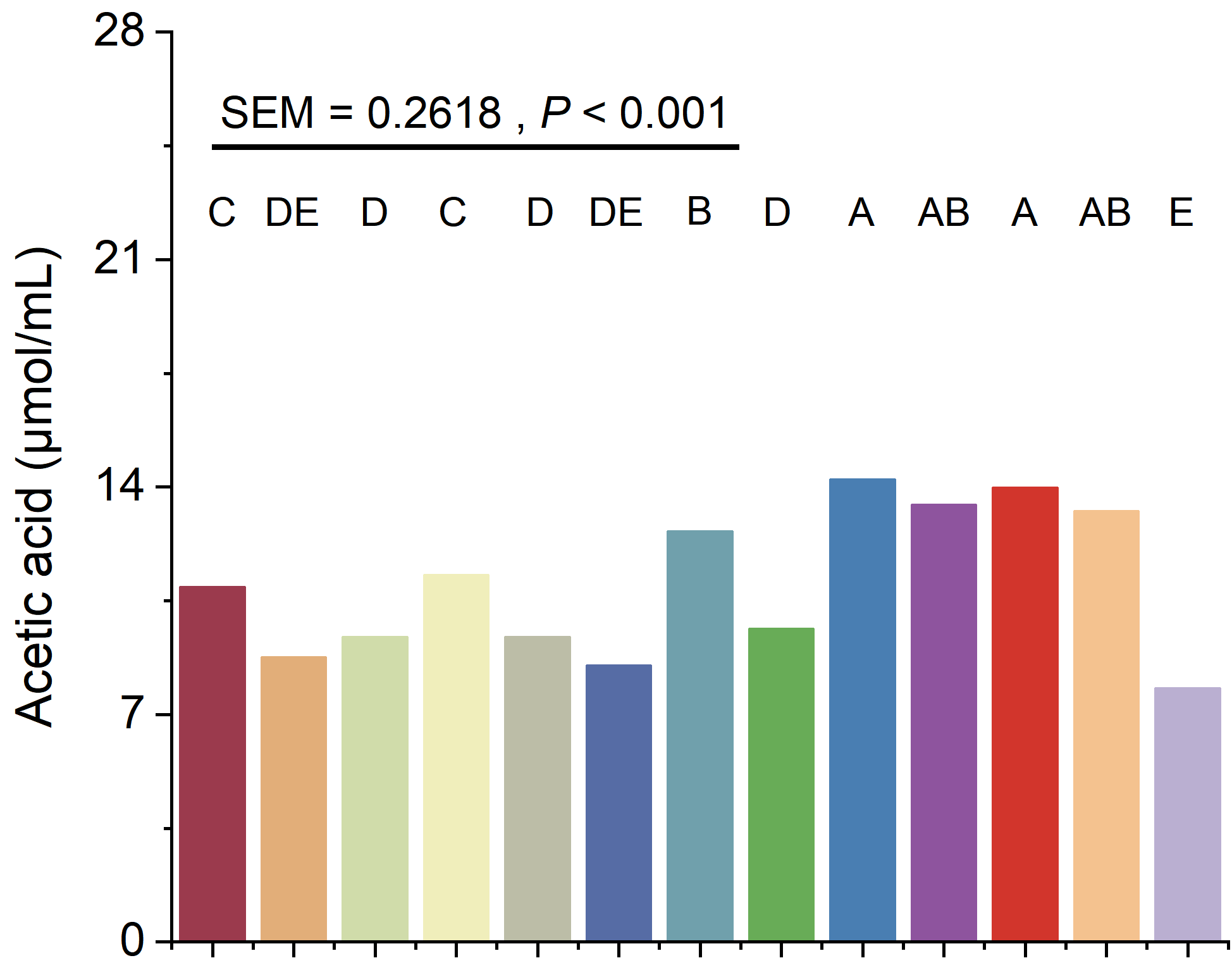

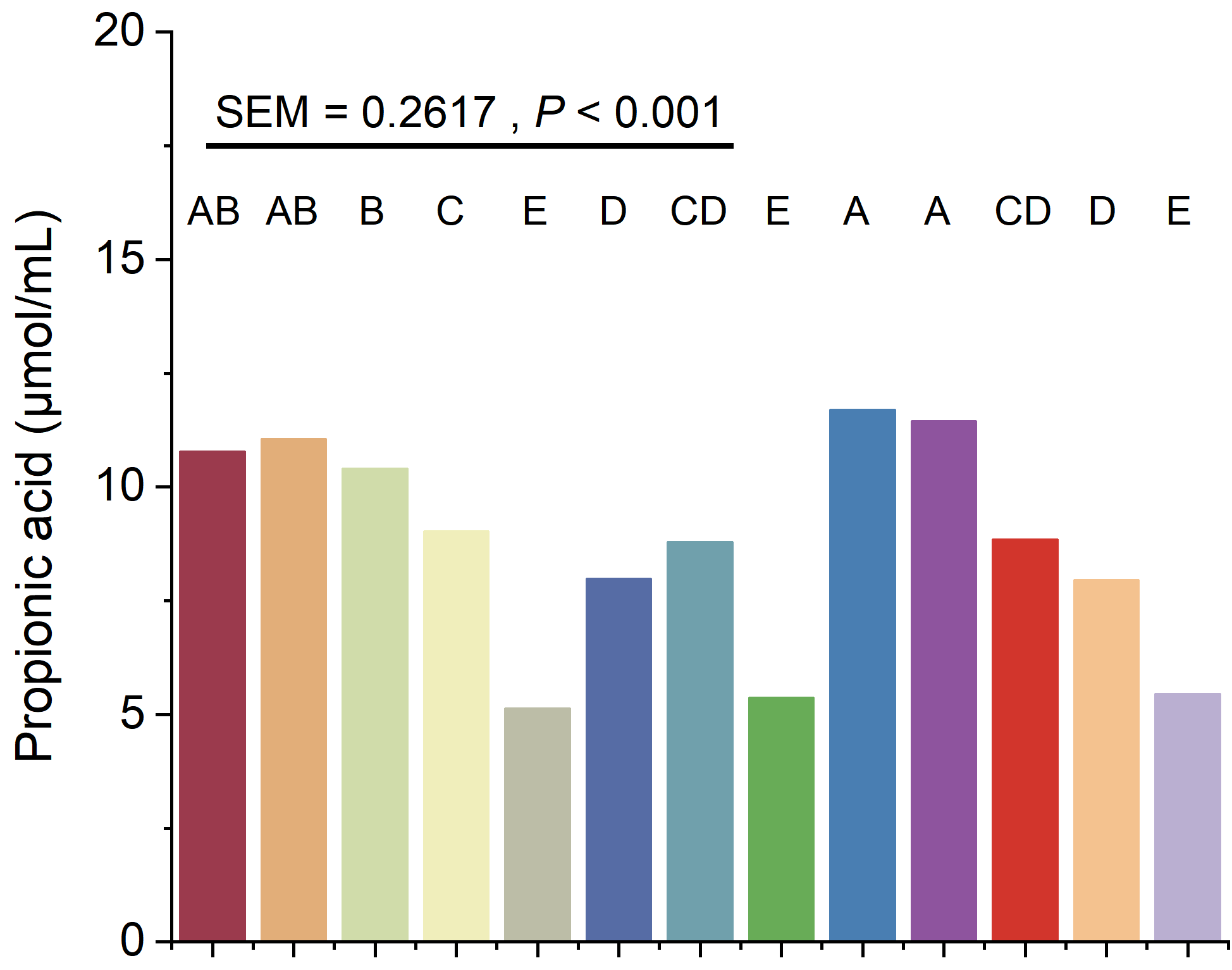

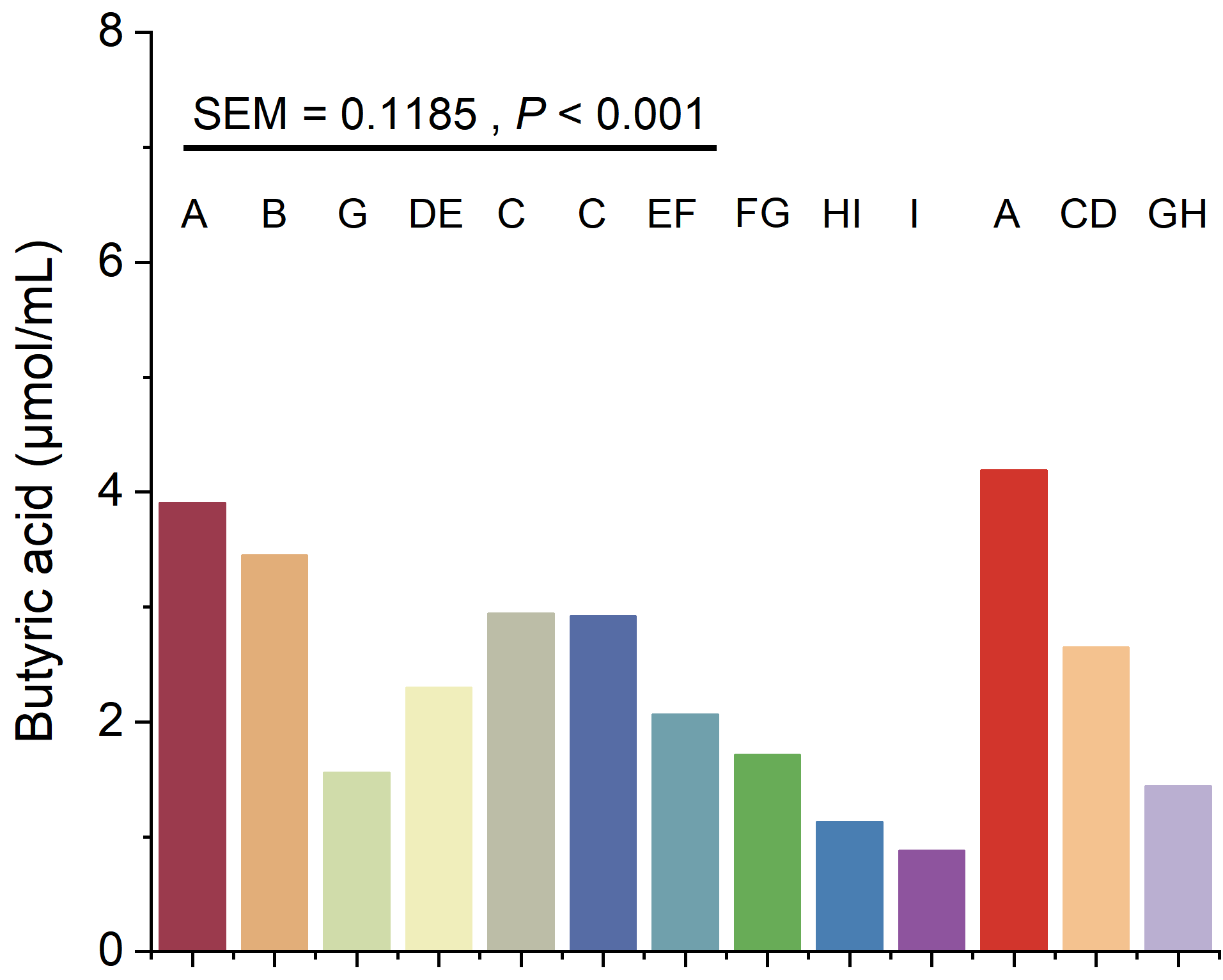


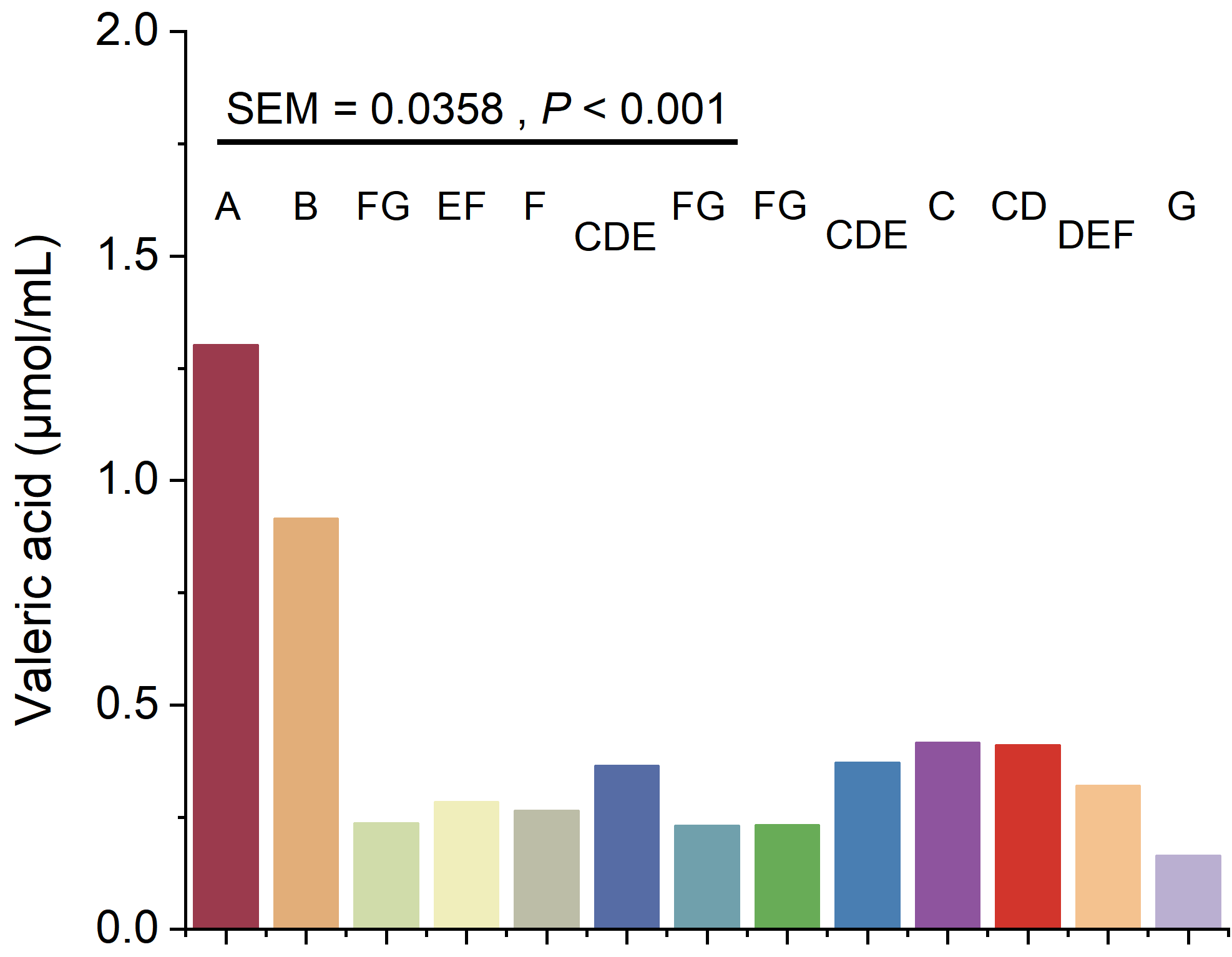

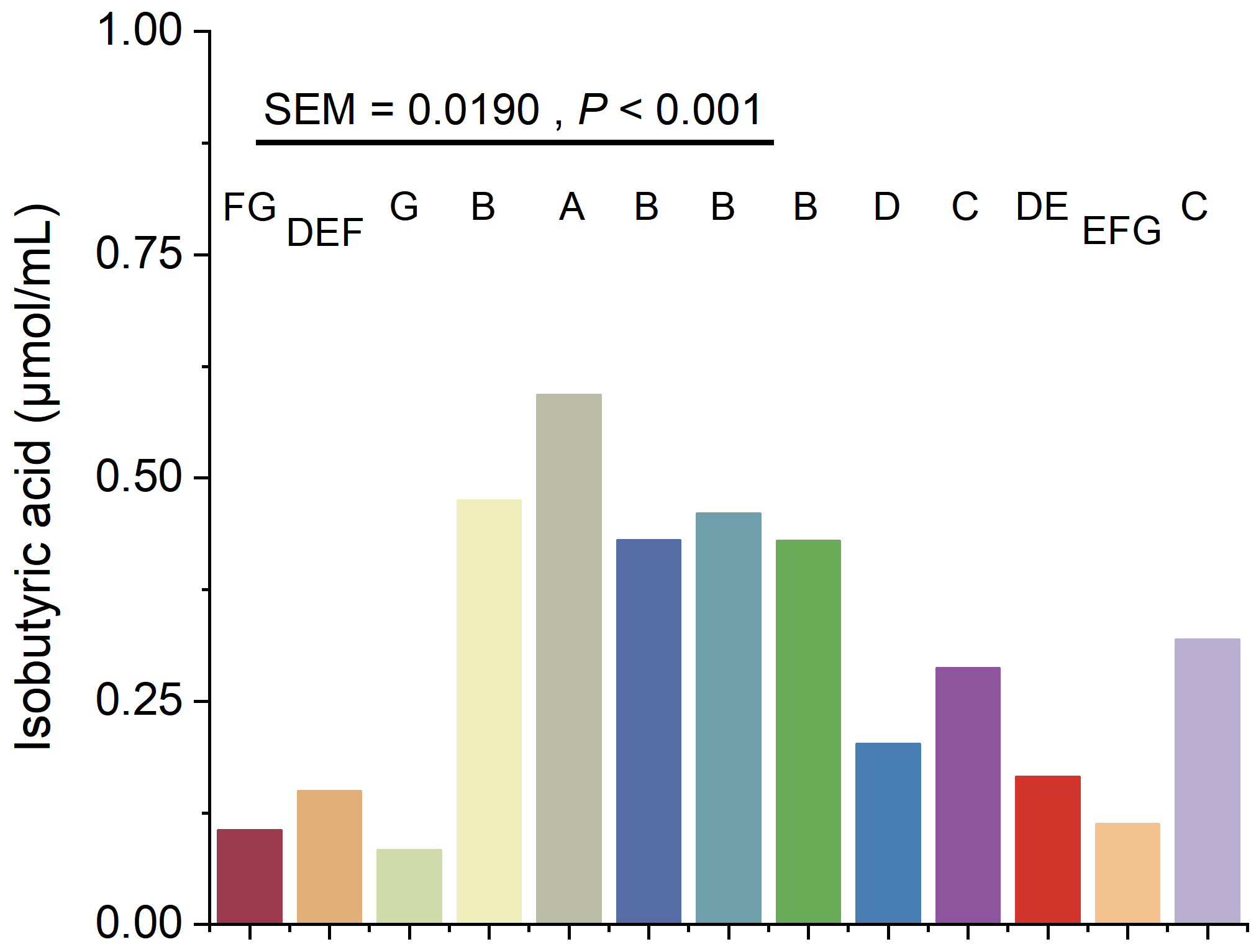

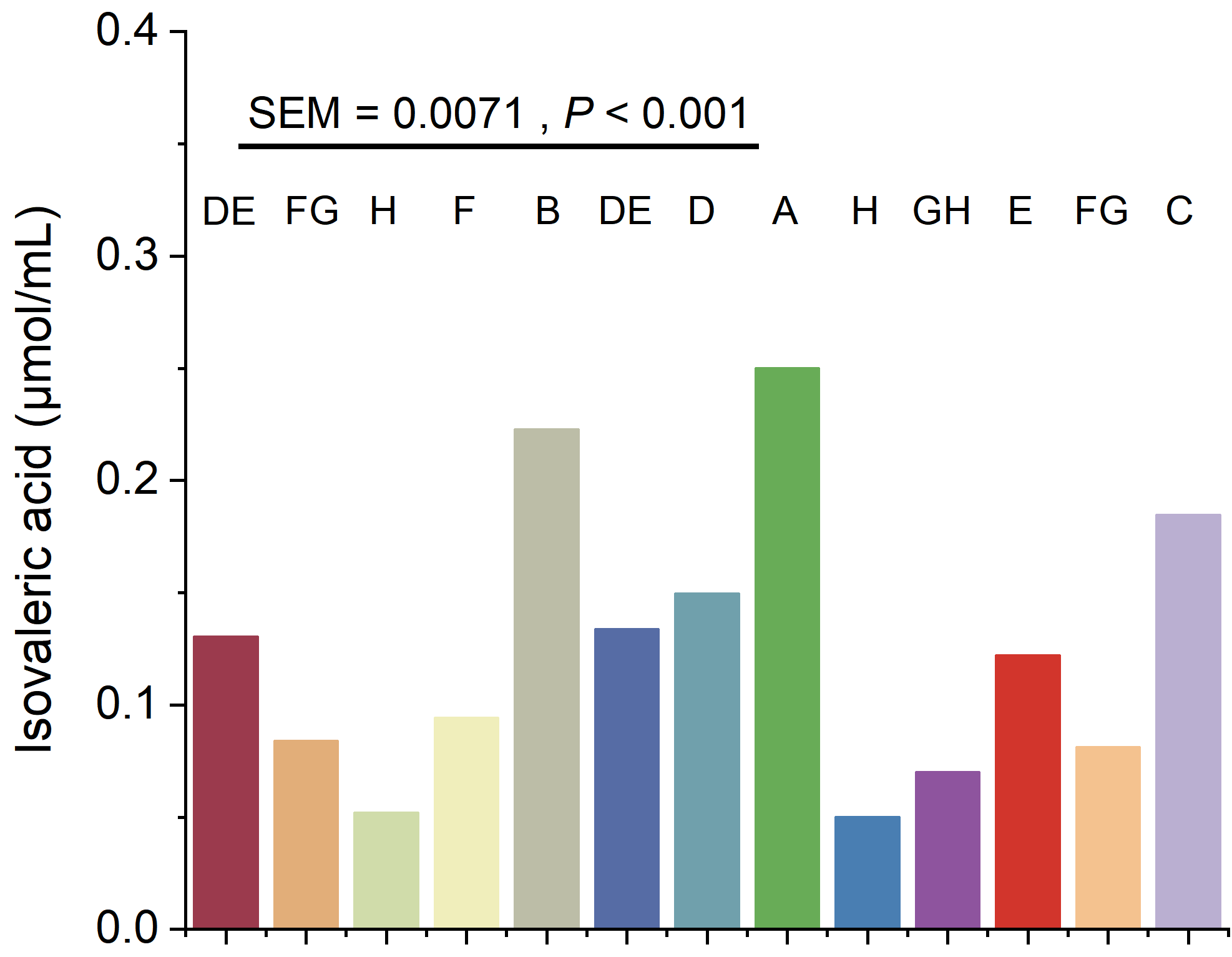


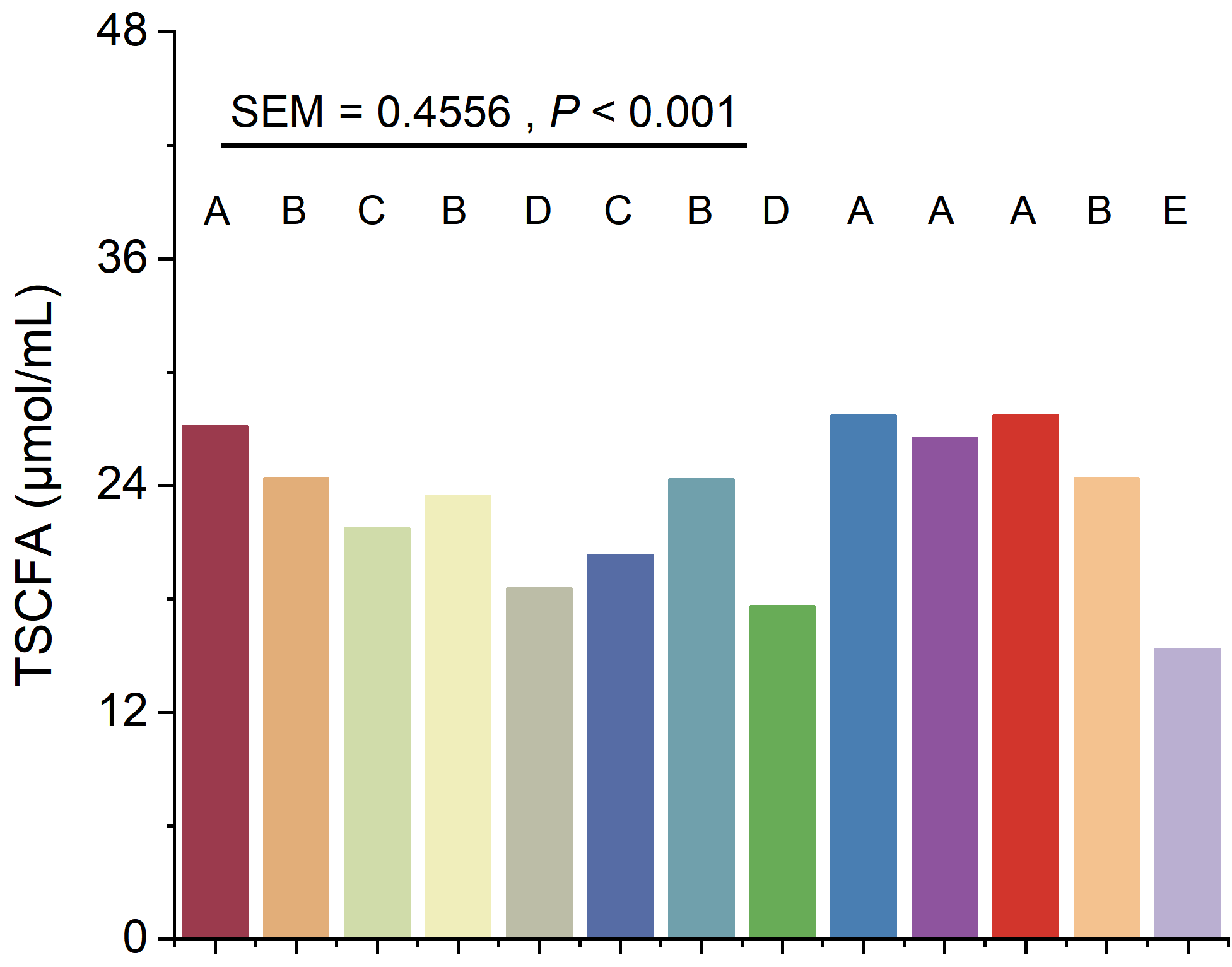

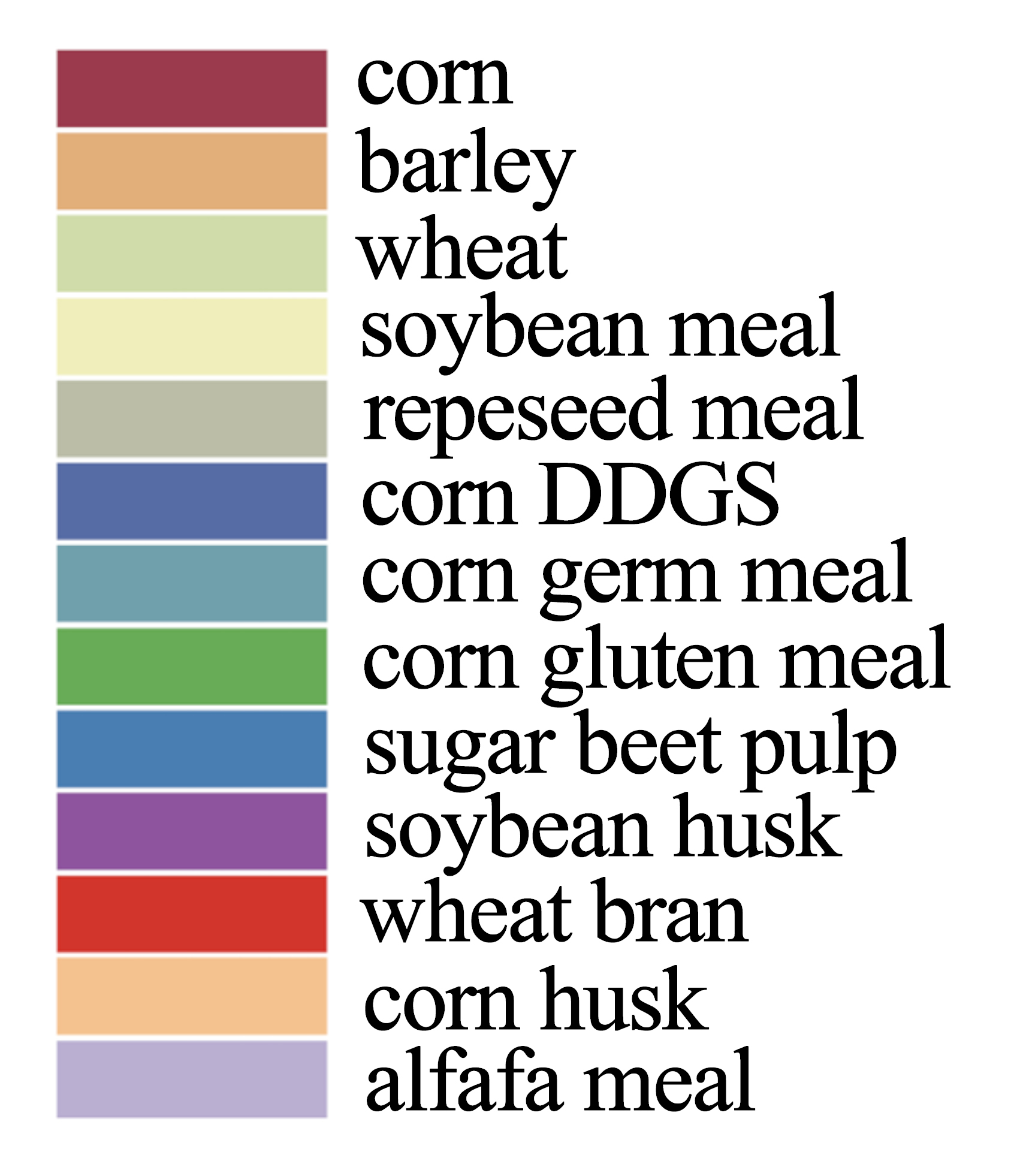


K


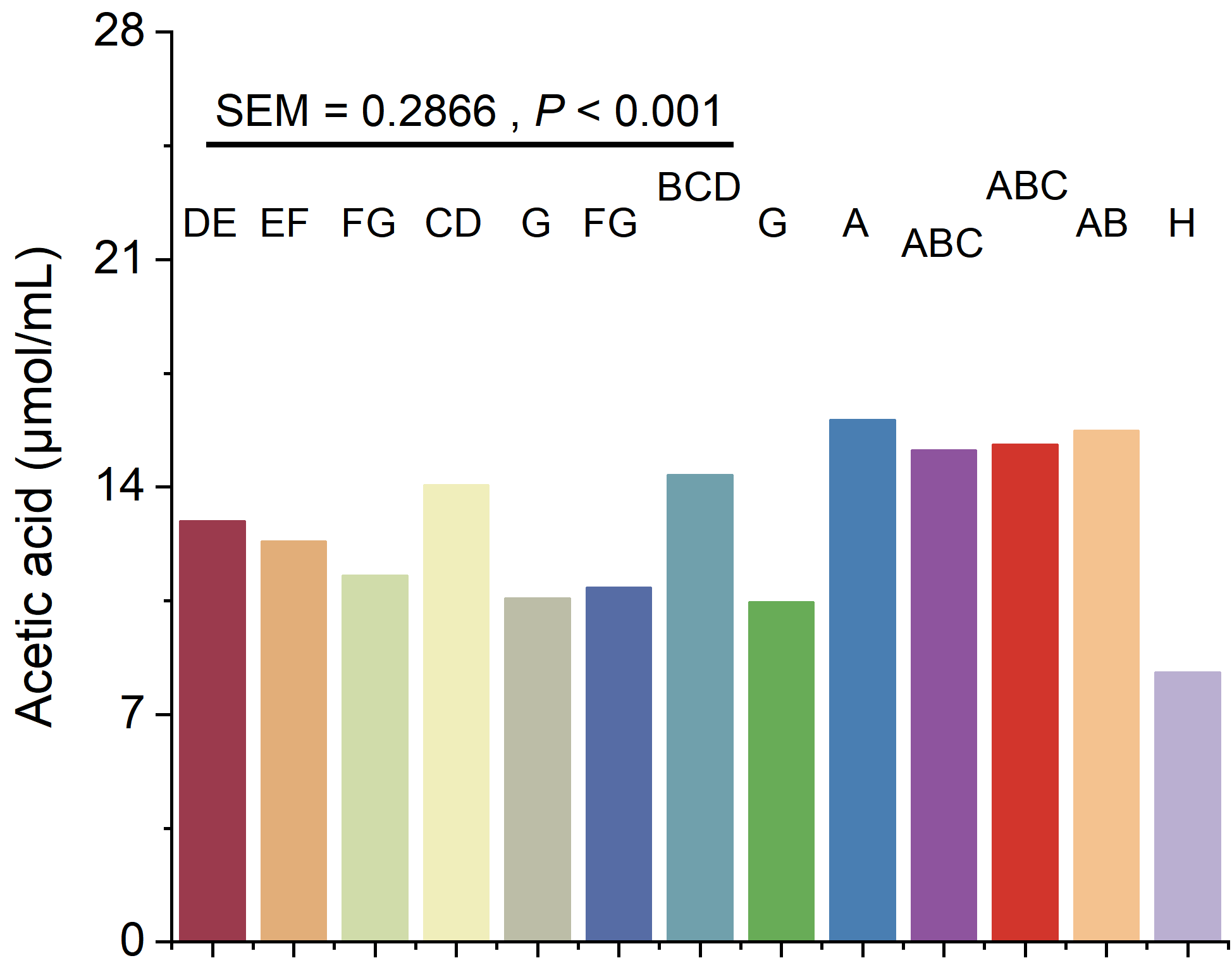

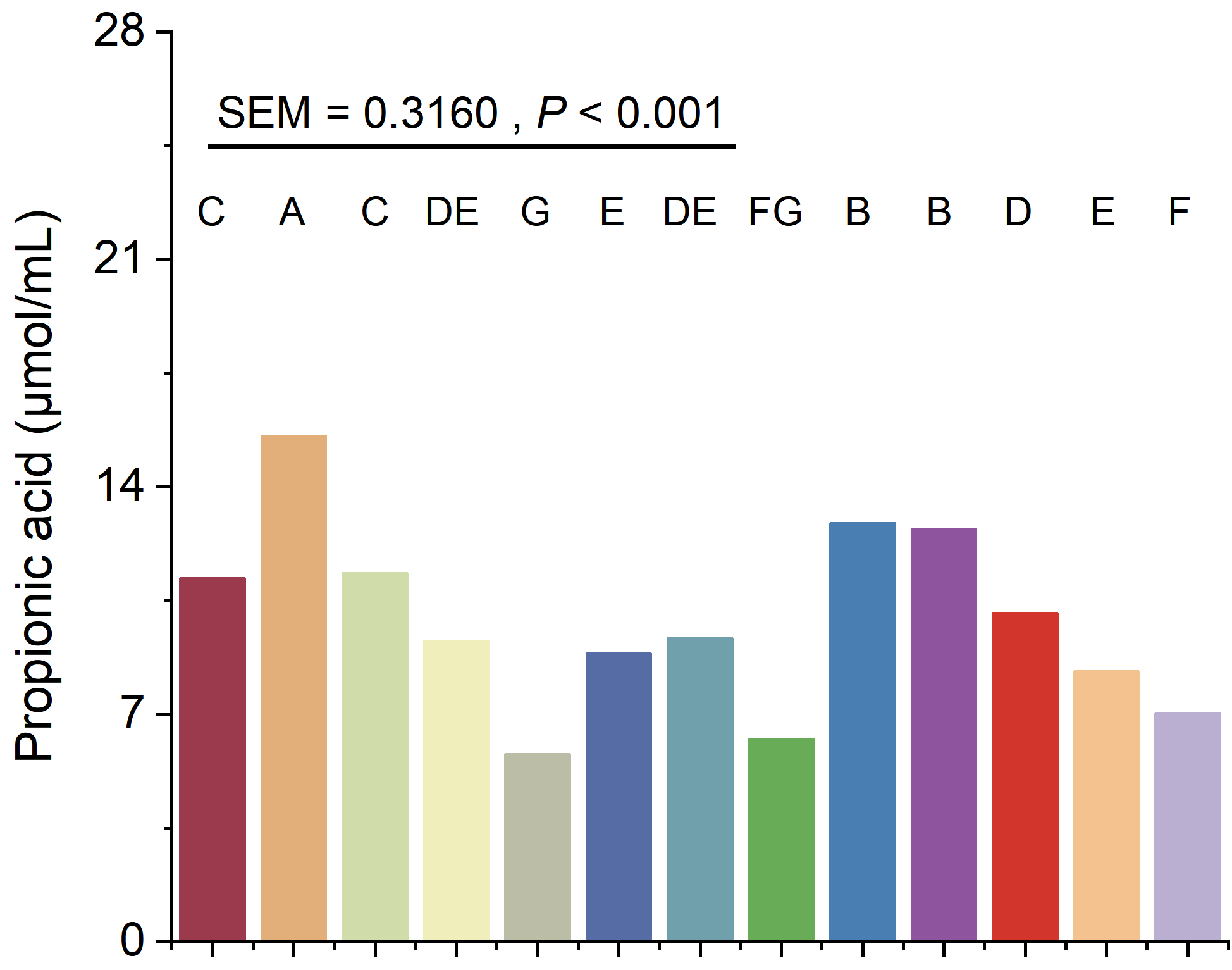

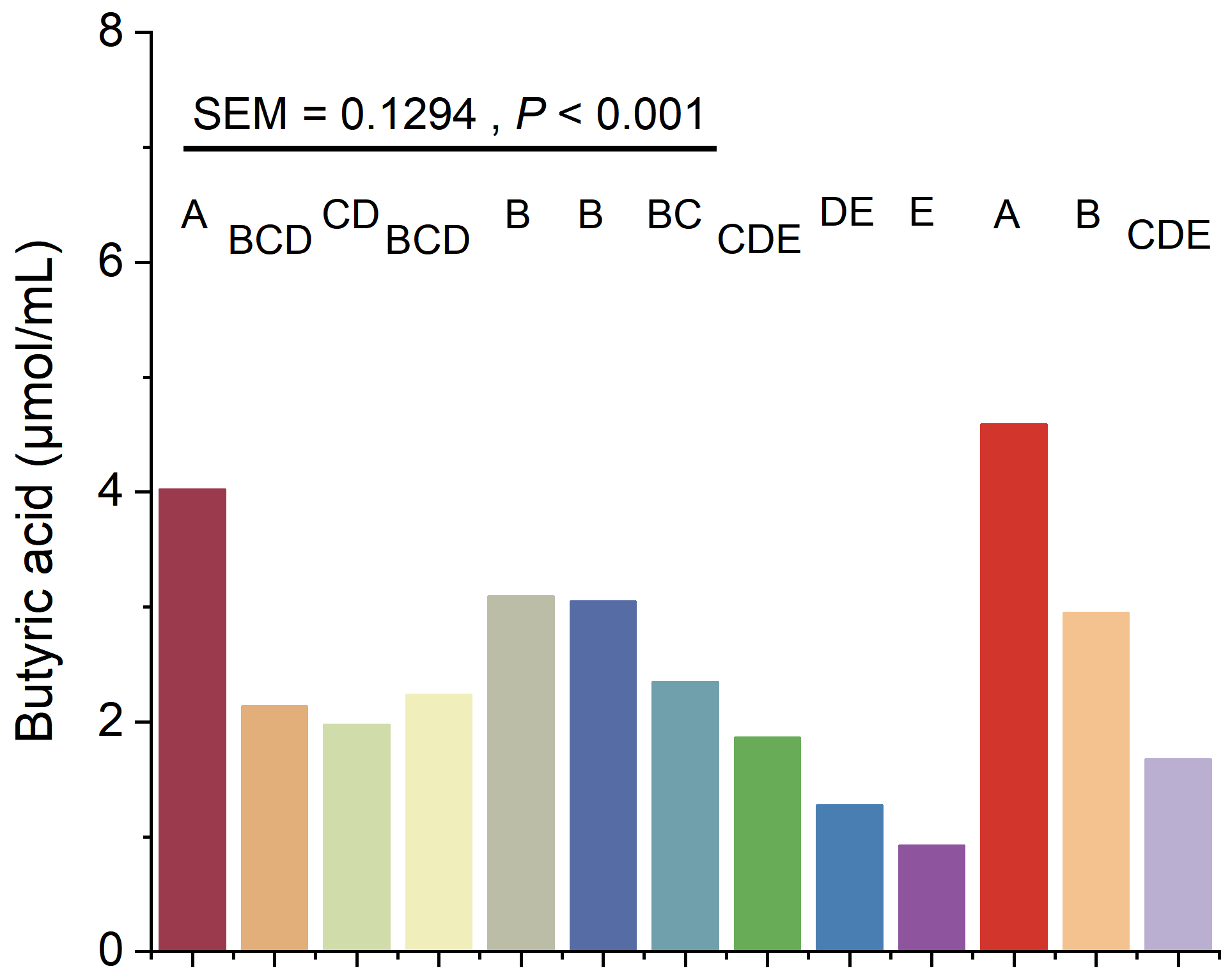


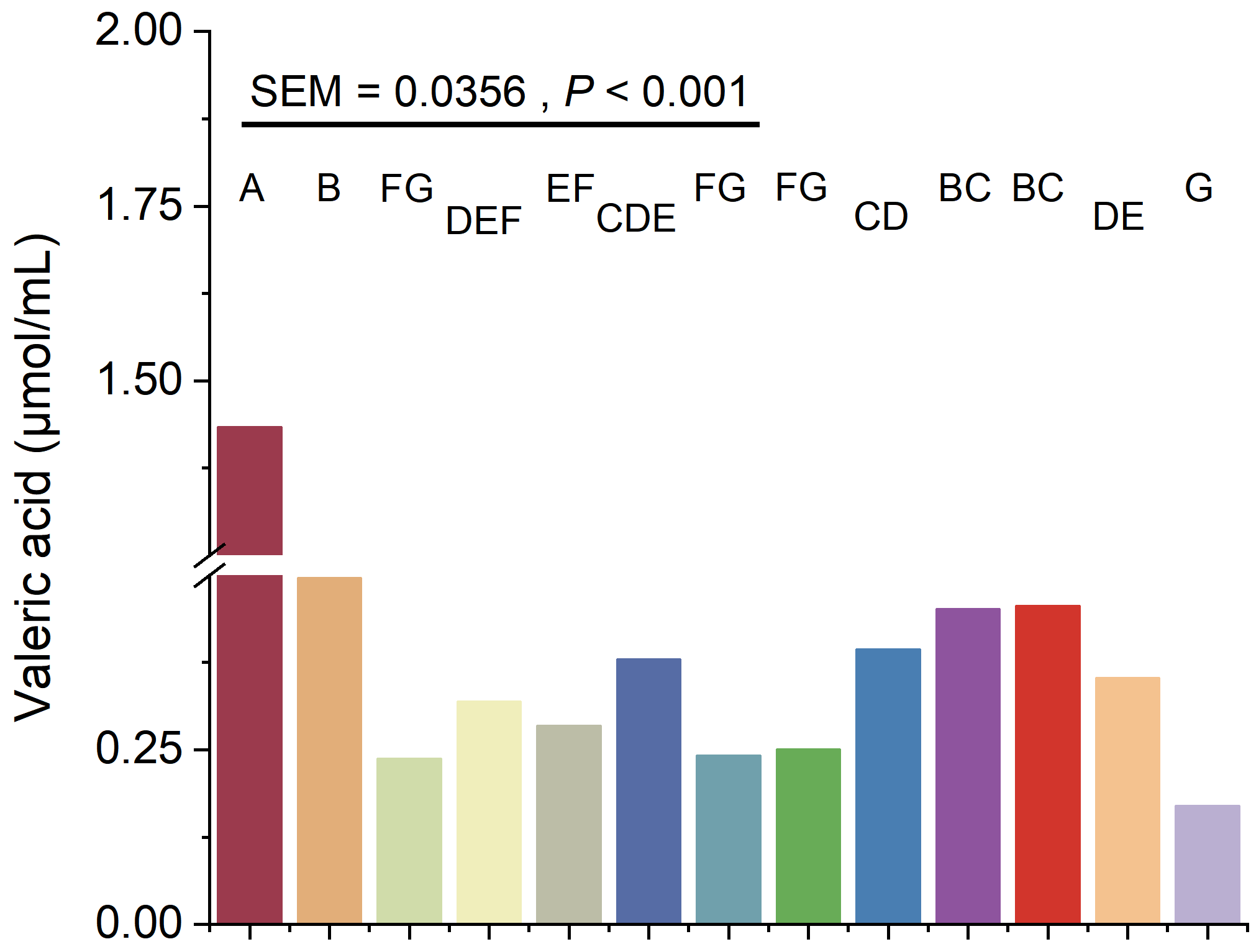

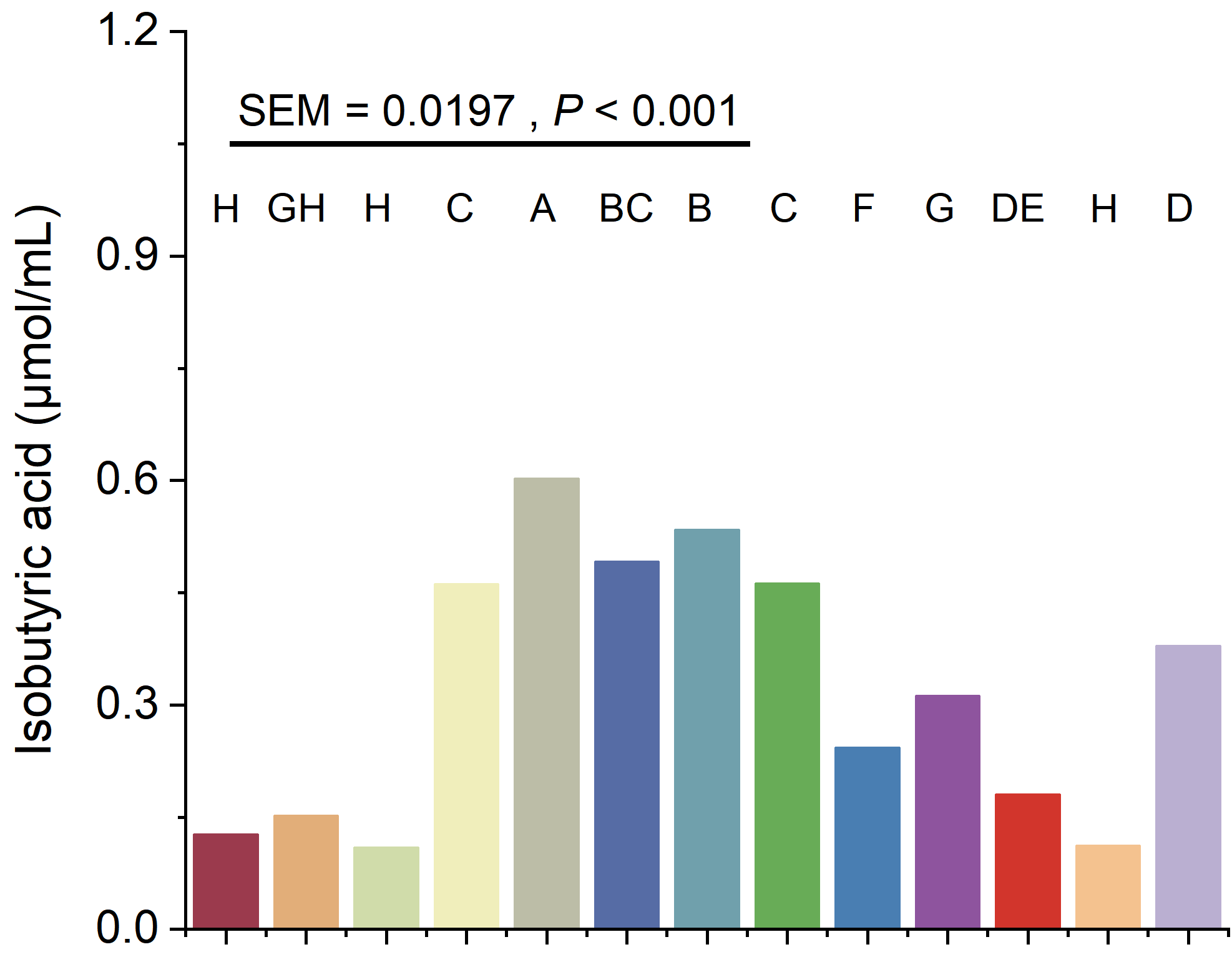

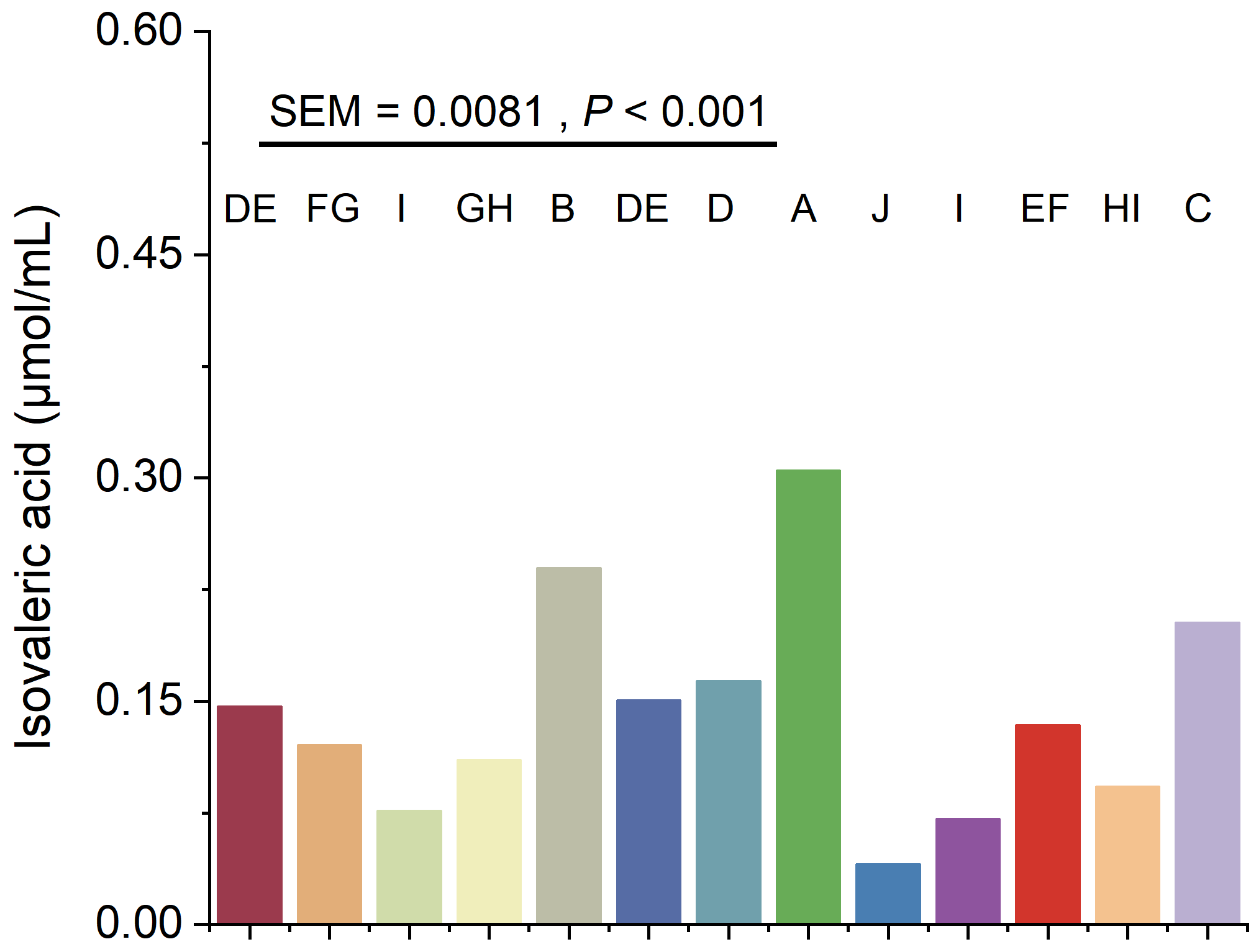


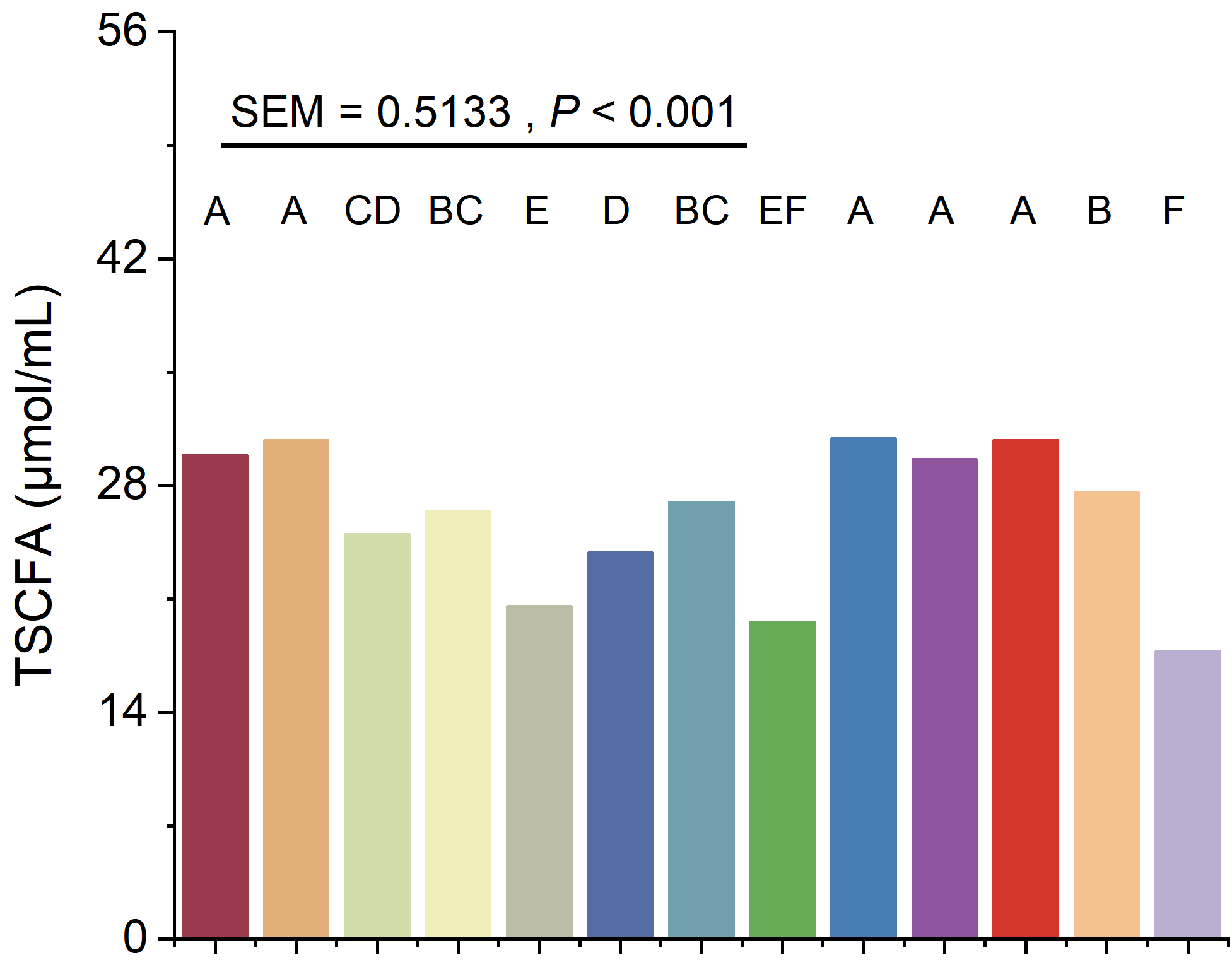

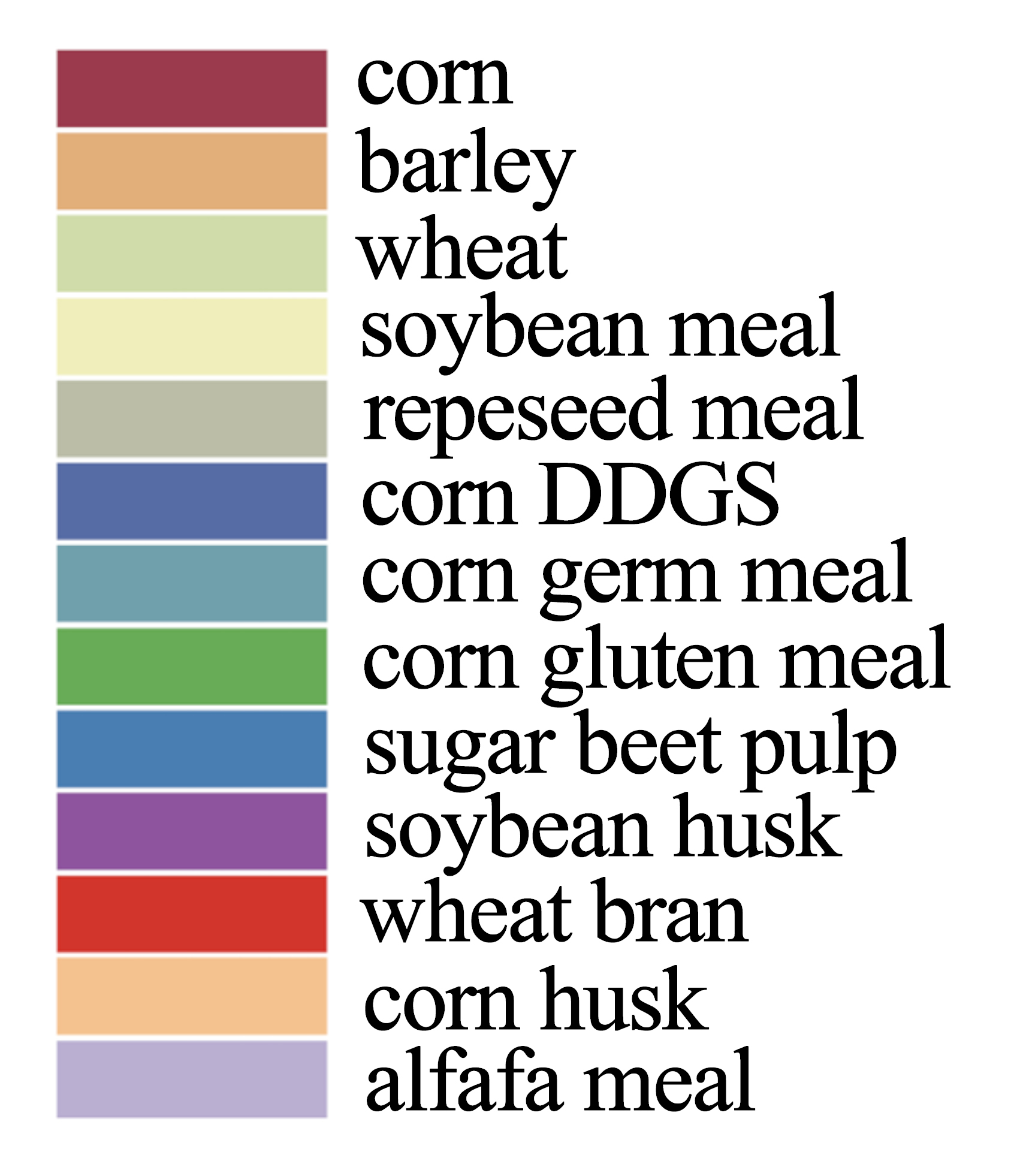


L


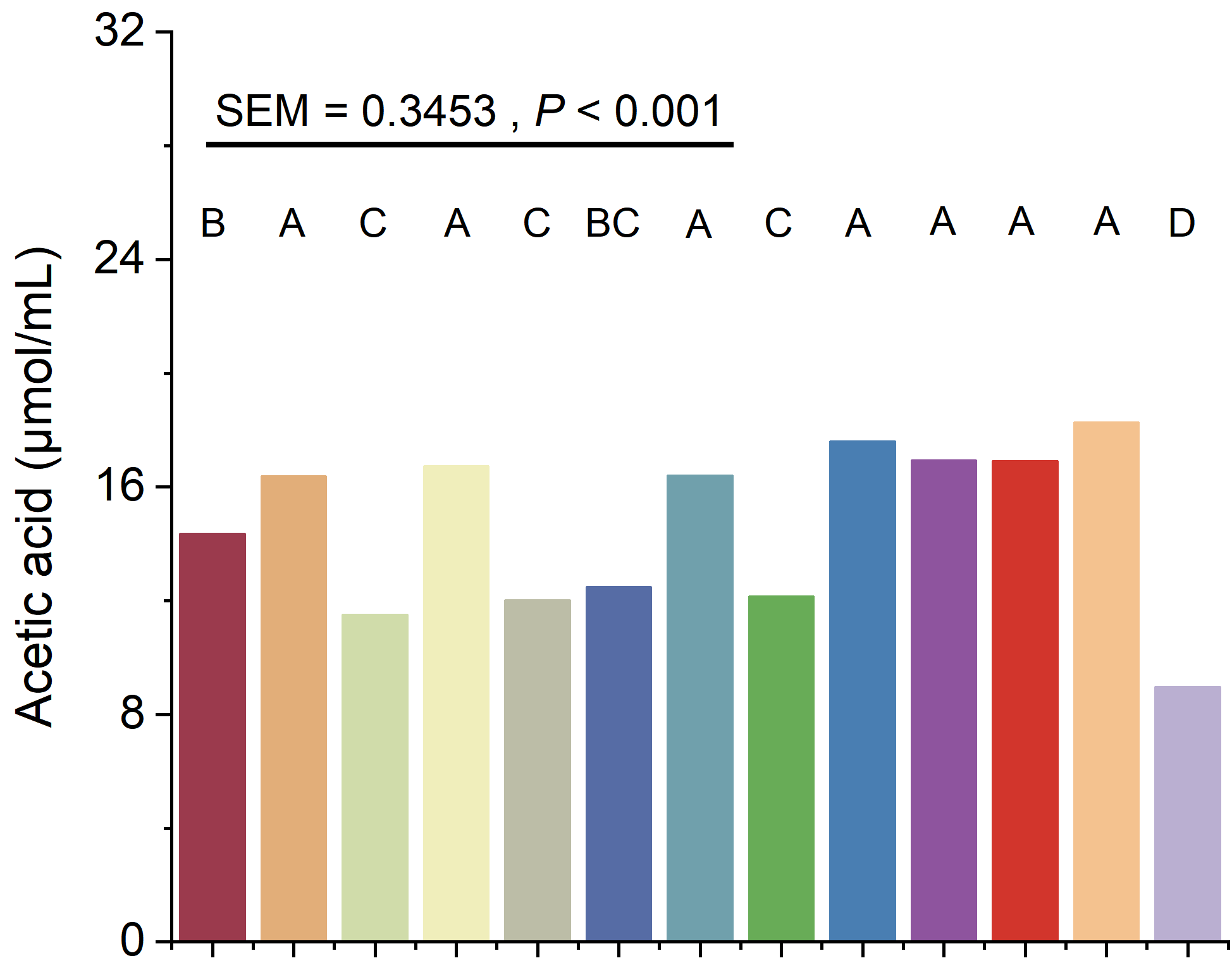

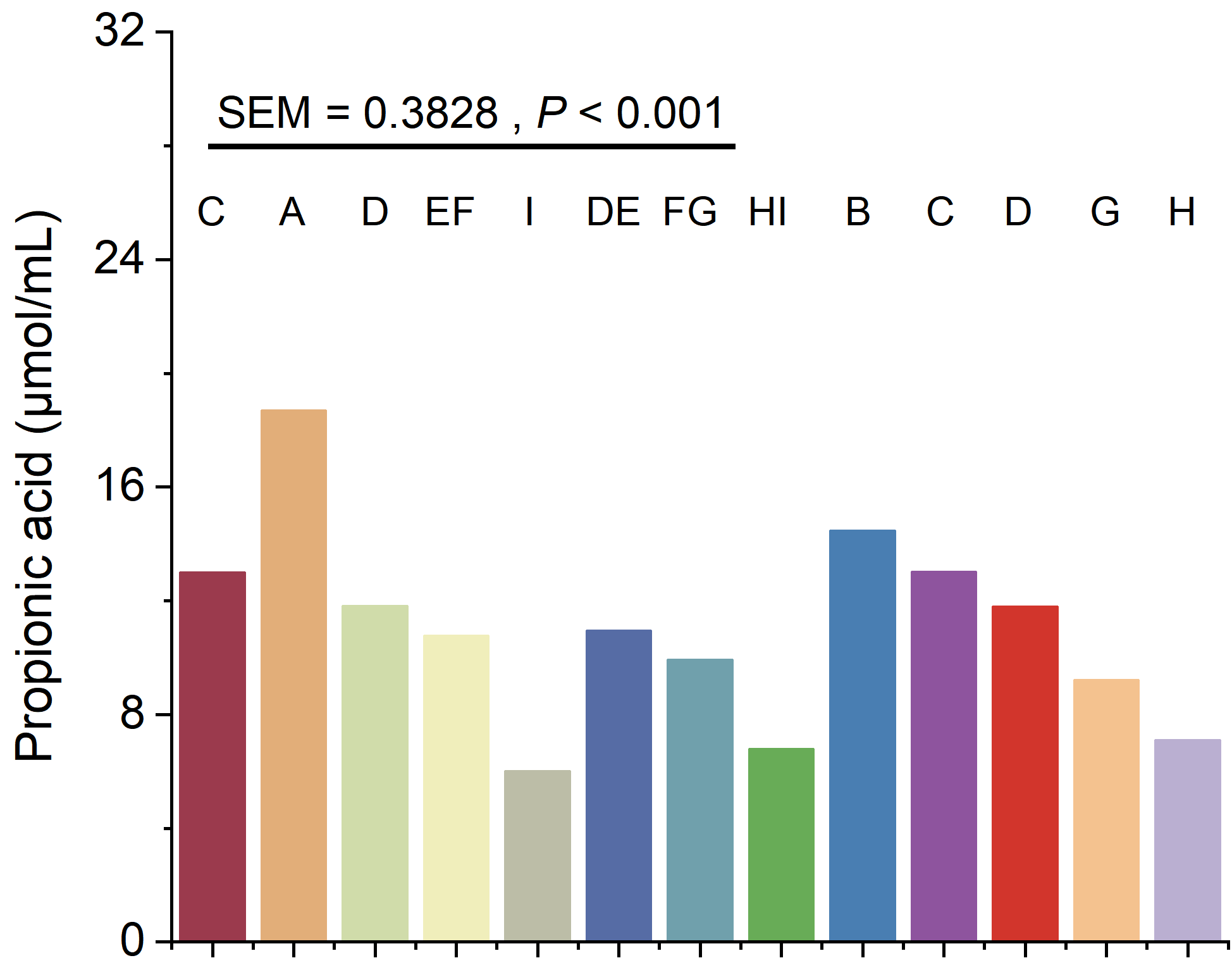

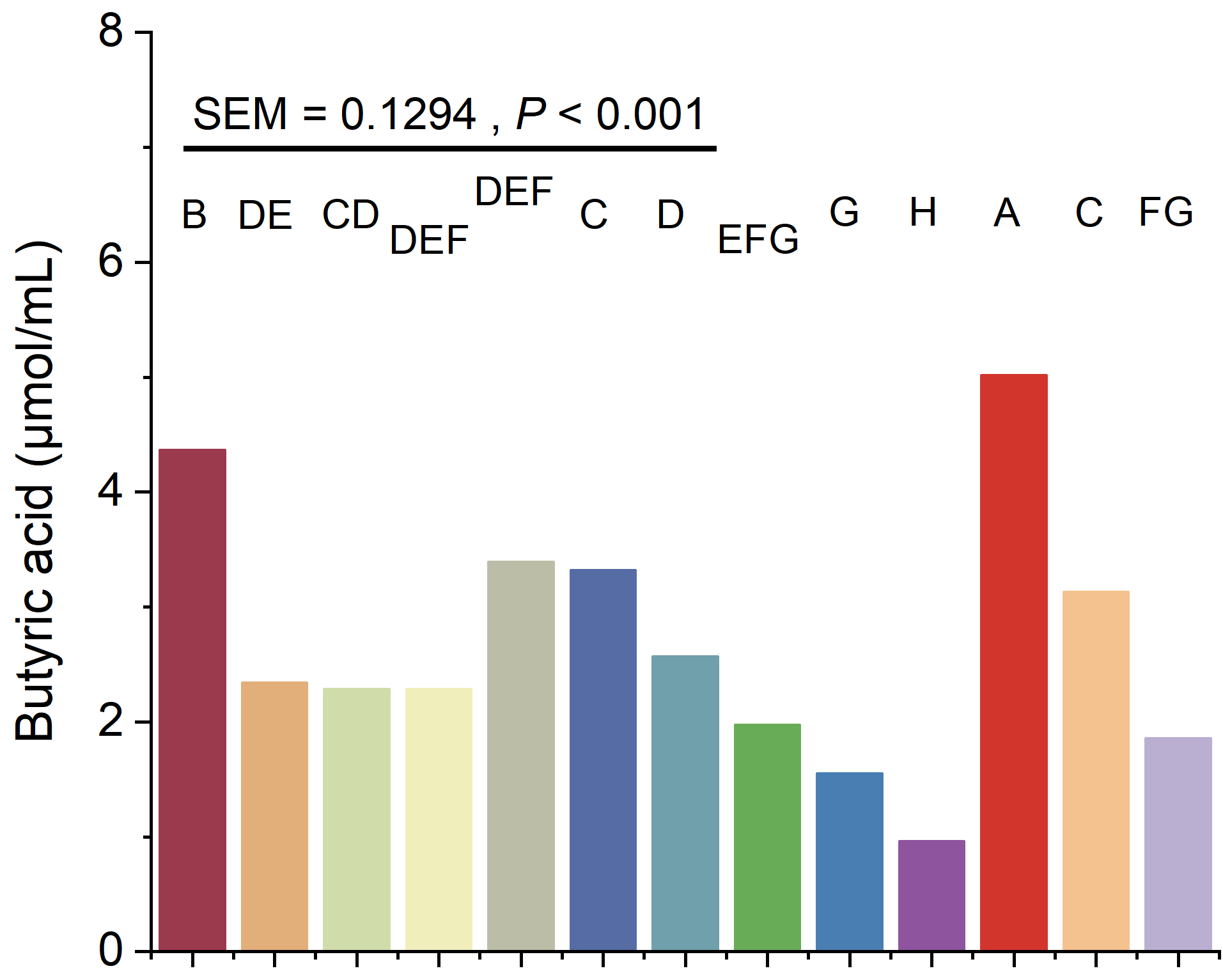


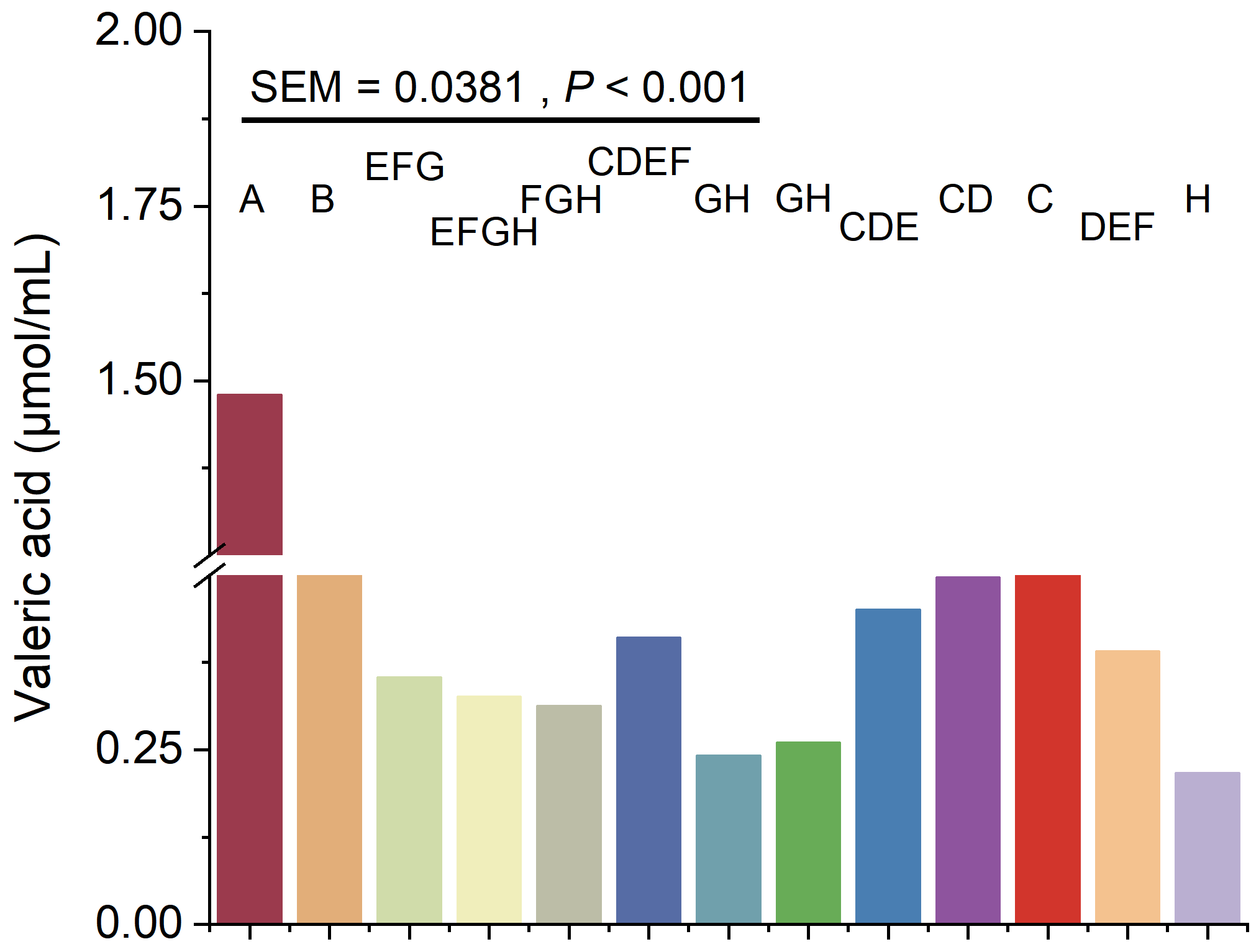

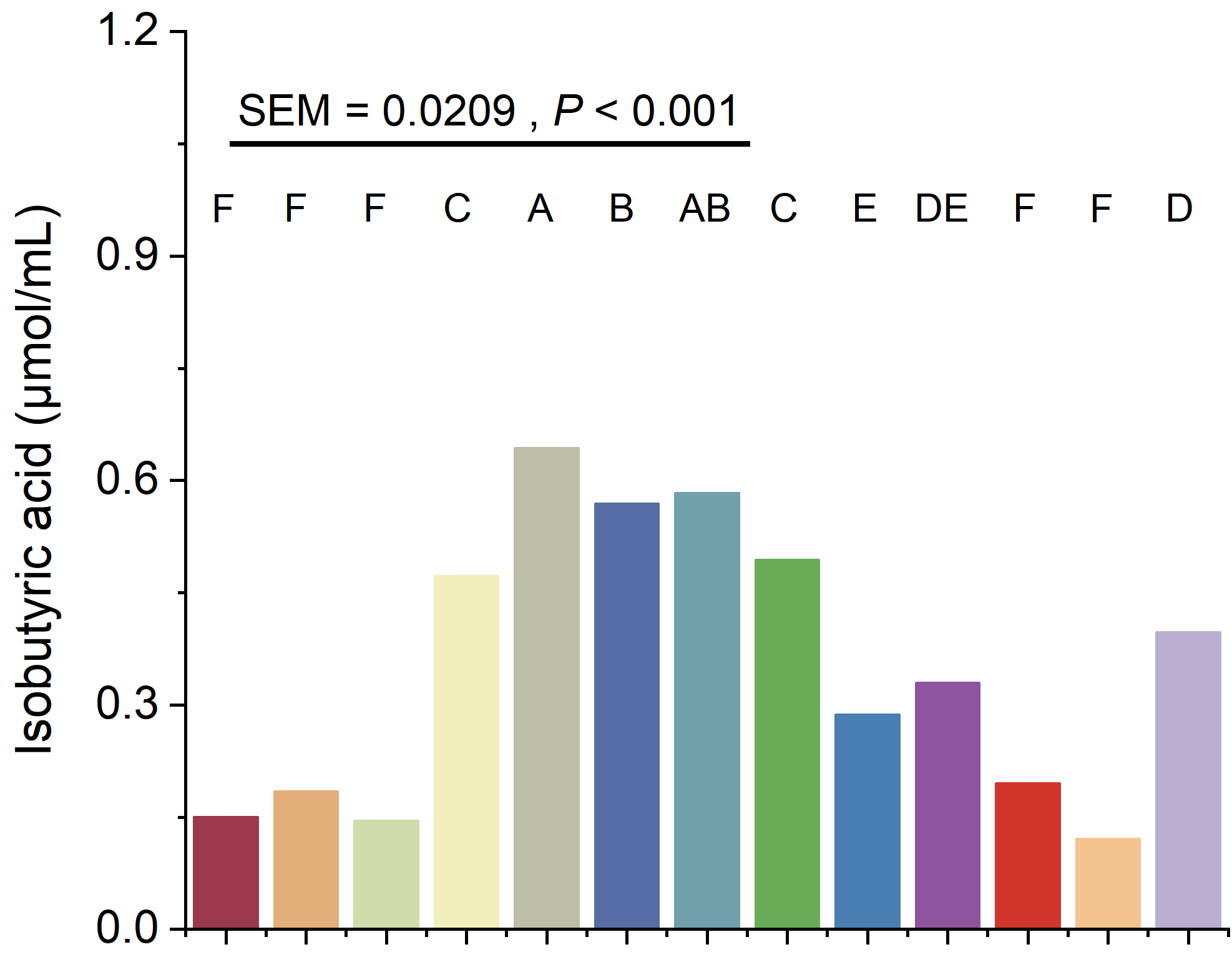

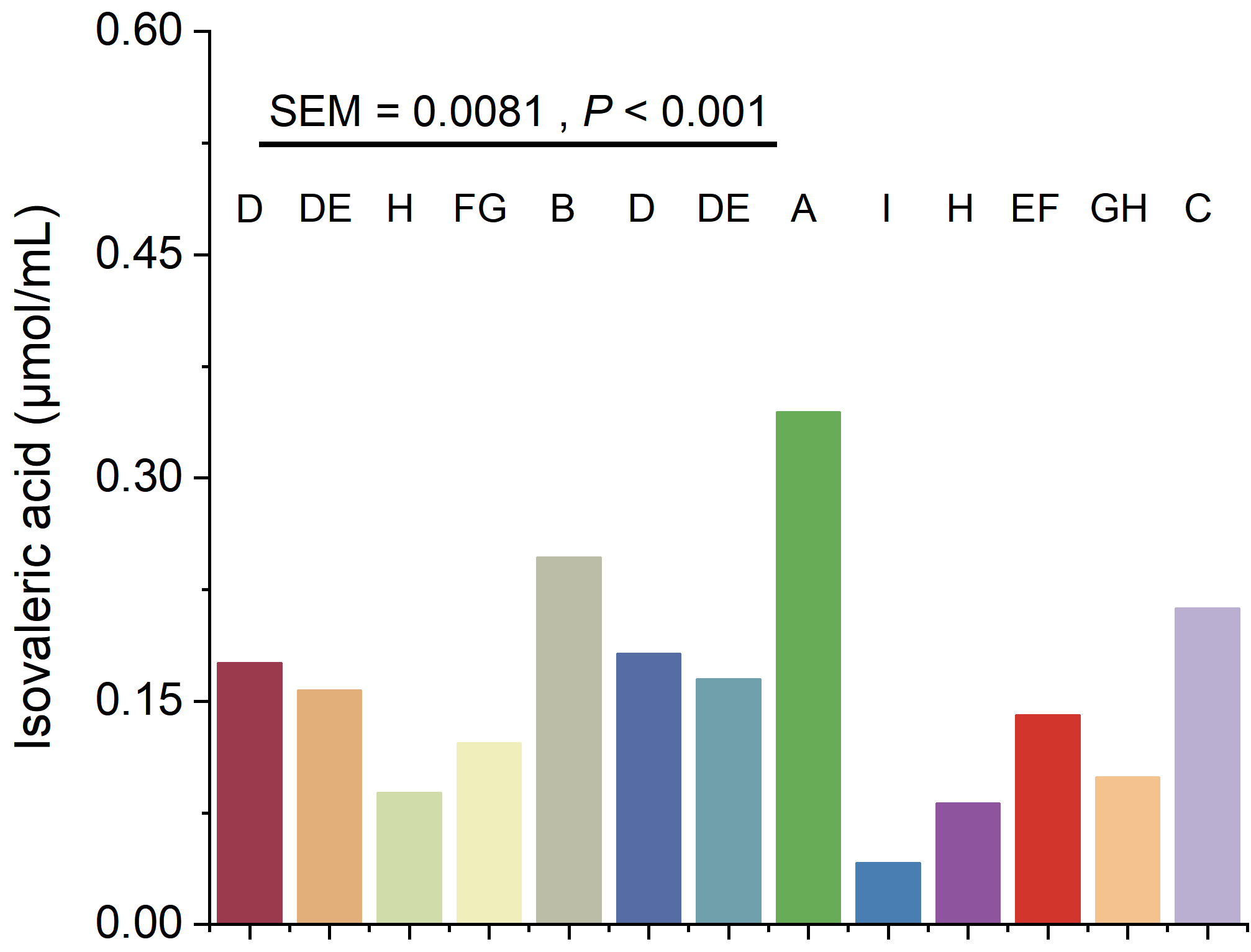


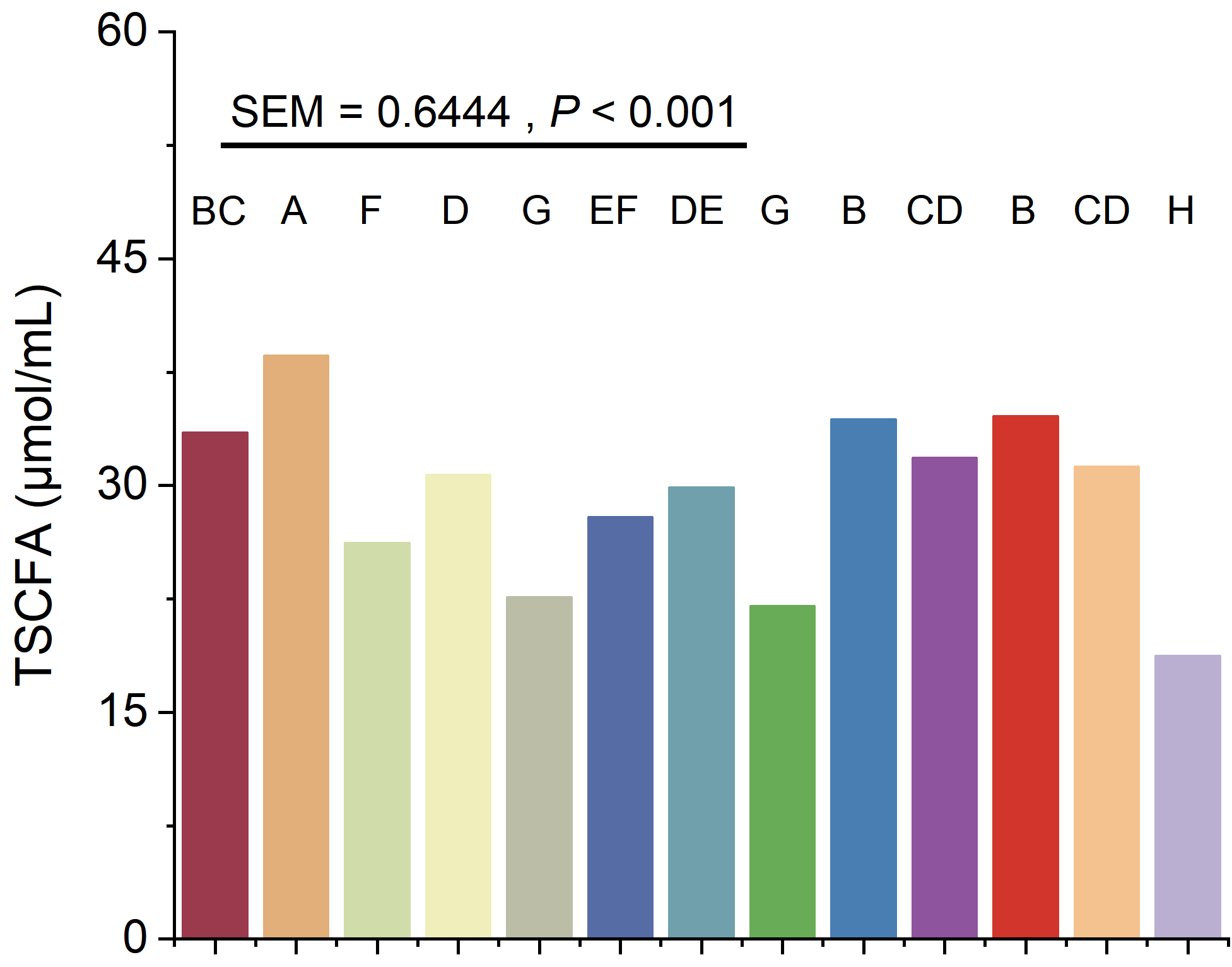

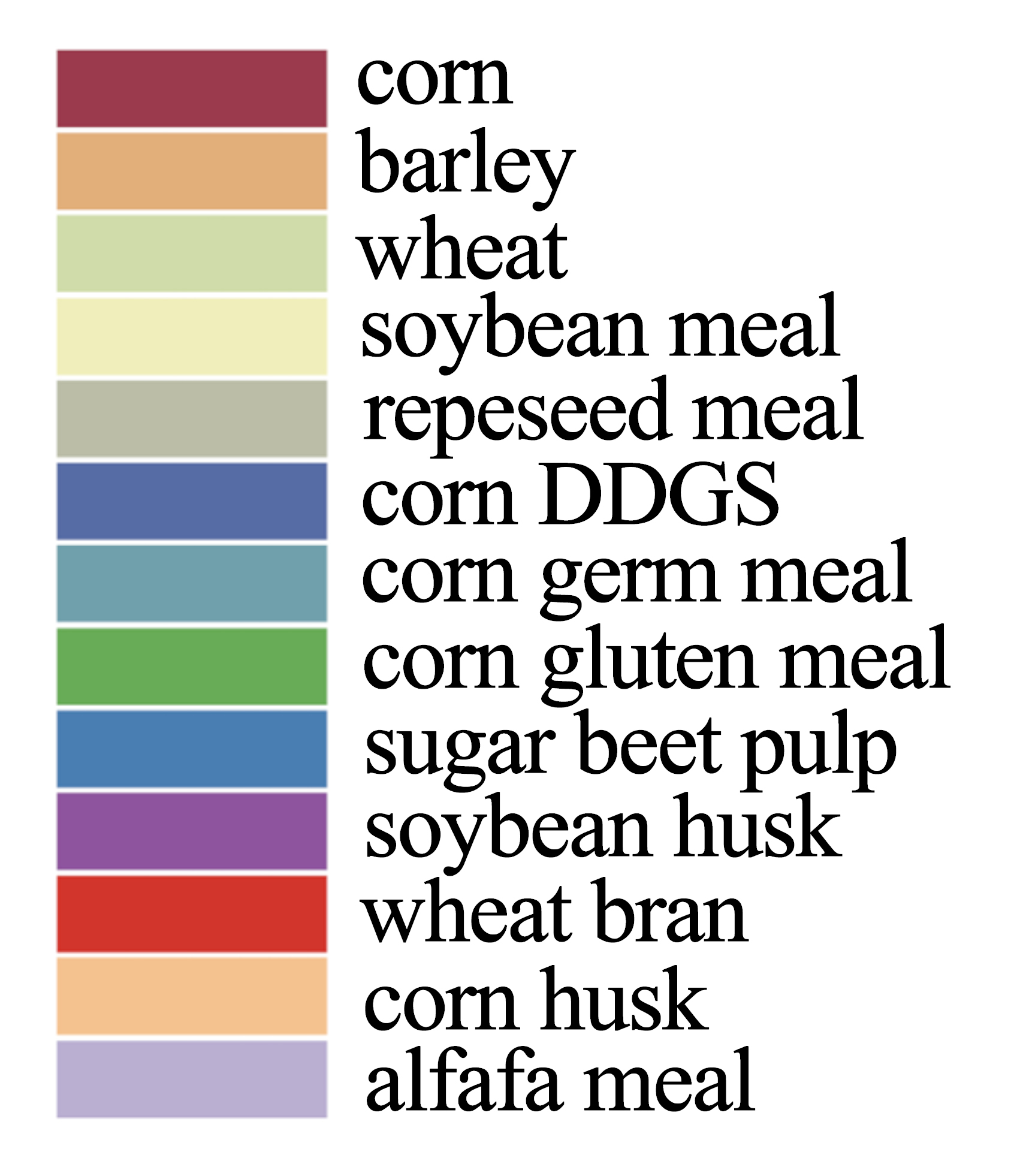


M


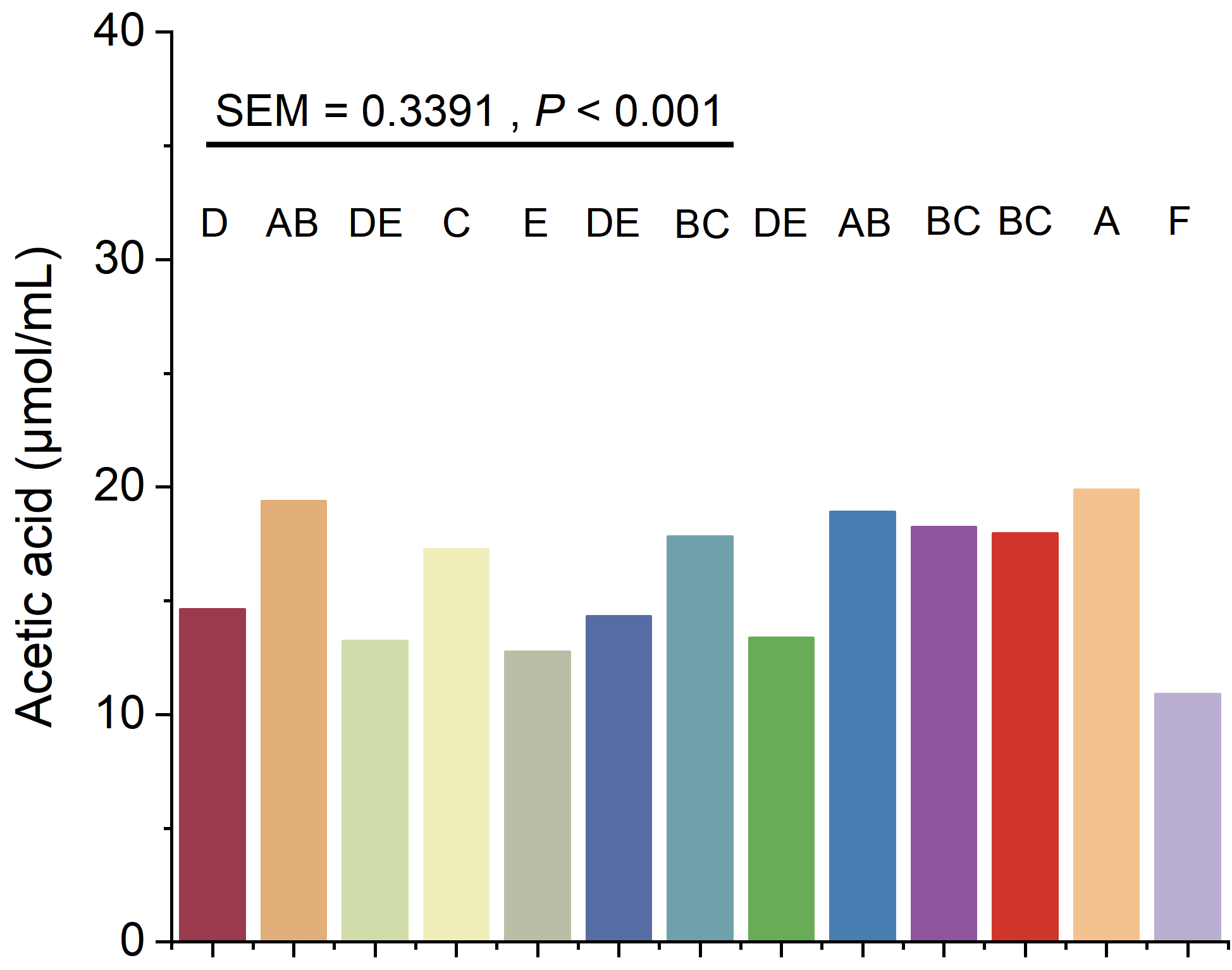

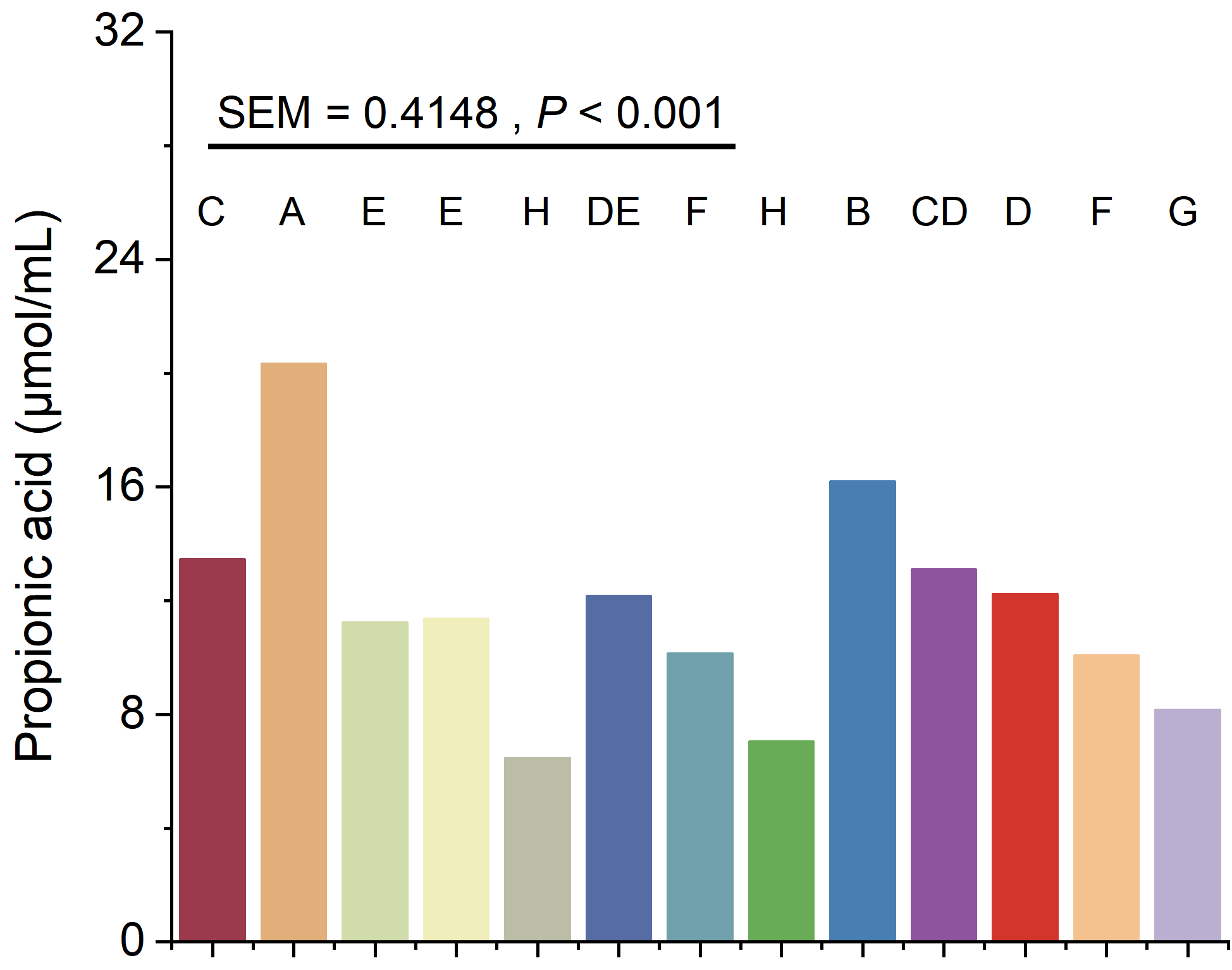

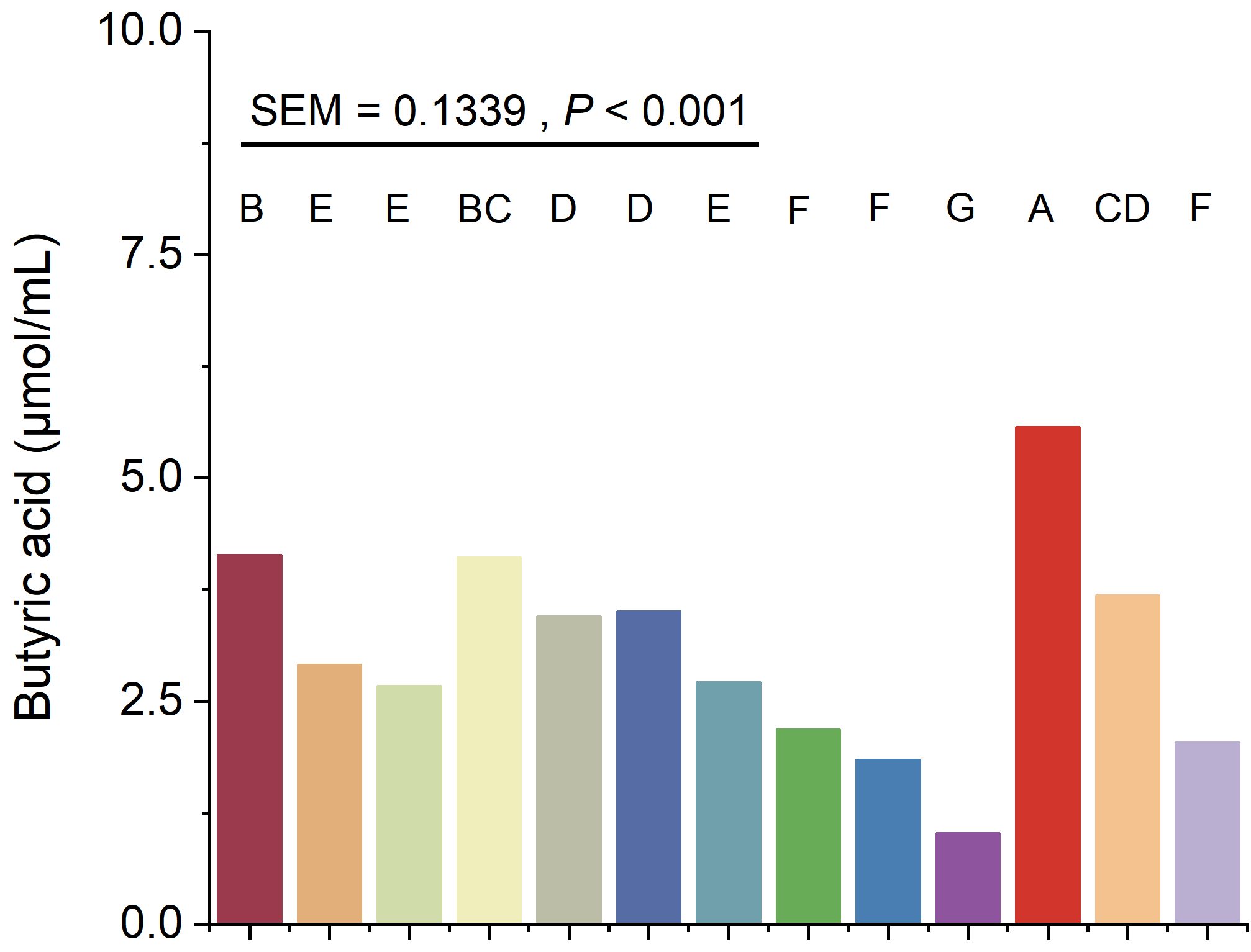


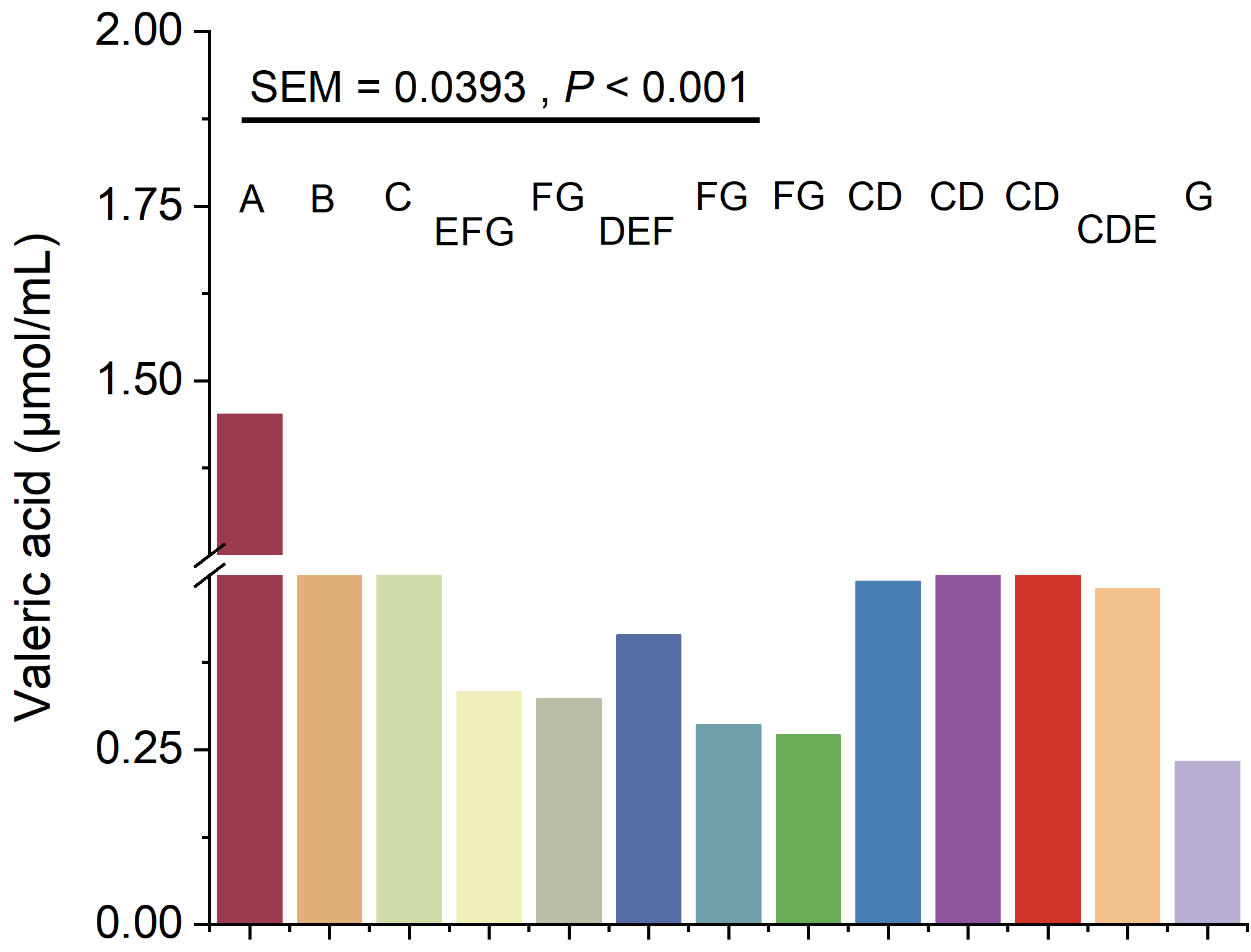

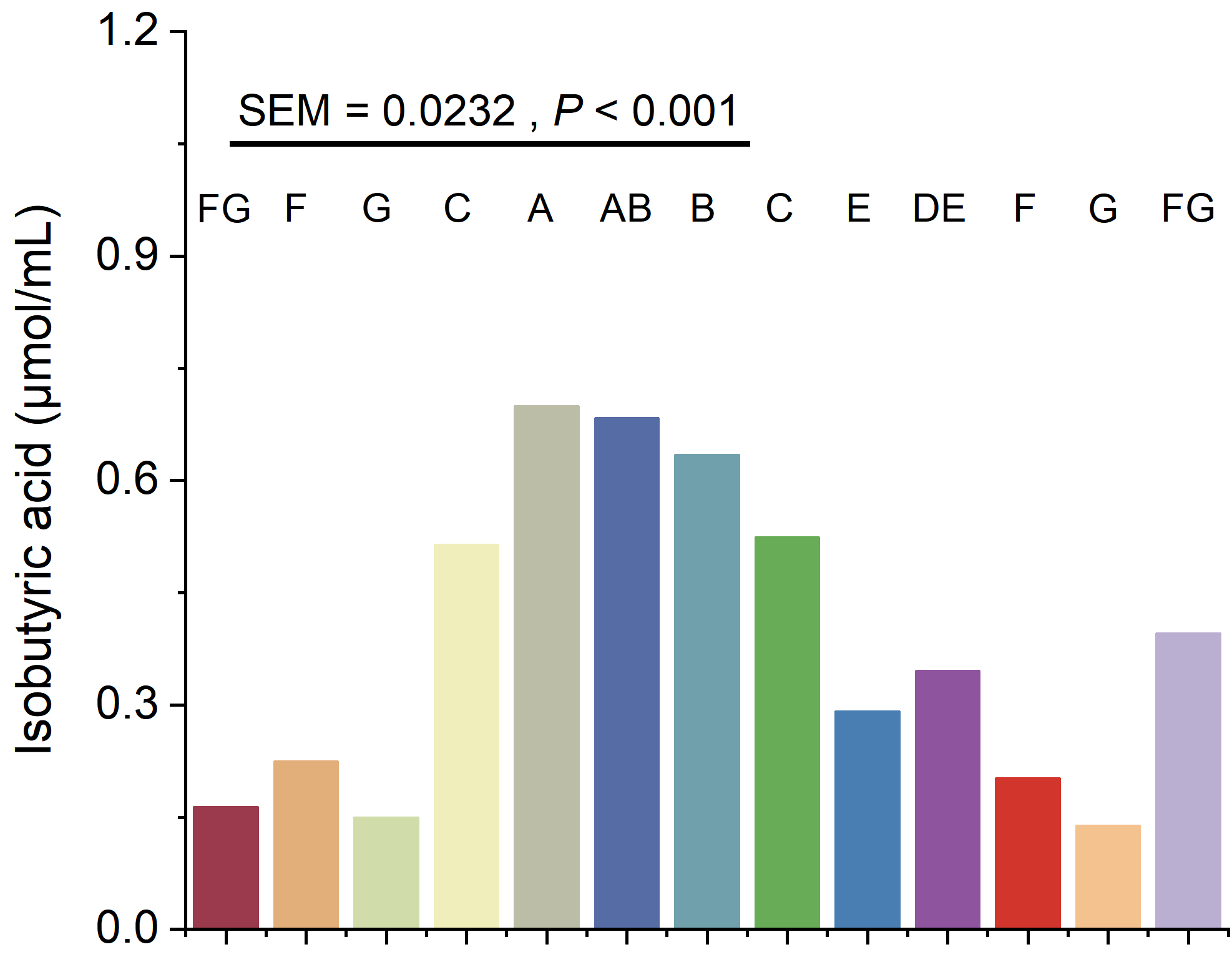

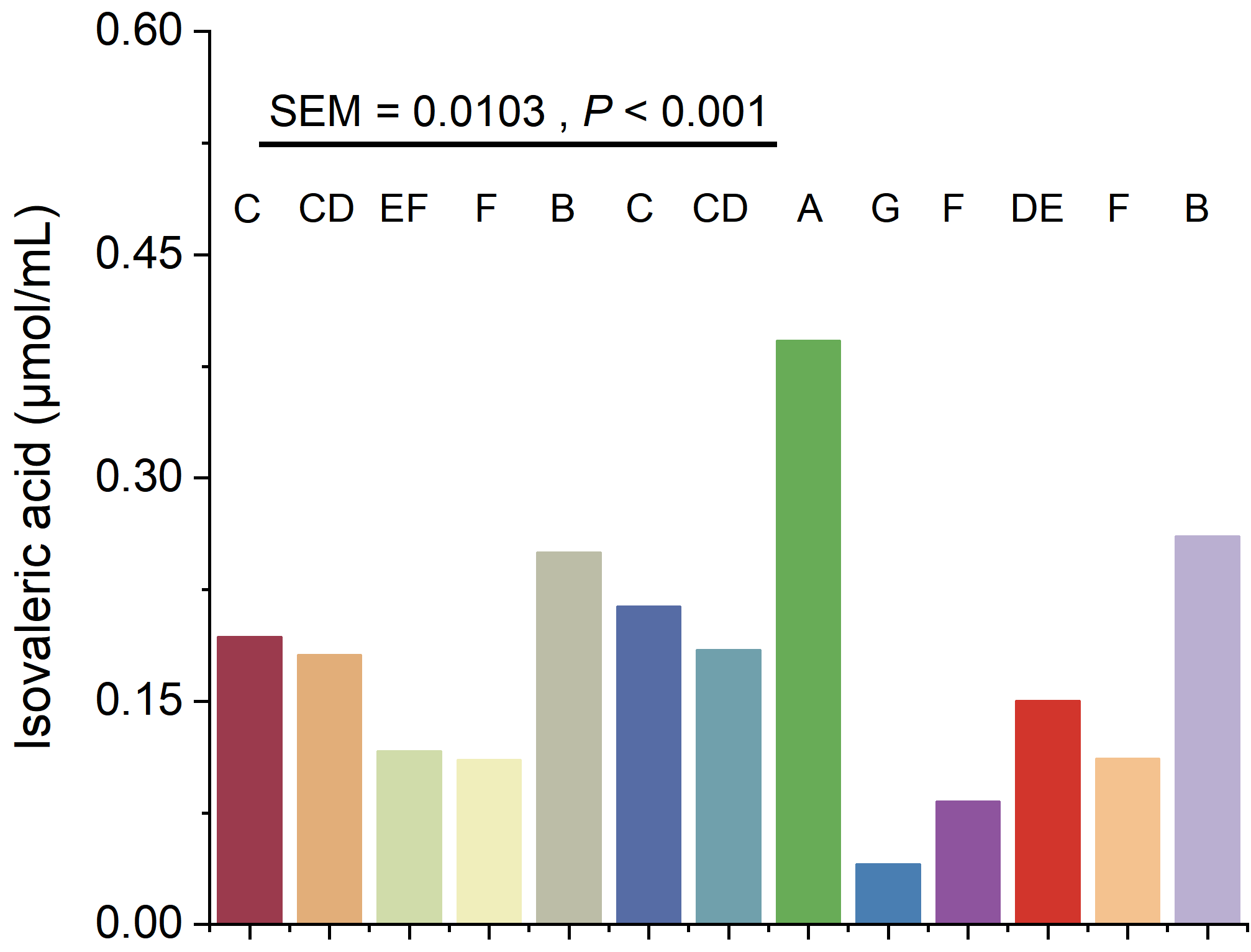


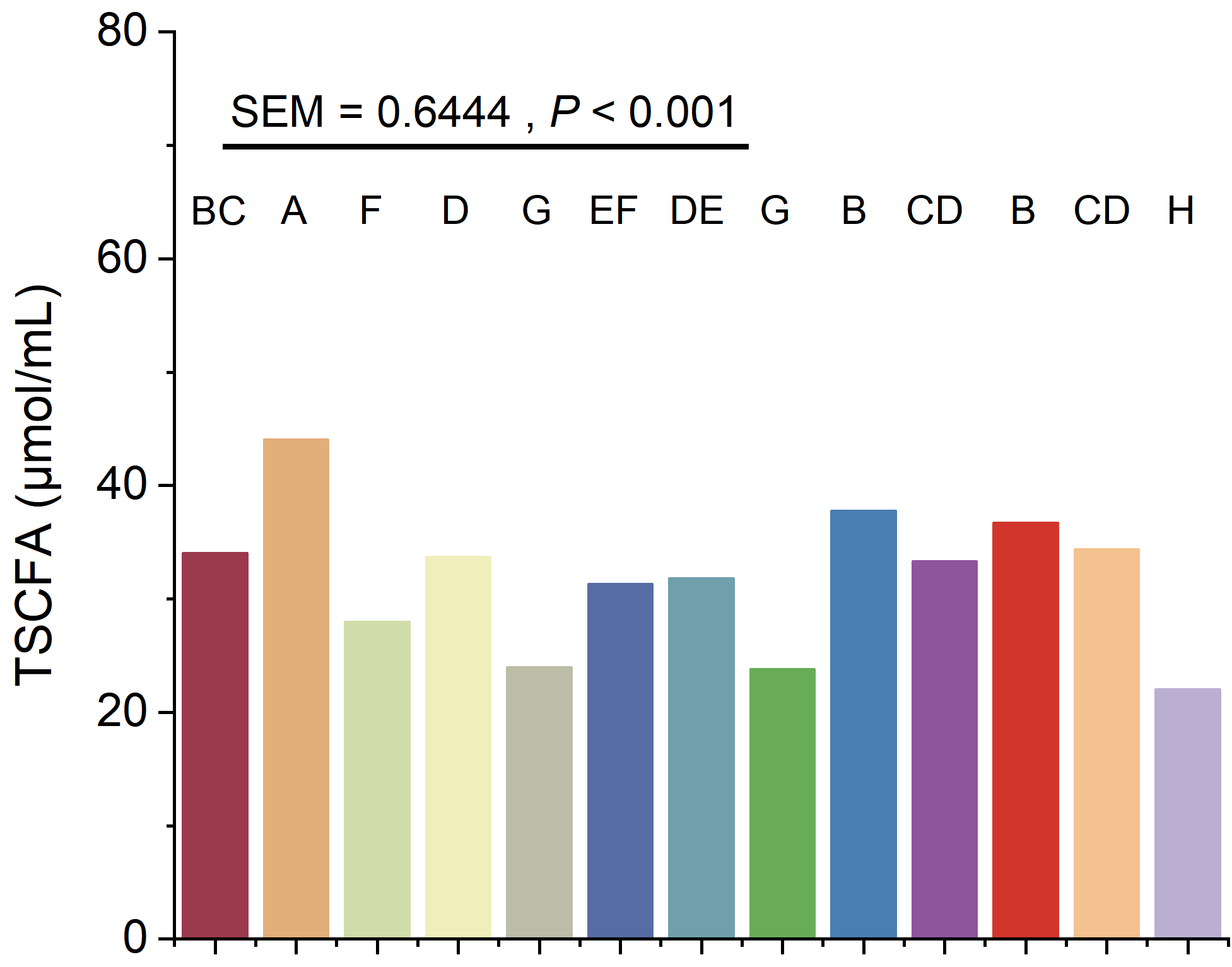

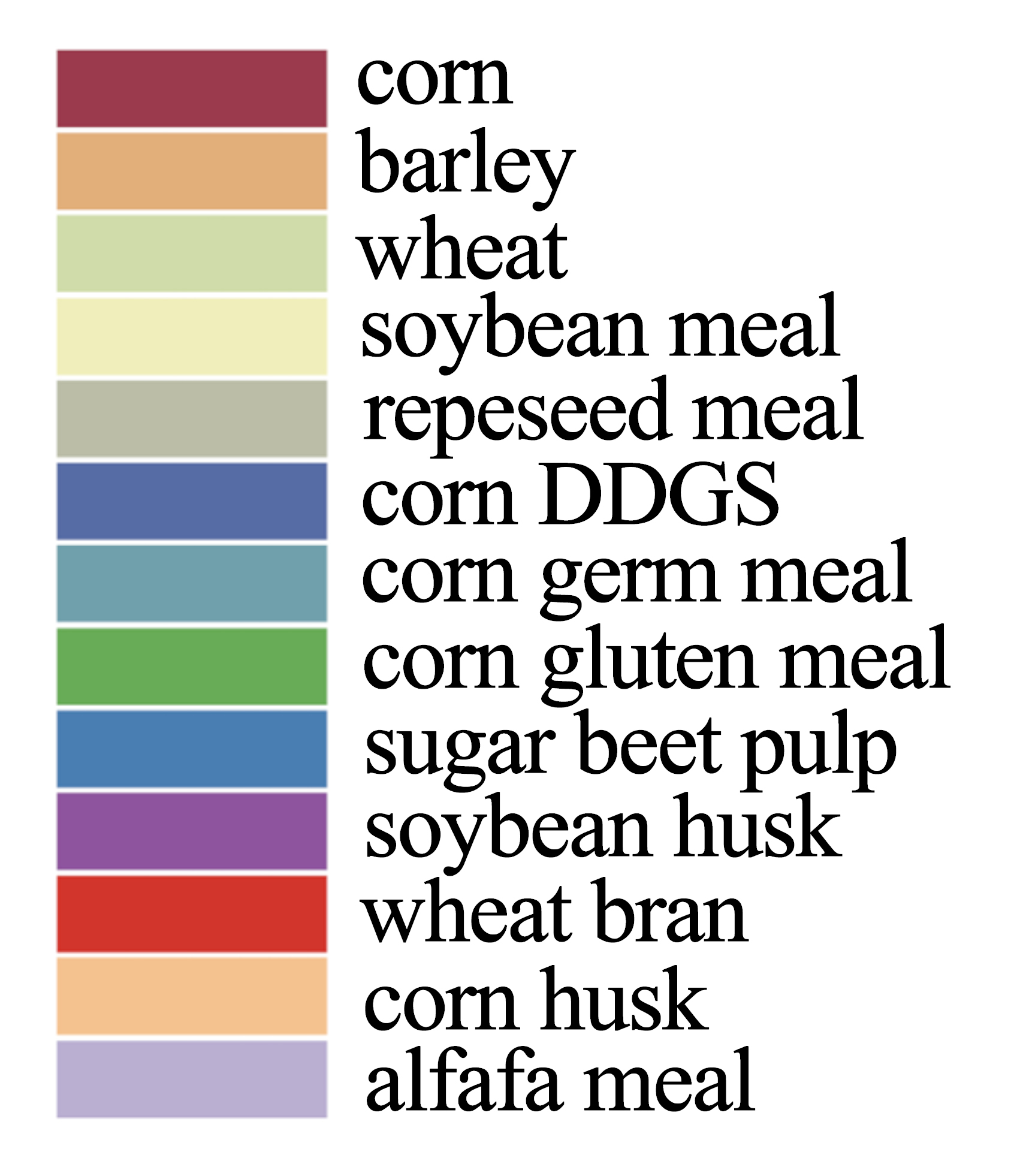


**Fig. S1** Production of SCFAs at various time points during in vitro fermentation of different feedstuffs

Note: Bar graph of the production of acetic acid, propionic acid, butyric acid, valeric acid, isobutyric acid, isovaleric acid, and total short-chain fatty acids (TSCFA) from different feedstuffs after in vitro fermentation for (A) 0 h; (B) 3 h; (C) 6 h; (D) 9 h; (E) 12 h; (F) 15 h; (G) 18 h; (H) 21 h; (I) 24 h; (J) 36 h; (K) 48 h; (L) 60 h; (M) 72 h. The differences between the bars with different uppercase letters are extremely significant (*P* < 0.01; *n* = 6).

A B C

D E F

G H I

J K L


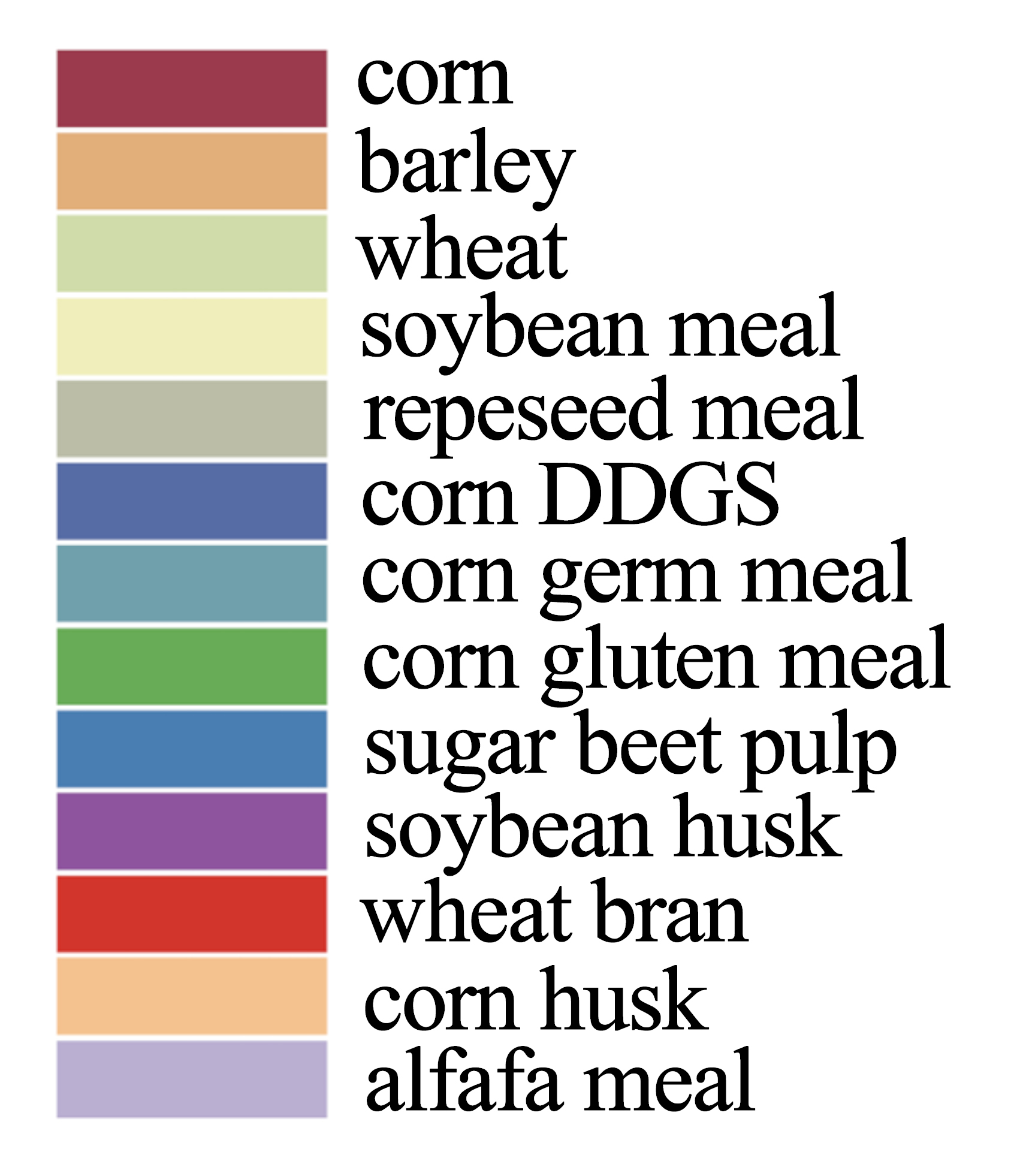


**Fig. S2** Gas production at various time points during in vitro fermentation of different feedstuffs Note: Bar graph of dry matter correction gas production (DMCV) from different feedstuffs after in vitro fermentation for (A) 3 h; (B) 6 h; (C) 9 h; (D) 12 h; (E) 15 h; (F) 18 h; (G) 21 h; (H) 24 h; (I) 36 h; (J) 48 h; (K) 60 h; (L) 72 h. The differences between the bars with different uppercase letters are extremely significant (*P* < 0.01; *n* = 6).
